# Ocean Species Discoveries 1–12 — A primer for accelerating marine invertebrate taxonomy

**DOI:** 10.3897/BDJ.12.e128431

**Published:** 2024-08-06

**Authors:** Senckenberg Ocean Species Alliance (SOSA), Angelika Brandt, Chong Chen, Laura Engel, Patricia Esquete, Tammy Horton, Anna M. Jażdżewska, Nele Johannsen, Stefanie Kaiser, Terue C. Kihara, Henry Knauber, Katharina Kniesz, Jannes Landschoff, Anne-Nina Lörz, Fabrizio M. Machado, Carlos A. Martínez-Muñoz, Torben Riehl, Amanda Serpell-Stevens, Julia D. Sigwart, Anne Helene S. Tandberg, Ramiro Tato, Miwako Tsuda, Katarzyna Vončina, Hiromi K. Watanabe, Christian Wenz, Jason D. Williams

**Affiliations:** 1 Senckenberg Research Institute and Natural History Museum Frankfurt, Department of Marine Zoology, Senckenberganlage 25, 60325, Frankfurt am Main, Germany Senckenberg Research Institute and Natural History Museum Frankfurt, Department of Marine Zoology, Senckenberganlage 25, 60325 Frankfurt am Main Germany; 2 Johann Wolfgang Goethe University Frankfurt, Department of Biological Sciences, Institute of Ecology, Evolution and Diversity, Max-von-Laue-Str. 13, 60438, Frankfurt am Main, Germany Johann Wolfgang Goethe University Frankfurt, Department of Biological Sciences, Institute of Ecology, Evolution and Diversity, Max-von-Laue-Str. 13, 60438 Frankfurt am Main Germany; 3 X-STAR, Japan Agency for Marine-Earth Science and Technology (JAMSTEC), 2–15 Natsushima-cho, 237-0061, Yokosuka, Kanagawa, Japan X-STAR, Japan Agency for Marine-Earth Science and Technology (JAMSTEC), 2–15 Natsushima-cho, 237-0061 Yokosuka, Kanagawa Japan; 4 Institute of Marine Ecosystem and Fishery Science (IMF) Center for Earth System Research and Sustainability (CEN) University of Hamburg, Große Elbstraße 133, 22767, Hamburg, Germany Institute of Marine Ecosystem and Fishery Science (IMF) Center for Earth System Research and Sustainability (CEN) University of Hamburg, Große Elbstraße 133, 22767 Hamburg Germany; 5 Departamento de Biologia & CESAM (Centro de estudos do Ambiente e do Mar), Universidade de Aveiro, Aveiro, Portugal Departamento de Biologia & CESAM (Centro de estudos do Ambiente e do Mar), Universidade de Aveiro Aveiro Portugal; 6 National Oceanography Centre, Southampton, United Kingdom National Oceanography Centre Southampton United Kingdom; 7 University of Lodz, Faculty of Biology and Environmental Protection, Department of Invertebrate Zoology and Hydrobiology, Banacha 12/16, 90-237, Łódź, Poland University of Lodz, Faculty of Biology and Environmental Protection, Department of Invertebrate Zoology and Hydrobiology, Banacha 12/16, 90-237 Łódź Poland; 8 Scharnhorststraße 44, 21335, Lüneburg, Germany Scharnhorststraße 44, 21335 Lüneburg Germany; 9 Integrated Environmental Solutions UG—INES, c/o DZMB, Südstrand 44, 26382, Wilhelmshaven, Germany Integrated Environmental Solutions UG—INES, c/o DZMB, Südstrand 44, 26382 Wilhelmshaven Germany; 10 Leibniz Institute for Baltic Sea Research Warnemünde, Seestraße 15, 18119, Rostock, Germany Leibniz Institute for Baltic Sea Research Warnemünde, Seestraße 15, 18119 Rostock Germany; 11 German Centre for Marine Biodiversity Research (DZMB), Senckenberg am Meer, Südstrand 44, 26382, Wilhelmshaven, Germany German Centre for Marine Biodiversity Research (DZMB), Senckenberg am Meer, Südstrand 44, 26382 Wilhelmshaven Germany; 12 Sea Change Trust, Cape Town, Western Cape, South Africa Sea Change Trust Cape Town, Western Cape South Africa; 13 Department of Botany and Zoology, Department of Botany and Zoology, Stellenbosch University, Private Bag X1, 7602, Matieland, South Africa Department of Botany and Zoology, Department of Botany and Zoology, Stellenbosch University, Private Bag X1, 7602 Matieland South Africa; 14 Institute of Biology, Universidade Estadual de Campinas, 13083-970, Campinas, São Paulo, Brazil Institute of Biology, Universidade Estadual de Campinas, 13083-970 Campinas, São Paulo Brazil; 15 University Museum of Bergen, University of Bergen, Bergen, Norway University Museum of Bergen, University of Bergen Bergen Norway; 16 Estación de Bioloxía Mariña de A Graña, Universidade de Santiago de Compostela, A Coruña, Spain Estación de Bioloxía Mariña de A Graña, Universidade de Santiago de Compostela A Coruña Spain; 17 Project Team for Development of New-Generation Research Protocol for Submarine Resources, Japan Agency for Marine-Earth Science and Technology (JAMSTEC), 2–15 Natsushima-cho, 237-0061, Yokosuka, Kanagawa, Japan Project Team for Development of New-Generation Research Protocol for Submarine Resources, Japan Agency for Marine-Earth Science and Technology (JAMSTEC), 2–15 Natsushima-cho, 237-0061 Yokosuka, Kanagawa Japan; 18 Department of Biology, Hofstra University, 11549-1140, Hempstead, New York, United States of America Department of Biology, Hofstra University, 11549-1140 Hempstead, New York United States of America

**Keywords:** new species, shelf-life, Mollusca, Arthropoda, Echinodermata, alpha taxonomy, taxonomic bottleneck, biodiversity data, deep sea, estuary, hydrothermal vent

## Abstract

**Background:**

Discoveries of new species often depend on one or a few specimens, leading to delays as researchers wait for additional context, sometimes for decades. There is currently little professional incentive for a single expert to publish a stand-alone species description. Additionally, while many journals accept taxonomic descriptions, even specialist journals expect insights beyond the descriptive work itself. The combination of these factors exacerbates the issue that only a small fraction of marine species are known and new discoveries are described at a slow pace, while they face increasing threats from accelerating global change. To tackle this challenge, this first compilation of *Ocean Species Discoveries* (OSD) presents a new collaborative framework to accelerate the description and naming of marine invertebrate taxa that can be extended across all phyla. Through a mode of publication that can be speedy, taxonomy-focused and generate higher citation rates, OSD aims to create an attractive home for single species descriptions. This *Senckenberg Ocean Species Alliance* (SOSA) approach emphasises thorough, but compact species descriptions and diagnoses, with supporting illustrations and with molecular data when available. Even basic species descriptions carry key data for distributions and ecological interactions (e.g., host-parasite relationships) besides universally valid species names; these are essential for downstream uses, such as conservation assessments and communicating biodiversity to the broader public.

**New information:**

This paper presents thirteen marine invertebrate taxa, comprising one new genus, eleven new species and one re-description and reinstatement, covering wide taxonomic, geographic, bathymetric and ecological ranges. The taxa addressed herein span three phyla (Mollusca, Arthropoda, Echinodermata), five classes, eight orders and twelve families. Apart from the new genus, an updated generic diagnosis is provided for four other genera. The newly-described species of the phylum Mollusca are *Placiphorellamethanophila* Vončina, **sp. nov.** (Polyplacophora, Mopaliidae), *Lepetodrilusmarianae* Chen, Watanabe & Tsuda, **sp. nov.** (Gastropoda, Lepetodrilidae), *Shinkailepasgigas* Chen, Watanabe & Tsuda, **sp. nov.** (Gastropoda, Phenacolepadidae) and *Lyonsiellaillaesa* Machado & Sigwart, **sp. nov.** (Bivalvia, Lyonsiellidae). The new taxa of the phylum Arthropoda are all members of the subphylum Crustacea: *Lepechinellanaces* Lörz & Engel, **sp. nov.** (Amphipoda, Lepechinellidae), *Cuniculomaeragrata* Tandberg & Jażdżewska, **gen. et sp. nov.** (Amphipoda, Maeridae), *Pseudionellapumulaensis* Williams & Landschoff, **sp. nov.** (Isopoda, Bopyridae), *Mastigoniscusminimus* Wenz, Knauber & Riehl, **sp. nov.** (Isopoda, Haploniscidae), *Macrostylispapandreas* Jonannsen, Riehl & Brandt, **sp. nov.** (Isopoda, Macrostylidae), *Austroniscusindobathyasellus* Kaiser, Kniesz & Kihara, **sp. nov.** (Isopoda, Nannoniscidae) and *Apseudopsisdaria* Esquete & Tato, **sp. nov.** (Tanaidacea, Apseudidae). In the phylum Echinodermata, the reinstated species is *Psychropotesbuglossa* E. Perrier, 1886 (Holothuroidea, Psychropotidae).

The study areas span the North and Central Atlantic Ocean, the Indian Ocean and the North, East and West Pacific Ocean and depths from 5.2 m to 7081 m. Specimens of eleven free-living and one parasite species were collected from habitats ranging from an estuary to deep-sea trenches. The species were illustrated with photographs, line drawings, micro-computed tomography, confocal laser scanning microscopy and scanning electron microscopy images. Molecular data are included for nine species and four species include a molecular diagnosis in addition to their morphological diagnosis.

The five new geographic and bathymetric distribution records comprise *Lepechinellanaces* Lörz & Engel, **sp. nov.**, *Cuniculomaeragrata* Tandberg & Jażdżewska, **sp. nov.**, *Pseudionellapumulaensis* Williams & Landschoff, **sp. nov.**, *Austroniscusindobathyasellus* Kaiser, Kniesz & Kihara, **sp. nov.** and *Psychropotesbuglossa* E. Perrier, 1886, with the novelty spanning from the species to the family level. The new parasite record is *Pseudionellapumulaensis* Williams & Landschoff, **sp. nov.**, found in association with the hermit crab *Pagurusfraserorum* Landschoff & Komai, 2018.

## Introduction

Two-thirds of the Earth’s surface are covered by a largely unexplored ocean, harbouring vast biodiversity and threatened by the impacts of human activities (Ramirez-Llodra 2020). Unlike terrestrial species richness, dominated by Arthropoda, marine environments boast diversity across numerous phyla (Glover et al. 2009). To date, taxonomists have described approximately 242,000 marine species living in the world's oceans (Bouchet et al. 2023), a small fraction of perhaps two million total living marine species (Mora et al. 2011). New marine species continue to be published at average rates in the range of 2,000 species per year (marine eukaryotes, Appeltans et al. (2012)) to 2,332 species per year (marine biota, Bouchet et al. (2023)). However, the process of taxonomic description is famously slow: the delay from discovery to description is on average 20–40 years, resulting in a discovery to publication time measured in decades (e.g., Goodwin et al. 2020). The challenge to describe the species of the Earth’s oceans represents interconnected problems of diversity, scale and speed, as well as prioritisation and resourcing on the background of accelerating global change.

Innovative strategies are vital to expedite species description and naming, leveraging international collaboration, data mobilisation, new technologies and accessibility (Wheeler et al. 2012, Orr et al. 2021, Sigwart et al. 2023). Some discipline-specific challenges included a cultural dominance of single- or few-author taxonomic papers and a well-known problem of journal-based metrics that under-represent taxonomic contributions (Löbl et al. 2023). The time lag between discovery and description has been quantified in several studies especially on flowering plants, as well as further delays involved in gaining practical expertise with particular taxa (Goodwin et al. 2020). The motivation to have a more complete story to tell about new species, in the context of ecological or phylogenetic insights, is one factor that causes the decades-long process to publish new species descriptions. While there are numerous journals that are appropriate venues to publish new taxonomic descriptions, even specialist taxonomic journals have editorial guidelines that require discussion or insights beyond the descriptive work itself. The publication of an initial discovery is delayed, while sometimes waiting decades for additional context. In the current publishing landscape, there is much labour and little incentive involved for a single expert to publish a stand-alone species description.

To tackle these challenges, various initiatives have been developed to accelerate and expand the scope of taxonomic studies. In the mycological community, a publication series called *Fungal Diversity Notes* (e.g., Liu et al. 2015, Li et al. 2016, Hyde et al. 2020, Boonmee et al. 2021) exemplifies successful collaborative efforts in taxonomy, describing over 1500 new taxa since 2015. It is this project that has inspired the present approach to marine species: The *Senckenberg Ocean Species Alliance* (SOSA) has launched the *Ocean Species Discoveries* (OSD) platform to enable efficient high-quality taxonomic descriptions of marine invertebrate taxa and to increase the incentive for taxonomists to carry out taxonomic work swiftly and with high throughput.

Here, species are presented as a collection of short, concise, but complete taxonomic descriptions, without requiring a specific overarching taxonomic or ecological theme. While each species authority is clearly credited to the specific contributor(s), the authorship of the article includes all contributors. This presents several advantages: (1) the descriptions can be prepared rapidly and further findings published separately in due course; (2) large-scale collaborative article authorship means that authors are cited for any of the species included, such that the entire consortium receives a citation, not just the respective species' authors; (3) there is a standardised look and feel to the descriptions, across diverse taxa, which make the descriptive information more accessible; and (4) once the style is established, the streamlined and simplified publication process means the descriptions are published quickly. These points underlie the success of the *Fungal Diversity Notes* series, which we hope to emulate in the marine realm. This inaugural contribution sets the stage for future editions dedicated to these goals.

With this paper, we provide descriptions of twelve new marine invertebrate taxa and one re-validated taxon, with supporting morphological and — where available — molecular evidence. On average, the 11 new species described herein were named 7 years after their initial discovery, with a shelf-life (Fontaine et al. 2012) of 21 years maximum and 1 year minimum. Our 7-year average is shorter than the 13.5-year average for marine species reported by Bouchet et al. (2023), while matching their 7-year median shelf-life.

We have adopted relatively new approaches, such as including explicit molecular diagnoses where feasible, but the format remains flexible; this should speed up species descriptions without losing quality (Renner 2016, Vences 2020). This first contribution serves as a methodological reference and as a starting point for an entire series dedicated to thorough and rapid description of novel marine invertebrate taxa.

The Anthropocene is characterised by alarming rates of species extinction (Wiens and Zelinka 2024), that brings an urgency to the mission to explore and document Earth’s species. Recent efforts have emphasised the advantages of globally united efforts to study the oceans (Howell et al. 2020). This includes taxonomy. The ambitious goal of describing 1.8 million ocean species is achievable, if we can leverage the collective strengths of global progress, expertise and technological advancements.

**Outlook**

In response to the pressing need for expedited taxonomic efforts, OSD presents a novel approach to species description in the marine realm. This is not bound by a geographic region or any *a priori* consortium and focuses solely on establishing nomenclature. This inaugural contribution underscores the importance and feasibility of concise, yet thorough integrative taxonomic descriptions. As OSD gains traction, there is immense potential for growth, with the possibility of including more species per edition, the development of marine-taxonomist consortia and even replication beyond the confines of the SOSA project. By fostering collaboration and accessibility, OSD is poised to catalyse advancements in marine biodiversity research, offering a valuable resource for scientists and conservationists worldwide to explore and protect our oceans' rich ecosystems.

### Summary of contents

**Classification of the taxa (re-)described in this article**

**Phylum Mollusca Linnaeus, 1758**

Class Polyplacophora Gray, 1821

Subclass Neoloricata Bergenhayn, 1955

Order Chitonida Thiele, 1909

Suborder Acanthochitonina Bergenhayn, 1930

Superfamily Mopalioidea Dall, 1889

Family Mopaliidae Dall, 1889

Genus *Placiphorella* Carpenter in Dall, 1879

**1.** *Placiphorellamethanophila* Voncina, **sp. nov.** (contributed by Katarzyna Voncina)

Class Gastropoda Cuvier, 1795

Subclass Vetigastropoda Salvini-Plawen, 1980

Order Lepetellida Moskalev, 1971

Superfamily Lepetodriloidea McLean, 1988

Family Lepetodrilidae McLean, 1988

Genus *Lepetodrilus* McLean, 1988

**2.** *Lepetodrilusmarianae* Chen, Watanabe & Tsuda, **sp. nov.** (contributed by Chong Chen, Hiromi Kayama Watanabe and Miwako Tsuda)

Subclass Neritimorpha Koken, 1896

Order Cycloneritida Fryda, 1998, nom. emend. Bouchet et al. (2017)

Superfamily Neritoidea Rafinesque, 1815

Family Phenacolepadidae Pilsbry, 1895

Genus *Shinkailepas* Okutani, Saito & Hashimoto, 1989

**3.** *Shinkailepasgigas* Chen, Watanabe & Tsuda, **sp. nov.** (contributed by Chong Chen, Hiromi Kayama Watanabe and Miwako Tsuda)

Class Bivalvia Linnaeus, 1758

Superorder Anomalodesmata Dall, 1889

Order Poromyida Ridewood, 1903

Superfamily Verticordioidea Stoliczka, 1870

Family Lyonsiellidae Dall, 1895

Subfamily Lyonsiellinae Dall, 1895

Genus *Lyonsiella* G. O. Sars, 1872

**4.** *Lyonsiellaillaesa* Machado & Sigwart, **sp. nov.** (contributed by Fabrizio Marcondes Machado and Julia Sigwart)

**Phylum Arthropoda Gravenhorst, 1843** (auct. emend., see Martínez-Muñoz 2023)

Subphylum Crustacea Brunnich, 1772

Class Malacostraca Latreille, 1802

Superorder Peracarida Calman, 1904

Order Amphipoda Latreille, 1816

Family Lepechinellidae Schellenberg, 1926

Genus *Lepechinella* Stebbing, 1908

**5.** *Lepechinellanaces* Lorz & Engel, **sp. nov.** (contributed by Anne-Nina Lorz and Laura Engel)

Family Maeridae Krapp-Schickel, 2008

Genus *Cuniculomaera* Tandberg & Jazdzewska, **gen. nov.** (contributed by Anne Helene S. Tandberg and Anna M. Jazdzewska)

**6.** *Cuniculomaeragrata* Tandberg & Jazdzewska, **sp. nov.** (contributed by Anne Helene S. Tandberg and Anna M. Jazdzewska)

Order Isopoda Latreille, 1816

Family Bopyridae Rafinesque, 1815

Subfamily Pseudioninae R. Codreanu, 1967

Genus *Pseudionella* Shiino, 1949

**7.** *Pseudionellapumulaensis* Williams & Landschoff, **sp. nov.** (contributed by Jason D. Williams and Jannes Landschoff)

Family Haploniscidae Hansen, 1916

Genus *Mastigoniscus* Lincoln, 1985

**8.** *Mastigoniscusminimus* Wenz, Knauber & Riehl, **sp. nov.** (contributed by Christian Wenz, Henry Knauber and Torben Riehl)

Family Macrostylidae Hansen, 1916

Genus *Macrostylis* G.O. Sars, 1864

**9.** *Macrostylispapandreas* Johannsen, Riehl & Brandt, **sp. nov.** (contributed by Nele Johannsen, Torben Riehl and Angelika Brandt)

Family Nannoniscidae Hansen, 1916

Genus *Austroniscus* Vanhoffen, 1914

**10.** *Austroniscusindobathyasellus* Kaiser, Kniesz & Kihara, **sp. nov.** (contributed by Stefanie Kaiser, Katharina Kniesz and Terue C. Kihara)

Order Tanaidacea Dana, 1849

Family Apseudidae Leach, 1814

Genus *Apseudopsis* Norman, 1899

**11.** *Apseudopsisdaria* Esquete & Tato, **sp. nov.** (contributed by Patricia Esquete and Ramiro Tato)

**Phylum Echinodermata Bruguiere, 1791 (ex Klein, 1734)**

Class Holothuroidea Blainville, 1834

Order Elasipodida Theel, 1882

Family Psychropotidae Theel, 1882

Genus *Psychropotes* Theel, 1882

**12.** *Psychropotesbuglossa* E. Perrier, 1886, **revived status** (contributed by Amanda Serpell-Stevens, Tammy Horton and Julia Sigwart)

**New geographical distributions**

Five out of twelve species supported new geographical distributions (including depth) at different taxonomic levels.

The genus *Lepechinella* Stebbing, 1908 (Crustacea, Amphipoda, Lepechinellidae) is known from the North Atlantic by 13 species (Lörz et al. 2020). Here the first species collected in the abyssal depth of the Atlantic is described, *Lepechinellanaces* Lorz & Engel, **sp. nov.**

The family Maeridae Krapp-Schickel, 2008 (Crustacea, Amphipoda) and *Cuniculomaera* Tandberg & Jazdzewska, **gen. nov.** are reported for the first time from the deep parts of the Bering Sea, based on *Cuniculomaeragrata* Tandberg & Jazdzewska, **sp. nov.** At 3416 m depth, the type locality is the deepest record of any Maeridae.

The genus *Pseudionella* Shiino, 1949 (Crustacea, Isopoda, Bopyridae) is first reported from the Indian Ocean, based on *Pseudionellapumulaensis* Williams & Landschoff, **sp. nov.**

The family Nannoniscidae Hansen, 1916 (Crustacea, Isopoda) and the genus *Austroniscus* Vanhoffen, 1914 are first reported from the Indian Ocean, based on *Austroniscusindobathyasellus* Kaiser, Kniesz & Kihara, **sp. nov.**

*Psychropotesbuglossa* E. Perrier, 1886 (Echinodermata, Holothuroidea, Elasipodida, Elasipodidae) is reported for the first time from the Porcupine Abyssal Plain, northeast Atlantic Ocean, 4840–4629 m depth.

**New host records**

The undescribed species of *Pseudionella* Shiino, 1949 (Crustacea, Isopoda, Bopyridae) mentioned by Landschoff et al. (2018) from its host *Pagurusfraserorum* Landschoff & Komai in Landschoff et al. 2018 (Crustacea, Decapoda, Anomura, Paguridae) is herein described as *Pseudionellapumulaensis* Williams & Landschoff, **sp. nov.**

## Materials and methods

### General methods

The structure of this publication follows the journal template with additional inspiration from similar taxonomic projects. For example, the abstract of this publication is a combination of the Biodiversity Data Journal template, which includes a "Background" and a "New information" subsection, with the structure followed in the *Fungal Diversity Notes* series (e.g., Jayawardena et al. 2023). As a note on the text, here we do follow Meier (2016) as to the "why, when, what and what not" of citing taxonomic publications, meaning all taxonomic authorities and dates are provided, but only relevant citations have been added to the bibliography.

Taxa described in this study were collected from the North and Central Atlantic Ocean, the Indian Ocean and the North, East and West Pacific Ocean and depths from 5.2 m to 7081 m (Suppl. material 1). Specimens of eleven free-living and one parasite species were obtained from the following habitats: estuary, rocky subtidal coral reef, continental shelf methane seeps, deep-sea hydrothermal vents, abyssal fracture zones, abyssal plains and deep-sea trenches; from both soft and hard substrates and from a living host in the case of the parasite (Suppl. material 1). Specimen data are provided separately within each taxon treatment.

Data acquisition methods are briefly described in each taxon treatment. Species were illustrated with photographs, line drawings, micro-computed tomography, confocal laser scanning microscopy and/or scanning electron microscopy images. Molecular data were included for nine of the twelve species and four species include an explicit molecular diagnosis in addition to a morphological diagnosis. From the twelve genus-level taxa included, five had morphological diagnoses updated (this count includes one new genus).

Among all descriptions of crustacean taxa herein, abbreviations of important morphological terminology were standardised: A1 – Antenna 1/antennula; A2 – Antenna 2; Acc flag – Accessory flagellum; Art – Article (of antennae and legs); C – Coxa; Ceph – Cephalothorax; Ch – Cheliped (in Tanaidacea only); Ep – Epimeral plate; Lbi – Labium; Lbr – Labrum; Md – Mandible; Mx1 – Maxilla 1/maxillula; Mx2 – Maxilla 2; Mxp – Maxilliped; Op – Operculum; P – Pereopod; Pl – Pleomere/pleonite; Plp – Pleopod; Plt – Pleotelson; Prn – Pereomere/pereonite; T – Telson; U – Uropod.

## Taxon treatments

### Placiphorella methanophila

Vončina sp. nov.

46AD2093-E0F8-5D5A-80D1-C730475B984A

2281755E-885A-4201-A3EB-71ACBE9E0B5A

*Placiphorella* sp. nov.: Sellanes et al. 2004: 1066.
*Placiphorellaatlantica*: Sellanes et al. 2008: 1105, non *Placiphorellaatlantica* (A. E. Verrill & S. I. Smith in Verrill, 1882).
*Placiphorella* sp.: Schwabe and Sellanes 2010: 47, Figs. 14 I–J, 15.
*Placiphorella* sp.: Schwabe 2010: 172, Fig. 1B, 174: Fig. 3B, 188: Fig. 18A.

#### Materials

- **Type status:** Holotype. **Occurrence:** catalogNumber: ZSM Mol 20041044; recordedBy: Vessel R/V Kay Kay, leg. Javier Sellanes Lopez; individualCount: 1; lifeStage: adult; preparations: EtOH 75%, partly disarticulated (ZSM Mol 20041044) | SEM stubs with parts of girdle, precephalic lappet and radula (ZSM Mol 20220314); otherCatalogNumbers: ZSM Mol 20220314; occurrenceID: 9A562FEA-D154-5A2D-9909-D3C42E9E754A; **Taxon:** scientificName: *Placiphorellamethanophila* Vončina; kingdom: Animalia; phylum: Mollusca; class: Polyplacophora; order: Chitonida; family: Mopaliidae; genus: Placiphorella; specificEpithet: *methanophila*; scientificNameAuthorship: Vončina; nomenclaturalCode: ICZN; **Location:** higherGeography: Pacific Ocean; continent: South America; country: Chile; stateProvince: Biobío Region, Concepción Province; locality: off Concepción; minimumDepthInMeters: 870; maximumDepthInMeters: 930; verbatimLatitude: 36°20'60''S; verbatimLongitude: 73°43'60''W; **Identification:** identifiedBy: Enrico Schwabe; dateIdentified: 04/08/2008; **Event:** samplingProtocol: Agassiz trawl (AGT), 1.5 m wide, operated in 20 min. hauls; eventDate: 2003; habitat: shelf margin, on pieces of carbonate crusts; **Record Level:** institutionCode: SNSB-ZSM; collectionCode: Mol; basisOfRecord: PreservedSpecimen
- **Type status:** Paratype. **Occurrence:** catalogNumber: SMF 376539; recordedBy: Vessel R/V Vidal Gormáz, leg. Javier Sellanes Lopez; individualCount: 1; lifeStage: adult; preparations: EtOH 75%, partly disarticulated; otherCatalogNumbers: SMF 376539; occurrenceID: CC1E4F2E-B73F-5B2C-81F1-85EB904472DA; **Taxon:** scientificName: *Placiphorellamethanophila* Vončina; kingdom: Animalia; phylum: Mollusca; class: Polyplacophora; order: Chitonida; family: Mopaliidae; genus: Placiphorella; specificEpithet: *methanophila*; scientificNameAuthorship: Vončina; nomenclaturalCode: ICZN; **Location:** higherGeography: Pacific Ocean; continent: South America; country: Chile; stateProvince: Biobío Region, Concepción Province; locality: off Concepción; maximumDepthInMeters: 922; verbatimLatitude: 36°00.23’S; verbatimLongitude: 73°38.41’W; **Identification:** identifiedBy: Enrico Schwabe; dateIdentified: 04/08/2008; **Event:** samplingProtocol: Agassiz trawl (AGT), 1.5 m wide, operated in 20 min. hauls; eventDate: 04/2007; **Record Level:** institutionCode: SMF; basisOfRecord: PreservedSpecimen
- **Type status:** Paratype. **Occurrence:** catalogNumber: ZSM Mol 20080824; recordedBy: Vessel R/V Vidal Gormáz, leg. Javier Sellanes Lopez; individualCount: 2; lifeStage: adult; preparations: EtOH 95%, a fragment of foot sampled for DNA barcoding; associatedSequences: https://www.ncbi.nlm.nih.gov/nuccore/PP133101; occurrenceID: CA9298E4-5204-5350-ACF9-4CC6547B5887; **Taxon:** scientificName: *Placiphorellamethanophila* Vončina; kingdom: Animalia; phylum: Mollusca; class: Polyplacophora; order: Chitonida; family: Mopaliidae; genus: Placiphorella; specificEpithet: *methanophila*; scientificNameAuthorship: Vončina; nomenclaturalCode: ICZN; **Location:** higherGeography: Pacific Ocean; continent: South America; country: Chile; stateProvince: Biobío Region, Concepción Province; locality: off Concepción; maximumDepthInMeters: 922; verbatimLatitude: 36°00.23’S; verbatimLongitude: 73°38.41’W; **Identification:** identifiedBy: Enrico Schwabe; dateIdentified: 04/08/2008; **Event:** samplingProtocol: Agassiz trawl (AGT), 1.5 m wide, operated in 20 min. hauls; eventDate: 04/2007; **Record Level:** institutionCode: SNSB-ZSM; collectionCode: Mol; basisOfRecord: PreservedSpecimen

#### Description

**Figure 1.**
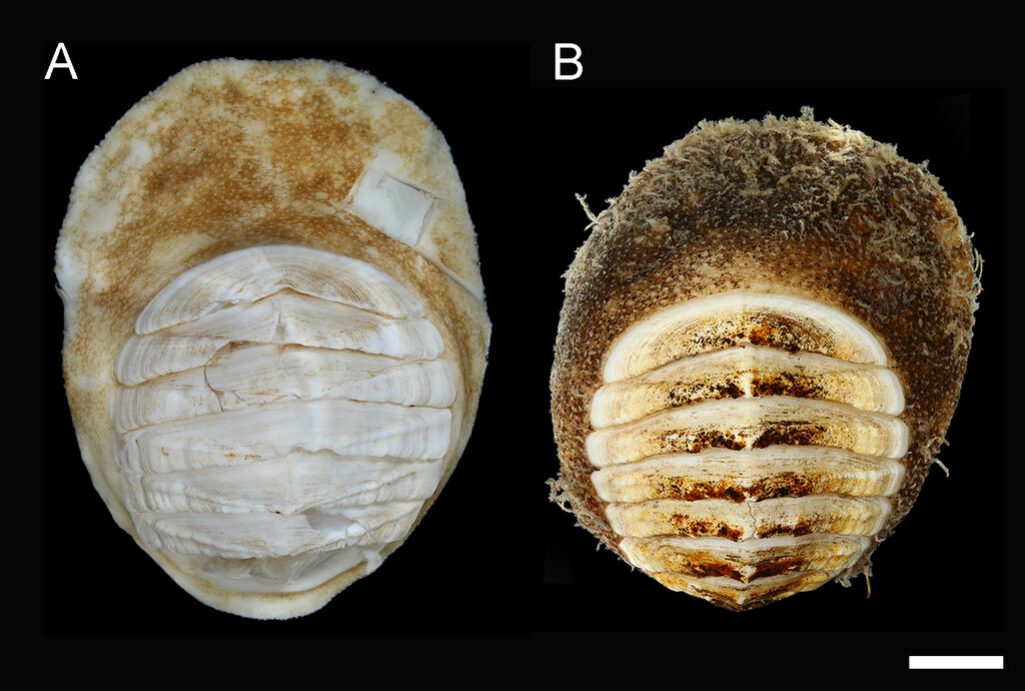
*Placiphorellamethanophila* Vončina, **sp. nov.**, dorsal view. **A** Holotype, ZSM Mol 20041044, body length: 29 mm; **B** Paratype, SMF 376539, body length: 26 mm. Scale bar: 5 mm.

**Figure 2.**
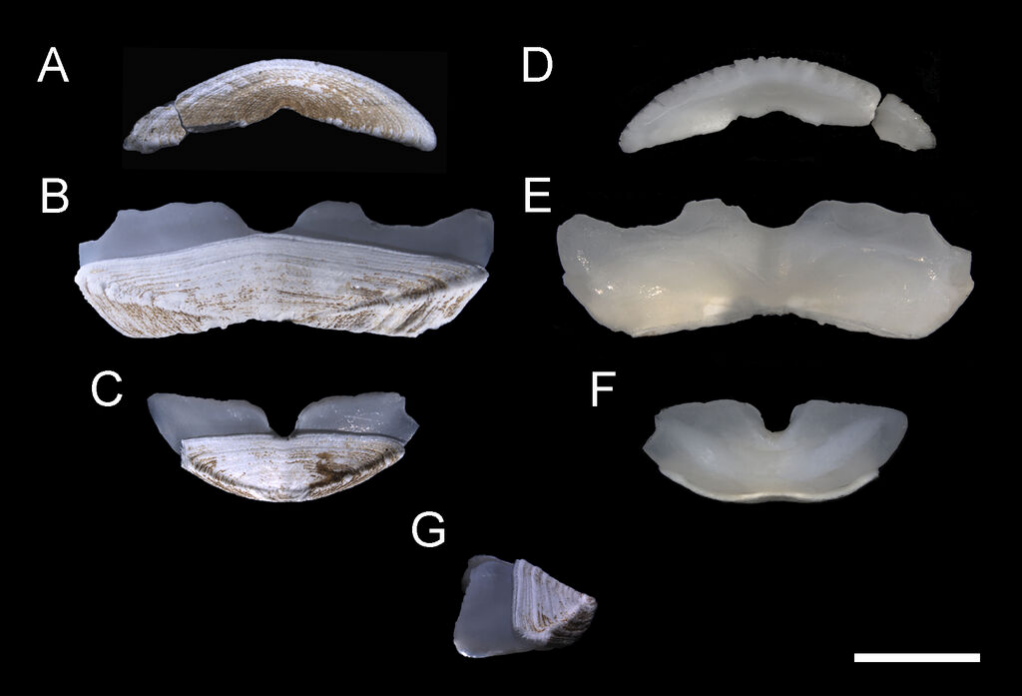
*Placiphorellamethanophila* Vončina, **sp. nov. A, D** Paratype. ZSM Mol 20080824; **B, C, E–G** Holotype, ZSM Mol 20041044. **A–C.** Valves I, VII, VIII, respectively, dorsal view; **D–F** Valves I, VII, VIII, respectively, ventral view; **G** Valve VIII, lateral view. Scale bar: 2 mm.

**Body** of medium size (18–30 mm x 12–23 mm, holotype 30 mm x 23 mm), broadly oval, low-elevated, subcarinated, side slopes straight to slightly convex; valves not beaked or with small, not pronounced apex (Fig. 1). Tegmentum minutely and irregularly granulated, white or yellowish, usually mottled with brown along the posterior valve margins; girdle broadly expanded anteriorly, usually white mottled with light-brown and brown (Figs 1, 2). See Schwabe and Sellanes (2010), Figs. 14 I–J and 15, for additional holotype photograph and detailed SEM photos of the girdle spicules and radula.

**Head** valve crescent-shaped, front slope straight, posterior margin very widely V-shaped, with a small median notch and little raised apex, tegmentum minutely and irregularly granulated, with some inconspicuous, irregular radial ridges and concentric growth lines (Figs 1, 2A and D). Intermediate valves broadly rectangular, very wide, with valve III being the widest, short, front margin widely angular, weakly projected forward at jugal part; side margins rounded, hind margin concave, apex weakly or not indicated, lateral areas raised, bordered by raised diagonal ribs, interspace shallowly excavated, crossed by conspicuous concentric growth lines (Figs 1, 2B and E). Tail valve small, roughly triangular in outline, about half as wide as widest intermediate valve, front margin straight to slightly convex, hind margin with a shallow or obsolete caudal sinus, mucro distinct, raised, terminal, overhanging, antemucronal area almost straight, postmucronal area slightly convex, tegmentum with two ribs separating the ante- and postmucronal areas (Fig. 2C and F–G).

**Articulamentum** strongly developed, white, valves calloused, apophyses very wide, short, slightly rounded to subtrapezoidal, trapezoid in the tail valve, separated by a narrow jugal sinus, insertion plates short, slit formula 15–16/1/sinus, slits shallow, no slit rays, teeth thick, bilobed and crenulated (Fig. 2).

**Figure 3.**
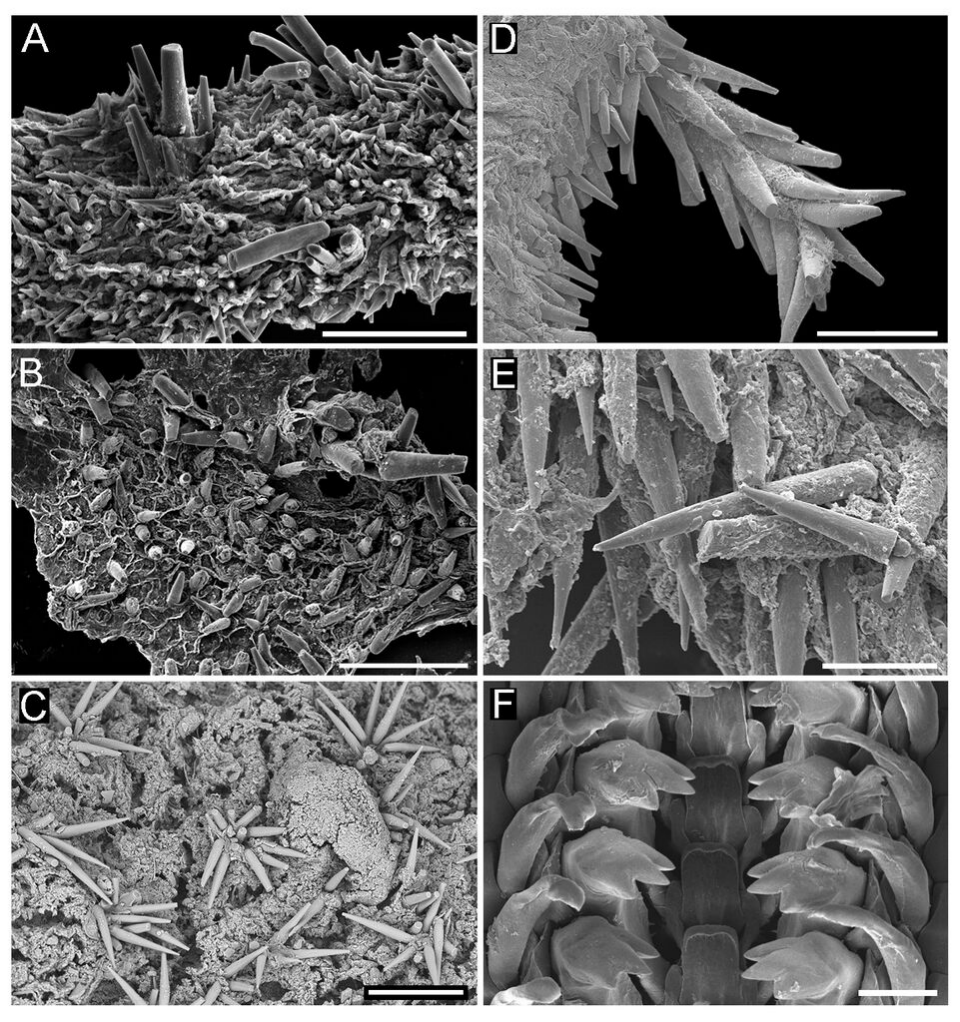
*Placiphorellamethanophila* Vončina, **sp. nov. A–B.** Specimen from ZIN collection (ZIN 2587), photos by courtesy of Boris Sirenko. **C–F.** Holotype, ZSM Mol 20041044. **A–B.** Dorsal spicules of perinotum; **C.** Longer dorsal spicules of perinotum clustered in groups; **D.** Bristle with spicules and marginal spicules; **E.** Spicules of precephalic tentacles; **F.** Central portion of radula. Scale bars: 200 µm (A –D) , 50 µm (E) , 20 µm (F).

**Girdle** broadly expanded anteriorly, uniformly brown or yellowish, dorsally covered with two kinds of spicules: single, smooth, and sharply pointed spicules, L: 75–100 μm (mean = 85 μm, n = 5), W: 15–25 μm (mean = 21, n = 5) and similar, but longer spicules gathered in groups of a few, L: 107–250 μm (mean = 120 μm, n = 12), W: 12–17 μm (mean = 15 μm, n = 5) (Fig. 3A–C). Sparsely scattered (more numerous near the front) large bristles, beset with slender, smooth and sharply-pointed spicules, L: 268 μm (n = 1), W: 29–36 μm (mean = 32 μm, n = 4), arranged in oblique series along axis (Fig. 3D). Marginal fringe composed of straight, smooth, sharp-topped spicules, L: 120–135 μm (mean = 125 μm, n = 3), W: 15–20 μm (mean = 17 μm, n = 5) (Fig. 3D). Ventral girdle spicules smooth, flattened, L: 80 μm (n = 1), W: 18–20 μm (mean = 19 μm, n = 2). Pallial fold with 6–14 tentacles, up to 2 mm in length with the middle ones the longest, spicules of the precephalic tentacles smooth, sharply-pointed L: 120–150 μm (mean = 130 μm, n = 5), W: 15–20 μm (mean = 17 μm, n = 5) (Fig. 3E).

**Radula** of holotype small, 5.8 mm in length, with 52 rows of teeth, of which 42 are of mature. Central tooth subrectangular, with wide base and curved blade, first lateral tooth elongate, wing-shaped with a narrow blade, major lateral tooth with tricuspid head, denticles pointed, central denticle somewhat longer than others, outer denticle widest and shallowest notched, first uncinal very prominent with high elevated lamellae, major uncinal elevated, slender with only slightly increased tip (Fig. 3F).

**Gills** merobranchial, 13–18 ctenidia per side (15 and 18 on the left and right side, respectively, in the holotype) in specimens 18–30 mm long.

#### Diagnosis

Chitons of medium size, up to 30 mm, body broadly oval, girdle expanded anteriorly; colour of the tegmentum white or yellow, mottled with brown; girdle white with light-brown and brown maculation. Valves depressed, subcarinated, minutely and irregularly granulated. Tail valve roughly triangular in shape, mucro terminal, overhanging. Girdle covered with two kinds of spicules: single, smooth and sharply pointed spicules and similar, but longer spicules gathered in groups of a few; sparsely scattered large bristles beset with elongated slender spicules.

Molecular diagnosis: COI: 132 – **C** , 168 – **G**, 258 – **A**, 267 – **C**, 300 – **T**, 348 – **C**, 411 – **A**, 420 – **A**, 438 – **G**.

#### Etymology

The specific epithet *methanophila* is a feminine adjective formed from the Latin noun methanum = methane, and the suffix -phila = “loving”, “friendly” or “friend”, underlining the close association of the new species with methane seeps.

#### Distribution

At present, only known from off Concepción, Chile; all specimens found in close relationship to the methane seeps (see discussion of Chilean records of *P.pacifica* in Schwabe and Sellanes (2010): 48).

#### Taxon discussion

Phenotypic characters discussion:

There are five species that share a head valve incision number > 10 with *Placiphorellamethanophila* **sp. nov.**, namely *Placiphorellaatlantica* (Verrill & S.I. Smith, 1882), *P.laurae* Clark, 2019, *P.isaotakii* Saito, Fujikura & Tsuchida, 2008, *P.okutanii* Saito, Fujikura & Tsuchida, 2008 and *P.pacifica* S. S. Berry, 1919. However, they differ from the new species by several characters, named below:

1. *Placiphorellamethanophila* **sp. nov.** differs from *P.atlantica* (source: Kaas and Van Belle (1994); Clark (1994)) by the shape of the tail valve (see also Schwabe and Sellanes (2010), Figs. 14 J–L for comparison; triangular with the straight antemucronal area in *P.methanophila* **sp. nov.** vs. trapezoidal with the concave antemucronal area in *P.atlantica*); sculpture of dorsal spicules (smooth, single and sharply pointed in *P.methanophila* **sp. nov.** vs. longitudinally striated and mamillated in *P.atlantica*); by the sculpture and size of marginal spicules (smooth and 120–135 μm long in *P.methanophila* **sp. nov.** vs. striated and 80 μm long in *P.atlantica*);
2. *Placiphorellamethanophila* **sp. nov.** differs from *P.isaotakii* (source: Saito et al. (2008)) by the sculpture of the tegmentum (minutely and irregularly granulated in *P.methanophila* **sp. nov.** vs. densely packed, elongate granules, occasionally merging into longitudinal threads in *P.isaotakii*); by the girdle ornamentation (smooth, single and sharply pointed, 75–100 μm long and similar, but gathered in groups and longer, 107–250 μm in *P.methanophila* **sp. nov.** vs. spicules mamillated at tip 50 μm × 40 µm long and longer, grouped spicules, 440 µm long in *P.isaotakii*); number of gills (13–18 gills in *P.methanophila* **sp. nov.** vs. 20–21 gills in *P.isaotakii*);
3. *Placiphorellamethanophila* **sp. nov.** differs from *P.laurae* (source: Clark (2019)) by the shape of the tail valve (straight antemucronal area, obtuse triangle-shaped in *P.methanophila* **sp. nov.** vs. concave antemucronal area, acute-angle triangle in *P.laurae*); length of dorsal spicules (107–250 μm long in *P.methanophila* **sp. nov.** vs. 400 μm long in *P.laurae*); size of spicules of bristles: 268 x 29–36 μm in *P.methanophila* **sp. nov.** vs. 350 x 40 μm in *P.laurae*;
4. *Placiphorellamethanophila* **sp. nov.** differs from *P.okutanii* (source: Saito et al. (2008)) by the shape of the tail valve (widely triangular in outline in *P.methanophila* **sp. nov.** vs. inversed trapezoidal, less wide in *P.okutanii*); by the dorsal girdle ornamentation (single sharply pointed spicules, 80–100 x 24–25 μm and similar, but clustered together spicules 107–250 μm x 12–17 μm in *P.methanophila* **sp. nov.** vs. spicules mamillated at tip 150 μm × 30 µm and longer, grouped spicules, 400 x 50 µm in *P.okutanii*);
5. *Placiphorellamethanophila* **sp. nov.** differs from *P.pacifica* (source: Berry (1919)) by the sculpture of the tegmentum (minutely and irregularly granulated in *P.methanophila* **sp. nov.** vs. smooth in *P.pacifica*); by the sculpture of the head valve (inconspicuous, irregular radial ridges in *P.methanophila* **sp. nov.** vs. 12-14 low, radiating ribs in *P.pacifica*); position of the mucro (terminal in *P.methanophila* **sp. nov.** vs. subterminal in *P.pacifica*).

Genetic discussion:

The ranges of uncorrected genetic p-distances between *Placiphorellamethanophila* **sp. nov.** and all *Placiphorella* available from GenBank in mitochondrial cytochrome oxidase subunit 1 (COI) gene sequences ranged from 4.1% to 14.6% (Table 1). The closest COI sequences are: KJ574090 (4.1% distance), representing *Placiphorella* sp. A from Irisarri et al. (2014), which was mentioned by authors as a potentially new species, but the description has never been published; and GU806074, GU806077-8, GU806080, GU806115 and GU806118 (4.6–5.0% distance) representing *P.atlantica*, from which *P.methanophila* **sp. nov.** can be also differentiated morphologically.

#### Notes

##### Methods

Live animals were collected at depths of 870 – 930 m during two cruises along the Chilean coast. Collecting was done using a 1.5 m wide Agassiz trawl (AGT), during hauls of 20 minutes. Specimens were fixed in 4% buffered formalin and preserved in 75% ethanol (ZSM Mol 20041044 and SMF 376539) or directly preserved in 95% ethanol (ZSM Mol 20080824). The systematic classification follows Sirenko (2006) with slight modifications. The morphological terminology follows Schwabe (2010).

For scanning electron microscopy (SEM), the valves and radula were removed, cleaned with a 5% sodium hydroxide (NaOH) solution and rinsed in distilled water. The pieces of the perinotum were only air-dried. Objects were placed on SEM stubs using double-sided adhesive tabs. After coating with gold for 135 seconds in a Polaron sputter coater, they were examined with a LEO 1430VP SEM. All figures were assembled in Adobe Photoshop CS6.

For DNA barcoding, a small fragment of tissue from two chitons ZSM Mol 20080824 was sampled. DNA was extracted using QIAamp DNA Micro Kit (QIAGEN), following the manufacturer’s protocol. The cytochrome oxidase subunit I (COI primers LCO1490 and HCO2198; Folmer et al. (1994)) was amplified using repliQa HiFi ToughMix from ThermoFisher, following the PCR programme for COI in Bonfitto et al. (2011). Out of two samples, only one was amplified and sent for sequencing. The obtained sequence was manually inspected in Geneious Prime v.2023.1 and was made publicly available on GenBank under the accession number PP133101. Additionally, fourteen COI sequences of *Placiphorella* were downloaded from GenBank and aligned with the new sequence from this study, using default settings of MAFFT7 (Katoh et al. 2002, Katoh and Toh 2008) under the Q-INS-I strategy. Uncorrected pairwise distances were calculated using MEGA11 (Tamura et al. 2021). There was a total of 460 positions in the final dataset. The number of base differences per site from between sequences are shown in Table 1. The molecular diagnosis of *P.methanophila* **sp. nov.** was composed in comparison with genetically closest *Placiphorella* (4.1–5.0% in uncorrected genetic p-distances in COI) with DeSignate web application (Hütter et al. 2020). The diagnostic molecular character (signature characters) for *P.methanophila* **sp. nov.** was defined as the position of a nucleotide in an alignment, which is monomorphic within each species and differs between species. Only binary positions, k-window = 1 and no deletions were used (Hütter et al. 2020).

Abbreviations used in the text are as follows: BL – body length; L – length; W – width; ZSM Mol – Bavarian State Collection of Zoology; SMF – Senckenberg Research Institute and Natural History Museum Frankfurt, Frankfurt, Germany, ZIN – Zoological Institute of Russian Academy of Sciences, St. Petersburg, Russia.

Holotype (ZSM Mol 20041044), now disarticulated: parts of girdle, precephalic lappet, radula, on three SEM stubs ZSM Mol 20220314 and two paratypes ZSM Mol 20080824 are deposited in the collection of Zoologische Staatssammlung München, Munich, Germany; a paratype SMF 376539 is deposited in the malacological collection of Senckenberg Research Institute and Natural History Museum Frankfurt, Frankfurt, Germany.

### Lepetodrilus marianae

Chen, Watanabe & Tsuda sp. nov.

6E6256D4-56AF-579F-8CA1-1728BBB60F23

76468C58-9FF2-48FE-BF51-4A7B9219A0D3

Lepetodrilusaff.schrolli MT: Johnson et al. (2008): fig. 1, table 2; Giguère and Tunnicliffe (2021): table 2
Lepetodrilusaff.schrolli Mariana Trough: Poitrimol et al. (2022): fig. 2A

#### Materials

- **Type status:** Holotype. **Occurrence:** catalogNumber: SMF 373150; recordedBy: R/V KAIMEI cruise KM23-05; individualCount: 1; sex: female; lifeStage: adult; preparations: 99% EtOH; occurrenceID: 54FC6400-9086-515C-9A40-FF588BC6160E; **Taxon:** scientificName: *Lepetodrilusmarianae* Chen, Watanabe & Tsuda; kingdom: Animalia; phylum: Mollusca; class: Gastropoda; order: Lepetellida; family: Lepetodrilidae; genus: Lepetodrilus; specificEpithet: *marianae*; taxonRank: species; scientificNameAuthorship: Chen, Watanabe & Tsuda; nomenclaturalCode: ICZN; **Location:** higherGeography: Pacific Ocean; waterBody: Western Pacific Ocean; islandGroup: Northern Mariana Islands; country: United States of America; locality: Northwest Eifuku Volcano vent field, near Champagne vent; verbatimDepth: 1660 m; verbatimLatitude: 21°29.2506'N; verbatimLongitude: 144°02.4498'E; **Event:** samplingProtocol: suction sampler mounted on ROV KM-ROV, dive #223; eventDate: 25/03/2023; habitat: white bacterial mats on rocks around diffuse flow venting; **Record Level:** institutionCode: SMF; basisOfRecord: PreservedSpecimen
- **Type status:** Paratype. **Occurrence:** catalogNumber: NSMT-Mo 79482; recordedBy: R/V KAIMEI cruise KM23-05; individualCount: 1; sex: male; lifeStage: adult; preparations: 99% EtOH; occurrenceID: 29F12B98-B99E-514F-A9F1-A17B77E9B9C9; **Taxon:** scientificName: *Lepetodrilusmarianae* Chen, Watanabe & Tsuda; kingdom: Animalia; phylum: Mollusca; class: Gastropoda; order: Lepetellida; family: Lepetodrilidae; genus: Lepetodrilus; specificEpithet: *marianae*; taxonRank: species; scientificNameAuthorship: Chen, Watanabe & Tsuda; nomenclaturalCode: ICZN; **Location:** higherGeography: Pacific Ocean; waterBody: Western Pacific Ocean; islandGroup: Northern Mariana Islands; country: United States of America; locality: Northwest Eifuku Volcano vent field, near Champagne vent; verbatimDepth: 1660 m; verbatimLatitude: 21°29.2506'N; verbatimLongitude: 144°02.4498'E; **Event:** samplingProtocol: suction sampler mounted on ROV KM-ROV, dive #223; eventDate: 25/03/2023; habitat: white bacterial mats on rocks around diffuse flow venting; **Record Level:** institutionCode: NSMT; basisOfRecord: PreservedSpecimen
- **Type status:** Paratype. **Occurrence:** catalogNumber: NSMT-Mo 79483; recordedBy: R/V KAIMEI cruise KM23-05; individualCount: 1; sex: female; lifeStage: adult; preparations: fixed and preserved in 10% buffered formalin; occurrenceID: 9D0216B3-C5D4-5E6A-88AB-C7F8B24BD463; **Taxon:** scientificName: *Lepetodrilusmarianae* Chen, Watanabe & Tsuda; kingdom: Animalia; phylum: Mollusca; class: Gastropoda; order: Lepetellida; family: Lepetodrilidae; genus: Lepetodrilus; specificEpithet: *marianae*; taxonRank: species; scientificNameAuthorship: Chen, Watanabe & Tsuda; nomenclaturalCode: ICZN; **Location:** higherGeography: Pacific Ocean; waterBody: Western Pacific Ocean; islandGroup: Northern Mariana Islands; country: United States of America; locality: Northwest Eifuku Volcano vent field, near Champagne vent; verbatimDepth: 1660 m; verbatimLatitude: 21°29.2506'N; verbatimLongitude: 144°02.4498'E; **Event:** samplingProtocol: suction sampler mounted on ROV KM-ROV, dive #223; eventDate: 25/03/2023; habitat: white bacterial mats on rocks around diffuse flow venting; **Record Level:** institutionCode: NSMT; basisOfRecord: PreservedSpecimen
- **Type status:** Paratype. **Occurrence:** catalogNumber: MNHN-IM-2019-34806; recordedBy: R/V KAIMEI cruise KM23-05; individualCount: 1; lifeStage: adult; preparations: 99% EtOH; occurrenceID: E4CBEB40-C841-5405-BC70-6E59179B58E8; **Taxon:** scientificName: *Lepetodrilusmarianae* Chen, Watanabe & Tsuda; kingdom: Animalia; phylum: Mollusca; class: Gastropoda; order: Lepetellida; family: Lepetodrilidae; genus: Lepetodrilus; specificEpithet: *marianae*; taxonRank: species; scientificNameAuthorship: Chen, Watanabe & Tsuda; nomenclaturalCode: ICZN; **Location:** higherGeography: Pacific Ocean; waterBody: Western Pacific Ocean; islandGroup: Northern Mariana Islands; country: United States of America; locality: Northwest Eifuku Volcano vent field, near Champagne vent; verbatimDepth: 1660 m; verbatimLatitude: 21°29.2506'N; verbatimLongitude: 144°02.4498'E; **Event:** samplingProtocol: suction sampler mounted on ROV KM-ROV, dive #223; eventDate: 25/03/2023; habitat: white bacterial mats on rocks around diffuse flow venting; **Record Level:** institutionCode: MNHN; basisOfRecord: PreservedSpecimen
- **Type status:** Paratype. **Occurrence:** catalogNumber: MNHN-IM-2023-431; recordedBy: R/V KAIMEI cruise KM23-05; individualCount: 1; lifeStage: adult; preparations: fixed and preserved in 10% buffered formalin; occurrenceID: 34525B69-92DA-5441-B18A-7E22198C73D4; **Taxon:** scientificName: *Lepetodrilusmarianae* Chen, Watanabe & Tsuda; kingdom: Animalia; phylum: Mollusca; class: Gastropoda; order: Lepetellida; family: Lepetodrilidae; genus: Lepetodrilus; specificEpithet: *marianae*; taxonRank: species; scientificNameAuthorship: Chen, Watanabe & Tsuda; nomenclaturalCode: ICZN; **Location:** higherGeography: Pacific Ocean; waterBody: Western Pacific Ocean; islandGroup: Northern Mariana Islands; country: United States of America; locality: Northwest Eifuku Volcano vent field, near Champagne vent; verbatimDepth: 1660 m; verbatimLatitude: 21°29.2506'N; verbatimLongitude: 144°02.4498'E; **Event:** samplingProtocol: suction sampler mounted on ROV KM-ROV, dive #223; eventDate: 25/03/2023; habitat: white bacterial mats on rocks around diffuse flow venting; **Record Level:** institutionCode: MNHN; basisOfRecord: PreservedSpecimen
- **Type status:** Paratype. **Occurrence:** catalogNumber: SMF 373151; recordedBy: R/V KAIMEI cruise KM23-05; individualCount: 1; lifeStage: adult; preparations: fixed and preserved in 10% buffered formalin; occurrenceID: E0369298-32C6-556A-825E-4A4F51164311; **Taxon:** scientificName: *Lepetodrilusmarianae* Chen, Watanabe & Tsuda; kingdom: Animalia; phylum: Mollusca; class: Gastropoda; order: Lepetellida; family: Lepetodrilidae; genus: Lepetodrilus; specificEpithet: *marianae*; taxonRank: species; scientificNameAuthorship: Chen, Watanabe & Tsuda; nomenclaturalCode: ICZN; **Location:** higherGeography: Pacific Ocean; waterBody: Western Pacific Ocean; islandGroup: Northern Mariana Islands; country: United States of America; locality: Northwest Eifuku Volcano vent field, near Champagne vent; verbatimDepth: 1660 m; verbatimLatitude: 21°29.2506'N; verbatimLongitude: 144°02.4498'E; **Event:** samplingProtocol: suction sampler mounted on ROV KM-ROV, dive #223; eventDate: 25/03/2023; habitat: white bacterial mats on rocks around diffuse flow venting; **Record Level:** institutionCode: SMF; basisOfRecord: PreservedSpecimen
- **Type status:** Paratype. **Occurrence:** catalogNumber: SMF 373152; recordedBy: R/V KAIMEI cruise KM23-05; individualCount: 5; lifeStage: growth series; preparations: 99% EtOH; occurrenceID: C0511CEB-4708-55D3-B60C-ECF23380473A; **Taxon:** scientificName: *Lepetodrilusmarianae* Chen, Watanabe & Tsuda; kingdom: Animalia; phylum: Mollusca; class: Gastropoda; order: Lepetellida; family: Lepetodrilidae; genus: Lepetodrilus; specificEpithet: *marianae*; taxonRank: species; scientificNameAuthorship: Chen, Watanabe & Tsuda; nomenclaturalCode: ICZN; **Location:** higherGeography: Pacific Ocean; waterBody: Western Pacific Ocean; islandGroup: Northern Mariana Islands; country: United States of America; locality: Northwest Eifuku Volcano vent field, near Champagne vent; verbatimDepth: 1660 m; verbatimLatitude: 21°29.2506'N; verbatimLongitude: 144°02.4498'E; **Event:** samplingProtocol: suction sampler mounted on ROV KM-ROV, dive #223; eventDate: 25/03/2023; habitat: white bacterial mats on rocks around diffuse flow venting; **Record Level:** institutionCode: SMF; basisOfRecord: PreservedSpecimen
- **Type status:** Paratype. **Occurrence:** catalogNumber: NSMT-Mo 79484; recordedBy: R/V KAIMEI cruise KM23-05; individualCount: 5; lifeStage: growth series; preparations: 99% EtOH; occurrenceID: 18D8E94F-2DC7-5B47-9FDC-4BE5D33EF30A; **Taxon:** scientificName: *Lepetodrilusmarianae* Chen, Watanabe & Tsuda; kingdom: Animalia; phylum: Mollusca; class: Gastropoda; order: Lepetellida; family: Lepetodrilidae; genus: Lepetodrilus; specificEpithet: *marianae*; taxonRank: species; scientificNameAuthorship: Chen, Watanabe & Tsuda; nomenclaturalCode: ICZN; **Location:** higherGeography: Pacific Ocean; waterBody: Western Pacific Ocean; islandGroup: Northern Mariana Islands; country: United States of America; locality: Northwest Eifuku Volcano vent field, near Champagne vent; verbatimDepth: 1660 m; verbatimLatitude: 21°29.2506'N; verbatimLongitude: 144°02.4498'E; **Event:** samplingProtocol: suction sampler mounted on ROV KM-ROV, dive #223; eventDate: 25/03/2023; habitat: white bacterial mats on rocks around diffuse flow venting; **Record Level:** institutionCode: NSMT; basisOfRecord: PreservedSpecimen
- **Type status:** Paratype. **Occurrence:** catalogNumber: MNHN-IM-2019-34807; recordedBy: R/V KAIMEI cruise KM23-05; individualCount: 5; lifeStage: growth series; preparations: 99% EtOH; occurrenceID: 7DC6CE20-ACE6-5F45-BC6C-083947555E92; **Taxon:** scientificName: *Lepetodrilusmarianae* Chen, Watanabe & Tsuda; kingdom: Animalia; phylum: Mollusca; class: Gastropoda; order: Lepetellida; family: Lepetodrilidae; genus: Lepetodrilus; specificEpithet: *marianae*; taxonRank: species; scientificNameAuthorship: Chen, Watanabe & Tsuda; nomenclaturalCode: ICZN; **Location:** higherGeography: Pacific Ocean; waterBody: Western Pacific Ocean; islandGroup: Northern Mariana Islands; country: United States of America; locality: Northwest Eifuku Volcano vent field, near Champagne vent; verbatimDepth: 1660 m; verbatimLatitude: 21°29.2506'N; verbatimLongitude: 144°02.4498'E; **Event:** samplingProtocol: suction sampler mounted on ROV KM-ROV, dive #223; eventDate: 25/03/2023; habitat: white bacterial mats on rocks around diffuse flow venting; **Record Level:** institutionCode: MNHN; basisOfRecord: PreservedSpecimen
- **Type status:** Other material. **Occurrence:** catalogNumber: NSMT-Mo 79485; recordedBy: R/V KAIMEI cruise KM23-05; individualCount: about 50; preparations: 99% EtOH, -80°C; associatedSequences: https://www.ncbi.nlm.nih.gov/nuccore/OR640969 | https://www.ncbi.nlm.nih.gov/nuccore/OR640970; occurrenceID: FF29D3D8-D51C-580F-BA53-BF26335C9C19; **Taxon:** scientificName: *Lepetodrilusmarianae* Chen, Watanabe & Tsuda; kingdom: Animalia; phylum: Mollusca; class: Gastropoda; order: Lepetellida; family: Lepetodrilidae; genus: Lepetodrilus; specificEpithet: *marianae*; taxonRank: species; scientificNameAuthorship: Chen, Watanabe & Tsuda; nomenclaturalCode: ICZN; **Location:** higherGeography: Pacific Ocean; waterBody: Western Pacific Ocean; islandGroup: Northern Mariana Islands; country: United States of America; locality: Northwest Eifuku Volcano vent field, near Champagne vent; verbatimDepth: 1660 m; verbatimLatitude: 21°29.2506'N; verbatimLongitude: 144°02.4498'E; **Event:** samplingProtocol: suction sampler mounted on ROV KM-ROV, dive #223; eventDate: 25/03/2023; habitat: white bacterial mats on rocks around diffuse flow venting; **Record Level:** institutionCode: NSMT; basisOfRecord: PreservedSpecimen

#### Description

**Figure 4.**
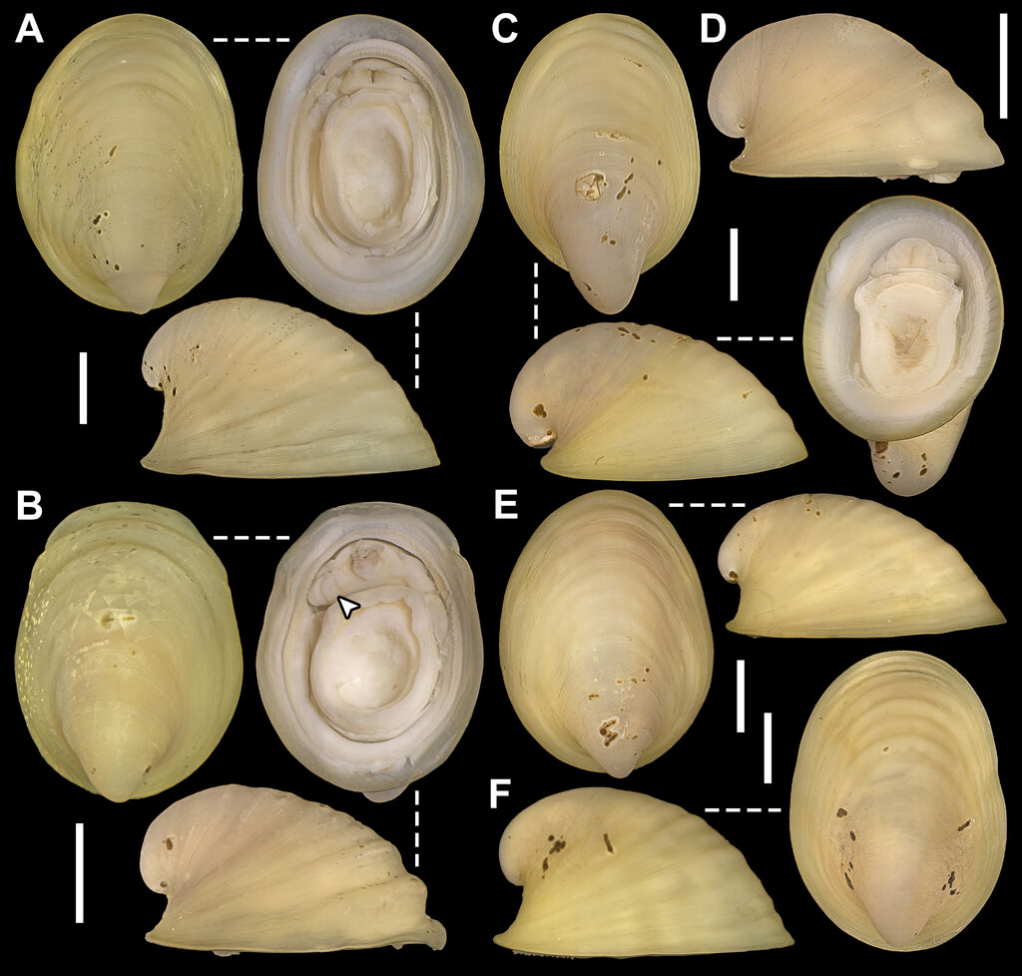
*Lepetodrilusmarianae* Chen, Watanabe & Tsuda, **sp. nov.**, habitus photographs of representative type specimens. **A** Holotype (SMF 373150), dorsal, ventral and lateral views; **B** Paratype 1 (NSMT-Mo 79482), dorsal, ventral and lateral views, arrowhead on ventral view indicates the penis; **C** Paratype 2 (NSMT-Mo 79483), dorsal, ventral and lateral views; **D** Paratype 3 (MNHN-IM-2019-34806), lateral view; **E** Paratype 4 (MNHN-IM-2023-431), dorsal and lateral views; **F** Paratype 5 (SMF 373151), dorsal and lateral views. Scale bars: 2 mm.

**Figure 5.**
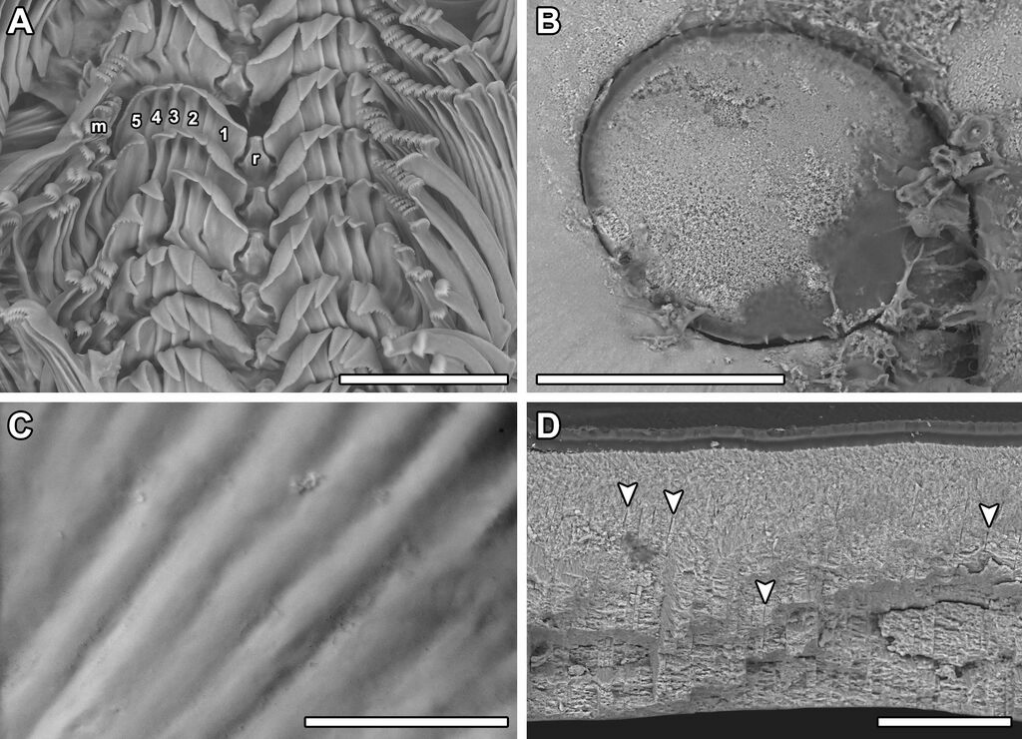
*Lepetodrilusmarianae* Chen, Watanabe & Tsuda, **sp. nov.**, scanning electron micrographs, NSMT-Mo 79485. **A** Radula (r, central or rachidian tooth; 1–5 denoting lateral tooth from inside to outside; m, marginal teeth); **B** Protoconch; **C** Fine concentric sculpture on shell surface; **D** Fractured cross section of a shell showing microstructure (arrowheads indicate shell pores). Scale bars: 100 μm (A, B, D), 50 μm (C).

Shell (Fig. 4): very loosely coiled, juvenile shell becomes limpet-shaped in adult stages. Medium-sized for genus, aperture oval, maximum shell length around 8.5 mm. Shell dimensions of the holotype and paratypes 1–5 are given in Table 2. Apex at posterior end of shell (Fig. 4A) or protruding beyond it (Fig. 4C) depending on substrate morphology, also influencing shell height. Fine concentric ribs (Fig. 5C) present across all growth stages, adults developing broad, strongly raised concentric ridges to give strongly undulated profiles (Fig. 4A-F, see lateral views); strength and frequency of latter ridges varying amongst individuals. Shell microstructure (Fig. 5D) with thin outer homogenous layer and thick inner complex crossed lamellar layer, numerous fine shell pores are presented perpendicular to shell surface. Protoconch (Fig. 5B) indistinctly coiled, approximately 170 μm in diameter. Protoconch sculpture corroded and indiscernible on all specimens investigated. Periostracum light green, smooth, moderately thick, enveloping edge of shell.

External anatomy (Fig. 4A–C) overall typical for *Lepetodrilus*. Oval foot, muscle scar horseshoe-shaped, left arm longer than right arm. Cephalic tentacles simple, conical, extending from broad base. Eyes lacking. Penis in males simple conical with narrow base, tapering to blunt tip, seminal groove on dorsal side. Two pairs of epipodial tentacles present on posterior end of foot, simple conical with broad base. Mantle edge with two folds, outer fold thin, extending to edge of periostracum; inner fold thick, densely lining with (presumably sensory) small tentacles. Operculum lacking.

Radula (Fig. 5A) rhipidoglossate, formula ca. 25-5-1-5- ca. 25. Central (rachidian) tooth low with broad shaft carrying one lateral ridge on either side, rapidly tapering to single narrow, pointed cusp with smooth cutting edges. Innermost lateral (‘1’, Fig. 5A) twice as broad as central tooth, with slanted, very broad overhanging cusp only carrying very weak serrations on outermost one-third. Three middle laterals (‘2–4’, Fig. 5A) narrower, of similar breadth to central tooth, carrying simple triangular overhanging cusps, inner edges carrying weak serrations, outer edges smooth. Outermost lateral (‘5’, Fig. 5A) much broader again, with triangular overhanging cusp carrying weak serrations on inner edge plus strong serrations on outer edge. Marginals with very elongated shafts ending in spoon-like cusps finely serrated into about 20 denticles, innermost denticle strongest by far. Size of marginals decrease gradually outwards.

#### Diagnosis

A medium-sized *Lepetodrilus* with two types of concentric sculpture: regular, fine concentric ribs across all growth stages and irregular, strongly raised concentric ridges in adults. Apex at the posterior end of shell or overhanging it.

#### Etymology

The specific epithet *marianae* is a noun in the genitive case, after the species distribution range in Mariana Arc and Mariana Trough. This species was amongst many western Pacific vent gastropods first recognised as new by the late German malacologist Lothar A. Beck (Chen and Sigwart 2023). During the course of searching for unpublished material and manuscript by Beck (see Chen and Sigwart (2023)), we came across unpublished writing by Beck and his former student Kathrin Sobjinski suggesting the specific epithet *marianae* for this species, which we have decided to honour.

#### Distribution

Specimens of *L.marianae* Chen, Watanabe & Tsuda, **sp. nov.** have been collected at several hot vent fields in both Mariana Arc (northwest Eifuku, northwest Rota, Seamount X) and Mariana Trough (Alice Springs, Illium, Burke, Hafa Adai, Perseverence, Forecast, Snail and Archaean), confirmed by COI sequences (Johnson et al. 2008, Giguère and Tunnicliffe 2021).

#### Taxon discussion

*Lepetodrilus* is a genus of vetigastropod limpets commonly found in hydrothermal vents globally. The first comprehensive molecular phylogenetic reconstruction of this genus using the COI gene by Johnson et al. (2008) revealed that *Lepetodrilus* inhabiting the south-western Pacific vents consisted of three cryptic species showing clear barcoding gaps, including *L.schrolli* L. Beck, 1993 from Manus Basin, ‘Lepetodrilusaff.schrolli LF’ from Lau and Fiji Basins and ‘Lepetodrilusaff.schrolli MT’ from Mariana Trough. Later, further sampling and sequencing of material from Mariana Arc by Giguère and Tunnicliffe (2021) revealed highly similar COI sequences to ‘Lepetodrilusaff.schrolli MT’ (*sensu* Johnson et al. (2008)), extending its distribution to Mariana Arc vents including the Northwest Eifuku Volcano vent field (the type locality as per this work). Although Giguère and Tunnicliffe (2021) did not carry out a phylogenetic analysis using their sequences of this species (COI, MW807763-MW807765; 18S rRNA, MZ128922-MZ128923), this was done by Poitrimol et al. (2022) using the COI gene which confirmed that the Mariana Trough sequences from Johnson et al. (2008) (EU306431-EU306436) and the Mariana Arc sequences from Giguère and Tunnicliffe (2021) (MW807763-MW807765) fall into the same species-level clade. In both abovementioned phylogenetic analyses, ‘Lepetodrilusaff.schrolli MT’ (*sensu* Johnson et al. (2008)) was recovered sister to a clade containing *L.schrolli* and ‘Lepetodrilusaff.schrolli LF’ (*sensu* Johnson et al. (2008)). The lowest uncorrected sequence divergence for the COI gene amongst these three species was 3.1% (Johnson et al. 2008).

Lothar A. Beck attempted to describe these two *Lepetodrilus* species from the southwest Pacific, but they were never published before he passed away in 2020 (Chen and Sigwart 2023). Recently, many new species Beck recognised were formally published by Chen and Sigwart (2023), based on Beck’s unpublished manuscript, including ‘Lepetodrilusaff.schrolli LF’ *sensu* Johnson et al. (2008) which is now *Lepetodrilusfijiensis* Beck in Chen & Sigwart, 2023. ‘Lepetodrilusaff.schrolli MT’ (*sensu* Johnson et al. (2008)), however, remained undescribed until now and corresponds to *Lepetodrilusmarianae* Chen, Watanabe & Tsuda, **sp. nov.** described herein. Although we were not able to study the previously sequenced specimens, we could examine *Lepetodrilus* specimens from NW Eifuku (Scripps Institution of Oceanography, Benthic Invertebrate Collection SIO-BIC M18546) collected together with those sequenced in Giguère and Tunnicliffe (2021) and they agreed morphologically with *L.marianae* **sp. nov.** Two new COI sequences were generated for this work from two specimens in paratype series 7 (NSMT-Mo 79484) and deposited in GenBank under the accession numbers OR640969-OR640970. All abovementioned sequences of *L.marianae* **sp. nov.** exhibited uncorrected pairwise genetic similarities above 99.2%.

Morphologically, *L.marianae* **sp. nov.** can be easily distinguished from other described *Lepetodrilus* species by its strong concentric sculpture, including its closest relatives (Johnson et al. 2008) *L.schrolli* and *L.fijiensis* L. Beck in Chen & Sigwart, 2023 (Beck 1993, Chen and Sigwart 2023). The only other *Lepetodrilus* species with similar shell profile and concentric sculpture is *L.concentricus* Linse, Roterman & Chen, 2019 from Antarctic vents on the East Scotia Ridge, but *L.concentricus* only exhibits one type of sculpture intermediate in strength between the two types seen in *L.marianae* **sp. nov.** (Linse et al. 2019) and, therefore, they are morphologically distinct.

#### Notes

##### Methods

Gastropods were collected near Champagne vent (Tunnicliffe et al. 2009), Northwest Eifuku Volcano vent field (21°29.2506'N, 144°02.4498'E), 1660 m depth, using a suction sampler mounted on the remotely-operated vehicle (ROV) *KM-ROV* on-board R/V *KAIMEI* cruise KM23-05. The animals were sieved on a 1 mm sieve using cold seawater and sorted into different morphospecies. Specimens were placed into 10% buffered formalin or 99% ethanol as soon as they were sorted. Dissections were done under a stereomicroscope (Olympus SZX7). Investigation of the gross morphology and genetic barcoding of the mitochondrial cytochrome *c* oxidase subunit I (COI) gene were conducted using the *Lepetodrilus*-specific primer pairs LepetESR-F/LepetESR-R following Linse et al. (2019). Scanning electron microscopy was done using a Hitachi TM-3000 table-top system at 15 kV. Shell microstructure terminology follows Kiel (2004). Specimens are deposited in Senckenberg Museum Frankfurt (SMF), National Museum of Nature and Science, Tsukuba (NSMT) or Muséum national d’Histoire naturelle, Paris (MNHN).

### Shinkailepas gigas

Chen, Watanabe & Tsuda sp. nov.

C6489CD9-2101-5198-BA24-3C7100F00D76

A7359192-34E4-4707-87C8-2AD89B69E61C

*Shinkailepas* sp. nov. – Giguère and Tunnicliffe (2021): table S5
*Shinkailepas* sp. Manus Basin/Mariana Volcanic Arc – Poitrimol et al. (2022): fig. 6

#### Materials

- **Type status:** Holotype. **Occurrence:** catalogNumber: SMF 373153; recordedBy: R/V KAIMEI cruise KM23-05; individualCount: 1; sex: male; lifeStage: adult; preparations: fixed and preserved in 10% buffered formalin; occurrenceID: E8CBFB26-01DB-5085-8071-20E65C686665; **Taxon:** scientificName: *Shinkailepasgigas* Chen, Watanabe & Tsuda; kingdom: Animalia; phylum: Mollusca; class: Gastropoda; order: Lepetellida; family: Lepetodrilidae; genus: Shinkailepas; specificEpithet: *gigas*; taxonRank: species; scientificNameAuthorship: Chen, Watanabe & Tsuda; nomenclaturalCode: ICZN; **Location:** higherGeography: Pacific Ocean; waterBody: Western Pacific Ocean; islandGroup: Northern Mariana Islands; country: United States of America; locality: Northwest Eifuku Volcano vent field, near Champagne vent; verbatimDepth: 1615 m; verbatimLatitude: 21°29.2383'N; verbatimLongitude: 144°02.4937'E; **Event:** samplingProtocol: suction sampler mounted on ROV KM-ROV, dive #213; eventDate: 07/03/2023; habitat: white bacterial mats on rocks around diffuse flow venting; **Record Level:** institutionCode: SMF; basisOfRecord: PreservedSpecimen
- **Type status:** Paratype. **Occurrence:** catalogNumber: NSMT-Mo 79486; recordedBy: R/V KAIMEI cruise KM23-05; individualCount: 1; sex: male; lifeStage: adult; preparations: fixed and preserved in 10% buffered formalin; occurrenceID: 1A32DE7D-9C5D-5C5F-8058-0CB78A9C00D9; **Taxon:** scientificName: *Shinkailepasgigas* Chen, Watanabe & Tsuda; kingdom: Animalia; phylum: Mollusca; class: Gastropoda; order: Lepetellida; family: Lepetodrilidae; genus: Shinkailepas; specificEpithet: *gigas*; taxonRank: species; scientificNameAuthorship: Chen, Watanabe & Tsuda; nomenclaturalCode: ICZN; **Location:** higherGeography: Pacific Ocean; waterBody: Western Pacific Ocean; islandGroup: Northern Mariana Islands; country: United States of America; locality: Northwest Eifuku Volcano vent field, near Champagne vent; verbatimDepth: 1615 m; verbatimLatitude: 21°29.2383'N; verbatimLongitude: 144°02.4937'E; **Event:** samplingProtocol: suction sampler mounted on ROV KM-ROV, dive #213; eventDate: 07/03/2023; habitat: white bacterial mats on rocks around diffuse flow venting; **Record Level:** institutionCode: NSMT; basisOfRecord: PreservedSpecimen
- **Type status:** Paratype. **Occurrence:** catalogNumber: SMF 373154; recordedBy: R/V KAIMEI cruise KM23-05; individualCount: 1; sex: female; lifeStage: adult; preparations: 99% EtOH; occurrenceID: 495CC54B-399F-5D22-B3FB-D3A5C9DC141D; **Taxon:** scientificName: *Shinkailepasgigas* Chen, Watanabe & Tsuda; kingdom: Animalia; phylum: Mollusca; class: Gastropoda; order: Lepetellida; family: Lepetodrilidae; genus: Shinkailepas; specificEpithet: *gigas*; taxonRank: species; scientificNameAuthorship: Chen, Watanabe & Tsuda; nomenclaturalCode: ICZN; **Location:** higherGeography: Pacific Ocean; waterBody: Western Pacific Ocean; islandGroup: Northern Mariana Islands; country: United States of America; locality: Northwest Eifuku Volcano vent field, near Champagne vent; verbatimDepth: 1615 m; verbatimLatitude: 21°29.2383'N; verbatimLongitude: 144°02.4937'E; **Event:** samplingProtocol: suction sampler mounted on ROV KM-ROV, dive #213; eventDate: 07/03/2023; habitat: white bacterial mats on rocks around diffuse flow venting; **Record Level:** institutionCode: SMF; basisOfRecord: PreservedSpecimen
- **Type status:** Paratype. **Occurrence:** catalogNumber: MNHN-IM-2019-34808; recordedBy: R/V KAIMEI cruise KM23-05; individualCount: 1; sex: female; lifeStage: adult; preparations: 99% EtOH; occurrenceID: 9C442EE1-49C3-52F5-B984-DD998283F040; **Taxon:** scientificName: *Shinkailepasgigas* Chen, Watanabe & Tsuda; kingdom: Animalia; phylum: Mollusca; class: Gastropoda; order: Lepetellida; family: Lepetodrilidae; genus: Shinkailepas; specificEpithet: *gigas*; taxonRank: species; scientificNameAuthorship: Chen, Watanabe & Tsuda; nomenclaturalCode: ICZN; **Location:** higherGeography: Pacific Ocean; waterBody: Western Pacific Ocean; islandGroup: Northern Mariana Islands; country: United States of America; locality: Northwest Eifuku Volcano vent field, near Champagne vent; verbatimDepth: 1615 m; verbatimLatitude: 21°29.2383'N; verbatimLongitude: 144°02.4937'E; **Event:** samplingProtocol: suction sampler mounted on ROV KM-ROV, dive #213; eventDate: 07/03/2023; habitat: white bacterial mats on rocks around diffuse flow venting; **Record Level:** institutionCode: MNHN; basisOfRecord: PreservedSpecimen
- **Type status:** Paratype. **Occurrence:** catalogNumber: NSMT-Mo 79487; occurrenceRemarks: fragmented specimen; recordedBy: R/V KAIMEI cruise KM23-05; individualCount: 1; lifeStage: adult; preparations: 99% EtOH; associatedSequences: https://www.ncbi.nlm.nih.gov/nuccore/OR640971; occurrenceID: AD20F976-B32C-5B4A-AA92-A050891CEB37; **Taxon:** scientificName: *Shinkailepasgigas* Chen, Watanabe & Tsuda; kingdom: Animalia; phylum: Mollusca; class: Gastropoda; order: Lepetellida; family: Lepetodrilidae; genus: Shinkailepas; specificEpithet: *gigas*; taxonRank: species; scientificNameAuthorship: Chen, Watanabe & Tsuda; nomenclaturalCode: ICZN; **Location:** higherGeography: Pacific Ocean; waterBody: Western Pacific Ocean; islandGroup: Northern Mariana Islands; country: United States of America; locality: Northwest Eifuku Volcano vent field, near Champagne vent; verbatimDepth: 1615 m; verbatimLatitude: 21°29.2383'N; verbatimLongitude: 144°02.4937'E; **Event:** samplingProtocol: suction sampler mounted on ROV KM-ROV, dive #213; eventDate: 07/03/2023; habitat: white bacterial mats on rocks around diffuse flow venting; **Record Level:** institutionCode: NSMT; basisOfRecord: PreservedSpecimen

#### Description

**Figure 6.**
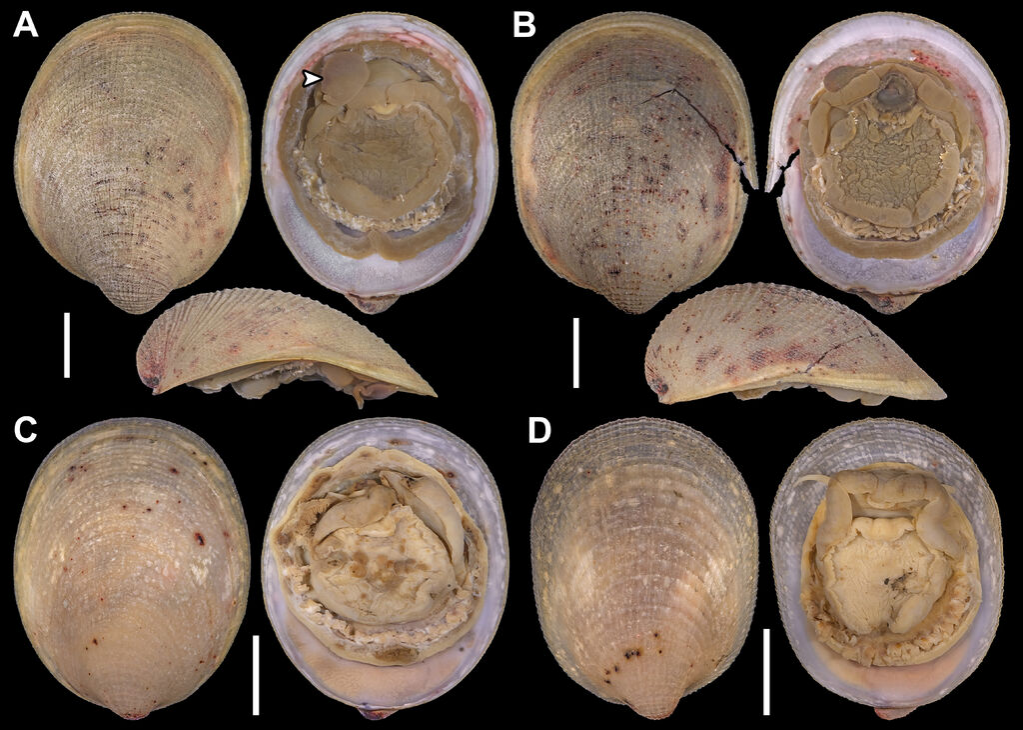
*Shinkailepasgigas* Chen, Watanabe & Tsuda, **sp. nov.**, habitus photographs of representative type specimens. **A.** holotype (SMF 373153), dorsal, ventral and lateral views, arrowhead on ventral view indicates the penis; **B.** Paratype 1 (NSMT-Mo 79486), dorsal, ventral and lateral views; **C.** Paratype 2 (SMF 373154), dorsal and ventral views; **D.** Paratype 3 (MNHN-IM-2019-34808), dorsal and ventral views. Scale bars: 5 mm.

**Figure 7.**
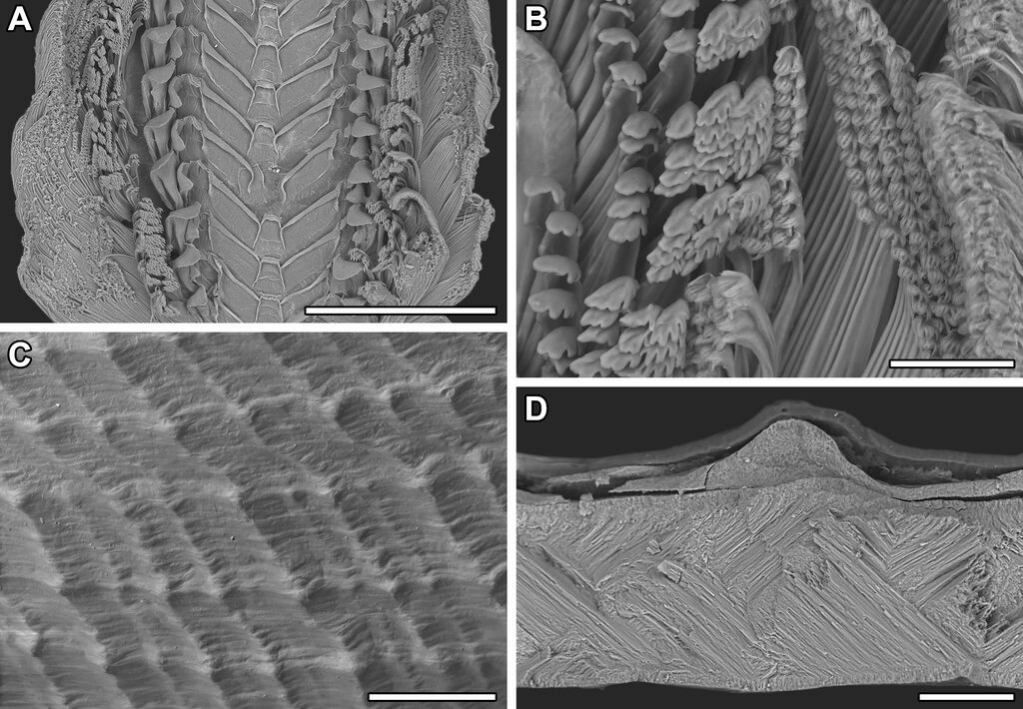
*Shinkailepasgigas* Chen, Watanabe & Tsuda, **sp. nov.**, scanning electron micrographs of paratype 4 (NSMT-Mo 79487). **A.** Radula overview; **B.** Close-up of marginal teeth; **C.** Shell sculpture; **D.** Fractured cross section of a shell showing microstructure. Scale bars: 500 μm (A, C), 50 μm (B, D).

Shell (Fig. 6): limpet form as adult with evidence of tighter coiling when juvenile, very large for genus. Shell dimensions of the holotype and paratypes are given in Table 3. Aperture oval, clear ventral columellar deck present posteriorly. Apex very posterior, protruding beyond posterior shell edge, slightly recurved to right side. Shell sculpture (Fig. 7C) finely cancellate with concentric ribs slightly stronger than radial ribs, minor protuberances occur where two ribs cross. Lateral profile of shell strongly curved. Shell microstructure (Fig. 7D) consisting of four layers: thin outermost dense homogenous layer followed by another thin granular homogenous layer which transitions to thick complex crossed lamellar layer then finally to thin simple prismatic layer. Protoconch corroded in all five adult specimens available for study.

External anatomy typical for the genus. Head large, broad, snout wide. Cephalic tentacles moderate in length. Males exhibit one large, swollen penis (Fig. 6A) just anterior of right cephalic tentacle. Mouth circular, oral disc papillated, oral lobes very large in size, extending laterally. Pallial margin swollen, simple, without tentacles. Foot circular, posterior half of epipodial fold serrated into approximately 50 paddle-like, broad projections. Operculum lacking in all five adults available for study.

Radula (Fig. 7A–B) rhipidoglossate, formula ca. 70-4-1-4-ca.70. Central tooth tall trapezoid, shaft thin, film-like, weakly developed with broad overhanging cusp, cutting edge smooth. Innermost laterals enlarged, three times as broad as rachidian, oblique in outline, shaft film-like; anterior edge rolled to form simple cusps lacking any serration. Second lateral narrower than rachidian, shaft sickle-like, with double overhanging cusps, upper cusp lacking serration, lower cusp shallowly serrated into about 10 cusps. Third lateral of similar breath to rachidian, twice as long as second lateral, cusp deformed, outer edge of shaft carrying broad lateral extension rolled inwards. Outermost lateral twice as large as third lateral, shaft divided into two columns, fusing anteriorly into one broad overhanging, simple cusp lacking serration. Inner marginals with semi-circular cusps serrated into 4-8 cusps, becoming more deeply serrated outwards. Outer marginals becoming much smaller in size with cusps serrated into several long denticles, gradually decreasing in size outwards.

#### Diagnosis

A very large *Shinkailepas* species up to 23 mm in shell length with apex bent to the right side and protruding beyond the posterior shell margin. Shell sculpture finely cancellate, with concentric ribs only slightly stronger than radial ones.

#### Etymology

The specific epithet *gigas* is Greek for ‘giant’; referring to the relatively large size of the species. Used as noun in apposition.

#### Distribution

Specimens of *S.gigas* Chen, Watanabe & Tsuda, **sp. nov.** with identities confirmed by COI barcoding have been collected from the hydrothermal vents on Northwest Eifuku and Northwest Rota volcanoes on Mariana Arc (Giguère and Tunnicliffe 2021) and the Susu vent field in Manus Basin (Poitrimol et al. 2022).

#### Taxon discussion

Previously available COI sequences of this species include one from Northwest Eifuku (MW807775) and one from Northwest Rota (MW807774), Mariana Arc published by Giguère and Tunnicliffe (2021), as well as two from Susu vent field, Manus Basin (OM264371-OM264372) published by Poitrimol et al. (2022). One new COI sequence was generated for this work from paratype 4 (NSMT-Mo 79487) and deposited in GenBank under the accession number OR640971. We did not examine specimens sequenced by Giguère and Tunnicliffe (2021) or Poitrimol et al. (2022), but their COI sequences are highly similar to ours with the uncorrected pairwise percentage genetic similarities being 99.3% or above across all pairs.

*Shinkailepasgigas* Chen, Watanabe & Tsuda, **sp. nov.** was already recognised as an undescribed species by Giguère and Tunnicliffe (2021). Molecular phylogenetic reconstruction using the COI gene by Poitrimol et al. (2022) confirmed its genetic distinctness from described *Shinkailepas* species, but it remained unnamed until now. Its most closely related known species, Poitrimol et al. (2022), is *Shinkailepaskaikatensis* Okutani, Saito & Hashimoto, 1989 with a known distribution from vents on the Izu-Ogasawara Arc (Kaikata Seamount and Myojin Knoll), Okinawa Trough (Yoron Hole) and Mariana Arc (Kasuga 2 Seamount and East Diamante Seamount) (Fukumori et al. 2019). Though *S.kaikatensis* and *S.gigas* **sp. nov.** have, so far, not been sampled from the same vent fields, their distribution ranges overlap on the Mariana Arc and could potentially occur sympatrically. Although neither Giguère and Tunnicliffe (2021) nor Poitrimol et al. (2022) applied particular species delimitation tools, their distinctness is supported by a lowest known uncorrected COI genetic distance of 4.2% (Poitrimol et al. 2022). Morphologically, *S.gigas* **n. sp.** is clearly distinct from other named *Shinkailepas* species including *S.kaikatensis* by combining the following morphological features: 1) a finely cancellate sculpture where concentric ribs are slightly stronger than radial ones; 2) a very posterior apex bending slightly to the right and protruding beyond the posterior shell edge and 3) a very large size (Okutani et al. 1989, Sasaki et al. 2003, Fukumori et al. 2019). The only described congener with comparable size and sculpture is *S.conspira* Beck in Chen & Sigwart, 2023, but the apex of *S.conspira* is more anterior and central compared to *S.gigas* **sp. nov.** (Chen and Sigwart 2023). Furthermore, the central and inner laterals in *S.gigas* **sp. nov.** are exceptionally weakly developed for the genus. In all five adult specimens of *S.gigas* **sp. nov.** the operculum was lacking and, therefore, the juvenile operculum is likely naturally lost in this species during growth.

#### Notes

##### Methods

As for *Lepetodrilusmarianae* Chen, Watanabe & Tsuda, **sp. nov.**, including the type specimen repositories SMF, NSMT and MNHN. Gastropods were collected near Champagne vent (Tunnicliffe et al. 2009), Northwest Eifuku Volcano vent field (21°29.2383'N, 144°02.4937'E), 1615 m depth, collected by suction sampler mounted on ROV *KM-ROV* dive #213, 2023/iii/07, R/V *KAIMEI* cruise KM23-05. Genetic barcoding of the mitochondrial cytochrome c oxidase subunit I (COI) gene was conducted using the universal primer pair HCO2198/LCO1490 (Folmer et al. 1994) following Chen et al. (2021).

### Lyonsiella illaesa

Machado & Sigwart sp. nov.

60E51ACC-06CA-5320-8447-26D5EF5FC449

15AAE398-6B82-49BD-B4D6-CAE63D90D144

#### Materials

- **Type status:** Holotype. **Occurrence:** catalogNumber: SMF 373402; recordedBy: AleutBio Expedition; individualCount: 1; occurrenceID: B722383F-07E8-577F-BB10-3F6DC87E2366; **Taxon:** scientificName: *Lyonsiellaillaesa* Machado & Sigwart; kingdom: Animalia; phylum: Mollusca; class: Bivalvia; order: Poromyida; family: Lyonsiellidae; genus: Lyonsiella; specificEpithet: *illaesa*; scientificNameAuthorship: Machado & Sigwart; nomenclaturalCode: ICZN; **Location:** higherGeography: Pacific Ocean; waterBody: U.S. Exclusive Economic Zone, Alaska Region, Aleutian Trench area; country: USA; locality: station 6-4; verbatimDepth: 5261-5318; verbatimLatitude: 50°37.959' N; verbatimLongitude: 169°44.368' W; **Event:** samplingProtocol: Epibenthic sledge (EBS); eventDate: 07/08/2022; **Record Level:** institutionCode: SMF; basisOfRecord: PreservedSpecimen
- **Type status:** Paratype. **Occurrence:** catalogNumber: SMF 374320; recordedBy: AleutBio Expedition; individualCount: 1; occurrenceID: 1AF2E5B8-C43B-5541-82FB-61C0C8BCD0AD; **Taxon:** scientificName: *Lyonsiellaillaesa* Machado & Sigwart; kingdom: Animalia; phylum: Mollusca; class: Bivalvia; order: Poromyida; family: Lyonsiellidae; genus: Lyonsiella; specificEpithet: *illaesa*; scientificNameAuthorship: Machado & Sigwart; nomenclaturalCode: ICZN; **Location:** higherGeography: Pacific Ocean; waterBody: U.S. Exclusive Economic Zone, Alaska Region, Aleutian Trench area; country: USA; locality: station 6-4; verbatimDepth: 5100-5170; verbatimLatitude: 51°41.711' N; verbatimLongitude: 166°28.024' W; **Event:** samplingProtocol: Epibenthic sledge (EBS); eventDate: 20/08/2022; **Record Level:** institutionCode: SMF; basisOfRecord: PreservedSpecimen

#### Description

**Figure 8.**
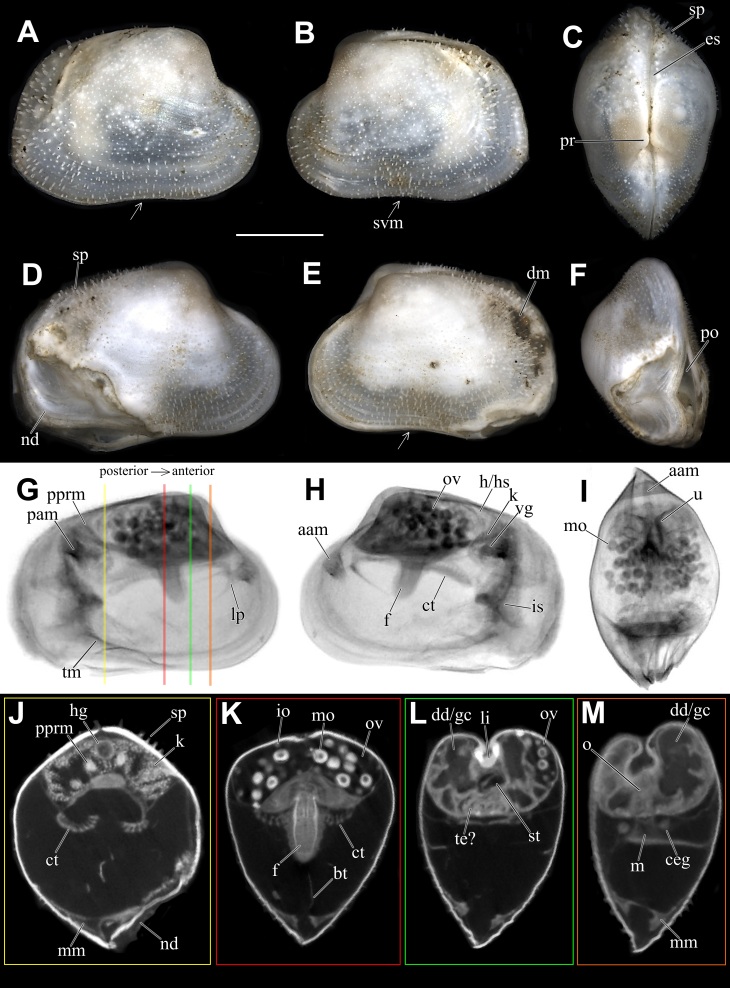
*Lyonsiellaillaesa* Machado & Sigwart, **sp. nov.**, outer view of shell and internal tissues. **1A–C.** Photomicrography of holotype (SMF 373402), right and left valves plus a dorsal view, respectively; **1D–M.** Paratype (SMF 374320). **1D**–**F.** Photomicrography of right and left valves plus a postero-ventral view, showing a natural deformity in the shell (nd) and a permanent opening (po) in the posterior margin; **1G**–**I**. X-ray images showing the arrangement of the pallial cavity organs and visceral mass; **1J**–**M.** Tomographic transverse sections of different parts of the specimen (yellow, red, green and orange squares). Abbreviations: aam, anterior adductor muscle; bt, byssal thread; ceg, cerebro-pleural (= circum-oesophagic glanglia); ct, ctenidia, dd/gc, digestive diverticula/gastric caecum; dm, detrital material attached; es, escutcheon; f, foot; h/hs, heart/haemocoel spaces; hg, hind gut; io, immature oocyte; is, inverted/contracted inhalant siphon; k, kidney; li, lithodesma; lp, labial palps; m, mouth; mm, mantle margin (ventral); mo, mature oocyte; nd, natural deformity in the shell; o, oesophagus; ov, ovary; pam, posterior adductor muscle; po, permanent opening in the shell; pprm, posterior pedal retractor muscle; pr, prodissoconch; sp, spikes; svm, sinuous ventral margin; te?, testis; tm, taenioid muscle, u, umbones. Scale bars: 1 mm (A–M).

Holotype: SMF 373402, one whole individual with soft parts. Dimensions: Length: 2.7 mm; Height: 1.8 mm; Width: 1.6 mm (Fig. 8A-C).

Paratype: SMF 374320, one whole individual with soft parts. Dimensions: Length: 3.1 mm; Height: 2 mm; Width: 1.8 mm (Fig. 8D–M). This individual presents a natural deformity (nd) at the shell margin with subsequent repairs and a permanent opening (po) on the posteroventral margin, both probably caused by a teratological process during embryonic development. Similar deformities have already been observed in other species of verticordiid, such as *Policordiauschakovi* (Gorbunov, 1946), *Policordialisbetae* Knudsen, 1970, *Policordiaochotica* Scarlato, 1981 and *Lyonsiellasubquadrata* (Jeffreys, 1882) (see Knudsen (1970), fig. 89, Allen and Turner (1974), fig. 26b, Safonova and Krylova (2020), figs. 3 and 4b).

*Shell*: small (up to 3.1 mm in length), whitish to translucent, inflated, inequilateral; subrectangular, anterior margin rounded, posterior margin slightly truncate, ventral margin sinuous (svm/white arrows); inequivalve; umbones inflated, slightly prosogyrate; prodissoconch (200 ± 5 μm, n = 2), circular, smooth, preserved in the two individuals analysed (Fig. 8C, pr). Shell surface spiked (Fig. 8C, D and J, sp), densely occupied by series of long and pointed projections (= spikes) extending over all margins; small pustules or granules (= pustule-shaped spikes) in umbones and central part of shell, sometimes arranged radially; indistinct radial threads and concentric growth lines. Right valve overlapping left valve on dorsal and antero-posterior margins (Fig. 8B and C); lunule absent; shallow escutcheon (Fig. 8C, es); hinge edentulous; with lithodesma (Fig. 8L, li).

Anatomy:

*Mantle margin*: with two fused points, anteriorly forming short pedal gape and posteriorly forming siphonal apertures; posteroventrally, mantle margin fusion (Fig. 8J–M, mm) involving inner folds only (Type A) (Yonge 1982); absence of fourth pallial aperture. No evidence of arenophilic glands (= radial mantle glands) and/or arenophilic secretions in outer surface of the shell. Perhaps functions of camouflage, strengthening thin shells and anchoring/stability in soft substrata, usually associated with such glands (Prezant 1985, Machado et al. 2017, Morton and Machado 2019), are performed here by the thousands of spikes on the outer surface of the shell.

*Siphons*: Separated; inhalant siphon inverted (Fig. 8H, is), probably cone-shaped; both surrounded by small tentacles at base, ~ 8 in inhalant and ~ 3 in exhalant siphon, only a single row of tentacles was observed. Similar to other carnivorous anomalodesmatan, *L.illaesa* **sp. nov.** can be also characterised as ‘lie-in-wait’ micro-predator (Machado et al. 2017, Morton et al. 2019, Morton and Machado 2019), that is camouflaged within its habitat by detrital material attached to its shell by its thousands of spikes and capturing prey by eversion of the cone-shaped inhalant siphon.

*Ctenidia*: Reduced, non-plicate and horizontally aligned (Fig. 8H, J and K, ct); incomplete, with only inner demibranch, moderately shorter filaments in comparison with other lyonsiellids (e.g., *Lyonsiellasubquadrata*, *Lyonsiellafragilis* Allen & J. F. Turner, 1974) and range in number from 8 to 10.

*Labial palps*: Non-lamellate, outer and inner palps short, unfused and elongated tips forming two wing-like lateral flaps (Fig. 8G, lp).

*Musculature*: Posterior and anterior adductor muscles present, isomyarian; with posterior and anterior pedal retractor muscle; posterior pedal retractor muscle bifurcated and dorsally attached to the shell close to the posterior adductor muscle (Fig. 8G, pprm); presence of taenioid muscle (= inhalant siphon retractor muscle) (Fig. 8G, tm).

*Foot*: Small, unpronounced heel (Fig. 8G and K, f); presence of a single byssal thread (Fig. 8K, bt).

*Digestive system*: Funnel-shaped mouth opening into a long oesophagus (Fig. 8M m, o) that enters into the anterodorsal portion of the stomach (Fig. 8L, st); stomach small, rounded, no crystalline style sac, no prey inside, surrounded dorsally, anteriorly and laterally by the gonads and the digestive gland; digestive diverticula with large gastric caecum (= blind ending tubules on both sides of the stomach walls) (Fig. 8L and M, dd/gc).

*Organs of visceral mass*: Pericardium/heart area well delimited (Fig. 8H, h/hs); paired kidneys restricted to the most posterior part of the body close to the posterior adductor muscle, posterior pedal retractor muscle and hind gut (Fig. 8H and J, k, pprm, hg). A lacunal system, formed by haemocoel spaces, is present in the posterior portion of the visceral mass, associated with the kidney and pericardium (Fig. 8H, h/hs). This system is commonly reported for members of Verticordioidea and appears to be an exclusive feature of this superfamily, having been associated with an important prey capture mechanism (Morton 1984).

*Reproductive system*: Probably hermaphroditic, ovary well visible (> 30 mature oocytes counted, ~ 150 μm in diameter) (Fig. 8H, I, K and L, ov); at least two stages of gametogenesis observed, i.e. immature oocytes - small cells attached in the wall of the ovarian follicle and mature oocytes - larger and usually free in the ovarian lumen (Fig. 8H, I, K and L, io, mo); testis relatively small, oval and probably located in the anteroventral portion of visceral mass close to the ventral wall of the stomach (Fig. 8L, te?). It is also worth highlighting the possibility of *L.illaesa* **sp. nov.** having a brooding behaviour, since it presents some indirect evidence, for example, larval prodissoconch size, small dimensions of the body (miniaturisation) and the possibility of mature oocytes are, in fact, encapsulated oocytes (post-fertilisation). The brooding care has already been described for other anomalodesmatans, such as members of the Spheniopsidae (Morton et al. 2016a, Morton et al. 2016b) and hypothesised for the verticordiid *Trigonulinaornata* d'Orbigny, 1853 (Morton et al. 2019).

*Nervous system*: Cerebro-pleural (Fig. 8M, ceg), visceral (Fig. 8H, vg) and pedal ganglia present; statocysts were not observed associated with the latter.

#### Diagnosis

Shell whitish to translucent, thin, inflated, subrectangular, ventral margin sinuous; outer surface ornamented by spikes (> 1000) covering entire shell surface, except for prodissoconch (pr); umbones inflated and slightly prosogyrate; radial lines absent; hinge edentate; posterior pedal retractor muscle bifurcated; presence of taenioid muscle; ctenidia reduced, with only inner demibranch, horizontally aligned; absence of crystalline style sac; paired kidney restricted to the most posterior part of the body; presence of a single byssal thread; probably hermaphrodite with large oocytes.

#### Etymology

Relative to the absence of any damage caused during the morphological description. The Latin word *illaesa* [*in* (“non”) +‎ *laesa* (“lesion”, “wounded”, “damaged")]. For the first time, a new species of mollusc is described in detail (including shell and internal tissues) without the use of any invasive tool.

#### Distribution

Known only from the Aleutian Islands, off Alaska, North Pacific. Bathymetric range: 5,100–5,318 m, a new record for the genus previously recorded at 4,429 m in the North Atlantic (see Poutiers and Bernard (1995)).

#### Taxon discussion

This species was compared with all morphologically similar species of the genus and differs from the other Pacific species, such as *Lyonsiellaquaylei* F. R. Bernard 1969 (Coan and Valentich-Scott 2012, plate 326), *Lyonsiellamagnifica* Dall, 1913 (holotype USNM 266802), *Lyonsiellapacifica* Dall, 1908 (holotype USNM 110583), *Lyonsiellaquadrata* Hedley, 1907, *Lyonsiellaaotearoa* Dell, 1995 and others in having an outline more rectangular, a ventral margin sinuous, absence of radial lines and umbones more inflated and central (= less prosogyrate). These same features were also used to differentiate it from the Atlantic species *Lyonsiellafrielei* Allen & Turner 1974 (holotype MCZ 272672), *Lyonsiellaabyssicola* (Machado and Passos 2022, fig. 3j, MCZ 272772), *Lyonsiellasubquadrata* (syntype USNM 63238, MCZ 272774, 348035) and all its possible shell variations illustrated by Allen and Turner (1974), figs. 1–2, 26–27. Regarding anatomy of new species, some features, such as the number of ctenidia filaments and siphonal tentacles, posterior pedal retractor muscle bifurcated, a single byssal thread, absence of crystalline style sac, paired kidney restricted to the most posterior part of the body, can also be used to differentiate it from their congeners, although the first two may be associated with intraspecific variations in individual size and shape, perhaps related to age.

#### Notes

##### Methods

The two well-preserved specimens analysed here were collected in the eastern part of the Aleutian Trench, Alaska by SO-293 AleutBio Expedition, using epibenthic sledge (EBS). The new species individuals are part of a larger collection with more than 1,200 lots and 3,500 individuals of Mollusca collected between 2,500 and 7,500 m depth. Both were described using only non-invasive techniques/tools, such as photos by stereomicroscope (Nikon) and tomographic images using the micro-CT scanner TomoScope® XS Plus (Werth). Only the paratype was scanned, for this purpose, it was previously immersed in a contrast solution containing 0.3% phosphotungstic acid (PTA at a concentration of 99.995%) with 3% dimethyl sulphoxide (DMSO) in 95% ethanol by 7 days (adapted from Machado et al. (2019)). Scanning parameters were as follows: source voltage = 140 kV, current = 140 µA, exposure time = 666 ms, ignore images = 0, image quality = 10, voxel size = 3.56 µm, number of images per revolution = 1770, CTsensor = Kurzer_AAI_Size_007_XL, filter = no, drift correction = on, time of acquision = 3h 16 min. Images were reconstructed and analysed using the software WinWerth^®^ RAW Viewer, Viewer and 3D Viewer available in  https://www.werth.de/en/downloads.html. In addition, all the volumetric datasets are on-line available at the Harvard’s Dataverse under the link https://doi.org/10.7910/DVN/ARIDIS. The holotype and paratype are archived at Senckenberg mollusc collection in Frankfurt am Main (SMF). Other museum lots were also analysed for comparison with the new species, most of them available on the websites of the respective institutions (MCZ – Museum of Comparative Zoology, Harvard, https://mczbase.mcz.harvard.edu/SpecimenSearch.cfm?collection_id=1; USNM – Smithsonian National Museum of Natural History, https://collections.nmnh.si.edu/search/iz/).

### Lepechinella naces

Lörz & Engel sp. nov.

0CCD5367-ECF8-5675-8F69-DCC87EE7E8F5

0AF2DD6E-D401-46DD-BD0F-9AE7CA4023A9

#### Materials

- **Type status:** Holotype. **Occurrence:** catalogNumber: NHMW-CR-29747; recordedBy: IceDIVA2; individualCount: 1; sex: female; lifeStage: adult; associatedSequences: http://www.ncbi.nlm.nih.gov/nuccore/OR839896; occurrenceID: 1426EB5B-F7E9-52CC-9106-BF28033300EC; **Taxon:** scientificName: *Lepechinellanaces* Lörz & Engel; kingdom: Animalia; phylum: Arthropoda; class: Malacostraca; order: Amphipoda; family: Lepechinellidae; genus: Lepechinella; specificEpithet: *naces*; taxonRank: species; scientificNameAuthorship: Lörz & Engel; nomenclaturalCode: ICZN; **Location:** higherGeography: Atlantic Ocean; waterBody: North Atlantic Ocean; locality: Marine Protected Area NACES, SO 286 station 46; verbatimDepth: 3677 m; verbatimCoordinates: latitude longitude start to end 51.96, -38.989583 — 51.95755, -38.98883; decimalLatitude: 51.96; decimalLongitude: -38.989583; **Identification:** identifiedBy: Anne-Nina Lörz; dateIdentified: 2023; **Event:** samplingProtocol: Epibenthic sledge, dragged; eventDate: 27/11/2021; **Record Level:** institutionCode: NHMW; collectionCode: CR; basisOfRecord: Preserved Specimen
- **Type status:** Paratype. **Occurrence:** catalogNumber: NHMW-CR-29748; recordedBy: IceDIVA2; individualCount: 1; lifeStage: juvenile; associatedSequences: http://www.ncbi.nlm.nih.gov/nuccore/OR839894; occurrenceID: 4197F382-6B13-5917-A6A3-5ABC36EFB81C; **Taxon:** scientificName: *Lepechinellanaces* Lörz & Engel; kingdom: Animalia; phylum: Arthropoda; class: Malacostraca; order: Amphipoda; family: Lepechinellidae; genus: Lepechinella; specificEpithet: *naces*; taxonRank: species; scientificNameAuthorship: Lörz & Engel; nomenclaturalCode: ICZN; **Location:** higherGeography: Atlantic Ocean; waterBody: North Atlantic Ocean; locality: Marine Protected Area NACES, SO 286 station 46; verbatimDepth: 3677 m; verbatimCoordinates: latitude longitude start to end 51.96, -38.989583 — 51.95755, -38.98883; decimalLatitude: 51.96; decimalLongitude: -38.989583; **Identification:** identifiedBy: Anne-Nina Lörz; dateIdentified: 2023; **Event:** samplingProtocol: Epibenthic sledge, dragged; eventDate: 27/11/2021; **Record Level:** institutionCode: NHMW; collectionCode: CR; basisOfRecord: Preserved Specimen
- **Type status:** Other material. **Occurrence:** catalogNumber: NHMW-CR-29749; recordedBy: IceDIVA2; individualCount: 1; lifeStage: juvenile; associatedSequences: http://www.ncbi.nlm.nih.gov/nuccore/OR839895; occurrenceID: 664E1AC9-94D1-5F23-A5F6-052733033BAF; **Taxon:** scientificName: *Lepechinellanaces* Lörz & Engel; kingdom: Animalia; phylum: Arthropoda; class: Malacostraca; order: Amphipoda; family: Lepechinellidae; genus: Lepechinella; specificEpithet: *naces*; taxonRank: species; scientificNameAuthorship: Lörz & Engel; nomenclaturalCode: ICZN; **Location:** higherGeography: Atlantic Ocean; waterBody: North Atlantic Ocean; locality: Marine Protected Area NACES, SO 286 station 46; verbatimDepth: 3677 m; verbatimCoordinates: latitude longitude start to end 51.96, -38.989583 — 51.95755, -38.98883; decimalLatitude: 51.96; decimalLongitude: -38.989583; **Identification:** identifiedBy: Anne-Nina Lörz; dateIdentified: 2023; **Event:** samplingProtocol: Epibenthic sledge, dragged; eventDate: 27/11/2021; **Record Level:** institutionCode: NHMW; collectionCode: CR; basisOfRecord: Preserved Specimen

#### Description

**Figure 9.**
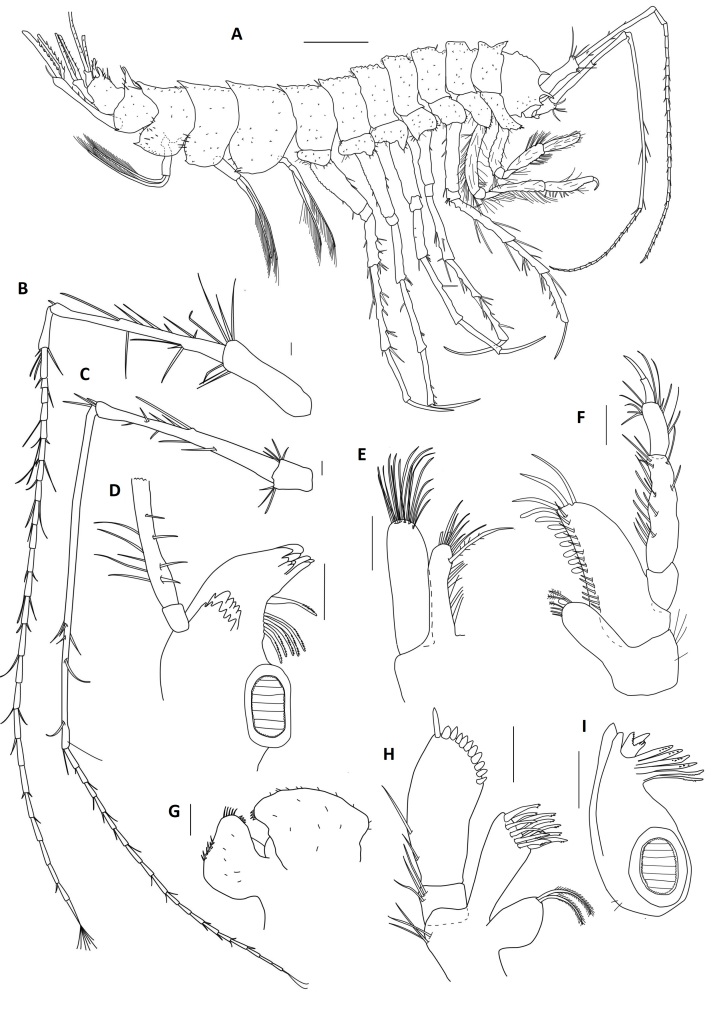
*Lepechinellanaces* Lörz & Engel, **sp. nov.**, NHMW-CR-29747, holotype, adult female, 7.3 mm. **A.** Habitus, right lateral view; **B.** Antenna 1; **C.** Antenna 2; **D.** Right mandible; **E.** Maxilla 2; **F.** Maxilliped; **G.** Hypopharynx; **H.** Maxilla 1; **I.** Left mandible. Scale bars: 1 mm (A), 0.1 mm (B–H).

**Figure 10.**
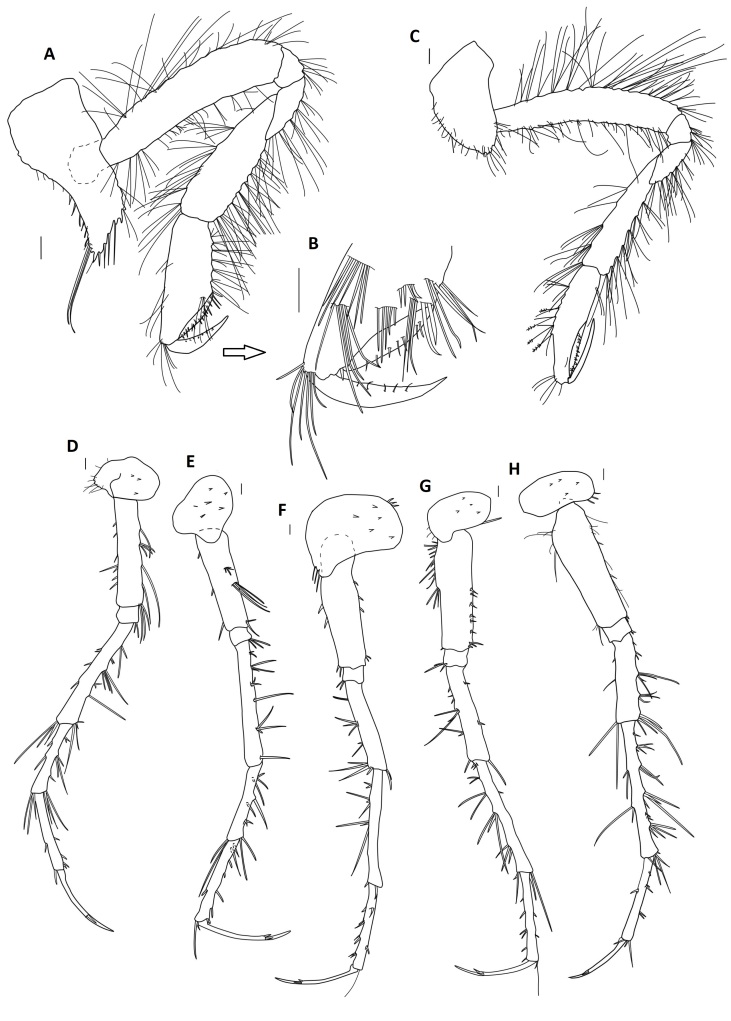
*Lepechinellanaces* Lörz & Engel, **sp. nov.**, NHMW-CR-29747, holotype, female, 7.3 mm. **A.** Pereopod 1; **B.** Detail of pereopod 1 palm; **C.** Pereopod 2; **D.** Pereopod 3; **E.** Pereopod 4; **F.** Pereopod 5; **G.** Pereopod 6; **H.** Pereopod 7. Scale bars: 0.1 mm.

**Figure 11.**
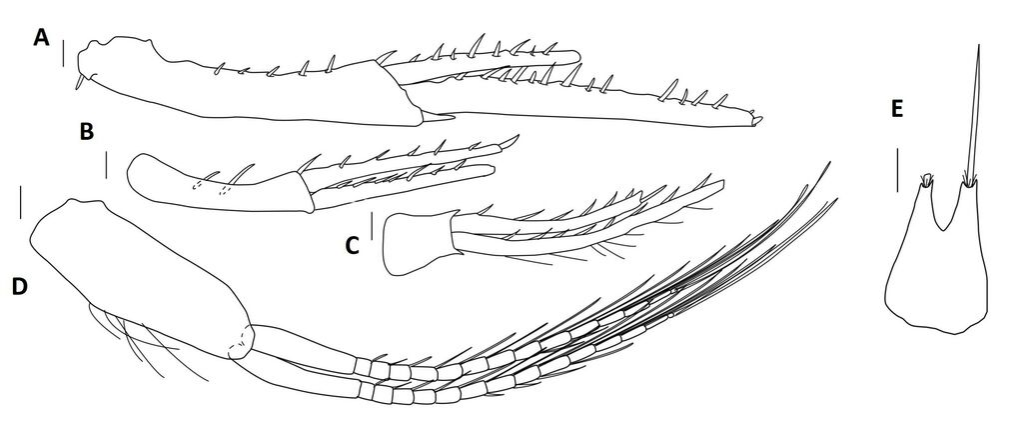
*Lepechinellanaces* Lörz & Engel, **sp. nov.**, NHMW-CR-29747, holotype, female, 7.3 mm. **A.** Uropod 1; **B.** Uropod 2; **C.** Uropod 3; **D.** Pleopod 2; **E.** Telson. Scale bars: 0.1 mm.

**Figure 12.**
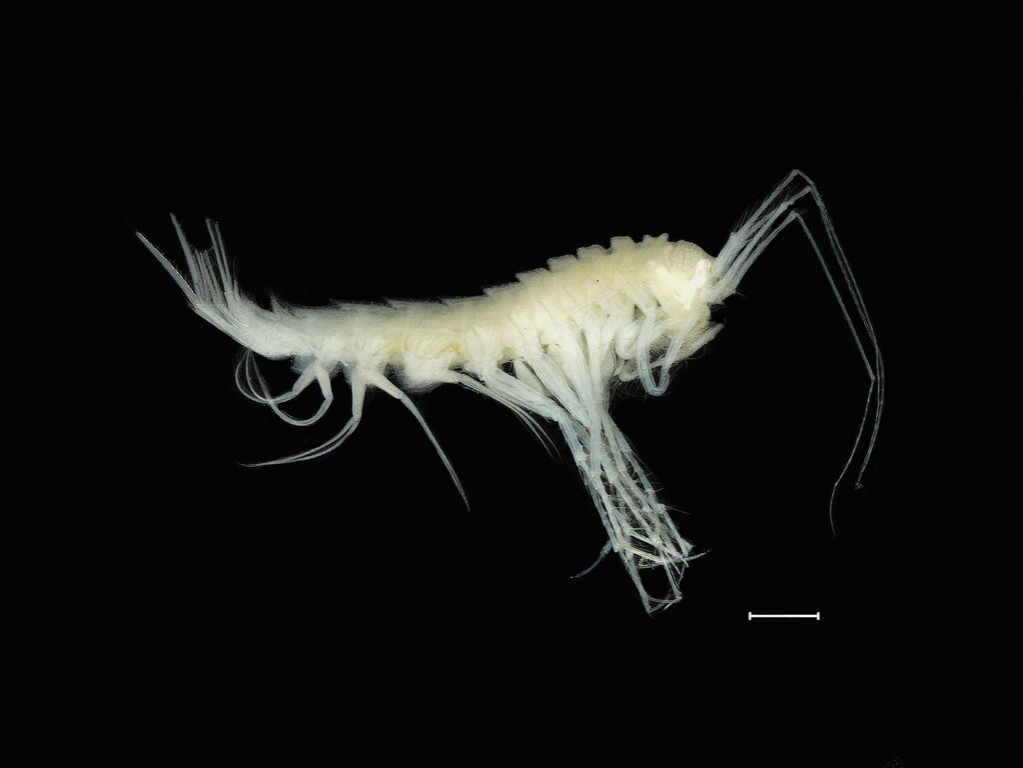
*Lepechinellanaces* Lörz & Engel, **sp. nov.**, NHMW-CR-29747, holotype, female, 7.3 mm. Habitus, right lateral view. Photographed in the lab via a Keyence 6000 microscope. Scale bar: 1 mm.

Holotype, NHMW-CR-29747, DZMB 10099, adult female, 7.3 mm, GenBank number OR839896. Figs 9, 10, 11, 12.

**Body** setose. **Head** with rostrum slightly curved, 20% of length of first peduncle art of A1; first cephalic tooth long, slender, longer than rostrum; second cephalic tooth acute. **A1**, almost reaching length of A2, shorter than length of body; first article 50% length of second art, bundle of setae at distal end; third art half the length of first art; flagellum 25 art. **A2**, art3, 4 and 5 with setae, art5 very slender, longer than art1–4 combined, with fourth art 60 % length of fifth art; flagellum 18 art.

**Md** with incisor process dentate, *lacina mobilis* dentate, molar triturative, palp broken off. **Mx1**, inner plate with two plumose setae; outer plate with 11 dentate spine teeth; palp second art distally expanded and slightly truncate, distal margin with 10 blunt spine teeth. **Mx2**, inner plate with one plumose seta, inner plate narrower and shorter than outer. **Mxp**, inner plate slender, distally rounded, distal setae; outer plate reaches to the last third of second art of palp, distal part of inner margin with short thick spines apically ending in long spines; palp four art, second art twice the length of third art. **Hypopharynx**, bilobate, inner lobes well developed extending to half of outer lobe.

**Prn and Pleon**, Prn1 with two upright teeth. Prn2–7 and pleon dorsally carinate, each carina terminating in a subacute, posteriorly directed tooth.

**P1**, coxa not bifid, anterodistally produced, distal margin convex, serrate; basis as long as ischium, merus and carpus combined; basis to propodus setose, palm half the length of posterior margin, dactylus smooth. **P2**, coxa marginally setose, tapering distally, anterior margin convex; propodus slender, slightly shorter than basis, palm almost half the length of posterior margin; dactylus small setae.

**P3 and P4**, similar in shape, coxae slightly bifid, lobes same length. **P5 and P6**, coxa anterior lobe pointed ventrally, setae on all segments. **P7**, coxa rounded, basis to propodus setose.

**Ep** convex posteriorly, each with prominent postero-distal tooth.

**Urosome** Urosomite 1 strongly produced dorsally, urosomite 3 produced extending to more than half of telson length.

**U1**, peduncle and outer ramus subequal; inner ramus broken; strong spines on peduncle and rami. **U2**, outer ramus slightly shorter than inner ramus, subequal in length to peduncle; peduncle and rami with strong spines. **U3**, peduncle very short, quarter of length of outer ramus; inner ramus similar length to outer ramus. **T**, cleft 30 %, lobes each with apical spine, telson length equals length of spine.

**Figure 13.**
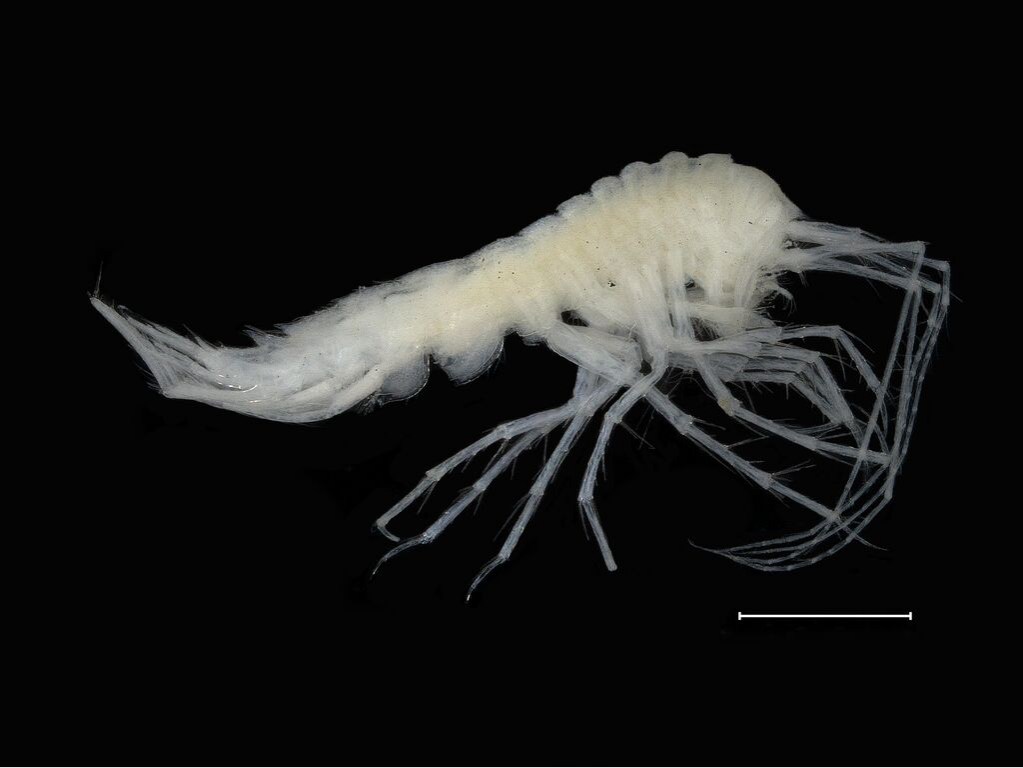
*Lepechinellanaces* Lörz & Engel, **sp. nov.**, NHMW-CR-29748, paratype, juvenile, 3.2 mm. Habitus, right lateral view. Photographed in the lab via a Keyence 6000 microscope. Scale bar: 1 mm.

**Variation**: The paratype NHMW-CR-29748, juvenile, 3.2 mm, GenBank number OR839894 (Fig. 13), morphologically differing from the adult (Figs 9, 10, 11, 12) in the absence of teeth on pereonite 1, weakly-developed carinae on the pereonites and pleonites. Morphological characters are known to vary between age and stages of growth amongst lepechinellid amphipods (Barnard 1973, Thurston 1980, Lörz et al. 2020). The non-type specimen NHMW-CR-29749, juvenile, 3 mm, collected at the same station as the holotype and paratype, was heavily damaged, but a barcode analysis (GenBank number OR839895) confirmed 100% similarity to the holotype.

#### Diagnosis

*Lepechinellanaces* Lörz & Engel, **sp. nov.** is characterised by a setose body, antenna subequal in length, shorter than body, two dorsal teeth on pereonite 1, coxa 1 tapering non-bifid, coxa 7 being rounded and the telson being cleft 30%.

#### Etymology

The specific epithet *naces* is a noun in apposition, referring to the high seas North Atlantic Current and Evlanov Sea Basin (NACES), a marine protected area (MPA), which covers nearly 600,000 km^2^.

#### Taxon discussion

*Lepechinellanaces* Lörz & Engel, **sp. nov.** is morphologically closest to *Lepechinellagrimi* Thurston, 1980 (Thurston 1980), *Lepechinellaocclo* Barnard, 1973 (Barnard 1973), *Lepechinellapangola* J.L. Barnard, 1962 (Barnard 1962) and *Lepechinellavictoriae* Johansen & Vader, 2015 (Johansen and Vader 2015); these five species bear two distinct extensions on pereonite 1. Table 4 lists differential characters.

The closest hits, when blasting the COI sequences of *L.naces* **sp. nov.** in BOLD and GenBank, were a *Lepechinella* specimen not identified to species level, collected in the Kuril Kamchatka Trench (Jażdżewska and Mamos 2019) with 6% difference and *L.grimi* from the North Atlantic (Lörz et al. 2020) with 15% difference.

A further species of *Lepechinella*, which we identified as *Lepechinella* sp. (NHMW-CR-29750, GenBank number OR839893) was collected at the same station as *L.naces* **sp. nov.** This specimen morphologically resembles L. *skarphedini*, which has not been genetically sequenced to date. It differed from *L.naces* **sp. nov.** by comparison of the COI segment by more than 20%.

#### Notes

##### Methods

The IceDIVA2 expedition, initiated and coordinated by the department German Centre for Marine Biodiversity (DZMB), Senckenberg, took place in November and December 2021 via RV Sonne (Brix et al. 2022) in the North Atlantic. Material collected from the Marine Protected Area NACES SO 286 Station 46 by an epibenthic sledge (Brenke 2005) was sorted on board and in the laboratories of the DZMB Hamburg using a Leica 12.5 stereoscope following the protocol described by Riehl et al. (2014b) with preservation ensuring an undisturbed cooling chain. All lepechinellid specimens sampled are stored at the Crustacea Division, Zoology Department, Natural History Museum Vienna, Austria.

Amphipod length was measured from the tip of the rostrum to the end of the telson. The appendages of the left side of the holotype were dissected and temporarily mounted on a slide in glycerine for illustration. Terminology of setae and spines follows Garm and Watling (2013). Pencil drawings were made under a stereoscope Leica MDG33 and microscope Leitz Diaplan Type 020-437.035 via a camera lucida and digitally inked via the free software Vectr, Pixlr. Photographs of the specimens were made via a Keyence 6000 microscope.

The molecular methods follow the protocol of Schwentner and Lörz (2020) for the cytochrome c oxidase subunit 1 (COI), the primers LCO1490-JJ/HCO2198-JJ were unchanged from Astrin and Stüben (2008). All specimens have a GenBank reference number for their barcode region of the COI.

### Cuniculomaera

Tandberg & Jażdżewska gen. nov.

E0DD3647-FCA0-5FF5-AB9C-FDDA5D98C51B

796926BB-EE87-4145-B91C-6F90496FA6DA

**Composition**: One valid species, which is described herein; *Cuniculomaeragrata* Tandberg & Jażdżewska, **sp. nov.**
Cuniculomaera grata Tandberg & Jażdżewska. Type species.

#### Diagnosis

Small round eyes. Accessory flagellum 8-articulate. Pereopod 2 propodus suboval, palmar corner not sharply defined, dactylus smooth and sharp. Epimeral plates smooth, epimeral plate 3 with sharp tooth. Uropod 3 elongate, rami of similar length, covered by bushy setae. Telson deeply and widely cleft.

#### Etymology

*Cuniculomaera* is a compound noun formed from the Latin second-declension noun *cuniculus* (burrow) and the suffix -maera from *Maera*, the type genus of the family Maeridae. Gender: feminine.

#### Taxon discussion

The decision to erect a new genus for *Cuniculomaeragrata* Tandberg & Jażdżewska, **sp. nov.** (described below) comes from it differing in diagnostic characters from all initially eligible genera within the family Maeridae Krapp-Schickel, 2008. The placement of the genus into Maeridae follows the discussion in Krapp-Schickel (2008), where she separates the *Ceradocus-Elasmopus-Maera*-group from Melitidae Bousfield, 1973, based on the equiramous uropod 3 in this group compared with the parviramous uropod 3 in Melitidae. The use of the defining morphological characters of uropod 3 is kept by Lowry and Myers (2013), who also keeps Maeridae separate from the morphologically quite similar Hadziidae S. Karaman, 1943 due to the morphology of the gills. It should also be noted that Hadziidae are exclusively freshwater species. The gills of *Cuniculomaeragrata* **sp. nov.** are not stalked (rather “normal” for marine species), again placing the new genus within Maeridae.

**Figure 14.**
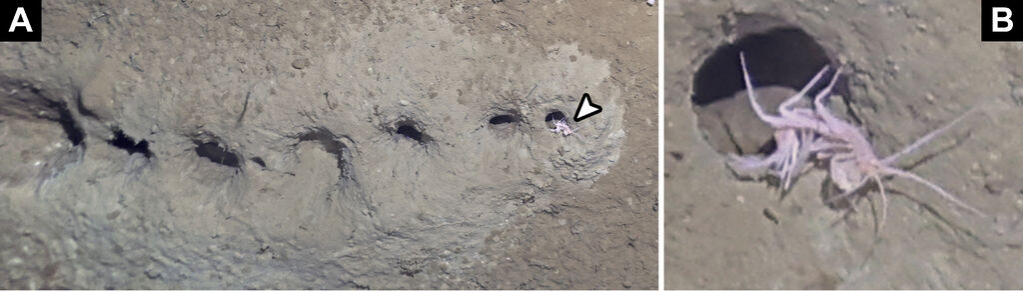
Burrows where *Cuniculomaeragrata* Tandberg & Jażdżewska, **sp. nov.** is suggested to live. Photos from AleutBio expedition. **A.** Amphipod in last of burrow openings; **B.** Zoom in of amphipod from A.

The equiramous uropod 3 of *Cuniculomaera* **gen. nov.** can be observed in Fig. 14. Sadly, uropod 3 was lost in the specimen that was sampled. Placing it in the *Maera* Leach, 1814 s. l. group (*sensu* Krapp-Schickel 2008) comes from it differing from *Ceradocus* A. Costa, 1853 in its epimeral plate 3 not being serrated (it is smooth, with a clear tooth), the shape of the telson (the lobes being slender and with two small teeth compared to the one tip on each lobe “*Ceradocus*-telson”) and the dorsal shape of the urosome which is smooth compared to the “can have teeth” of *Ceradocus*. It differs from *Elasmopus* A. Costa, 1853 by having a long accessory flagellum (not short as for *Elasmopus*) and in the shape of the mandibular palp article 3 (the *Elasmopus* article is falcate, *Cuniculomaera* has this palp straight) and epimeral plate 3 posterior corner being a sharp tooth compared to the *Elasmopus* blunt corner.

*Maera* s. l. was split into several genera by Krapp-Schickel (2008); the present new genus can be separated from these by the combination of the accessory flagellum, eye-shape, pereopods 1 and 2 shapes, epimeral plate 3 posterior corner and telson-shapes. It also differs in its depth range, albeit this is not a diagnostic character.

The obtained sequence was positively checked as belonging to Amphipoda against the Barcode of Life Data System (BOLD) and GenBank databases; however, with only ca. 80% similarity to published sequences. The closest relative in GenBank was *Uristesgigas* Dana, 1852 (78.97% similarity, acc. No. MH825809.1) while in BOLD: *Wimvadocustorelli* (Goës, 1866) (79.97%, sample ID: BSM08T16-01, record not available in GenBank). The number of publicly available sequences of Maeridae is relatively low (164 sequences of which 62 belong to only two species), while the family is represented by 423 species grouped in 48 genera (Horton et al. 2023). A phylogenetic analysis was not conducted; only the barcode of the new species is provided for future use.

### Cuniculomaera grata

Tandberg & Jażdżewska sp. nov.

8B798ACE-F6F5-5AEF-8953-C8C32FCE5C4E

B0A3C83D-95E3-4DB0-A8B0-313FBAC54A8E

Maeridae: Brandt et al. (2022): 107, Table 5.18; p 109, fig. 5.60M.
“maerid amphipod”: Brandt et al. (2022): 131, text.
*Maera* sp.: Brandt et al. (2023): 4, text.
“maerid amphipod”: Brandt et al. (2023): 4, fig. 2.

#### Materials

- **Type status:** Holotype. **Occurrence:** catalogNumber: SMF-61334; recordNumber: AB447; recordedBy: Anne Helene S. Tandberg; individualCount: 1; sex: male; lifeStage: adult; preparations: EtOH 96%; otherCatalogNumbers: 1-10-Maer_2022_1; previousIdentifications: Maeridae; associatedReferences: https://doi.org/10.1002/ece3.9867; associatedSequences: BOLD DS-CUNIFOS | GenBank: PP131298; occurrenceID: B8A04BB2-2DCE-5212-8BDF-AB6AC6D12CD7; **Taxon:** scientificName: *Cuniculomaeragrata* Tandberg & Jażdżewska; kingdom: Animalia; phylum: Arthropoda; class: Malacostraca; order: Amphipoda; family: Maeridae; genus: Cuniculomaera; specificEpithet: *grata*; taxonRank: species; scientificNameAuthorship: Tandberg & Jażdżewska; **Location:** higherGeography: Northern Pacific Ocean; waterBody: Bering Sea; country: USA; locality: Bering Sea AleutBio expedition station SO-293_1-10; minimumDepthInMeters: 3504; maximumDepthInMeters: 3516; locationRemarks: endpoint 54°32.495' N 172°32.014' W, 3504 m depth, water temperature 1.6°C; verbatimLatitude: 54°31.419' N; verbatimLongitude: 172°36.594' W; georeferenceProtocol: Ship GPS; **Identification:** identifiedBy: A.H.S. Tandberg; dateIdentified: 2023; **Event:** samplingProtocol: Epibenthic sled with camera (C-EBS); eventDate: 27/07/2022; **Record Level:** institutionCode: SMF; basisOfRecord: PreservedSpecimen

#### Description

**Figure 15.**
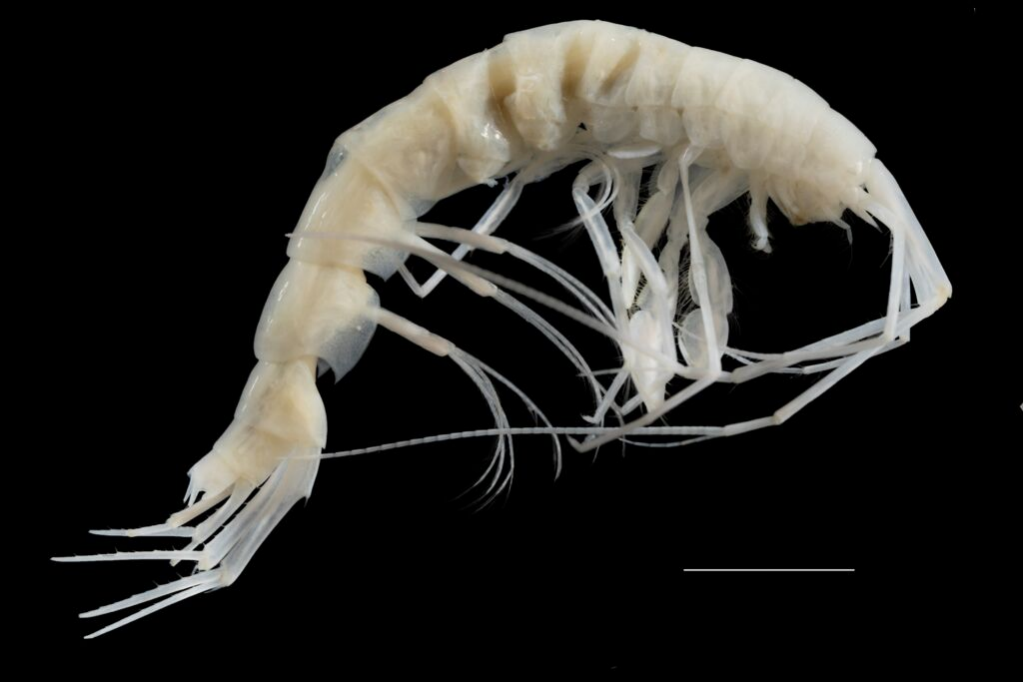
*Cuniculomaeragrata* Tandberg & Jażdżewska, **sp. nov.**, male holotype (SMF-61334). Habitus photography. Scale bar: 5 mm.

Body dorsally smooth (Fig. 15), elongate with short coxae. Length 24 mm.

**Figure 16.**
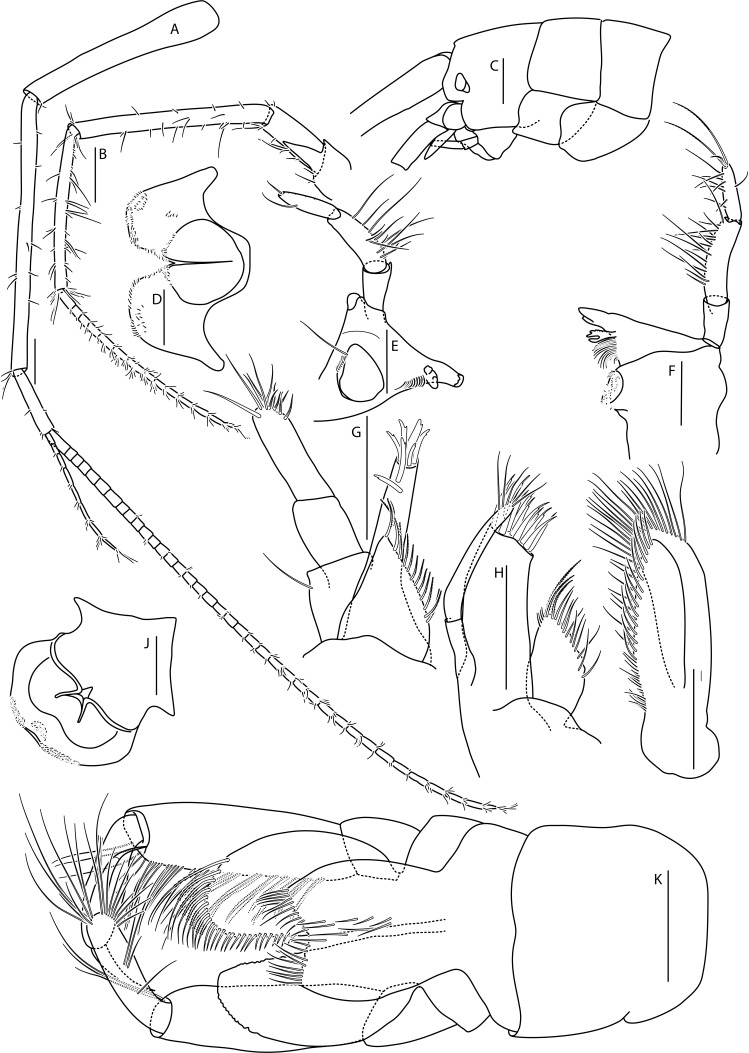
*Cuniculomaeragrata* Tandberg & Jażdżewska, **sp. nov.**, male holotype (SMF-61334). **A.** Antenna 1; **B.** Antenna 2; **C.** Head; **D.** Labrum; **E.** L mandible; **F.** R mandible; **G.** R Maxilla 1; **H.** L maxilla 1; **I.** Maxilla 2; **J.** Labium; **K.** Maxilliped. Scale bars: 1 mm (A–C), 0.5 mm (D–K).

**Head** (Fig. 16C) shorter than deep, cephalic lobes rounded, eye small (0.5x height of A1 insert) and roundish. **A1** (Figs 15, 16A) as long as body; art2 longer than art1 which is longer than art3; acc flag 8-articulate and ¼ of flagellum length, all art with 2 short setae; flagellum with approximately 40 art, short thin setae on each. **A2** (Fig. 16B) half the length of A1, art4 and 5 subequal in length, both with several thin setae; flagellum 15-articulate, all art with several short setae. **Lbr** (Fig. 16D) symmetric. **Md** (Fig. 16E, F) molar triturative; palp 3-articulate, art2 longer than art3 which is longer than art1, art3 is straight, art2–art3 setose; incisor smooth and bifurcate; 11 raker setae decreasing in thickness from incisor towards molar; *lacinia mobilis* on right Md. **Mx1** (Fig. 16G, H) inner plate subtriangulate, bushy setae distally; outer plate elongate and blunt with 7 cusped setae distally, palp 2-articulate with 6 long and 5 short straight setae distally on art2. **Mx2** (Fig. 16I) inner and outer lobe subequal, inner lobe strongly setose along inner margin. **Lbi** (Fig. 16J) slightly notched, symmetrical. **Mxp** (Fig. 16K) inner plate totally separate, distal margin concave and heavily setose; outer plate reaching ⅔ of palp art2 sensory setae along distal and inner margin; palp art3 4x as long as art2, heavily setose inner margin; dactylus ½ art3 length.

**Figure 17.**
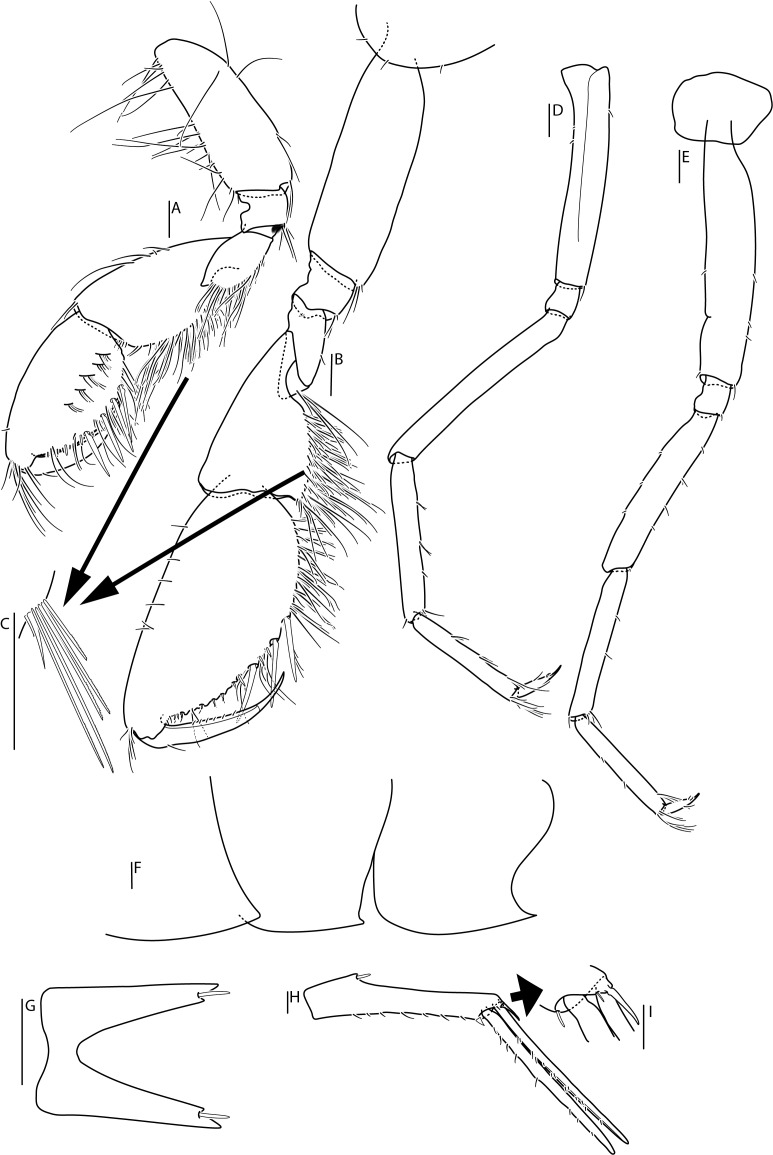
*Cuniculomaeragrata* Tandberg & Jażdżewska, **sp. nov.**, male holotype (SMF-61334). **A.** Pereopod 1; **B.** Pereopod 2; **C.** Seta on posterior margin of carpus, P1 and P2; **D.** Pereopod 3; **E.** Pereopod 4; **F.** Epimeral plates 1–3; **G.** Telson; **H.** Uropod 1; **I.** close-up of U1 peduncle distal portion. Scale bars: 0.5 mm.

**Pereon**: All coxae shown on Fig. 15 (habitus photo). **P1** (Fig. 17A and C) C1 (Fig. 16C) with small rounded extension on distal anterior corner; basis straight, anterior margin with long setae; ischium and merus short with distal margins setose; carpus suboval, sensory setae (Fig. 17C) along posterior margin; propodus suboval, palmar corner weakly defined by strong setae, palm and hind margin strongly setose, palm oblique; dactylus strong and sharp. **P2** (Fig. 17B and C) slightly larger than P1; C2 (Fig. 16C) rounded with anterior margin bulging, smooth distal margin; basis straight; ischium and merus short; carpus subtriangulate heavily setose with sensory setae (Fig. 17C) along posterior margin; propodus suboval, palm oblique and weakly serrate with palmar corner defined by 3 large setae, hind margin and palm with sensory setae, along hind margin in tufts; dactylus sharp, inner margin with 6 short setae. **P3** (Fig. 17D) and **P4** (Fig. 17E) straight long and thin, longer than P1 and P2, dactyli with sensory setae. **P5**–**P7** only known from *in situ* photo (Fig. 14) and video, where they are clearly longer than P1–P4, straight and slim.

**Pleon: Ep1–Ep3** (Fig. 17F) all smooth, Ep1 posterodistal corner with rounded extension, Ep2 posterodistal corner with small rounded tooth, Ep3 posterodistal corner medium-sized sharp tooth.

**Urosome**: Smooth (Fig. 15), **U1** (Fig. 17H) peduncle with spur 1/6 length of rami, rami of equal length, slim, short setae along outer margins. **U2** (Fig. 15) peduncle half length of U1 peduncle, rami similar length to U1 rami. **U3** (Fig. 14) longer than U1 and U2, rami similar length, wide and setose. **T** (Fig. 17G) deeply (80%) cleft, gaping, lobes thin with slightly concave inner margin, two short teeth at tip, one strong seta at each tip.

**Colour** *in vivo* (Fig. 14): light pink.

#### Diagnosis

As for the genus.

#### Etymology

The Latin feminine noun *grata*, meaning "favourite", alludes to the term "favourite burrows" given originally to the strange sediment constructions recorded on the sea bottom by the members of AleutBio expedition while watching and analysing the video footages from the Bering Sea. Now we know they were constructed by the presently-described species, so we call it favourite as well.

#### Taxon discussion

See discussion for the genus.

#### Notes

##### Methods

During the AleutBio expedition to the Bering Sea and Aleutian Trench during summer 2022 (Brandt et al. 2022), a visual inspection of the seafloor using the video- and photo platform OFOS (Ocean Floor Observation System) documented several series of burrows in the soft sediments of the Bering Sea. Thorough examinations of the videos and photos showed that these burrows were connected below the seafloor surface and it was suggested that these tunnels were created by amphipods (Brandt et al. 2023). The specific amphipod was designated, but not formally described until now.

The epibenthic sled equipped with camera (C-EBS (Brandt et al. 2013)) at AleutBio Station SO-293-1-10 contained one specimen - confirmed with OFOS photo of an amphipod in a burrow (Fig. 1) to most possibly be the burrow maker. Material from EBS cod-end was immediately sieved (1 mm) in a cold room (4°C) and examined through a cold chain (Riehl et al. 2014b) before a pleopod was removed for genetic analysis and the remaining specimen was fixed on 96% undenatured ethanol before storage at 4°C until morphological examination. The specimen was photographed using a Canon EOS5 after fixation due to time restraints onboard and re-photographed using a Leica photography and stacking system (LAS-X), dissected and drawn from glycerol slides using a Leica M125C stereoscope and drawn with a camera lucida fitted on a Leica DM2500 LED (facilities of SOSA Senckenberg, Frankfurt). The drawings were inked using Adobe Illustrator (version CC 2023).

The DNA extraction from the described individual was performed on board with 70 μl InstaGene™ Matrix (BIO-RAD). The digestion was done at 56°C for 40 min. The DNA barcoding fragment of cytochrome-*c*-oxidase subunit I gene (COI; ca. 670 bp) was amplified using LCO1490-JJ (CHACWAAYCATAAAGATATYGG) and HCO2198-JJ (AWACTTCVGGRTGVCCAAARAATCA) primers (Astrin and Stüben 2008) with DreamTaq Green PCR Mastermix (Thermo Scientific) and reaction conditions following Hou et al. (2007). Sequencing was done bidirectionally using the Applied Biosystems 3730xl capillary sequencer by Macrogen Europe, the Netherlands. Sequences were edited using Geneious 10.1.2 leading to a fragment of 658 bp (excluding primers). The sequences were blasted using default parameters on NCBI BLAST and translated into amino-acid sequences to confirm that no stop codons were present. The sequence was included in the Barcode of Life Data System database (Ratnasingham and Hebert 2007) in the project devoted to Amphipoda collected during the AleutBio cruise and is available in the dedicated dataset: DS-CUNIFOS (http://boldsystems.org/index.php/MAS_Management_DataConsole?codes=DS-CUNIFOS doi: dx.doi.org/10.5883/DS-CUNIFOS) as well as in GenBank (Acc. No. PP131298).

The holotype is deposited in the crustacean collections of Senckenberg Naturmuseum in Frankfurt, Germany, with the collections registration-number SMF-61334. The video-material is stored in the same institution. As the species is so different from other genera, the comparison was performed against the available literature for the different genera and for the family Maeridae (e.g., Barnard and Karaman 1991, Krapp-Schickel and Jarrett 2000, Krapp-Schickel 2008, Ariyama et al. 2020). The description follows the terminologies presented in Krapp-Schickel (2008).

### Pseudionella pumulaensis

Williams & Landschoff sp. nov.

B5EC48E9-CC16-59AE-A7E0-9681388A3957

5E7C8F85-472C-47EB-95FC-619BCAC3D571

"undescribed *Pseudionella* sp.": Landschoff et al. (2018): 2, 12: figs. 6D, 14.

#### Materials

- **Type status:** Holotype. **Occurrence:** catalogNumber: SAMC-A096401; occurrenceRemarks: infesting left branchial chamber of male *Pagurusfraserorum* Landschoff & Komai in Landschoff et al., 2018 (2.4 mm shield length; SAMC-A066407); recordedBy: Jannes Landschoff; individualCount: 1; sex: female; lifeStage: adult; reproductiveCondition: egg-bearing; preparations: 96% EtOH; occurrenceID: 04AA858B-3D1B-51FB-8E57-FCE19D28C43C; **Taxon:** scientificName: *Pseudionellapumulaensis* Williams & Landschoff; kingdom: Animalia; phylum: Arthropoda; class: Malacostraca; order: Isopoda; family: Bopyridae; genus: Pseudionella; specificEpithet: *pumulaensis*; taxonRank: species; scientificNameAuthorship: Williams & Landschoff; nomenclaturalCode: ICZN; **Location:** higherGeography: Indian Ocean; continent: Africa; country: South Africa; stateProvince: KwaZulu-Natal; locality: off Pumula; verbatimDepth: 20 m; verbatimLatitude: S 30° 38.34’; verbatimLongitude: E 30° 32.94’; **Identification:** identifiedBy: J.D. Williams, J. Landschoff; **Event:** samplingProtocol: SCUBA; eventDate: 14/10/2015; habitat: rocky subtidal reef; **Record Level:** institutionCode: SAMC; basisOfRecord: PreservedSpecimen
- **Type status:** Paratype. **Occurrence:** catalogNumber: SAMC-A096402; recordedBy: Jannes Landschoff; individualCount: 1; sex: male; lifeStage: adult; preparations: 96% EtOH; occurrenceID: F2F76206-2B7A-5616-95A0-4694C9BDAE05; **Taxon:** scientificName: *Pseudionellapumulaensis* Williams & Landschoff; kingdom: Animalia; phylum: Arthropoda; class: Malacostraca; order: Isopoda; family: Bopyridae; genus: Pseudionella; specificEpithet: *pumulaensis*; taxonRank: species; scientificNameAuthorship: Williams & Landschoff; nomenclaturalCode: ICZN; **Location:** higherGeography: Indian Ocean; continent: Africa; country: South Africa; stateProvince: KwaZulu-Natal; locality: off Pumula; verbatimDepth: 20 m; verbatimLatitude: S 30° 38.34’; verbatimLongitude: E 30° 32.94’; **Identification:** identifiedBy: J.D. Williams, J. Landschoff; **Event:** samplingProtocol: SCUBA; eventDate: 14/10/2015; habitat: rocky subtidal reef; **Record Level:** institutionCode: SAMC; basisOfRecord: PreservedSpecimen

#### Description

**Figure 18.**
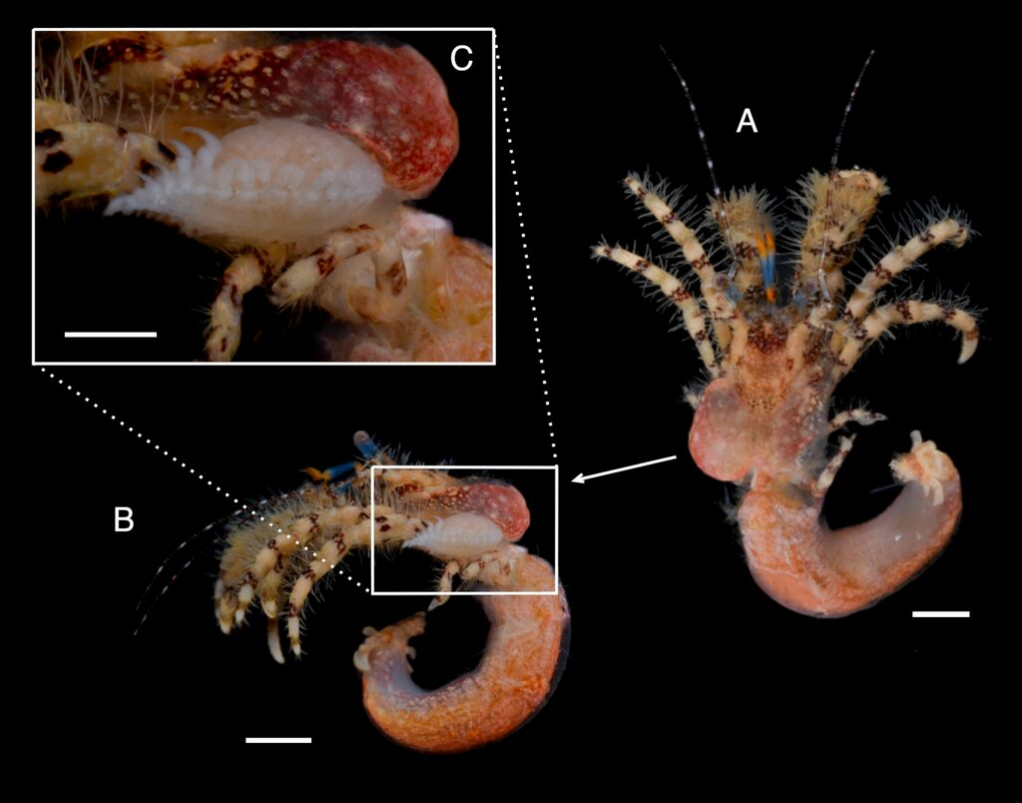
Live image of *Pagurusfraserorum* Landschoff & Komai in Landschoff et al., 2018 and *Pseudionellapumulaensis* Williams & Landschoff, **sp. nov. A.** Dorsal view of *P.fraserorum* with inflated left branchial chamber; **B.** Left lateral view with branchial chamber showing *P.pumulaensis* sp. nov.; **C.** Close-up view of B. Scale bars: 2 mm (A, B), 1 mm (C).

**Figure 19.**
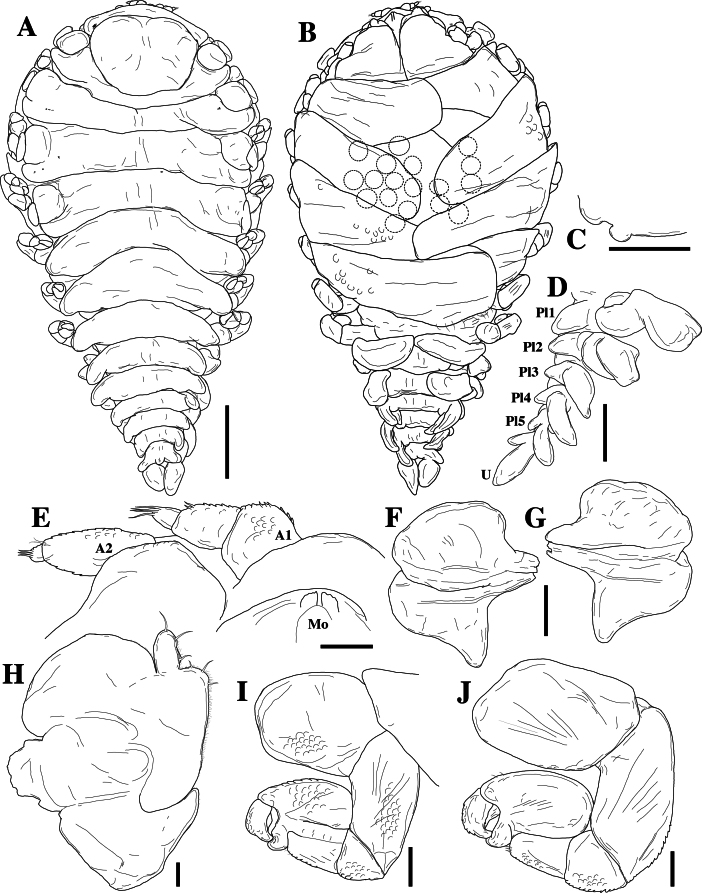
*Pseudionellapumulaensis* Williams & Landschoff, **sp. nov.**, adult female holotype (SAMC A096401). **A.** Habitus, dorsal view; **B.** Habitus, some eggs shown in dashed lines, ventral view; **C.** Right barbula; **D.** Pleon, right lateral view; **E.** Right antennule, antenna and mouthparts, ventral view; **F.** Right oostegite 1, internal view; **G.** Right oostegite 1, external view; **H.** Right maxilliped, external view; **I.** Left pereopod 1; **J.** Left pereopod 7. Abbreviations: A1 = antennula, A2 = antenna, Mo = mouthparts, Pl1–Pl5 = pleomeres 1–5, U = uropod. Scale bars: 500 µm (A, B), 250 µm (C, D, F, G), 50 µm (E, H–J).

**Female holotype** (Figs 18, 19) body length 3.4 mm, maximum width 1.8 mm, head length 0.5 mm, head width 0.7 mm, pleon length 1.1 mm. Body tear-drop-shaped (Fig. 18B and C, Fig. 19A and B), approximately symmetrical, head slightly sinistrally rotated (< 5°). Very small patches of pigment present on some Prn (Fig. 19A).

**Head** subquadrate (Fig. 19A) with slight medial indentation at anterior end, narrow frontal lamina extending slightly beyond lateral margins of head. Small eyes near posterolateral corners of raised lamina edge. Barbula with two pairs of small, smooth rounded lobes (Fig. 19C). **A1** (Fig. 19E) of three art each, extending beyond anterior margin of head; **A2** (Fig. 19E) of three art each, but basal art broadly rounded and fused with head (see Remarks); all art of A1 and A2 with spinous scales and distal setae. Mxp (Fig. 19H) anterior lobe broad, rounded, with unarticulated palp with few large setae and many fine setae, lateral edge of lobe setose; posterior lobe with small rounded spur.

**Prn** (Fig. 19A) of seven Prn, broadest across Prn3, tapering anteriorly and posteriorly. Small coxal plates on sides of pereomeres all similar, but larger on left side of body; dorsolateral bosses on Prn1–Prn4. First oostegite anterior lobe ovate, posterior lobe subtriangular, internal ridge smooth (Fig. 19F and G). Oostegites 3–5 with some minute circular tubercles; oostegite 5 with fringe of setae on posterior margin (Fig. 19B). **P1–7** (Fig. 19I, J) slightly increasing in size posteriorly and of similar morphology, small curved dactylus, ovate propodus indistinctly separated from triangular carpus, small triangular merus, elongate ischium and subquadrate basis; all art with spinous scales, propodus with few small setae on anterolateral margin, carpus with stout setae at anterior tip (Fig. 19I and J).

**Pleon** (Fig. 19B and D) of six Pl. **Pl1–Pl5** with uniramous, digitiform Plp (Fig. 19B and D), first two Plp with indentation giving appearance of biramous morphology, but each a single lobe (possible fusion of endopod and exopod); uniramous rounded lateral plates (Fig. 19B and D); lateral plates and Plp reduced in size from anterior to posterior. **Plt** (Fig. 19B and D) with sixth pair of lateral plates projecting laterally and pair of large broad, distally rounded uniramous uropods.

**Eggs** (Fig. 19B) approximately 195 in number, a few potentially lost in transfer, 150.3 ± 9.9 µm in diameter (n = 30).

**Figure 20.**
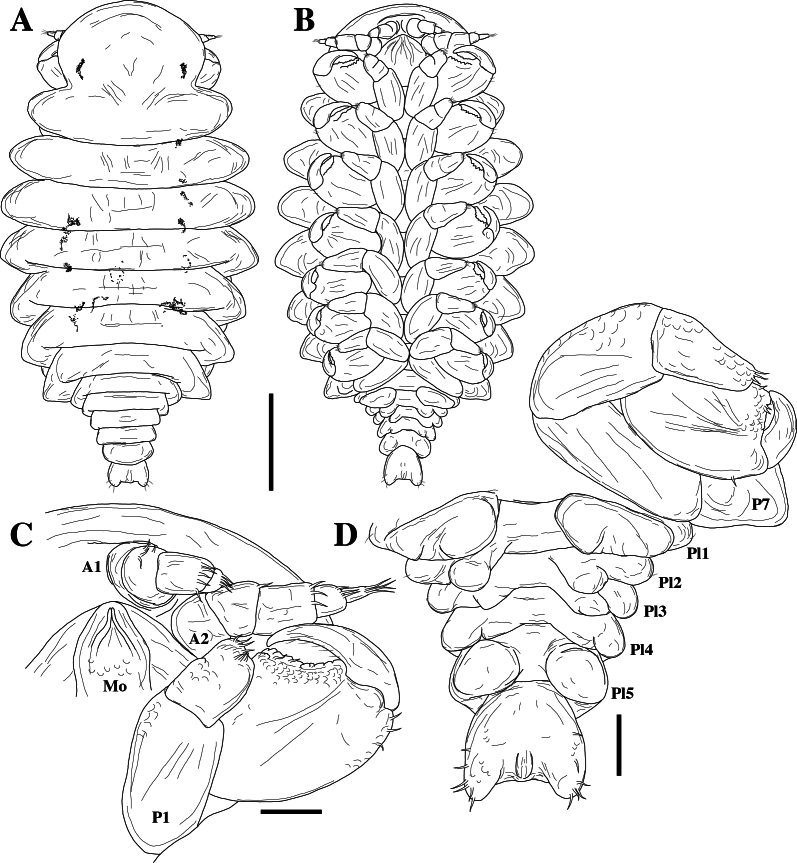
*Pseudionellapumulaensis* Williams & Landschoff, **sp. nov.**, adult male paratype (allotype) (SAMC A096402). **A.** Habitus, dorsal view; **B.** Habitus, ventral view; **C.** Left antennula, antenna, mouthparts and pereopod 1, ventral view; **D.** Left pereopod 7 and pleon, ventral view. Abbreviations: A1 = antennule, A2 = antenna, Mo = mouthparts, P1 = pereopod 1, P7 = pereopod 7, Pl1–Pl5 = pleomeres 1–5. Scale bars: 250 µm (A, B), 50 µm (C, D).

**Male paratype (allotype)** (Fig. 20) body length 1.3 mm, maximum width 0.7 mm, head length 0.2 mm (not including Prn1), head width 0.4 mm, pleon length 0.3 mm. Body suboval-shaped, squat. Small, irregular patches of dark pigmentation on nearly all Prn.

**Head** fused with Prn1, anterior margin of head broadly rounded (Fig. 20A), irregularly-shaped eyes near posterolateral margin. **A1** of three art each; **A2** of five art each, extending slightly beyond margin of head; A2 and A1 with few spinous scales and distal setae (Fig. 20C).

**Pereon** of seven Prn, broadest across pereomere 4, tapering anteriorly and posteriorly. Lateral margins of Prn1–3 directed anteriorly, Prn4–7 directed posteriorly. **P1** (Fig. 20C) largest, slightly decreasing in size posteriorly; all art distinct, curved dactylus with minute setae, ovate propodus, subquadrate carpus, elongate merus and ischium, small subquadrate basis; all art with spinous scales, propodus scales and spines adjacent to dactylus plus few setae on anterolateral margin, carpus with stout setae at anterior tip; **P7** (Fig. 20D) similar in morphology to P1, except dactylus shorter and carpus more triangular in shape.

**Pleon** (Fig. 20D) of six Pl, markedly narrower than pereon, Pl tapering posteriorly, all Pl distinctly segmented and bearing lateral plates, Pl1­–4 with pleopods indistinctly separated from lateral plates, Pl5 with broad, rounded lateral plates; no mid-ventral tubercles (Fig. 20D). **Plt** (Fig. 20D) notched medially with anal cone, produced distolaterally into rounded lobes, with scales and setae; Urp absent.

#### Diagnosis

Female body nearly straight, barbula with two small, smooth lobes on each side, all pleopods uniramous. Male head fused with pereomere 1, antennulae and antennae of 3 and 5 articles, respectively.

#### Etymology

The specific epithet *pumulaensis* refers to the locality of Pumula in KwaZulu-Natal, South Africa, where the species was first discovered. The word Pumula is Zulu and means "a place of rest".

#### Distribution

KwaZulu-Natal, east coast of South Africa (Indian Ocean); rocky subtidal reef.

#### Ecology

**Host**: *Pagurusfraserorum* Landschoff & Komai in Landschoff et al. (2018) (Crustacea, Decapoda, Anomura, Paguridae Latreille, 1802), a hermit crab described from KwaZulu-Natal, South Africa. **Parasite location on host**: Branchial chamber (gill-parasitic). See Fig. 18 and Landschoff et al. (2018), page 14.

Only one out of nine specimens of *Pagurusfraserorum* Landschoff & Komai in Landschoff et al. (2018) was found parasitised by *P.pumulaensis* sp. nov. (11% overall prevalence). An additional specimen of *P.fraserorum* was reported by Landschoff et al. (2018) to have another branchial parasitic isopod (provisionally identified as *Pseudionella* sp.); unfortunately, that specimen no longer exists.

#### Taxon discussion

Formerly, *Pseudionella* was known to contain five species, which parasitise diogenid (1 species) and pagurid (4 species) hermit crabs from the South Pacific Ocean, North Pacific Ocean, North Atlantic Ocean, Caribbean Sea and South China Sea (Shiino 1949, Adkison and Heard 1978, Bourdon 1979, Boyko and Williams 2001, An et al. 2013). The new species of *Pseudionella* described herein parasitising *Pagurusfraserorum* Landschoff & Komai in Landschoff et al. (2018) is the first reported from the genus on hermit crabs from the Indian Ocean.

*Pseudionellapumulaensis* Williams & Landschoff, **sp. nov.** can be distinguished from the previously described species (*P.akuaku* Boyko & Williams, 2001, *P.attenuata* Shiino, 1949, *P.deflexa* Bourdon, 1979, *P.markhami* (Adkison & Heard, 1978) and *P.spiropaguri* An, Li & Markham, 2013), based on several characters. In contrast to females of *P.akuaku*, *P.attenuata*, *P.deflexa* and *P.markhami*, which are highly asymmetrical (dextral or sinistral deflexion of the head), *P.pumulaensis* and *P.spiropaguri* are nearly straight. In overall body form of females and males, *P.pumulaensis* and *P.spiropaguri* appear most similar, but can be distinguished, based on the following characters: barbula morphology (two small, smooth lobes on each side in *P.pumulaensis* vs. three lobes with digitate tips on each side in *P.spiropaguri*), female pleopod morphology (all uniramous in *P.pumulaensis* vs. first three biramous in *P.spiropaguri*), male antennae morphology (antennules and antennae of 3 and 5 articles, respectively in *Pseudionellapumulaensis* vs. 3 and 4 articles in *P.spiropaguri*) and male head fusion (fused with pereomere 1 in *P.pumulaensis* vs. separated in *P.spiropaguri*).

#### Notes

##### Methods

Hermit crabs containing the bopyrid isopod specimens were sampled under the University of Cape Town Science Faculty collection permit and Animal Ethics Committee approval, protocol number 2014/DC1/CLG. The host specimens of *Pagurusfraserorum* were all collected during two days of SCUBA diving on near-shore reefs off Pumula and Hibberdene, approximately 100 km south of Durban, KwaZulu-Natal, South Africa. Live pictures were taken after the process of anaesthetising (> 10 min in 0.125 ml/l clove oil-seawater solution), freezing and thawing of the specimens. Thereafter, specimens were preserved in 96% ethanol.

Line drawings of the parasite bopyrid isopods were made by using camera lucida drawing tubes attached to Olympus compound (Olympus CX41) and dissecting microscopes (Olympus sZX12). Adobe Illustrator and a Wacom Cintiq pen display were used to trace original sketches and produce final figures. Parasite sizes are given as maximum total length (TL). All specimen measurements were made from camera lucida drawing tube sketches and slide micrometers. Morphological terminology follows that of Boyko and Williams (2001) and An et al. (2013). Specimens of the new species were deposited at the Iziko South African Museum, Cape Town, South Africa (SAMC).

### Mastigoniscus minimus

Wenz, Knauber & Riehl sp. nov.

8C464EA2-104A-50D6-968A-2748D5D858B5

4CCBA599-209D-43B0-BCFA-40C140832E28

#### Materials

- **Type status:** Holotype. **Occurrence:** catalogNumber: SMF 56475; recordNumber: KB2 Hap193; recordedBy: Angelika Brandt | Saskia Brix | Nils Brenke | Magdalena Blazewicz | Olga Golovan | Nele Heitland | Anna Jazdzewska | Karen Jeskulke | Gennady Kamenev | Anna Lavrenteva | Marina Malyutina | Torben Riehl; individualCount: 1; sex: male; lifeStage: adult; preparations: EtOH 96%; associatedSequences: https://www.ncbi.nlm.nih.gov/nuccore/OM782632.1; occurrenceID: A82AEA3B-B81A-55EF-BCA1-F8EB9D0106AB; **Taxon:** scientificName: *Mastigoniscusminimus* Wenz, Knauber & Riehl; kingdom: Animalia; phylum: Arthropoda; class: Malacostraca; order: Isopoda; family: Haploniscidae; genus: Mastigoniscus; specificEpithet: *minimus*; taxonRank: species; scientificNameAuthorship: Wenz, Knauber & Riehl; nomenclaturalCode: ICZN; **Location:** waterBody: Northwest Pacific Ocean; locality: Kuril-Kamchatka Trench, abyssal plains southeast of the Trench, KuramBio II expedition, station SO250-008; verbatimDepth: 5136 m; decimalLatitude: 43.82; decimalLongitude: 151.77; **Identification:** identificationID: KB2 Hap193; identifiedBy: Christian Wenz; dateIdentified: 2022; **Event:** samplingProtocol: Epibenthic Sledge; eventDate: 19/08/2016; habitat: deep-sea sediment; fieldNumber: SO250-008; **Record Level:** institutionCode: SMF; basisOfRecord: PreservedSpecimen
- **Type status:** Paratype. **Occurrence:** catalogNumber: SMF 56301; recordNumber: KB2 Hap003; recordedBy: Angelika Brandt | Saskia Brix | Nils Brenke | Magdalena Blazewicz | Olga Golovan | Nele Heitland | Anna Jazdzewska | Karen Jeskulke | Gennady Kamenev | Anna Lavrenteva | Marina Malyutina | Torben Riehl; individualCount: 1; sex: male; lifeStage: adult; preparations: EtOH 96%; occurrenceID: 56A6E05A-D536-5612-BC62-19F348987A7A; **Taxon:** scientificName: *Mastigoniscusminimus* Wenz, Knauber & Riehl; kingdom: Animalia; phylum: Arthropoda; class: Malacostraca; order: Isopoda; family: Haploniscidae; genus: Mastigoniscus; specificEpithet: *minimus*; taxonRank: species; scientificNameAuthorship: Wenz, Knauber & Riehl; nomenclaturalCode: ICZN; **Location:** waterBody: Northwest Pacific Ocean; locality: Kuril-Kamchatka Trench, abyssal plains southeast of the Trench, KuramBio II expedition, station SO250-008; verbatimDepth: 5136 m; decimalLatitude: 43.82; decimalLongitude: 151.77; **Identification:** identificationID: KB2 Hap003; identifiedBy: Christian Wenz; dateIdentified: 2022; **Event:** samplingProtocol: Epibenthic Sledge; eventDate: 19/08/2016; habitat: deep-sea sediment; fieldNumber: SO250-008; **Record Level:** institutionCode: SMF; basisOfRecord: PreservedSpecimen
- **Type status:** Paratype. **Occurrence:** catalogNumber: SMF 56477; recordNumber: KB2 Hap195; recordedBy: Angelika Brandt | Saskia Brix | Nils Brenke | Magdalena Blazewicz | Olga Golovan | Nele Heitland | Anna Jazdzewska | Karen Jeskulke | Gennady Kamenev | Anna Lavrenteva | Marina Malyutina | Torben Riehl; individualCount: 1; sex: female; lifeStage: adult; preparations: EtOH 96%; associatedSequences: https://www.ncbi.nlm.nih.gov/nuccore/OM782636.1; occurrenceID: 267360E1-3628-5C3D-BC7F-7EB68A09EF54; **Taxon:** scientificName: *Mastigoniscusminimus* Wenz, Knauber & Riehl; kingdom: Animalia; phylum: Arthropoda; class: Malacostraca; order: Isopoda; family: Haploniscidae; genus: Mastigoniscus; specificEpithet: *minimus*; taxonRank: species; scientificNameAuthorship: Wenz, Knauber & Riehl; nomenclaturalCode: ICZN; **Location:** waterBody: Northwest Pacific Ocean; locality: Kuril-Kamchatka Trench, abyssal plains southeast of the Trench, KuramBio II expedition, station SO250-008; verbatimDepth: 5136 m; decimalLatitude: 43.82; decimalLongitude: 151.77; **Identification:** identificationID: KB2 Hap195; identifiedBy: Christian Wenz; dateIdentified: 2022; **Event:** samplingProtocol: Epibenthic Sledge; eventDate: 19/08/2016; habitat: deep-sea sediment; fieldNumber: SO250-008; **Record Level:** institutionCode: SMF; basisOfRecord: PreservedSpecimen
- **Type status:** Other material. **Occurrence:** catalogNumber: SMF 56413; recordNumber: KB2 Hap131; recordedBy: Angelika Brandt | Saskia Brix | Nils Brenke | Magdalena Blazewicz | Olga Golovan | Nele Heitland | Anna Jazdzewska | Karen Jeskulke | Gennady Kamenev | Anna Lavrenteva | Marina Malyutina | Torben Riehl; individualCount: 1; sex: female; lifeStage: adult; preparations: EtOH 96%; associatedSequences: https://www.ncbi.nlm.nih.gov/nuccore/OM782634.1; occurrenceID: 0E3A6E43-357E-5BEA-8B2E-E8279CD5A66C; **Taxon:** scientificName: *Mastigoniscusminimus* Wenz, Knauber & Riehl; kingdom: Animalia; phylum: Arthropoda; class: Malacostraca; order: Isopoda; family: Haploniscidae; genus: Mastigoniscus; specificEpithet: *minimus*; taxonRank: species; scientificNameAuthorship: Wenz, Knauber & Riehl; nomenclaturalCode: ICZN; **Location:** waterBody: Northwest Pacific Ocean; locality: Kuril-Kamchatka Trench, hadal depths of the Trench, KuramBio II expedition, station SO250-040; verbatimDepth: 7081 m; decimalLatitude: 45.66; decimalLongitude: 152.95; **Identification:** identificationID: KB2 Hap131; identifiedBy: Christian Wenz; dateIdentified: 2022; **Event:** samplingProtocol: Epibenthic Sledge; eventDate: 29/08/2016; habitat: deep-sea sediment; fieldNumber: SO250-040; **Record Level:** institutionCode: SMF; basisOfRecord: PreservedSpecimen
- **Type status:** Other material. **Occurrence:** catalogNumber: SMF 56449; recordNumber: KB2 Hap167; recordedBy: Angelika Brandt | Saskia Brix | Nils Brenke | Magdalena Blazewicz | Olga Golovan | Nele Heitland | Anna Jazdzewska | Karen Jeskulke | Gennady Kamenev | Anna Lavrenteva | Marina Malyutina | Torben Riehl; individualCount: 1; sex: female; lifeStage: adult; preparations: EtOH 96%; associatedSequences: https://www.ncbi.nlm.nih.gov/nuccore/OM782627.1; occurrenceID: 829CC624-DDCF-5743-9531-7EEBB6E91D5E; **Taxon:** scientificName: *Mastigoniscusminimus* Wenz, Knauber & Riehl; kingdom: Animalia; phylum: Arthropoda; class: Malacostraca; order: Isopoda; family: Haploniscidae; genus: Mastigoniscus; specificEpithet: *minimus*; taxonRank: species; scientificNameAuthorship: Wenz, Knauber & Riehl; nomenclaturalCode: ICZN; **Location:** waterBody: Northwest Pacific Ocean; locality: Kuril-Kamchatka Trench, hadal depths of the Trench, KuramBio II expedition, station SO250-097; verbatimDepth: 6575 m; decimalLatitude: 44.11; decimalLongitude: 151.41; **Identification:** identificationID: KB2 Hap167; identifiedBy: Christian Wenz; dateIdentified: 2022; **Event:** samplingProtocol: Epibenthic Sledge; eventDate: 18/09/2016; habitat: deep-sea sediment; fieldNumber: SO250-097; **Record Level:** institutionCode: SMF; basisOfRecord: PreservedSpecimen
- **Type status:** Other material. **Occurrence:** catalogNumber: SMF 56450; recordNumber: KB2 Hap168; recordedBy: Angelika Brandt | Saskia Brix | Nils Brenke | Magdalena Blazewicz | Olga Golovan | Nele Heitland | Anna Jazdzewska | Karen Jeskulke | Gennady Kamenev | Anna Lavrenteva | Marina Malyutina | Torben Riehl; individualCount: 1; sex: female; lifeStage: adult; preparations: EtOH 96%; associatedSequences: https://www.ncbi.nlm.nih.gov/nuccore/OM782639.1; occurrenceID: D91C900F-69B0-5842-80B8-0A49A7CB1CB3; **Taxon:** scientificName: *Mastigoniscusminimus* Wenz, Knauber & Riehl; kingdom: Animalia; phylum: Arthropoda; class: Malacostraca; order: Isopoda; family: Haploniscidae; genus: Mastigoniscus; specificEpithet: *minimus*; taxonRank: species; scientificNameAuthorship: Wenz, Knauber & Riehl; nomenclaturalCode: ICZN; **Location:** waterBody: Northwest Pacific Ocean; locality: Kuril-Kamchatka Trench, hadal depths of the Trench, KuramBio II expedition, station SO250-097; verbatimDepth: 6575 m; decimalLatitude: 44.11; decimalLongitude: 151.41; **Identification:** identificationID: KB2 Hap168; identifiedBy: Christian Wenz; dateIdentified: 2022; **Event:** samplingProtocol: Epibenthic Sledge; eventDate: 18/09/2016; habitat: deep-sea sediment; fieldNumber: SO250-097; **Record Level:** institutionCode: SMF; basisOfRecord: PreservedSpecimen
- **Type status:** Other material. **Occurrence:** catalogNumber: SMF 56451; recordNumber: KB2 Hap169; recordedBy: Angelika Brandt | Saskia Brix | Nils Brenke | Magdalena Blazewicz | Olga Golovan | Nele Heitland | Anna Jazdzewska | Karen Jeskulke | Gennady Kamenev | Anna Lavrenteva | Marina Malyutina | Torben Riehl; individualCount: 1; sex: female; lifeStage: adult; preparations: EtOH 96%; associatedSequences: https://www.ncbi.nlm.nih.gov/nuccore/OM782638.1; occurrenceID: 15EC7754-BA81-5EF6-A91D-DC12C56C5C27; **Taxon:** scientificName: *Mastigoniscusminimus* Wenz, Knauber & Riehl; kingdom: Animalia; phylum: Arthropoda; class: Malacostraca; order: Isopoda; family: Haploniscidae; genus: Mastigoniscus; specificEpithet: *minimus*; taxonRank: species; scientificNameAuthorship: Wenz, Knauber & Riehl; nomenclaturalCode: ICZN; **Location:** waterBody: Northwest Pacific Ocean; locality: Kuril-Kamchatka Trench, hadal depths of the Trench, KuramBio II expedition, station SO250-097; verbatimDepth: 6575 m; decimalLatitude: 44.11; decimalLongitude: 151.41; **Identification:** identificationID: KB2 Hap169; identifiedBy: Christian Wenz; dateIdentified: 2022; **Event:** samplingProtocol: Epibenthic Sledge; eventDate: 18/09/2016; habitat: deep-sea sediment; fieldNumber: SO250-097; **Record Level:** institutionCode: SMF; basisOfRecord: PreservedSpecimen
- **Type status:** Other material. **Occurrence:** catalogNumber: SMF 56452; recordNumber: KB2 Hap170; recordedBy: Angelika Brandt | Saskia Brix | Nils Brenke | Magdalena Blazewicz | Olga Golovan | Nele Heitland | Anna Jazdzewska | Karen Jeskulke | Gennady Kamenev | Anna Lavrenteva | Marina Malyutina | Torben Riehl; individualCount: 1; sex: female; lifeStage: adult; preparations: EtOH 96%; associatedSequences: https://www.ncbi.nlm.nih.gov/nuccore/OM782641.1; occurrenceID: 0997E606-4FA8-50DB-AC35-E4312B0998A3; **Taxon:** scientificName: *Mastigoniscusminimus* Wenz, Knauber & Riehl; kingdom: Animalia; phylum: Arthropoda; class: Malacostraca; order: Isopoda; family: Haploniscidae; genus: Mastigoniscus; specificEpithet: *minimus*; taxonRank: species; scientificNameAuthorship: Wenz, Knauber & Riehl; nomenclaturalCode: ICZN; **Location:** waterBody: Northwest Pacific Ocean; locality: Kuril-Kamchatka Trench, hadal depths of the Trench, KuramBio II expedition, station SO250-097; verbatimDepth: 6575 m; decimalLatitude: 44.11; decimalLongitude: 151.41; **Identification:** identificationID: KB2 Hap170; identifiedBy: Christian Wenz; dateIdentified: 2022; **Event:** samplingProtocol: Epibenthic Sledge; eventDate: 18/09/2016; habitat: deep-sea sediment; fieldNumber: SO250-097; **Record Level:** institutionCode: SMF; basisOfRecord: PreservedSpecimen
- **Type status:** Other material. **Occurrence:** catalogNumber: SMF 56453; recordNumber: KB2 Hap171; recordedBy: Angelika Brandt | Saskia Brix | Nils Brenke | Magdalena Blazewicz | Olga Golovan | Nele Heitland | Anna Jazdzewska | Karen Jeskulke | Gennady Kamenev | Anna Lavrenteva | Marina Malyutina | Torben Riehl; individualCount: 1; sex: female; lifeStage: adult; preparations: EtOH 96%; associatedSequences: https://www.ncbi.nlm.nih.gov/nuccore/OM782630.1; occurrenceID: 87A48BC7-B676-580B-87A6-9211B44DB18A; **Taxon:** scientificName: *Mastigoniscusminimus* Wenz, Knauber & Riehl; kingdom: Animalia; phylum: Arthropoda; class: Malacostraca; order: Isopoda; family: Haploniscidae; genus: Mastigoniscus; specificEpithet: *minimus*; taxonRank: species; scientificNameAuthorship: Wenz, Knauber & Riehl; nomenclaturalCode: ICZN; **Location:** waterBody: Northwest Pacific Ocean; locality: Kuril-Kamchatka Trench, hadal depths of the Trench, KuramBio II expedition, station SO250-097; verbatimDepth: 6575 m; decimalLatitude: 44.11; decimalLongitude: 151.41; **Identification:** identificationID: KB2 Hap171; identifiedBy: Christian Wenz; dateIdentified: 2022; **Event:** samplingProtocol: Epibenthic Sledge; eventDate: 18/09/2016; habitat: deep-sea sediment; fieldNumber: SO250-097; **Record Level:** institutionCode: SMF; basisOfRecord: PreservedSpecimen
- **Type status:** Other material. **Occurrence:** catalogNumber: SMF 56454; recordNumber: KB2 Hap172; recordedBy: Angelika Brandt | Saskia Brix | Nils Brenke | Magdalena Blazewicz | Olga Golovan | Nele Heitland | Anna Jazdzewska | Karen Jeskulke | Gennady Kamenev | Anna Lavrenteva | Marina Malyutina | Torben Riehl; individualCount: 1; sex: male; lifeStage: adult; preparations: EtOH 96%; associatedSequences: https://www.ncbi.nlm.nih.gov/nuccore/OM782640.1; occurrenceID: 849B456A-A547-5E8D-A442-273357A9D955; **Taxon:** scientificName: *Mastigoniscusminimus* Wenz, Knauber & Riehl; kingdom: Animalia; phylum: Arthropoda; class: Malacostraca; order: Isopoda; family: Haploniscidae; genus: Mastigoniscus; specificEpithet: *minimus*; taxonRank: species; scientificNameAuthorship: Wenz, Knauber & Riehl; nomenclaturalCode: ICZN; **Location:** waterBody: Northwest Pacific Ocean; locality: Kuril-Kamchatka Trench, hadal depths of the Trench, KuramBio II expedition, station SO250-098; verbatimDepth: 6446 m; decimalLatitude: 44.10; decimalLongitude: 151.42; **Identification:** identificationID: KB2 Hap172; identifiedBy: Christian Wenz; dateIdentified: 2022; **Event:** samplingProtocol: Agassiz Trawl; eventDate: 19/09/2016; habitat: deep-sea sediment; fieldNumber: SO250-098; **Record Level:** institutionCode: SMF; basisOfRecord: PreservedSpecimen
- **Type status:** Other material. **Occurrence:** catalogNumber: SMF 56455; recordNumber: KB2 Hap173; recordedBy: Angelika Brandt | Saskia Brix | Nils Brenke | Magdalena Blazewicz | Olga Golovan | Nele Heitland | Anna Jazdzewska | Karen Jeskulke | Gennady Kamenev | Anna Lavrenteva | Marina Malyutina | Torben Riehl; individualCount: 1; sex: female; lifeStage: adult; preparations: EtOH 96%; associatedSequences: https://www.ncbi.nlm.nih.gov/nuccore/OM782626.1; occurrenceID: 907C249F-D568-5FF0-A044-D45B6A2AC835; **Taxon:** scientificName: *Mastigoniscusminimus* Wenz, Knauber & Riehl; kingdom: Animalia; phylum: Arthropoda; class: Malacostraca; order: Isopoda; family: Haploniscidae; genus: Mastigoniscus; specificEpithet: *minimus*; taxonRank: species; scientificNameAuthorship: Wenz, Knauber & Riehl; nomenclaturalCode: ICZN; **Location:** waterBody: Northwest Pacific Ocean; locality: Kuril-Kamchatka Trench, hadal depths of the Trench, KuramBio II expedition, station SO250-098; verbatimDepth: 6446 m; decimalLatitude: 44.10; decimalLongitude: 151.42; **Identification:** identificationID: KB2 Hap173; identifiedBy: Christian Wenz; dateIdentified: 2022; **Event:** samplingProtocol: Agassiz Trawl; eventDate: 19/09/2016; habitat: deep-sea sediment; fieldNumber: SO250-098; **Record Level:** institutionCode: SMF; basisOfRecord: PreservedSpecimen
- **Type status:** Other material. **Occurrence:** catalogNumber: SMF 56456; recordNumber: KB2 Hap174; recordedBy: Angelika Brandt | Saskia Brix | Nils Brenke | Magdalena Blazewicz | Olga Golovan | Nele Heitland | Anna Jazdzewska | Karen Jeskulke | Gennady Kamenev | Anna Lavrenteva | Marina Malyutina | Torben Riehl; individualCount: 1; sex: indet.; lifeStage: adult; preparations: EtOH 96%; associatedSequences: https://www.ncbi.nlm.nih.gov/nuccore/OM782631.1; occurrenceID: 71F73D3E-D528-5FA2-BB3D-B0EE912584F8; **Taxon:** scientificName: *Mastigoniscusminimus* Wenz, Knauber & Riehl; kingdom: Animalia; phylum: Arthropoda; class: Malacostraca; order: Isopoda; family: Haploniscidae; genus: Mastigoniscus; specificEpithet: *minimus*; taxonRank: species; scientificNameAuthorship: Wenz, Knauber & Riehl; nomenclaturalCode: ICZN; **Location:** waterBody: Northwest Pacific Ocean; locality: Kuril-Kamchatka Trench, hadal depths of the Trench, KuramBio II expedition, station SO250-098; verbatimDepth: 6446 m; decimalLatitude: 44.10; decimalLongitude: 151.42; **Identification:** identificationID: KB2 Hap174; identifiedBy: Christian Wenz; dateIdentified: 2022; **Event:** samplingProtocol: Agassiz Trawl; eventDate: 19/09/2016; habitat: deep-sea sediment; fieldNumber: SO250-098; **Record Level:** institutionCode: SMF; basisOfRecord: PreservedSpecimen
- **Type status:** Other material. **Occurrence:** catalogNumber: SMF 56467; recordNumber: KB2 Hap185; recordedBy: Angelika Brandt | Saskia Brix | Nils Brenke | Magdalena Blazewicz | Olga Golovan | Nele Heitland | Anna Jazdzewska | Karen Jeskulke | Gennady Kamenev | Anna Lavrenteva | Marina Malyutina | Torben Riehl; individualCount: 1; sex: female; lifeStage: adult; preparations: EtOH 96%; associatedSequences: https://www.ncbi.nlm.nih.gov/nuccore/OM782635.1; occurrenceID: C6FF82AE-95BC-55DF-94D3-BBE39C464CDB; **Taxon:** scientificName: *Mastigoniscusminimus* Wenz, Knauber & Riehl; kingdom: Animalia; phylum: Arthropoda; class: Malacostraca; order: Isopoda; family: Haploniscidae; genus: Mastigoniscus; specificEpithet: *minimus*; taxonRank: species; scientificNameAuthorship: Wenz, Knauber & Riehl; nomenclaturalCode: ICZN; **Location:** waterBody: Northwest Pacific Ocean; locality: Kuril-Kamchatka Trench, hadal depths of the Trench, KuramBio II expedition, station SO250-040; verbatimDepth: 7081 m; decimalLatitude: 45.66; decimalLongitude: 152.95; **Identification:** identificationID: KB2 Hap185; identifiedBy: Christian Wenz; dateIdentified: 2022; **Event:** samplingProtocol: Epibenthic Sledge; eventDate: 29/08/2016; habitat: deep-sea sediment; fieldNumber: SO250-040; **Record Level:** institutionCode: SMF; basisOfRecord: PreservedSpecimen
- **Type status:** Other material. **Occurrence:** catalogNumber: SMF 56468; recordNumber: KB2 Hap186; recordedBy: Angelika Brandt | Saskia Brix | Nils Brenke | Magdalena Blazewicz | Olga Golovan | Nele Heitland | Anna Jazdzewska | Karen Jeskulke | Gennady Kamenev | Anna Lavrenteva | Marina Malyutina | Torben Riehl; individualCount: 1; sex: indet.; lifeStage: manca; preparations: EtOH 96%; associatedSequences: https://www.ncbi.nlm.nih.gov/nuccore/OM782637.1; occurrenceID: CE077CCA-9C32-5800-B16B-98EC415E6794; **Taxon:** scientificName: *Mastigoniscusminimus* Wenz, Knauber & Riehl; kingdom: Animalia; phylum: Arthropoda; class: Malacostraca; order: Isopoda; family: Haploniscidae; genus: Mastigoniscus; specificEpithet: *minimus*; taxonRank: species; scientificNameAuthorship: Wenz, Knauber & Riehl; nomenclaturalCode: ICZN; **Location:** waterBody: Northwest Pacific Ocean; locality: Kuril-Kamchatka Trench, hadal depths of the Trench, KuramBio II expedition, station SO250-040; verbatimDepth: 7081 m; decimalLatitude: 45.66; decimalLongitude: 152.95; **Identification:** identificationID: KB2 Hap186; identifiedBy: Christian Wenz; dateIdentified: 2022; **Event:** samplingProtocol: Epibenthic Sledge; eventDate: 29/08/2016; habitat: deep-sea sediment; fieldNumber: SO250-040; **Record Level:** institutionCode: SMF; basisOfRecord: PreservedSpecimen
- **Type status:** Other material. **Occurrence:** catalogNumber: SMF 56474; recordNumber: KB2 Hap192; recordedBy: Angelika Brandt | Saskia Brix | Nils Brenke | Magdalena Blazewicz | Olga Golovan | Nele Heitland | Anna Jazdzewska | Karen Jeskulke | Gennady Kamenev | Anna Lavrenteva | Marina Malyutina | Torben Riehl; individualCount: 1; sex: male; lifeStage: juvenile; preparations: EtOH 96%; associatedSequences: https://www.ncbi.nlm.nih.gov/nuccore/OM782628.1; occurrenceID: 0B657B8D-6E83-525E-8E29-09E90D9CEF18; **Taxon:** scientificName: *Mastigoniscusminimus* Wenz, Knauber & Riehl; kingdom: Animalia; phylum: Arthropoda; class: Malacostraca; order: Isopoda; family: Haploniscidae; genus: Mastigoniscus; specificEpithet: *minimus*; taxonRank: species; scientificNameAuthorship: Wenz, Knauber & Riehl; nomenclaturalCode: ICZN; **Location:** waterBody: Northwest Pacific Ocean; locality: Kuril-Kamchatka Trench, abyssal plains southeast of the Trench, KuramBio II expedition, station SO250-008; verbatimDepth: 5136 m; decimalLatitude: 43.82; decimalLongitude: 151.77; **Identification:** identificationID: KB2 Hap192; identifiedBy: Christian Wenz; dateIdentified: 2022; **Event:** samplingProtocol: Epibenthic Sledge; eventDate: 19/08/2016; habitat: deep-sea sediment; fieldNumber: SO250-008; **Record Level:** institutionCode: SMF; basisOfRecord: PreservedSpecimen
- **Type status:** Other material. **Occurrence:** catalogNumber: SMF 56476; recordNumber: KB2 Hap194; recordedBy: Angelika Brandt | Saskia Brix | Nils Brenke | Magdalena Blazewicz | Olga Golovan | Nele Heitland | Anna Jazdzewska | Karen Jeskulke | Gennady Kamenev | Anna Lavrenteva | Marina Malyutina | Torben Riehl; individualCount: 1; sex: male; lifeStage: adult; preparations: EtOH 96%; associatedSequences: https://www.ncbi.nlm.nih.gov/nuccore/OM782633.1; occurrenceID: FDC78104-8645-5F3E-B716-B9DD006ABBE8; **Taxon:** scientificName: *Mastigoniscusminimus* Wenz, Knauber & Riehl; kingdom: Animalia; phylum: Arthropoda; class: Malacostraca; order: Isopoda; family: Haploniscidae; genus: Mastigoniscus; specificEpithet: *minimus*; taxonRank: species; scientificNameAuthorship: Wenz, Knauber & Riehl; nomenclaturalCode: ICZN; **Location:** waterBody: Northwest Pacific Ocean; locality: Kuril-Kamchatka Trench, abyssal plains southeast of the Trench, KuramBio II expedition, station SO250-008; verbatimDepth: 5136 m; decimalLatitude: 43.82; decimalLongitude: 151.77; **Identification:** identificationID: KB2 Hap194; identifiedBy: Christian Wenz; dateIdentified: 2022; **Event:** samplingProtocol: Epibenthic Sledge; eventDate: 19/08/2016; habitat: deep-sea sediment; fieldNumber: SO250-008; **Record Level:** institutionCode: SMF; basisOfRecord: PreservedSpecimen
- **Type status:** Other material. **Occurrence:** catalogNumber: SMF 56478; recordNumber: KB2 Hap196; recordedBy: Angelika Brandt | Saskia Brix | Nils Brenke | Magdalena Blazewicz | Olga Golovan | Nele Heitland | Anna Jazdzewska | Karen Jeskulke | Gennady Kamenev | Anna Lavrenteva | Marina Malyutina | Torben Riehl; individualCount: 1; sex: male; lifeStage: adult; preparations: EtOH 96%; associatedSequences: https://www.ncbi.nlm.nih.gov/nuccore/OM782629.1; occurrenceID: 975C0850-0B58-5D19-8BB9-ED01A9B10DE7; **Taxon:** scientificName: *Mastigoniscusminimus* Wenz, Knauber & Riehl; kingdom: Animalia; phylum: Arthropoda; class: Malacostraca; order: Isopoda; family: Haploniscidae; genus: Mastigoniscus; specificEpithet: *minimus*; taxonRank: species; scientificNameAuthorship: Wenz, Knauber & Riehl; nomenclaturalCode: ICZN; **Location:** waterBody: Northwest Pacific Ocean; locality: Kuril-Kamchatka Trench, abyssal plains southeast of the Trench, KuramBio II expedition, station SO250-006; verbatimDepth: 5146 m; decimalLatitude: 43.82; decimalLongitude: 151.76; **Identification:** identificationID: KB2 Hap196; identifiedBy: Christian Wenz; dateIdentified: 2022; **Event:** samplingProtocol: Box Corer; eventDate: 18/08/2016; habitat: deep-sea sediment; fieldNumber: SO250-006; **Record Level:** institutionCode: SMF; basisOfRecord: PreservedSpecimen

#### Description

**Type material remarks**: The adult male holotype (SMF 56475), despite being less intact than the adult male paratype (SMF 56301), was selected as such, based on the availability of a molecular barcode, which the male paratype lacks. When compared to known congeners, the pleopod 1 shape of the type material at hand presumably represents an adult male, but no terminal male stage. The pleopod 1 morphology is, therefore, most likely not fully developed and, thus, not included in the species diagnosis. As the holotype lacks the distal articles of antenna 2 beyond article 5, all antenna 2 characters have been scored based on the male paratype.

**Description of the adult male holotype (SMF 56475)**

**Figure 21.**
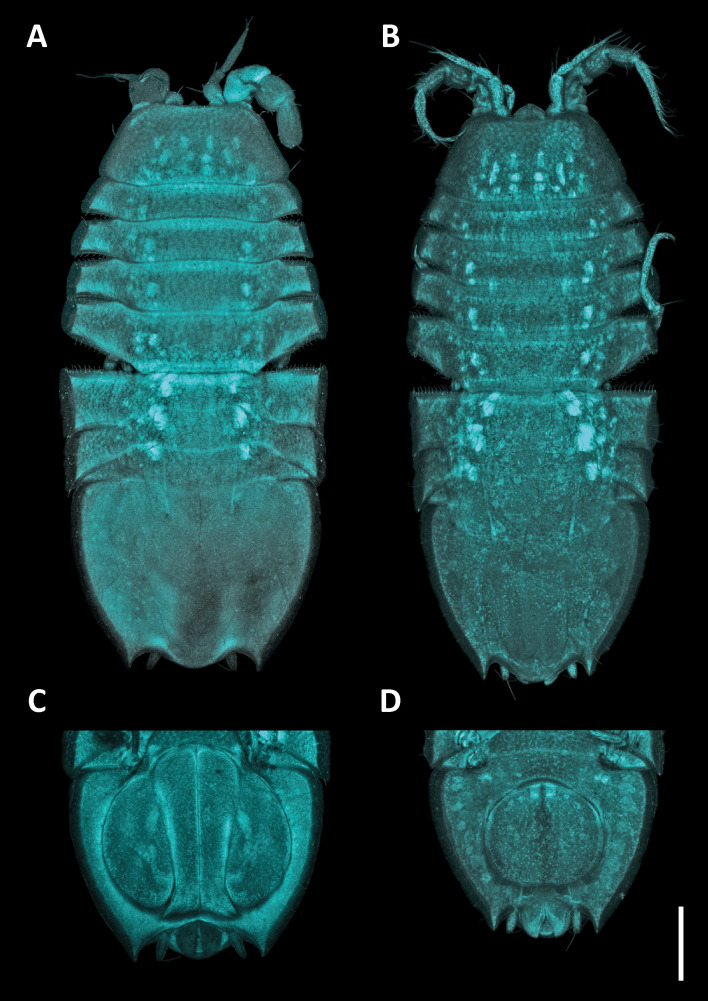
*Mastigoniscusminimus* Wenz, Knauber & Riehl, **sp. nov. A.** Male holotype SMF 56475, cLSM images of habitus, dorsal view; **B.** Female paratype SMF 56477, habitus, dorsal view; **C.** Male holotype SMF 56475, pleotelson, ventral view; **D.** Female paratype SMF 56477, pleotelson, ventral view. Scale bar: 0.2 mm.

**Figure 22.**
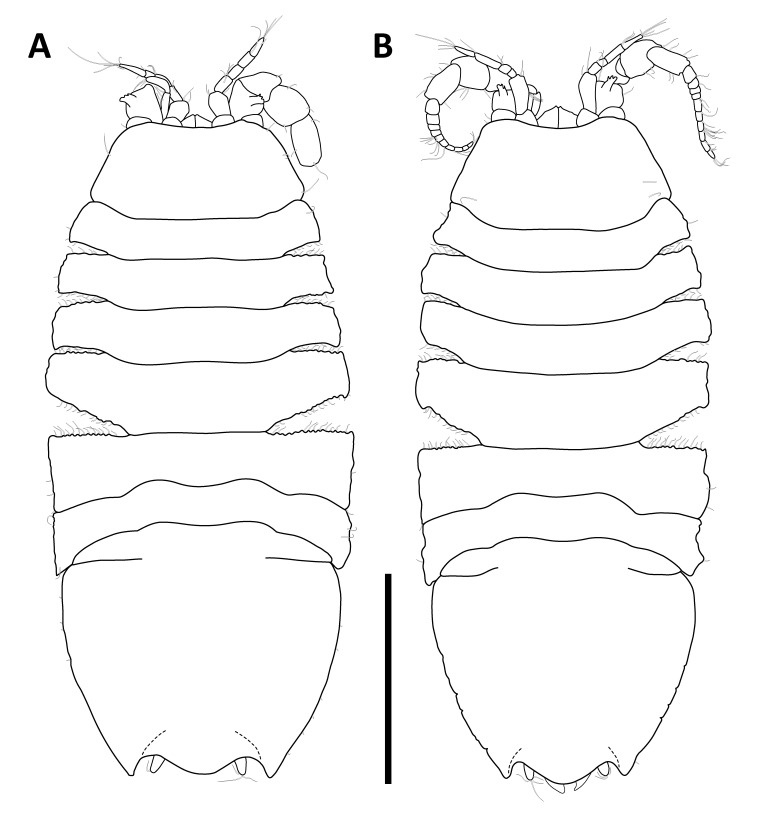
*Mastigoniscusminimus* Wenz, Knauber & Riehl, **sp. nov. A.** Male holotype SMF 56475, habitus, dorsal view; **B.** Female paratype SMF 56477, habitus, dorsal view. Scale bar: 0.5 mm.

**Body** (Fig. 21A and Fig. 22A) length 1.6 mm, 2.1 width; oval, dorsoventrally elliptical. Anterior body length (Ceph + Prn1–4) 0.90 posterior body length (Prn5–Plt). Lateral margin continuous; delicately serrated, setose from Prn1 posterolateral margin through to Prn5 anterolateral margin. Tergite surfaces tuberculate, ornamentation evenly distributed; convex cross-section of tergites broken by slight, uneven elevations at the muscle attachment points of pereopods, elevated areas without ornamentation. Non-conglobating.

**Ceph** (Fig. 21A and Fig. 22A) length 0.43 width, 0.15 body length, width 0.70 body width; trapezoidal, tergite surface tuberculate; anterolateral angles not projecting, rounded; frontal margin concave, width 0.42 Ceph width.

**Prn4** (Fig. 21A and Fig. 22A) posterolateral angle with minute, acute projection, lateral margin length 0.61 Prn5 lateral margin length. **Prn5** (Fig. 21A and Fig. 22A) anterolateral angle with minute, acute projection. **Prn7** (Fig. 21A and Fig. 22A) cone-shaped, shorter and narrower than Prn6; tergite medially conjoint with Plt, segment borders not expressed.

**Plt** (Fig. 21A and Fig. 22A) length 0.33 body length; anteriorly rectangular, caudally tapering to an obtuse point; posterior margin with convex apex, laterally concave; tergite surface smooth; posterolateral processes minute, less than 0.10 Plt length, straight, tapering to an acute tip, orientated posteriorly; width at posterolateral processes 0.50 Plt width.

**Figure 23.**
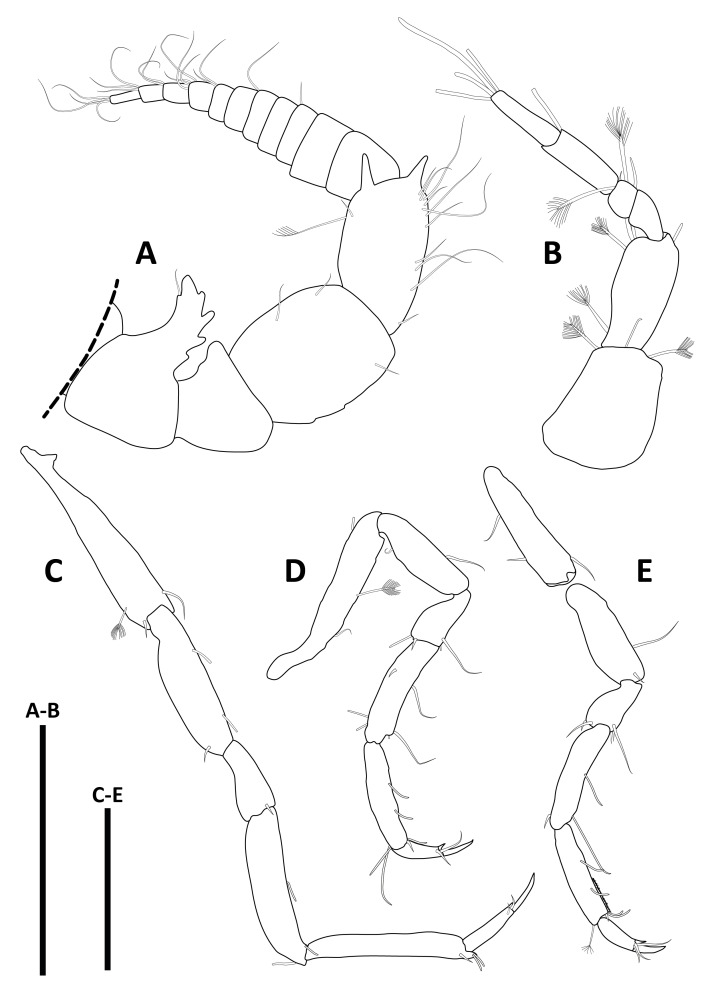
*Mastigoniscusminimus* Wenz, Knauber & Riehl, **sp. nov.** Male paratype SMF 56301. **A.** Antenna 2, lateral view. Male holotype SMF 56475; **B.** Antenna 1, lateral view; **C.** Pereopod 7; **D.** Pereopod 2. Female paratype SMF 56477; **E.** Pereopod 1. Scale bars: 0.2 mm.

**A1** (Fig. 23B) length 0.28 body length; flagellum with 3 art. **A2** (Fig. 23A) broken in the holotype, lacking the A2 art beyond art5. Adult male paratype (SMF 56301, body length 1.6 mm, same as holotype) with A2 length 0.44 body length; art3 length subsimilar width, dorsal projection hook-shaped, orientated posteriorly and dorsally, apically and anteriorly serrated, subapically with single seta, length 0.78 art3 length; art6 with two short, acute distal projections, length 0.21 art6 length; flagellum with 11 art.

**Figure 24.**
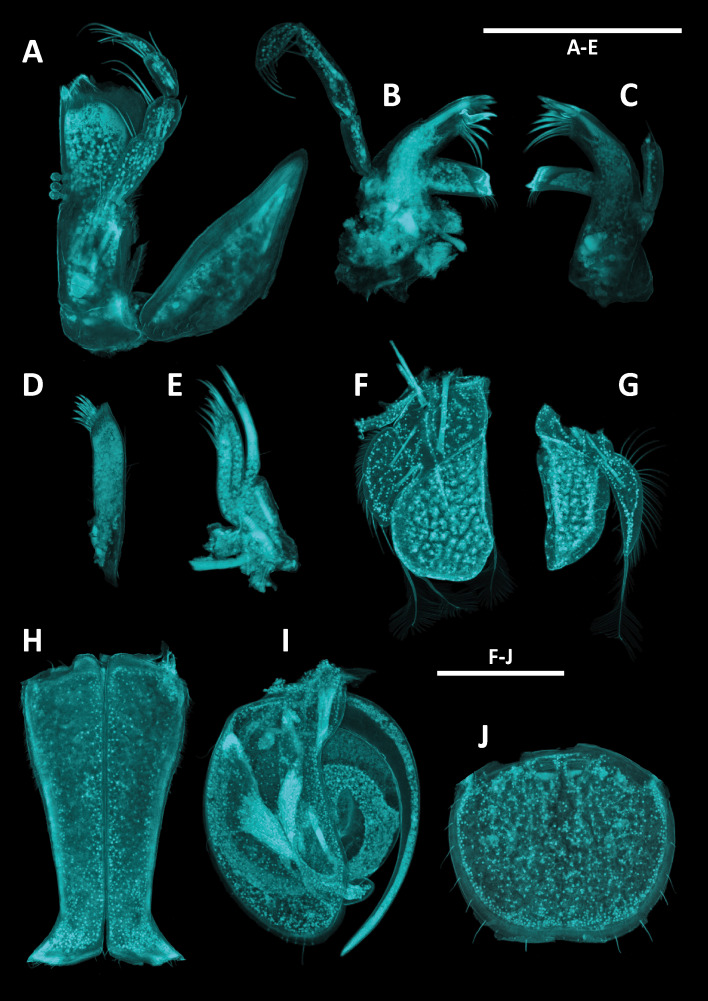
*Mastigoniscusminimus* Wenz, Knauber & Riehl, **sp. nov.** cLSM images of male holotype SMF 56475. **A.** Maxilliped; **B.** Left mandible; **C.** Right mandible; **D.** Maxilla 1; **E.** Maxilla 2; **F.** Pleopod 3; **G.** Pleopod 4; **H.** Pleopod 1; **I.** Pleopod 2. Female paratype SMF 56477. **J.** Operculum. Scale bars: 0.2 mm.

**Md** (Fig. 24B and C) incisors with five cusps each, left Md *lacinia mobilis* with 3 cusps. **Mxp** (Fig. 24A) with 3 coupling hooks.

**P1** (Fig. 23E) propodus dorsal margin smooth, with multiple groups of short setae and solitary elongated setae; P2 length 0.51 body length; P3 length 0.55 body length; P4 length 0.56 body length; P7 length 0.68 body length; P lengths gradually increasing posteriorly.

**Plp1** (Fig. 24H) proximal half subrectangular, distal half projecting caudolaterally; lateral lobes indistinct, fused with medial lobes; medial lobes triangular, separated at apex by narrow gap. **Plp2** (Fig. 24I) protopod semi-circular, distal margin with continuous row of simple setae, lateral margin with 1–3 short simple setae; endopod stylet distinctly longer than protopod. **Plp4** (Fig. 24G) exopod length 0.76 endopod length. **Urp** (Fig. 21A, C and Fig. 22A) cylindrical, short, projecting caudally as far as posterior Plt apex; socket position recessed in sternal fold laterally to anal valve.

**Description of the adult female paratype (SMF 56477; where different from male)**

**Body** (Fig. 21B and Fig. 22B) length 1.6 mm, 2.3 body width. Anterior and posterior body subsimilar in length. **Ceph** (Fig. 21B and Fig. 22B) length 0.49 width, 0.16 body length, width 0.76 body width; frontal margin width 0.45 Ceph width. **Prn4** lateral margin length 0.61 Prn5 lateral margin length. **Plt** length 0.32 body length; width at posterolateral processes 0.45 Plt width.

**A1** length 0.25 body length. **A2** length 0.42 body length; art3 dorsal projection length 0.60 art3 length; flagellum with 10 art. **P1** length 0.48 body length, P5 length 0.58 body length. **Op** length 0.59 Plt length; shape broad-oval, wider than long, surface smooth; distal and lateral margins with numerous, evenly distributed simple setae.

#### Diagnosis

Similar to *Mastigoniscuslatus* (Birstein, 1971), but differing in the following characters: body size up to 1.6 mm (2.1–2.2 mm in *M.latus*), dorsoventrally elliptical (dorsomedially vaulted, laterally flattened). Pleotelson anteriorly rectangular, caudally tapering to obtuse point (rounded). Antenna 2 article 3 dorsal projection curved (straight); article 6 with two short, acute distal projections (without distal projections).

**Molecular Diagnosis**: This species can be distinguished from *Mastigoniscuslatus* (Birstein, 1971), the sole other congeneric species in the KKT region, on the basis of the following combination of mtDNA 16S rRNA gene nucleotide substitutions: G (31), C (35), T (52), C (60), C (62), G (66), G (67), A (68), C (81), G (98), A (102), T (104), A (107), G (108), G (109), G (110), G (140), C (142), G (144), C (145), G (146), C (147), G (151), A (152), A (153), G (154), A (156), G (157), G (163), A (164), C (168), G (178), C (181), C (185), G (191), T (207), C (214), A (220), G (223), A (226), A (229), A (234), G (235), C (241), A (246), C (247), A (249), T (259), T (260), T (268), T (273) and G (448). The following combination of mtDNA COI rRNA gene nucleotide substitutions allow for further distinction: A (85), A (136), A (148), A (169), A (172), A (173), A (181), A (211), A (244), C (106), C (109), C (115), C (118), C (121), C (157), C (160), C (175), C (181), C (184), C (211), C (253), C (304), C (328), C (337), C (340), C (358), C (376), C (379), C (388), C (412), C (424), C (433), C (436), C (457), C (463), C (472), G (241), G (280), G (283), G (184), T (316), T (322), T (217) and T (238).

#### Etymology

The Latin adjective *minimus* ("smallest") refers to the small body size of this species in comparison to its known congeners.

#### Distribution

Northwest Pacific Ocean, Kuril-Kamchatka Trench and adjacent abyssal regions to the southeast, 5136 – 7081 m.

#### Taxon discussion

*Mastigoniscusminimus* Wenz, Knauber & Riehl, **sp. nov.** represents the third species known of this genus from the Northwest Pacific, alongside *M.microcephalus* (Gamó, 1989) and *M.latus* (Birstein, 1971). *Mastigoniscusminimus* reportedly occurs in sympatry with *M.latus* in the Kuril-Kamchatka Trench. All three of these Northwest Pacific species have a broad and relatively short habitus with a broadly rounded pleotelson and small posterolateral processes in common. Their shared morphology and geographical distribution set them apart from the remaining species in the genus, indicating a sub-group within the genus. With body lengths of up to 1.6 mm, *M.minimus* Wenz, Knauber & Riehl, **sp. nov.** represents the smallest *Mastigoniscus* species known yet, as adult specimens of most species usually reach body lengths beyond 2 mm.

When comparing *Mastigoniscusminimus* Wenz, Knauber & Riehl, **sp. nov.** to the original description and new material of *Mastigoniscuslatus* (Birstein, 1971), it became evident that the specimen depicted in the original description represents an adult male, but not the terminal male stage. However, a terminal male specimen (SMF 56420) was present amongst our newly-collected material (see Knauber et al. (2022)). It differs from the specimen depicted by Birstein (1971) – amongst other characters – in a wider, less trapezoidal shape of the cephalothorax, the posterolateral pleotelson processes less protruding and the distal half of the first pleopods resembling the habitus of other known congeners. A thorough re-description of *Mastigoniscuslatus* seems therefore necessary.

#### Notes

##### Methods

This study is based on collection material and molecular sequence information that has been previously published elsewhere (see Knauber et al. (2022) for details). Specimens are deposited at the Senckenberg Museum in Frankfurt, Germany. Associated (meta-)data are stored in the Barcode of Life Data System (BOLD System, Ratnasingham and Hebert (2007)) available at  https://doi.org/10.5883/DS-NWPHA22, GenBank (Benson et al. 2012) accession numbers OM782626 to OM782641; Ocean Biogeographic Information System (OBIS, Grassle 2000) available at https://ipt.iobis.org/obis-deepsea/resource?r=deep-sea_haploniscid_isopods; and Zenodo (European Organization For Nuclear Research and OpenAIRE 2013) available at  https://doi.org/10.5281/zenodo.6553796.

Material was collected during the KuramBio II (Kuril Kamchatka Biodiversity Studies II, Brandt (2016)) campaign on board RV *Sonne* to the Northwest Pacific Ocean. Box corer (BC; Hessler and Jumars (1974)), Agassiz trawl (AGT; Agassiz (1880)) and epibenthic sledge (EBS; Brenke (2005), Brandt et al. (2013)) were used. Samples were processed on board following Riehl et al. (2014b).

Using a Leica M60 stereomicroscope, an adult male was selected as holotype and an adult female as paratype following recent examples (Brökeland and Raupach 2008, Brökeland 2010, Brökeland and Svavarsson 2017). For drawings, a Leica DM 2500 LED with camera lucida was used. Damage-free transfer of specimens from ethanol to glycerine followed Knauber et al. (2022). Temporary slides were prepared following Wilson (2008). Pencil drawings were digitally traced with Adobe Illustrator 27.2 (Coleman 2003).

While in glycerine, specimens were stained using Congo Red dissolved in 70% denatured EtOH following Michels and Büntzow (2010) for Confocal Laser Scanning Microscopy (CLSM) using a Leica DM2500 with a Leica TCS SPE II and LEICA LAS X 3.5.5.19976. Post-production of CLSM-scans was carried out in Adobe Photoshop 24.1.1 and Adobe Illustrator 27.2. Measurements followed the standards of Hessler (1970).

In the description, all appendages’ article-length ratios are given in proximal to distal order, excluding setae. Many ratios are used for descriptions in this paper. To avoid multiple repetition of the word ‘times,’ these are reported as a multiplier of an object of a telegraphic phrase to indicate the size of the subject of the phrase. For example, ‘endopod length 2.2 width’ means ‘the length of the endopod is 2.2 times its width.’ This example is mathematically equivalent to the equation ‘L = 2.2W’. Dependent object clauses, separated off by a comma, do not repeat the subject. Descriptions of pereopod setae are provided in proximal to distal and lateral to medial order of description.

Morphological terminology was based on Brökeland and Svavarsson (2017) with modifications. Setae were named after Riehl and Brandt (2010). The molecular diagnosis was prepared using the online-tool DeSigNate (Hütter et al. 2020), only focusing on base positions with a discriminative power of 1.0.

### Macrostylis papandreas

Johannsen, Riehl & Brandt sp. nov.

67CA804B-3FFE-53E1-B1D7-7C6606216F30

46698C36-4C0D-46A6-9566-07A152D08A52

"MLpap": Riehl et al. (2018)
"*Macrostylis* sp. MLpap": Bober et al. (2018)

#### Materials

- **Type status:** Holotype. **Occurrence:** catalogNumber: ZMH K-45148; recordNumber: VTMac145; individualCount: 1; sex: female; lifeStage: adult; preparations: whole animal (ETOH); previousIdentifications: *Macrostylis* sp. Mlpap | MOTU Mlpap; associatedReferences: Riehl T, Lins L, Brandt A (2018) The effects of depth, distance, and the Mid-Atlantic Ridge on genetic differentiation of abyssal and hadal isopods (Macrostylidae). Deep Sea Research Part II: Topical Studies in Oceanography 148: 74–90. https://doi.org/10.1016/j.dsr2.2017.10.005 | Bober S, Brix S, Riehl T, Schwentner M, Brandt A (2018) Does the Mid-Atlantic Ridge affect the distribution of abyssal benthic crustaceans across the Atlantic Ocean? Deep-Sea Research Part II: Topical Studies in Oceanography 148: 91–104. https://doi.org/10.1016/j.dsr2.2018.02.011; associatedSequences: https://www.ncbi.nlm.nih.gov/nuccore/LT909298.1; occurrenceID: D1ADE97E-79B9-5F53-8F63-60B077430871; **Taxon:** scientificName: *Macrostylispapandreas* Johannsen, Riehl & Brandt; kingdom: Animalia; phylum: Arthropoda; class: Malacostraca; order: Isopoda; family: Macrostylidae; genus: Macrostylis; specificEpithet: *papandreas*; scientificNameAuthorship: Johannsen, Riehl & Brandt; nomenclaturalCode: ICZN; **Location:** higherGeography: Atlantic Ocean; locality: Eastern Vema Fracture Zone, Vema-TRANSIT station SO237-6-11; verbatimDepth: 5082 m; verbatimLatitude: 10°21.82' N; verbatimLongitude: 36°55.06' W; decimalLatitude: 10.36367; decimalLongitude: 36.91767; **Identification:** identifiedBy: Torben Riehl, Simon Bober; **Event:** samplingProtocol: Benthos trawl, Camera-Epibenthic Sledge, sieved through 0.3 mm mesh | Riehl T, Brenke N, Brix S, Driskell A, Kaiser S, Brandt A (2014) Field and laboratory methods for DNA studies on deep-sea isopod crustaceans. Polish Polar Research 35: 205–226. https://doi.org/10.2478/popore−2014−0018 | Devey CW (2015) RV SONNE Fahrtbericht / Cruise Report SO237 Vema-TRANSIT. Geomar Report 23: 130. https://doi.org/10.3289/GEOMAR_REP_NS_23_2019; eventDate: 02/01/2015; habitat: abyssal sediment; fieldNumber: SO237-6-7; **Record Level:** institutionCode: ZMH; collectionCode: K; basisOfRecord: PreservedSpecimen
- **Type status:** Paratype. **Occurrence:** catalogNumber: ZMH K-45165; recordNumber: VTMac134; individualCount: 1; sex: male; lifeStage: adult; preparations: whole animal (ETOH); previousIdentifications: *Macrostylis* sp. Mlpap | MOTU Mlpap; associatedReferences: Riehl T, Lins L, Brandt A (2018) The effects of depth, distance, and the Mid-Atlantic Ridge on genetic differentiation of abyssal and hadal isopods (Macrostylidae). Deep Sea Research Part II: Topical Studies in Oceanography 148: 74–90. https://doi.org/10.1016/j.dsr2.2017.10.005 | Bober S, Brix S, Riehl T, Schwentner M, Brandt A (2018) Does the Mid-Atlantic Ridge affect the distribution of abyssal benthic crustaceans across the Atlantic Ocean? Deep-Sea Research Part II: Topical Studies in Oceanography 148: 91–104. https://doi.org/10.1016/j.dsr2.2018.02.007; associatedSequences: https://www.ncbi.nlm.nih.gov/nuccore/LT909287.1 | https://www.ncbi.nlm.nih.gov/nuccore/LT960448.1; occurrenceID: F604E4E7-CF85-59F3-B18D-7F2AAFC3FF3D; **Taxon:** scientificName: *Macrostylispapandreas* Johannsen, Riehl & Brandt; kingdom: Animalia; phylum: Arthropoda; class: Malacostraca; order: Isopoda; family: Macrostylidae; genus: Macrostylis; specificEpithet: *papandreas*; scientificNameAuthorship: Johannsen, Riehl & Brandt; nomenclaturalCode: ICZN; **Location:** higherGeography: Atlantic Ocean; locality: Eastern Vema Fracture Zone, Vema-TRANSIT station SO237-6-7; verbatimDepth: 5082 m; verbatimLatitude: 10°21.82' N; verbatimLongitude: 36°55.06' W; decimalLatitude: 10.36367; decimalLongitude: 36.91767; **Identification:** identifiedBy: Torben Riehl, Simon Bober; **Event:** samplingProtocol: Benthos trawl, Camera-Epibenthic Sledge, sieved through 0.3 mm mesh | Riehl T, Brenke N, Brix S, Driskell A, Kaiser S, Brandt A (2014) Field and laboratory methods for DNA studies on deep-sea isopod crustaceans. Polish Polar Research 35: 205–226. https://doi.org/10.2478/popore−2014−0018 | Devey CW (2015) RV SONNE Fahrtbericht / Cruise Report SO237 Vema-TRANSIT. Geomar Report 23: 130. https://doi.org/10.3289/GEOMAR_REP_NS_23_2015; eventDate: 02/01/2015; habitat: abyssal sediment; fieldNumber: SO237-6-7; **Record Level:** institutionCode: ZMH; collectionCode: K; basisOfRecord: PreservedSpecimen
- **Type status:** Paratype. **Occurrence:** catalogNumber: ZMH K-45166; recordNumber: VTMac137; individualCount: 1; sex: male; lifeStage: adult; preparations: whole animal (ETOH); previousIdentifications: *Macrostylis* sp. Mlpap | MOTU Mlpap; associatedReferences: Riehl T, Lins L, Brandt A (2018) The effects of depth, distance, and the Mid-Atlantic Ridge on genetic differentiation of abyssal and hadal isopods (Macrostylidae). Deep Sea Research Part II: Topical Studies in Oceanography 148: 74–90. https://doi.org/10.1016/j.dsr2.2017.10.005 | Bober S, Brix S, Riehl T, Schwentner M, Brandt A (2018) Does the Mid-Atlantic Ridge affect the distribution of abyssal benthic crustaceans across the Atlantic Ocean? Deep-Sea Research Part II: Topical Studies in Oceanography 148: 91–104. https://doi.org/10.1016/j.dsr2.2018.02.008; associatedSequences: https://www.ncbi.nlm.nih.gov/nuccore/LT909290.1 | https://www.ncbi.nlm.nih.gov/nuccore/LT960449.1; occurrenceID: 449B5E29-8A25-531E-A8AD-4C7FC9573331; **Taxon:** scientificName: *Macrostylispapandreas* Johannsen, Riehl & Brandt; kingdom: Animalia; phylum: Arthropoda; class: Malacostraca; order: Isopoda; family: Macrostylidae; genus: Macrostylis; specificEpithet: *papandreas*; scientificNameAuthorship: Johannsen, Riehl & Brandt; nomenclaturalCode: ICZN; **Location:** higherGeography: Atlantic Ocean; locality: Eastern Vema Fracture Zone, Vema-TRANSIT station SO237-6-8; verbatimDepth: 5082 m; verbatimLatitude: 10°21.82' N; verbatimLongitude: 36°55.06' W; decimalLatitude: 10.36367; decimalLongitude: 36.91767; **Identification:** identifiedBy: Torben Riehl, Simon Bober; **Event:** samplingProtocol: Benthos trawl, Camera-Epibenthic Sledge, sieved through 0.3 mm mesh | Riehl T, Brenke N, Brix S, Driskell A, Kaiser S, Brandt A (2014) Field and laboratory methods for DNA studies on deep-sea isopod crustaceans. Polish Polar Research 35: 205–226. https://doi.org/10.2478/popore−2014−0018 | Devey CW (2015) RV SONNE Fahrtbericht / Cruise Report SO237 Vema-TRANSIT. Geomar Report 23: 130. https://doi.org/10.3289/GEOMAR_REP_NS_23_2016; eventDate: 02/01/2015; habitat: abyssal sediment; fieldNumber: SO237-6-7; **Record Level:** institutionCode: ZMH; collectionCode: K; basisOfRecord: PreservedSpecimen
- **Type status:** Paratype. **Occurrence:** catalogNumber: ZMH K-45146; recordNumber: VTMac140; individualCount: 1; sex: female; lifeStage: adult; preparations: whole animal (ETOH); previousIdentifications: *Macrostylis* sp. Mlpap | MOTU Mlpap; associatedReferences: Riehl T, Lins L, Brandt A (2018) The effects of depth, distance, and the Mid-Atlantic Ridge on genetic differentiation of abyssal and hadal isopods (Macrostylidae). Deep Sea Research Part II: Topical Studies in Oceanography 148: 74–90. https://doi.org/10.1016/j.dsr2.2017.10.005 | Bober S, Brix S, Riehl T, Schwentner M, Brandt A (2018) Does the Mid-Atlantic Ridge affect the distribution of abyssal benthic crustaceans across the Atlantic Ocean? Deep-Sea Research Part II: Topical Studies in Oceanography 148: 91–104. https://doi.org/10.1016/j.dsr2.2018.02.009; associatedSequences: https://www.ncbi.nlm.nih.gov/nuccore/LT909293.1 | https://www.ncbi.nlm.nih.gov/nuccore/LT960450.1; occurrenceID: 17C01CFF-068C-52A6-8BDA-3FC81DF853BB; **Taxon:** scientificName: *Macrostylispapandreas* Johannsen, Riehl & Brandt; kingdom: Animalia; phylum: Arthropoda; class: Malacostraca; order: Isopoda; family: Macrostylidae; genus: Macrostylis; specificEpithet: *papandreas*; scientificNameAuthorship: Johannsen, Riehl & Brandt; nomenclaturalCode: ICZN; **Location:** higherGeography: Atlantic Ocean; locality: Eastern Vema Fracture Zone, Vema-TRANSIT station SO237-6-9; verbatimDepth: 5082 m; verbatimLatitude: 10°21.82' N; verbatimLongitude: 36°55.06' W; decimalLatitude: 10.36367; decimalLongitude: 36.91767; **Identification:** identifiedBy: Torben Riehl, Simon Bober; **Event:** samplingProtocol: Benthos trawl, Camera-Epibenthic Sledge, sieved through 0.3 mm mesh | Riehl T, Brenke N, Brix S, Driskell A, Kaiser S, Brandt A (2014) Field and laboratory methods for DNA studies on deep-sea isopod crustaceans. Polish Polar Research 35: 205–226. https://doi.org/10.2478/popore−2014−0018 | Devey CW (2015) RV SONNE Fahrtbericht / Cruise Report SO237 Vema-TRANSIT. Geomar Report 23: 130. https://doi.org/10.3289/GEOMAR_REP_NS_23_2017; eventDate: 02/01/2015; habitat: abyssal sediment; fieldNumber: SO237-6-7; **Record Level:** institutionCode: ZMH; collectionCode: K; basisOfRecord: PreservedSpecimen
- **Type status:** Paratype. **Occurrence:** catalogNumber: ZMH K-45147; recordNumber: VTMac141; individualCount: 1; sex: female; lifeStage: adult; preparations: whole animal (ETOH); previousIdentifications: *Macrostylis* sp. Mlpap | MOTU Mlpap; associatedReferences: Riehl T, Lins L, Brandt A (2018) The effects of depth, distance, and the Mid-Atlantic Ridge on genetic differentiation of abyssal and hadal isopods (Macrostylidae). Deep Sea Research Part II: Topical Studies in Oceanography 148: 74–90. https://doi.org/10.1016/j.dsr2.2017.10.005 | Bober S, Brix S, Riehl T, Schwentner M, Brandt A (2018) Does the Mid-Atlantic Ridge affect the distribution of abyssal benthic crustaceans across the Atlantic Ocean? Deep-Sea Research Part II: Topical Studies in Oceanography 148: 91–104. https://doi.org/10.1016/j.dsr2.2018.02.010; associatedSequences: https://www.ncbi.nlm.nih.gov/nuccore/LT909294.1 | https://www.ncbi.nlm.nih.gov/nuccore/LT960451.1; occurrenceID: BC9D5A22-9156-59CE-8829-15BC66E0A2D1; **Taxon:** scientificName: *Macrostylispapandreas* Johannsen, Riehl & Brandt; kingdom: Animalia; phylum: Arthropoda; class: Malacostraca; order: Isopoda; family: Macrostylidae; genus: Macrostylis; specificEpithet: *papandreas*; scientificNameAuthorship: Johannsen, Riehl & Brandt; nomenclaturalCode: ICZN; **Location:** higherGeography: Atlantic Ocean; locality: Eastern Vema Fracture Zone, Vema-TRANSIT station SO237-6-10; verbatimDepth: 5082 m; verbatimLatitude: 10°21.82' N; verbatimLongitude: 36°55.06' W; decimalLatitude: 10.36367; decimalLongitude: 36.91767; **Identification:** identifiedBy: Torben Riehl, Simon Bober; **Event:** samplingProtocol: Benthos trawl, Camera-Epibenthic Sledge, sieved through 0.3 mm mesh | Riehl T, Brenke N, Brix S, Driskell A, Kaiser S, Brandt A (2014) Field and laboratory methods for DNA studies on deep-sea isopod crustaceans. Polish Polar Research 35: 205–226. https://doi.org/10.2478/popore−2014−0018 | Devey CW (2015) RV SONNE Fahrtbericht / Cruise Report SO237 Vema-TRANSIT. Geomar Report 23: 130. https://doi.org/10.3289/GEOMAR_REP_NS_23_2018; eventDate: 02/01/2015; habitat: abyssal sediment; fieldNumber: SO237-6-7; **Record Level:** institutionCode: ZMH; collectionCode: K; basisOfRecord: PreservedSpecimen
- **Type status:** Paratype. **Occurrence:** catalogNumber: ZMH K-45149; recordNumber: VTMac147; individualCount: 1; sex: female; lifeStage: adult; preparations: whole animal (ETOH); previousIdentifications: *Macrostylis* sp. Mlpap | MOTU Mlpap; associatedReferences: Riehl T, Lins L, Brandt A (2018) The effects of depth, distance, and the Mid-Atlantic Ridge on genetic differentiation of abyssal and hadal isopods (Macrostylidae). Deep Sea Research Part II: Topical Studies in Oceanography 148: 74–90. https://doi.org/10.1016/j.dsr2.2017.10.005 | Bober S, Brix S, Riehl T, Schwentner M, Brandt A (2018) Does the Mid-Atlantic Ridge affect the distribution of abyssal benthic crustaceans across the Atlantic Ocean? Deep-Sea Research Part II: Topical Studies in Oceanography 148: 91–104. https://doi.org/10.1016/j.dsr2.2018.02.012; associatedSequences: https://www.ncbi.nlm.nih.gov/nuccore/LT909299.1 | https://www.ncbi.nlm.nih.gov/nuccore/LT960452.1; occurrenceID: 8DFEE603-5718-52F4-BAB3-1FC538780D42; **Taxon:** scientificName: *Macrostylispapandreas* Johannsen, Riehl & Brandt; kingdom: Animalia; phylum: Arthropoda; class: Malacostraca; order: Isopoda; family: Macrostylidae; genus: Macrostylis; specificEpithet: *papandreas*; scientificNameAuthorship: Johannsen, Riehl & Brandt; nomenclaturalCode: ICZN; **Location:** higherGeography: Atlantic Ocean; locality: Eastern Vema Fracture Zone, Vema-TRANSIT station SO237-6-12; verbatimDepth: 5082 m; verbatimLatitude: 10°21.82' N; verbatimLongitude: 36°55.06' W; decimalLatitude: 10.36367; decimalLongitude: 36.91767; **Identification:** identifiedBy: Torben Riehl, Simon Bober; **Event:** samplingProtocol: Benthos trawl, Camera-Epibenthic Sledge, sieved through 0.3 mm mesh | Riehl T, Brenke N, Brix S, Driskell A, Kaiser S, Brandt A (2014) Field and laboratory methods for DNA studies on deep-sea isopod crustaceans. Polish Polar Research 35: 205–226. https://doi.org/10.2478/popore−2014−0018 | Devey CW (2015) RV SONNE Fahrtbericht / Cruise Report SO237 Vema-TRANSIT. Geomar Report 23: 130. https://doi.org/10.3289/GEOMAR_REP_NS_23_2020; eventDate: 02/01/2015; habitat: abyssal sediment; fieldNumber: SO237-6-7; **Record Level:** institutionCode: ZMH; collectionCode: K; basisOfRecord: PreservedSpecimen
- **Type status:** Paratype. **Occurrence:** catalogNumber: ZMH K-45150; recordNumber: VTMac148; individualCount: 1; sex: female; lifeStage: adult; preparations: whole animal (ETOH); previousIdentifications: *Macrostylis* sp. Mlpap | MOTU Mlpap; associatedReferences: Riehl T, Lins L, Brandt A (2018) The effects of depth, distance, and the Mid-Atlantic Ridge on genetic differentiation of abyssal and hadal isopods (Macrostylidae). Deep Sea Research Part II: Topical Studies in Oceanography 148: 74–90. https://doi.org/10.1016/j.dsr2.2017.10.005 | Bober S, Brix S, Riehl T, Schwentner M, Brandt A (2018) Does the Mid-Atlantic Ridge affect the distribution of abyssal benthic crustaceans across the Atlantic Ocean? Deep-Sea Research Part II: Topical Studies in Oceanography 148: 91–104. https://doi.org/10.1016/j.dsr2.2018.02.013; associatedSequences: https://www.ncbi.nlm.nih.gov/nuccore/LT909300.1 | https://www.ncbi.nlm.nih.gov/nuccore/LT960453.1; occurrenceID: DB0AB22C-2CB9-5ECC-B925-6ED6A940219C; **Taxon:** scientificName: *Macrostylispapandreas* Johannsen, Riehl & Brandt; kingdom: Animalia; phylum: Arthropoda; class: Malacostraca; order: Isopoda; family: Macrostylidae; genus: Macrostylis; specificEpithet: *papandreas*; scientificNameAuthorship: Johannsen, Riehl & Brandt; nomenclaturalCode: ICZN; **Location:** higherGeography: Atlantic Ocean; locality: Eastern Vema Fracture Zone, Vema-TRANSIT station SO237-6-13; verbatimDepth: 5082 m; verbatimLatitude: 10°21.82' N; verbatimLongitude: 36°55.06' W; decimalLatitude: 10.36367; decimalLongitude: 36.91767; **Identification:** identifiedBy: Torben Riehl, Simon Bober; **Event:** samplingProtocol: Benthos trawl, Camera-Epibenthic Sledge, sieved through 0.3 mm mesh | Riehl T, Brenke N, Brix S, Driskell A, Kaiser S, Brandt A (2014) Field and laboratory methods for DNA studies on deep-sea isopod crustaceans. Polish Polar Research 35: 205–226. https://doi.org/10.2478/popore−2014−0018 | Devey CW (2015) RV SONNE Fahrtbericht / Cruise Report SO237 Vema-TRANSIT. Geomar Report 23: 130. https://doi.org/10.3289/GEOMAR_REP_NS_23_2021; eventDate: 02/01/2015; habitat: abyssal sediment; fieldNumber: SO237-6-7; **Record Level:** institutionCode: ZMH; collectionCode: K; basisOfRecord: PreservedSpecimen
- **Type status:** Paratype. **Occurrence:** catalogNumber: ZMH K-45167; recordNumber: VTMac149; individualCount: 1; sex: male; lifeStage: adult; preparations: whole animal (ETOH); previousIdentifications: *Macrostylis* sp. Mlpap | MOTU Mlpap; associatedReferences: Riehl T, Lins L, Brandt A (2018) The effects of depth, distance, and the Mid-Atlantic Ridge on genetic differentiation of abyssal and hadal isopods (Macrostylidae). Deep Sea Research Part II: Topical Studies in Oceanography 148: 74–90. https://doi.org/10.1016/j.dsr2.2017.10.005 | Bober S, Brix S, Riehl T, Schwentner M, Brandt A (2018) Does the Mid-Atlantic Ridge affect the distribution of abyssal benthic crustaceans across the Atlantic Ocean? Deep-Sea Research Part II: Topical Studies in Oceanography 148: 91–104. https://doi.org/10.1016/j.dsr2.2018.02.014; associatedSequences: https://www.ncbi.nlm.nih.gov/nuccore/LT909301.1; occurrenceID: C200C08A-28F0-5F9D-9EEA-19E7CBADE32E; **Taxon:** scientificName: *Macrostylispapandreas* Johannsen, Riehl & Brandt; kingdom: Animalia; phylum: Arthropoda; class: Malacostraca; order: Isopoda; family: Macrostylidae; genus: Macrostylis; specificEpithet: *papandreas*; scientificNameAuthorship: Johannsen, Riehl & Brandt; nomenclaturalCode: ICZN; **Location:** higherGeography: Atlantic Ocean; locality: Eastern Vema Fracture Zone, Vema-TRANSIT station SO237-6-14; verbatimDepth: 5082 m; verbatimLatitude: 10°21.82' N; verbatimLongitude: 36°55.06' W; decimalLatitude: 10.36367; decimalLongitude: 36.91767; **Identification:** identifiedBy: Torben Riehl, Simon Bober; **Event:** samplingProtocol: Benthos trawl, Camera-Epibenthic Sledge, sieved through 0.3 mm mesh | Riehl T, Brenke N, Brix S, Driskell A, Kaiser S, Brandt A (2014) Field and laboratory methods for DNA studies on deep-sea isopod crustaceans. Polish Polar Research 35: 205–226. https://doi.org/10.2478/popore−2014−0018 | Devey CW (2015) RV SONNE Fahrtbericht / Cruise Report SO237 Vema-TRANSIT. Geomar Report 23: 130. https://doi.org/10.3289/GEOMAR_REP_NS_23_2022; eventDate: 02/01/2015; habitat: abyssal sediment; fieldNumber: SO237-6-7; **Record Level:** institutionCode: ZMH; collectionCode: K; basisOfRecord: PreservedSpecimen
- **Type status:** Paratype. **Occurrence:** catalogNumber: ZMH K-45151; recordNumber: VTMac150; individualCount: 1; sex: female; lifeStage: adult; preparations: whole animal (ETOH); previousIdentifications: *Macrostylis* sp. Mlpap | MOTU Mlpap; associatedReferences: Riehl T, Lins L, Brandt A (2018) The effects of depth, distance, and the Mid-Atlantic Ridge on genetic differentiation of abyssal and hadal isopods (Macrostylidae). Deep Sea Research Part II: Topical Studies in Oceanography 148: 74–90. https://doi.org/10.1016/j.dsr2.2017.10.005 | Bober S, Brix S, Riehl T, Schwentner M, Brandt A (2018) Does the Mid-Atlantic Ridge affect the distribution of abyssal benthic crustaceans across the Atlantic Ocean? Deep-Sea Research Part II: Topical Studies in Oceanography 148: 91–104. https://doi.org/10.1016/j.dsr2.2018.02.015; associatedSequences: https://www.ncbi.nlm.nih.gov/nuccore/LT909302.1; occurrenceID: CE232998-FB12-5D1D-AEDC-43835D29E575; **Taxon:** scientificName: *Macrostylispapandreas* Johannsen, Riehl & Brandt; kingdom: Animalia; phylum: Arthropoda; class: Malacostraca; order: Isopoda; family: Macrostylidae; genus: Macrostylis; specificEpithet: *papandreas*; scientificNameAuthorship: Johannsen, Riehl & Brandt; nomenclaturalCode: ICZN; **Location:** higherGeography: Atlantic Ocean; locality: Eastern Vema Fracture Zone, Vema-TRANSIT station SO237-6-15; verbatimDepth: 5082 m; verbatimLatitude: 10°21.82' N; verbatimLongitude: 36°55.06' W; decimalLatitude: 10.36367; decimalLongitude: 36.91767; **Identification:** identifiedBy: Torben Riehl, Simon Bober; **Event:** samplingProtocol: Benthos trawl, Camera-Epibenthic Sledge, sieved through 0.3 mm mesh | Riehl T, Brenke N, Brix S, Driskell A, Kaiser S, Brandt A (2014) Field and laboratory methods for DNA studies on deep-sea isopod crustaceans. Polish Polar Research 35: 205–226. https://doi.org/10.2478/popore−2014−0018 | Devey CW (2015) RV SONNE Fahrtbericht / Cruise Report SO237 Vema-TRANSIT. Geomar Report 23: 130. https://doi.org/10.3289/GEOMAR_REP_NS_23_2023; eventDate: 02/01/2015; habitat: abyssal sediment; fieldNumber: SO237-6-7; **Record Level:** institutionCode: ZMH; collectionCode: K; basisOfRecord: PreservedSpecimen
- **Type status:** Paratype. **Occurrence:** catalogNumber: ZMH K-45168; recordNumber: VTMac151; individualCount: 1; sex: male; lifeStage: adult; preparations: whole animal (ETOH); previousIdentifications: *Macrostylis* sp. Mlpap | MOTU Mlpap; associatedReferences: Riehl T, Lins L, Brandt A (2018) The effects of depth, distance, and the Mid-Atlantic Ridge on genetic differentiation of abyssal and hadal isopods (Macrostylidae). Deep Sea Research Part II: Topical Studies in Oceanography 148: 74–90. https://doi.org/10.1016/j.dsr2.2017.10.005 | Bober S, Brix S, Riehl T, Schwentner M, Brandt A (2018) Does the Mid-Atlantic Ridge affect the distribution of abyssal benthic crustaceans across the Atlantic Ocean? Deep-Sea Research Part II: Topical Studies in Oceanography 148: 91–104. https://doi.org/10.1016/j.dsr2.2018.02.016; associatedSequences: https://www.ncbi.nlm.nih.gov/nuccore/LT909303.1; occurrenceID: E0FFE2A7-97F0-5746-99DD-982F611659A4; **Taxon:** scientificName: *Macrostylispapandreas* Johannsen, Riehl & Brandt; kingdom: Animalia; phylum: Arthropoda; class: Malacostraca; order: Isopoda; family: Macrostylidae; genus: Macrostylis; specificEpithet: *papandreas*; scientificNameAuthorship: Johannsen, Riehl & Brandt; nomenclaturalCode: ICZN; **Location:** higherGeography: Atlantic Ocean; locality: Eastern Vema Fracture Zone, Vema-TRANSIT station SO237-6-16; verbatimDepth: 5082 m; verbatimLatitude: 10°21.82' N; verbatimLongitude: 36°55.06' W; decimalLatitude: 10.36367; decimalLongitude: 36.91767; **Identification:** identifiedBy: Torben Riehl, Simon Bober; **Event:** samplingProtocol: Benthos trawl, Camera-Epibenthic Sledge, sieved through 0.3 mm mesh | Riehl T, Brenke N, Brix S, Driskell A, Kaiser S, Brandt A (2014) Field and laboratory methods for DNA studies on deep-sea isopod crustaceans. Polish Polar Research 35: 205–226. https://doi.org/10.2478/popore−2014−0018 | Devey CW (2015) RV SONNE Fahrtbericht / Cruise Report SO237 Vema-TRANSIT. Geomar Report 23: 130. https://doi.org/10.3289/GEOMAR_REP_NS_23_2024; eventDate: 02/01/2015; habitat: abyssal sediment; fieldNumber: SO237-6-7; **Record Level:** institutionCode: ZMH; collectionCode: K; basisOfRecord: PreservedSpecimen
- **Type status:** Paratype. **Occurrence:** catalogNumber: ZMH K-45152; recordNumber: VTMac156; individualCount: 1; sex: female; lifeStage: adult; preparations: whole animal (ETOH); previousIdentifications: *Macrostylis* sp. Mlpap | MOTU Mlpap; associatedReferences: Riehl T, Lins L, Brandt A (2018) The effects of depth, distance, and the Mid-Atlantic Ridge on genetic differentiation of abyssal and hadal isopods (Macrostylidae). Deep Sea Research Part II: Topical Studies in Oceanography 148: 74–90. https://doi.org/10.1016/j.dsr2.2017.10.005 | Bober S, Brix S, Riehl T, Schwentner M, Brandt A (2018) Does the Mid-Atlantic Ridge affect the distribution of abyssal benthic crustaceans across the Atlantic Ocean? Deep-Sea Research Part II: Topical Studies in Oceanography 148: 91–104. https://doi.org/10.1016/j.dsr2.2018.02.017; associatedSequences: https://www.ncbi.nlm.nih.gov/nuccore/LT909308.1 | https://www.ncbi.nlm.nih.gov/nuccore/LT960454.1; occurrenceID: 78521BA3-8316-5272-83AE-448DFDB28C29; **Taxon:** scientificName: *Macrostylispapandreas* Johannsen, Riehl & Brandt; kingdom: Animalia; phylum: Arthropoda; class: Malacostraca; order: Isopoda; family: Macrostylidae; genus: Macrostylis; specificEpithet: *papandreas*; scientificNameAuthorship: Johannsen, Riehl & Brandt; nomenclaturalCode: ICZN; **Location:** higherGeography: Atlantic Ocean; locality: Eastern Vema Fracture Zone, Vema-TRANSIT station SO237-6-17; verbatimDepth: 5082 m; verbatimLatitude: 10°21.82' N; verbatimLongitude: 36°55.06' W; decimalLatitude: 10.36367; decimalLongitude: 36.91767; **Identification:** identifiedBy: Torben Riehl, Simon Bober; **Event:** samplingProtocol: Benthos trawl, Camera-Epibenthic Sledge, sieved through 0.3 mm mesh | Riehl T, Brenke N, Brix S, Driskell A, Kaiser S, Brandt A (2014) Field and laboratory methods for DNA studies on deep-sea isopod crustaceans. Polish Polar Research 35: 205–226. https://doi.org/10.2478/popore−2014−0018 | Devey CW (2015) RV SONNE Fahrtbericht / Cruise Report SO237 Vema-TRANSIT. Geomar Report 23: 130. https://doi.org/10.3289/GEOMAR_REP_NS_23_2025; eventDate: 02/01/2015; habitat: abyssal sediment; fieldNumber: SO237-6-7; **Record Level:** institutionCode: ZMH; collectionCode: K; basisOfRecord: PreservedSpecimen
- **Type status:** Paratype. **Occurrence:** catalogNumber: ZMH K-45153; recordNumber: VTMac157; individualCount: 1; sex: female; lifeStage: adult; preparations: whole animal (ETOH); previousIdentifications: *Macrostylis* sp. Mlpap | MOTU Mlpap; associatedReferences: Riehl T, Lins L, Brandt A (2018) The effects of depth, distance, and the Mid-Atlantic Ridge on genetic differentiation of abyssal and hadal isopods (Macrostylidae). Deep Sea Research Part II: Topical Studies in Oceanography 148: 74–90. https://doi.org/10.1016/j.dsr2.2017.10.005 | Bober S, Brix S, Riehl T, Schwentner M, Brandt A (2018) Does the Mid-Atlantic Ridge affect the distribution of abyssal benthic crustaceans across the Atlantic Ocean? Deep-Sea Research Part II: Topical Studies in Oceanography 148: 91–104. https://doi.org/10.1016/j.dsr2.2018.02.018; associatedSequences: https://www.ncbi.nlm.nih.gov/nuccore/LT909309.1 | https://www.ncbi.nlm.nih.gov/nuccore/LT960455.1; occurrenceID: 3AB1E376-03DA-52AC-9411-5F56053D54B8; **Taxon:** scientificName: *Macrostylispapandreas* Johannsen, Riehl & Brandt; kingdom: Animalia; phylum: Arthropoda; class: Malacostraca; order: Isopoda; family: Macrostylidae; genus: Macrostylis; specificEpithet: *papandreas*; scientificNameAuthorship: Johannsen, Riehl & Brandt; nomenclaturalCode: ICZN; **Location:** higherGeography: Atlantic Ocean; locality: Eastern Vema Fracture Zone, Vema-TRANSIT station SO237-6-18; verbatimDepth: 5082 m; verbatimLatitude: 10°21.82' N; verbatimLongitude: 36°55.06' W; decimalLatitude: 10.36367; decimalLongitude: 36.91767; **Identification:** identifiedBy: Torben Riehl, Simon Bober; **Event:** samplingProtocol: Benthos trawl, Camera-Epibenthic Sledge, sieved through 0.3 mm mesh | Riehl T, Brenke N, Brix S, Driskell A, Kaiser S, Brandt A (2014) Field and laboratory methods for DNA studies on deep-sea isopod crustaceans. Polish Polar Research 35: 205–226. https://doi.org/10.2478/popore−2014−0018 | Devey CW (2015) RV SONNE Fahrtbericht / Cruise Report SO237 Vema-TRANSIT. Geomar Report 23: 130. https://doi.org/10.3289/GEOMAR_REP_NS_23_2026; eventDate: 02/01/2015; habitat: abyssal sediment; fieldNumber: SO237-6-7; **Record Level:** institutionCode: ZMH; collectionCode: K; basisOfRecord: PreservedSpecimen
- **Type status:** Paratype. **Occurrence:** catalogNumber: ZMH K-45154; recordNumber: VTMac166; individualCount: 1; sex: female; lifeStage: adult; preparations: whole animal (ETOH); previousIdentifications: *Macrostylis* sp. Mlpap | MOTU Mlpap; associatedReferences: Riehl T, Lins L, Brandt A (2018) The effects of depth, distance, and the Mid-Atlantic Ridge on genetic differentiation of abyssal and hadal isopods (Macrostylidae). Deep Sea Research Part II: Topical Studies in Oceanography 148: 74–90. https://doi.org/10.1016/j.dsr2.2017.10.005 | Bober S, Brix S, Riehl T, Schwentner M, Brandt A (2018) Does the Mid-Atlantic Ridge affect the distribution of abyssal benthic crustaceans across the Atlantic Ocean? Deep-Sea Research Part II: Topical Studies in Oceanography 148: 91–104. https://doi.org/10.1016/j.dsr2.2018.02.019; associatedSequences: https://www.ncbi.nlm.nih.gov/nuccore/LT909318.1; occurrenceID: 1D8DCEAA-CF02-5DBC-B7BF-4F9B56720F72; **Taxon:** scientificName: *Macrostylispapandreas* Johannsen, Riehl & Brandt; kingdom: Animalia; phylum: Arthropoda; class: Malacostraca; order: Isopoda; family: Macrostylidae; genus: Macrostylis; specificEpithet: *papandreas*; scientificNameAuthorship: Johannsen, Riehl & Brandt; nomenclaturalCode: ICZN; **Location:** higherGeography: Atlantic Ocean; locality: Eastern Vema Fracture Zone, Vema-TRANSIT station SO237-6-19; verbatimDepth: 5082 m; verbatimLatitude: 10°21.82' N; verbatimLongitude: 36°55.06' W; decimalLatitude: 10.36367; decimalLongitude: 36.91767; **Identification:** identifiedBy: Torben Riehl, Simon Bober; **Event:** samplingProtocol: Benthos trawl, Camera-Epibenthic Sledge, sieved through 0.3 mm mesh | Riehl T, Brenke N, Brix S, Driskell A, Kaiser S, Brandt A (2014) Field and laboratory methods for DNA studies on deep-sea isopod crustaceans. Polish Polar Research 35: 205–226. https://doi.org/10.2478/popore−2014−0018 | Devey CW (2015) RV SONNE Fahrtbericht / Cruise Report SO237 Vema-TRANSIT. Geomar Report 23: 130. https://doi.org/10.3289/GEOMAR_REP_NS_23_2027; eventDate: 02/01/2015; habitat: abyssal sediment; fieldNumber: SO237-6-7; **Record Level:** institutionCode: ZMH; collectionCode: K; basisOfRecord: PreservedSpecimen
- **Type status:** Paratype. **Occurrence:** catalogNumber: ZMH K-45155; recordNumber: VTMac167; individualCount: 1; sex: female; lifeStage: adult; preparations: whole animal (ETOH); previousIdentifications: *Macrostylis* sp. Mlpap | MOTU Mlpap; associatedReferences: Riehl T, Lins L, Brandt A (2018) The effects of depth, distance, and the Mid-Atlantic Ridge on genetic differentiation of abyssal and hadal isopods (Macrostylidae). Deep Sea Research Part II: Topical Studies in Oceanography 148: 74–90. https://doi.org/10.1016/j.dsr2.2017.10.005 | Bober S, Brix S, Riehl T, Schwentner M, Brandt A (2018) Does the Mid-Atlantic Ridge affect the distribution of abyssal benthic crustaceans across the Atlantic Ocean? Deep-Sea Research Part II: Topical Studies in Oceanography 148: 91–104. https://doi.org/10.1016/j.dsr2.2018.02.020; associatedSequences: https://www.ncbi.nlm.nih.gov/nuccore/LT909319.1 | https://www.ncbi.nlm.nih.gov/nuccore/LT960456.1; occurrenceID: 077F3413-E76D-5D2A-9483-99DBC43F3D1C; **Taxon:** scientificName: *Macrostylispapandreas* Johannsen, Riehl & Brandt; kingdom: Animalia; phylum: Arthropoda; class: Malacostraca; order: Isopoda; family: Macrostylidae; genus: Macrostylis; specificEpithet: *papandreas*; scientificNameAuthorship: Johannsen, Riehl & Brandt; nomenclaturalCode: ICZN; **Location:** higherGeography: Atlantic Ocean; locality: Eastern Vema Fracture Zone, Vema-TRANSIT station SO237-6-20; verbatimDepth: 5082 m; verbatimLatitude: 10°21.82' N; verbatimLongitude: 36°55.06' W; decimalLatitude: 10.36367; decimalLongitude: 36.91767; **Identification:** identifiedBy: Torben Riehl, Simon Bober; **Event:** samplingProtocol: Benthos trawl, Camera-Epibenthic Sledge, sieved through 0.3 mm mesh | Riehl T, Brenke N, Brix S, Driskell A, Kaiser S, Brandt A (2014) Field and laboratory methods for DNA studies on deep-sea isopod crustaceans. Polish Polar Research 35: 205–226. https://doi.org/10.2478/popore−2014−0018 | Devey CW (2015) RV SONNE Fahrtbericht / Cruise Report SO237 Vema-TRANSIT. Geomar Report 23: 130. https://doi.org/10.3289/GEOMAR_REP_NS_23_2028; eventDate: 02/01/2015; habitat: abyssal sediment; fieldNumber: SO237-6-7; **Record Level:** institutionCode: ZMH; collectionCode: K; basisOfRecord: PreservedSpecimen
- **Type status:** Other material. **Occurrence:** catalogNumber: ZMH K-45156; recordNumber: VTMac182; individualCount: 1; sex: female; lifeStage: ovigerous; preparations: whole animal (ETOH); previousIdentifications: *Macrostylis* sp. Mlpap | MOTU Mlpap; associatedReferences: Riehl T, Lins L, Brandt A (2018) The effects of depth, distance, and the Mid-Atlantic Ridge on genetic differentiation of abyssal and hadal isopods (Macrostylidae). Deep Sea Research Part II: Topical Studies in Oceanography 148: 74–90. https://doi.org/10.1016/j.dsr2.2017.10.005 | Bober S, Brix S, Riehl T, Schwentner M, Brandt A (2018) Does the Mid-Atlantic Ridge affect the distribution of abyssal benthic crustaceans across the Atlantic Ocean? Deep-Sea Research Part II: Topical Studies in Oceanography 148: 91–104. https://doi.org/10.1016/j.dsr2.2018.02.021; associatedSequences: https://www.ncbi.nlm.nih.gov/nuccore/LT909333.1 | https://www.ncbi.nlm.nih.gov/nuccore/LT960457.1; occurrenceID: F758E488-918A-57D0-B9C5-6594B0DDFE14; **Taxon:** scientificName: *Macrostylispapandreas* Johannsen, Riehl & Brandt; kingdom: Animalia; phylum: Arthropoda; class: Malacostraca; order: Isopoda; family: Macrostylidae; genus: Macrostylis; specificEpithet: *papandreas*; scientificNameAuthorship: Johannsen, Riehl & Brandt; nomenclaturalCode: ICZN; **Location:** higherGeography: Atlantic Ocean; locality: Western Vema Fracture Zone, Vema-TRANSIT station SO237-9-8; verbatimDepth: 5002.5 m; verbatimLatitude: 11°39.36' N; verbatimLongitude: 47°53.99' W; decimalLatitude: 11.656; decimalLongitude: 47.89983; **Identification:** identifiedBy: Torben Riehl, Simon Bober; **Event:** samplingProtocol: Benthos trawl, Camera-Epibenthic Sledge, sieved through 0.3 mm mesh | Riehl T, Brenke N, Brix S, Driskell A, Kaiser S, Brandt A (2014) Field and laboratory methods for DNA studies on deep-sea isopod crustaceans. Polish Polar Research 35: 205–226. https://doi.org/10.2478/popore−2014−0018 | Devey CW (2015) RV SONNE Fahrtbericht / Cruise Report SO237 Vema-TRANSIT. Geomar Report 23: 130. https://doi.org/10.3289/GEOMAR_REP_NS_23_2029; eventDate: 12/01/2015; habitat: abyssal sediment; fieldNumber: SO237-9-8; **Record Level:** institutionCode: ZMH; collectionCode: K; basisOfRecord: PreservedSpecimen
- **Type status:** Other material. **Occurrence:** catalogNumber: ZMH K-45157; recordNumber: VTMac183; individualCount: 1; sex: female; lifeStage: adult; preparations: whole animal (ETOH); previousIdentifications: *Macrostylis* sp. Mlpap | MOTU Mlpap; associatedReferences: Riehl T, Lins L, Brandt A (2018) The effects of depth, distance, and the Mid-Atlantic Ridge on genetic differentiation of abyssal and hadal isopods (Macrostylidae). Deep Sea Research Part II: Topical Studies in Oceanography 148: 74–90. https://doi.org/10.1016/j.dsr2.2017.10.005 | Bober S, Brix S, Riehl T, Schwentner M, Brandt A (2018) Does the Mid-Atlantic Ridge affect the distribution of abyssal benthic crustaceans across the Atlantic Ocean? Deep-Sea Research Part II: Topical Studies in Oceanography 148: 91–104. https://doi.org/10.1016/j.dsr2.2018.02.022; associatedSequences: https://www.ncbi.nlm.nih.gov/nuccore/MN735415.1 | https://www.ncbi.nlm.nih.gov/nuccore/LT909334.1 | https://www.ncbi.nlm.nih.gov/nuccore/LT960458.1; occurrenceID: 36D89543-2F04-5CC5-8F93-6A6FD4CFB9AC; **Taxon:** scientificName: *Macrostylispapandreas* Johannsen, Riehl & Brandt; kingdom: Animalia; phylum: Arthropoda; class: Malacostraca; order: Isopoda; family: Macrostylidae; genus: Macrostylis; specificEpithet: *papandreas*; scientificNameAuthorship: Johannsen, Riehl & Brandt; nomenclaturalCode: ICZN; **Location:** higherGeography: Atlantic Ocean; locality: Western Vema Fracture Zone, Vema-TRANSIT station SO237-9-8; verbatimDepth: 5002.5 m; verbatimLatitude: 11°39.36' N; verbatimLongitude: 47°53.99' W; decimalLatitude: 11.656; decimalLongitude: 47.89983; **Identification:** identifiedBy: Torben Riehl, Simon Bober; **Event:** samplingProtocol: Benthos trawl, Camera-Epibenthic Sledge, sieved through 0.3 mm mesh | Riehl T, Brenke N, Brix S, Driskell A, Kaiser S, Brandt A (2014) Field and laboratory methods for DNA studies on deep-sea isopod crustaceans. Polish Polar Research 35: 205–226. https://doi.org/10.2478/popore−2014−0018 | Devey CW (2015) RV SONNE Fahrtbericht / Cruise Report SO237 Vema-TRANSIT. Geomar Report 23: 130. https://doi.org/10.3289/GEOMAR_REP_NS_23_2030; eventDate: 12/01/2015; habitat: abyssal sediment; fieldNumber: SO237-9-8; **Record Level:** institutionCode: ZMH; collectionCode: K; basisOfRecord: PreservedSpecimen
- **Type status:** Other material. **Occurrence:** catalogNumber: ZMH K-45158; recordNumber: VTMac184; individualCount: 1; sex: female; lifeStage: adult; preparations: whole animal (ETOH); previousIdentifications: *Macrostylis* sp. Mlpap | MOTU Mlpap; associatedReferences: Riehl T, Lins L, Brandt A (2018) The effects of depth, distance, and the Mid-Atlantic Ridge on genetic differentiation of abyssal and hadal isopods (Macrostylidae). Deep Sea Research Part II: Topical Studies in Oceanography 148: 74–90. https://doi.org/10.1016/j.dsr2.2017.10.005 | Bober S, Brix S, Riehl T, Schwentner M, Brandt A (2018) Does the Mid-Atlantic Ridge affect the distribution of abyssal benthic crustaceans across the Atlantic Ocean? Deep-Sea Research Part II: Topical Studies in Oceanography 148: 91–104. https://doi.org/10.1016/j.dsr2.2018.02.023; associatedSequences: https://www.ncbi.nlm.nih.gov/nuccore/MN735416.1 | https://www.ncbi.nlm.nih.gov/nuccore/LT909335.1 | https://www.ncbi.nlm.nih.gov/nuccore/LT960459.1; occurrenceID: 86A1304A-19D9-55D0-A781-2D8656D5E24A; **Taxon:** scientificName: *Macrostylispapandreas* Johannsen, Riehl & Brandt; kingdom: Animalia; phylum: Arthropoda; class: Malacostraca; order: Isopoda; family: Macrostylidae; genus: Macrostylis; specificEpithet: *papandreas*; scientificNameAuthorship: Johannsen, Riehl & Brandt; nomenclaturalCode: ICZN; **Location:** higherGeography: Atlantic Ocean; locality: Western Vema Fracture Zone, Vema-TRANSIT station SO237-9-8; verbatimDepth: 5002.5 m; verbatimLatitude: 11°39.36' N; verbatimLongitude: 47°53.99' W; decimalLatitude: 11.656; decimalLongitude: 47.89983; **Identification:** identifiedBy: Torben Riehl, Simon Bober; **Event:** samplingProtocol: Benthos trawl, Camera-Epibenthic Sledge, sieved through 0.3 mm mesh | Riehl T, Brenke N, Brix S, Driskell A, Kaiser S, Brandt A (2014) Field and laboratory methods for DNA studies on deep-sea isopod crustaceans. Polish Polar Research 35: 205–226. https://doi.org/10.2478/popore−2014−0018 | Devey CW (2015) RV SONNE Fahrtbericht / Cruise Report SO237 Vema-TRANSIT. Geomar Report 23: 130. https://doi.org/10.3289/GEOMAR_REP_NS_23_2031; eventDate: 12/01/2015; habitat: abyssal sediment; fieldNumber: SO237-9-8; **Record Level:** institutionCode: ZMH; collectionCode: K; basisOfRecord: PreservedSpecimen
- **Type status:** Other material. **Occurrence:** catalogNumber: ZMH K-45145; recordNumber: VTMac186; individualCount: 1; sex: neuter; lifeStage: manca; preparations: whole animal (ETOH); previousIdentifications: *Macrostylis* sp. Mlpap | MOTU Mlpap; associatedReferences: Riehl T, Lins L, Brandt A (2018) The effects of depth, distance, and the Mid-Atlantic Ridge on genetic differentiation of abyssal and hadal isopods (Macrostylidae). Deep Sea Research Part II: Topical Studies in Oceanography 148: 74–90. https://doi.org/10.1016/j.dsr2.2017.10.005 | Bober S, Brix S, Riehl T, Schwentner M, Brandt A (2018) Does the Mid-Atlantic Ridge affect the distribution of abyssal benthic crustaceans across the Atlantic Ocean? Deep-Sea Research Part II: Topical Studies in Oceanography 148: 91–104. https://doi.org/10.1016/j.dsr2.2018.02.024; associatedSequences: https://www.ncbi.nlm.nih.gov/nuccore/MN735418.1 | https://www.ncbi.nlm.nih.gov/nuccore/LT909337.1 | https://www.ncbi.nlm.nih.gov/nuccore/LT960460.1; occurrenceID: 1CE6F0F0-B733-5B2E-BFE3-AAFADF30F34A; **Taxon:** scientificName: *Macrostylispapandreas* Johannsen, Riehl & Brandt; kingdom: Animalia; phylum: Arthropoda; class: Malacostraca; order: Isopoda; family: Macrostylidae; genus: Macrostylis; specificEpithet: *papandreas*; scientificNameAuthorship: Johannsen, Riehl & Brandt; nomenclaturalCode: ICZN; **Location:** higherGeography: Atlantic Ocean; locality: Western Vema Fracture Zone, Vema-TRANSIT station SO237-9-8; verbatimDepth: 5002.5 m; verbatimLatitude: 11°39.36' N; verbatimLongitude: 47°53.99' W; decimalLatitude: 11.656; decimalLongitude: 47.89983; **Identification:** identifiedBy: Torben Riehl, Simon Bober; **Event:** samplingProtocol: Benthos trawl, Camera-Epibenthic Sledge, sieved through 0.3 mm mesh | Riehl T, Brenke N, Brix S, Driskell A, Kaiser S, Brandt A (2014) Field and laboratory methods for DNA studies on deep-sea isopod crustaceans. Polish Polar Research 35: 205–226. https://doi.org/10.2478/popore−2014−0018 | Devey CW (2015) RV SONNE Fahrtbericht / Cruise Report SO237 Vema-TRANSIT. Geomar Report 23: 130. https://doi.org/10.3289/GEOMAR_REP_NS_23_2032; eventDate: 12/01/2015; habitat: abyssal sediment; fieldNumber: SO237-9-8; **Record Level:** institutionCode: ZMH; collectionCode: K; basisOfRecord: PreservedSpecimen
- **Type status:** Other material. **Occurrence:** catalogNumber: ZMH K-45169; recordNumber: VTMac187; individualCount: 1; sex: male; lifeStage: adult; preparations: whole animal (ETOH); previousIdentifications: *Macrostylis* sp. Mlpap | MOTU Mlpap; associatedReferences: Riehl T, Lins L, Brandt A (2018) The effects of depth, distance, and the Mid-Atlantic Ridge on genetic differentiation of abyssal and hadal isopods (Macrostylidae). Deep Sea Research Part II: Topical Studies in Oceanography 148: 74–90. https://doi.org/10.1016/j.dsr2.2017.10.005 | Bober S, Brix S, Riehl T, Schwentner M, Brandt A (2018) Does the Mid-Atlantic Ridge affect the distribution of abyssal benthic crustaceans across the Atlantic Ocean? Deep-Sea Research Part II: Topical Studies in Oceanography 148: 91–104. https://doi.org/10.1016/j.dsr2.2018.02.025; associatedSequences: https://www.ncbi.nlm.nih.gov/nuccore/MN735419.1 | https://www.ncbi.nlm.nih.gov/nuccore/LT909338.1; occurrenceID: 9394B072-47B1-5931-A983-7D8D0A48DE0F; **Taxon:** scientificName: *Macrostylispapandreas* Johannsen, Riehl & Brandt; kingdom: Animalia; phylum: Arthropoda; class: Malacostraca; order: Isopoda; family: Macrostylidae; genus: Macrostylis; specificEpithet: *papandreas*; scientificNameAuthorship: Johannsen, Riehl & Brandt; nomenclaturalCode: ICZN; **Location:** higherGeography: Atlantic Ocean; locality: Western Vema Fracture Zone, Vema-TRANSIT station SO237-11-1; verbatimDepth: 5090.5 m; verbatimLatitude: 12°05.84' N; verbatimLongitude: 50°27.97' W; decimalLatitude: 12.0973; decimalLongitude: 50.466167; **Identification:** identifiedBy: Torben Riehl, Simon Bober; **Event:** samplingProtocol: Benthos trawl, Camera-Epibenthic Sledge, sieved through 0.3 mm mesh | Riehl T, Brenke N, Brix S, Driskell A, Kaiser S, Brandt A (2014) Field and laboratory methods for DNA studies on deep-sea isopod crustaceans. Polish Polar Research 35: 205–226. https://doi.org/10.2478/popore−2014−0018 | Devey CW (2015) RV SONNE Fahrtbericht / Cruise Report SO237 Vema-TRANSIT. Geomar Report 23: 130. https://doi.org/10.3289/GEOMAR_REP_NS_23_2033; eventDate: 14/01/2015; habitat: abyssal sediment; fieldNumber: SO237-11-1; **Record Level:** institutionCode: ZMH; collectionCode: K; basisOfRecord: PreservedSpecimen
- **Type status:** Other material. **Occurrence:** catalogNumber: ZMH K-45159; recordNumber: VTMac189; individualCount: 1; sex: female; lifeStage: ovigerous; preparations: whole animal (ETOH); previousIdentifications: *Macrostylis* sp. Mlpap | MOTU Mlpap; associatedReferences: Riehl T, Lins L, Brandt A (2018) The effects of depth, distance, and the Mid-Atlantic Ridge on genetic differentiation of abyssal and hadal isopods (Macrostylidae). Deep Sea Research Part II: Topical Studies in Oceanography 148: 74–90. https://doi.org/10.1016/j.dsr2.2017.10.005 | Bober S, Brix S, Riehl T, Schwentner M, Brandt A (2018) Does the Mid-Atlantic Ridge affect the distribution of abyssal benthic crustaceans across the Atlantic Ocean? Deep-Sea Research Part II: Topical Studies in Oceanography 148: 91–104. https://doi.org/10.1016/j.dsr2.2018.02.026; associatedSequences: https://www.ncbi.nlm.nih.gov/nuccore/LT909340.1; occurrenceID: 6D85388A-8C77-5108-8590-AC26EA612546; **Taxon:** scientificName: *Macrostylispapandreas* Johannsen, Riehl & Brandt; kingdom: Animalia; phylum: Arthropoda; class: Malacostraca; order: Isopoda; family: Macrostylidae; genus: Macrostylis; specificEpithet: *papandreas*; scientificNameAuthorship: Johannsen, Riehl & Brandt; nomenclaturalCode: ICZN; **Location:** higherGeography: Atlantic Ocean; locality: Western Vema Fracture Zone, Vema-TRANSIT station SO237-11-1; verbatimDepth: 5090.5 m; verbatimLatitude: 12°05.84' N; verbatimLongitude: 50°27.97' W; decimalLatitude: 12.0973; decimalLongitude: 50.466167; **Identification:** identifiedBy: Torben Riehl, Simon Bober; **Event:** samplingProtocol: Benthos trawl, Camera-Epibenthic Sledge, sieved through 0.3 mm mesh | Riehl T, Brenke N, Brix S, Driskell A, Kaiser S, Brandt A (2014) Field and laboratory methods for DNA studies on deep-sea isopod crustaceans. Polish Polar Research 35: 205–226. https://doi.org/10.2478/popore−2014−0018 | Devey CW (2015) RV SONNE Fahrtbericht / Cruise Report SO237 Vema-TRANSIT. Geomar Report 23: 130. https://doi.org/10.3289/GEOMAR_REP_NS_23_2034; eventDate: 14/01/2015; habitat: abyssal sediment; fieldNumber: SO237-11-1; **Record Level:** institutionCode: ZMH; collectionCode: K; basisOfRecord: PreservedSpecimen
- **Type status:** Other material. **Occurrence:** catalogNumber: ZMH K-45160; recordNumber: VTMac190; individualCount: 1; sex: female; lifeStage: adult; preparations: whole animal (ETOH); previousIdentifications: *Macrostylis* sp. Mlpap | MOTU Mlpap; associatedReferences: Riehl T, Lins L, Brandt A (2018) The effects of depth, distance, and the Mid-Atlantic Ridge on genetic differentiation of abyssal and hadal isopods (Macrostylidae). Deep Sea Research Part II: Topical Studies in Oceanography 148: 74–90. https://doi.org/10.1016/j.dsr2.2017.10.005 | Bober S, Brix S, Riehl T, Schwentner M, Brandt A (2018) Does the Mid-Atlantic Ridge affect the distribution of abyssal benthic crustaceans across the Atlantic Ocean? Deep-Sea Research Part II: Topical Studies in Oceanography 148: 91–104. https://doi.org/10.1016/j.dsr2.2018.02.027; associatedSequences: https://www.ncbi.nlm.nih.gov/nuccore/LT909341.1; occurrenceID: 54F30307-3ED1-5EA6-A41A-B57052D9C427; **Taxon:** scientificName: *Macrostylispapandreas* Johannsen, Riehl & Brandt; kingdom: Animalia; phylum: Arthropoda; class: Malacostraca; order: Isopoda; family: Macrostylidae; genus: Macrostylis; specificEpithet: *papandreas*; scientificNameAuthorship: Johannsen, Riehl & Brandt; nomenclaturalCode: ICZN; **Location:** higherGeography: Atlantic Ocean; locality: Western Vema Fracture Zone, Vema-TRANSIT station SO237-11-1; verbatimDepth: 5090.5 m; verbatimLatitude: 12°05.84' N; verbatimLongitude: 50°27.97' W; decimalLatitude: 12.0973; decimalLongitude: 50.466167; **Identification:** identifiedBy: Torben Riehl, Simon Bober; **Event:** samplingProtocol: Benthos trawl, Camera-Epibenthic Sledge, sieved through 0.3 mm mesh | Riehl T, Brenke N, Brix S, Driskell A, Kaiser S, Brandt A (2014) Field and laboratory methods for DNA studies on deep-sea isopod crustaceans. Polish Polar Research 35: 205–226. https://doi.org/10.2478/popore−2014−0018 | Devey CW (2015) RV SONNE Fahrtbericht / Cruise Report SO237 Vema-TRANSIT. Geomar Report 23: 130. https://doi.org/10.3289/GEOMAR_REP_NS_23_2035; eventDate: 14/01/2015; habitat: abyssal sediment; fieldNumber: SO237-11-1; **Record Level:** institutionCode: ZMH; collectionCode: K; basisOfRecord: PreservedSpecimen
- **Type status:** Other material. **Occurrence:** catalogNumber: ZMH K-45161; recordNumber: VTMac192; individualCount: 1; sex: female; lifeStage: ovigerous; preparations: whole animal (ETOH); previousIdentifications: *Macrostylis* sp. Mlpap | MOTU Mlpap; associatedReferences: Riehl T, Lins L, Brandt A (2018) The effects of depth, distance, and the Mid-Atlantic Ridge on genetic differentiation of abyssal and hadal isopods (Macrostylidae). Deep Sea Research Part II: Topical Studies in Oceanography 148: 74–90. https://doi.org/10.1016/j.dsr2.2017.10.005 | Bober S, Brix S, Riehl T, Schwentner M, Brandt A (2018) Does the Mid-Atlantic Ridge affect the distribution of abyssal benthic crustaceans across the Atlantic Ocean? Deep-Sea Research Part II: Topical Studies in Oceanography 148: 91–104. https://doi.org/10.1016/j.dsr2.2018.02.028; associatedSequences: https://www.ncbi.nlm.nih.gov/nuccore/LT909343.1; occurrenceID: F3E2DBEB-7968-5FDF-A61B-766C7B8E3BBC; **Taxon:** scientificName: *Macrostylispapandreas* Johannsen, Riehl & Brandt; kingdom: Animalia; phylum: Arthropoda; class: Malacostraca; order: Isopoda; family: Macrostylidae; genus: Macrostylis; specificEpithet: *papandreas*; scientificNameAuthorship: Johannsen, Riehl & Brandt; nomenclaturalCode: ICZN; **Location:** higherGeography: Atlantic Ocean; locality: Western Vema Fracture Zone, Vema-TRANSIT station SO237-9-8; verbatimDepth: 5002.5 m; verbatimLatitude: 11°39.36' N; verbatimLongitude: 47°53.99' W; decimalLatitude: 11.656; decimalLongitude: 47.89983; **Identification:** identifiedBy: Torben Riehl, Simon Bober; **Event:** samplingProtocol: Benthos trawl, Camera-Epibenthic Sledge, sieved through 0.3 mm mesh | Riehl T, Brenke N, Brix S, Driskell A, Kaiser S, Brandt A (2014) Field and laboratory methods for DNA studies on deep-sea isopod crustaceans. Polish Polar Research 35: 205–226. https://doi.org/10.2478/popore−2014−0018 | Devey CW (2015) RV SONNE Fahrtbericht / Cruise Report SO237 Vema-TRANSIT. Geomar Report 23: 130. https://doi.org/10.3289/GEOMAR_REP_NS_23_2036; eventDate: 12/01/2015; habitat: abyssal sediment; fieldNumber: SO237-9-8; **Record Level:** institutionCode: ZMH; collectionCode: K; basisOfRecord: PreservedSpecimen
- **Type status:** Other material. **Occurrence:** catalogNumber: ZMH K-45162; recordNumber: VTMac194; individualCount: 1; sex: female; lifeStage: ovigerous; preparations: whole animal (ETOH); previousIdentifications: *Macrostylis* sp. Mlpap | MOTU Mlpap; associatedReferences: Riehl T, Lins L, Brandt A (2018) The effects of depth, distance, and the Mid-Atlantic Ridge on genetic differentiation of abyssal and hadal isopods (Macrostylidae). Deep Sea Research Part II: Topical Studies in Oceanography 148: 74–90. https://doi.org/10.1016/j.dsr2.2017.10.005 | Bober S, Brix S, Riehl T, Schwentner M, Brandt A (2018) Does the Mid-Atlantic Ridge affect the distribution of abyssal benthic crustaceans across the Atlantic Ocean? Deep-Sea Research Part II: Topical Studies in Oceanography 148: 91–104. https://doi.org/10.1016/j.dsr2.2018.02.029; associatedSequences: https://www.ncbi.nlm.nih.gov/nuccore/MN735421.1 | https://www.ncbi.nlm.nih.gov/nuccore/LT909344.1 | https://www.ncbi.nlm.nih.gov/nuccore/LT960461.1; occurrenceID: 371C1BA6-CF27-59CD-883C-439FCBB44667; **Taxon:** scientificName: *Macrostylispapandreas* Johannsen, Riehl & Brandt; kingdom: Animalia; phylum: Arthropoda; class: Malacostraca; order: Isopoda; family: Macrostylidae; genus: Macrostylis; specificEpithet: *papandreas*; scientificNameAuthorship: Johannsen, Riehl & Brandt; nomenclaturalCode: ICZN; **Location:** higherGeography: Atlantic Ocean; locality: Western Vema Fracture Zone, Vema-TRANSIT station SO237-11-4; verbatimDepth: 5119 m; verbatimLatitude: 12°04.83' N; verbatimLongitude: 50°28.14' W; decimalLatitude: 12.0805; decimalLongitude: 50.469; **Identification:** identifiedBy: Torben Riehl, Simon Bober; **Event:** samplingProtocol: Benthos trawl, Camera-Epibenthic Sledge, sieved through 0.3 mm mesh | Riehl T, Brenke N, Brix S, Driskell A, Kaiser S, Brandt A (2014) Field and laboratory methods for DNA studies on deep-sea isopod crustaceans. Polish Polar Research 35: 205–226. https://doi.org/10.2478/popore−2014−0018 | Devey CW (2015) RV SONNE Fahrtbericht / Cruise Report SO237 Vema-TRANSIT. Geomar Report 23: 130. https://doi.org/10.3289/GEOMAR_REP_NS_23_2037; eventDate: 14/01/2015; habitat: abyssal sediment; fieldNumber: SO237-11-4; **Record Level:** institutionCode: ZMH; collectionCode: K; basisOfRecord: PreservedSpecimen
- **Type status:** Other material. **Occurrence:** catalogNumber: ZMH K-45163; recordNumber: VTMac197; individualCount: 1; sex: female; lifeStage: ovigerous; preparations: whole animal (ETOH); previousIdentifications: *Macrostylis* sp. Mlpap | MOTU Mlpap; associatedReferences: Riehl T, Lins L, Brandt A (2018) The effects of depth, distance, and the Mid-Atlantic Ridge on genetic differentiation of abyssal and hadal isopods (Macrostylidae). Deep Sea Research Part II: Topical Studies in Oceanography 148: 74–90. https://doi.org/10.1016/j.dsr2.2017.10.005 | Bober S, Brix S, Riehl T, Schwentner M, Brandt A (2018) Does the Mid-Atlantic Ridge affect the distribution of abyssal benthic crustaceans across the Atlantic Ocean? Deep-Sea Research Part II: Topical Studies in Oceanography 148: 91–104. https://doi.org/10.1016/j.dsr2.2018.02.030; associatedSequences: https://www.ncbi.nlm.nih.gov/nuccore/LT909347.1; occurrenceID: F5A9B2BC-9B66-5885-B3F5-EDC1C3899DAD; **Taxon:** scientificName: *Macrostylispapandreas* Johannsen, Riehl & Brandt; kingdom: Animalia; phylum: Arthropoda; class: Malacostraca; order: Isopoda; family: Macrostylidae; genus: Macrostylis; specificEpithet: *papandreas*; scientificNameAuthorship: Johannsen, Riehl & Brandt; nomenclaturalCode: ICZN; **Location:** higherGeography: Atlantic Ocean; locality: Western Vema Fracture Zone, Vema-TRANSIT station SO237-11-4; verbatimDepth: 5119 m; verbatimLatitude: 12°04.83' N; verbatimLongitude: 50°28.14' W; decimalLatitude: 12.0805; decimalLongitude: 50.469; **Identification:** identifiedBy: Torben Riehl, Simon Bober; **Event:** samplingProtocol: Benthos trawl, Camera-Epibenthic Sledge, sieved through 0.3 mm mesh | Riehl T, Brenke N, Brix S, Driskell A, Kaiser S, Brandt A (2014) Field and laboratory methods for DNA studies on deep-sea isopod crustaceans. Polish Polar Research 35: 205–226. https://doi.org/10.2478/popore−2014−0018 | Devey CW (2015) RV SONNE Fahrtbericht / Cruise Report SO237 Vema-TRANSIT. Geomar Report 23: 130. https://doi.org/10.3289/GEOMAR_REP_NS_23_2038; eventDate: 14/01/2015; habitat: abyssal sediment; fieldNumber: SO237-11-4; **Record Level:** institutionCode: ZMH; collectionCode: K; basisOfRecord: PreservedSpecimen
- **Type status:** Other material. **Occurrence:** catalogNumber: ZMH K-45164; recordNumber: VTMac198; individualCount: 1; sex: female; lifeStage: ovigerous; preparations: whole animal (ETOH); previousIdentifications: *Macrostylis* sp. Mlpap | MOTU Mlpap; associatedReferences: Riehl T, Lins L, Brandt A (2018) The effects of depth, distance, and the Mid-Atlantic Ridge on genetic differentiation of abyssal and hadal isopods (Macrostylidae). Deep Sea Research Part II: Topical Studies in Oceanography 148: 74–90. https://doi.org/10.1016/j.dsr2.2017.10.005 | Bober S, Brix S, Riehl T, Schwentner M, Brandt A (2018) Does the Mid-Atlantic Ridge affect the distribution of abyssal benthic crustaceans across the Atlantic Ocean? Deep-Sea Research Part II: Topical Studies in Oceanography 148: 91–104. https://doi.org/10.1016/j.dsr2.2018.02.031; associatedSequences: https://www.ncbi.nlm.nih.gov/nuccore/LT909348.1 | https://www.ncbi.nlm.nih.gov/nuccore/LT960462.1; occurrenceID: 54F0B37A-7871-5682-8038-C4211E7B6E98; **Taxon:** scientificName: *Macrostylispapandreas* Johannsen, Riehl & Brandt; kingdom: Animalia; phylum: Arthropoda; class: Malacostraca; order: Isopoda; family: Macrostylidae; genus: Macrostylis; specificEpithet: *papandreas*; scientificNameAuthorship: Johannsen, Riehl & Brandt; nomenclaturalCode: ICZN; **Location:** higherGeography: Atlantic Ocean; locality: Western Vema Fracture Zone, Vema-TRANSIT station SO237-11-4; verbatimDepth: 5119 m; verbatimLatitude: 12°04.83' N; verbatimLongitude: 50°28.14' W; decimalLatitude: 12.0805; decimalLongitude: 50.469; **Identification:** identifiedBy: Torben Riehl, Simon Bober; **Event:** samplingProtocol: Benthos trawl, Camera-Epibenthic Sledge, sieved through 0.3 mm mesh | Riehl T, Brenke N, Brix S, Driskell A, Kaiser S, Brandt A (2014) Field and laboratory methods for DNA studies on deep-sea isopod crustaceans. Polish Polar Research 35: 205–226. https://doi.org/10.2478/popore−2014−0018 | Devey CW (2015) RV SONNE Fahrtbericht / Cruise Report SO237 Vema-TRANSIT. Geomar Report 23: 130. https://doi.org/10.3289/GEOMAR_REP_NS_23_2039; eventDate: 14/01/2015; habitat: abyssal sediment; fieldNumber: SO237-11-4; **Record Level:** institutionCode: ZMH; collectionCode: K; basisOfRecord: PreservedSpecimen

#### Description

**Holotype**: non-ovigerous female, 2.3 mm, ZMH K-45148, Vema-TRANSIT station 6–7, designated here.

**Paratypes**: non-ovigerous female, 2.4 mm, ZMH K-45149, same locality as holotype; adult male, 2.0 mm, ZMH K-45166, dissected for illustration of the habitus and appendages, same locality as holotype; subadult male, 1.7 mm, ZMH K-45167, used for illustration of the habitus, same locality as holotype.

**Type locality**: Vema-TRANSIT expedition (SO-237) station 6–7: tropical North Atlantic Ocean, eastern Vema Fracture Zone, start trawl at 10° 20.659' N, 36° 57.010' W; 5085 m depth; end trawl at 10° 21.547' N, 36° 55.585' W; 5079 m depth; 02. January 2015, R/V SONNE.

**Further records**: Western Vema Fracture Zone, Vema-TRANSIT expedition (SO-237) stations SO237-9-8, SO237-11-1 and SO237-11-4.

**Description of non-ovigerous and ovigerous female**

**Figure 25.**
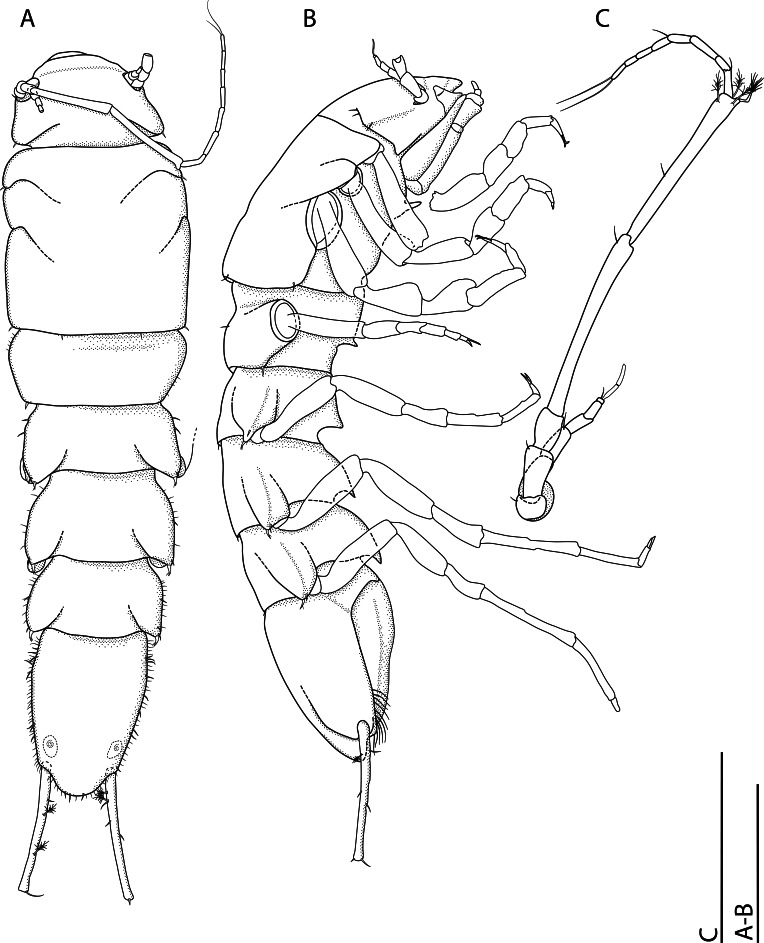
*Macrostylispapandreas* Johannsen, Riehl & Brandt, **sp. nov.** female habitus and antennae. **A–C.** Holotype non-ovigerous female (ZMH K-45148). **A.** Habitus dorsal, uropod endopods missing; **B.** Habitus lateral, uropod endopod missing; **C.** Antennae. Scale bars: 0.5 mm (A, B), 0.3 mm (C).

**Figure 26.**
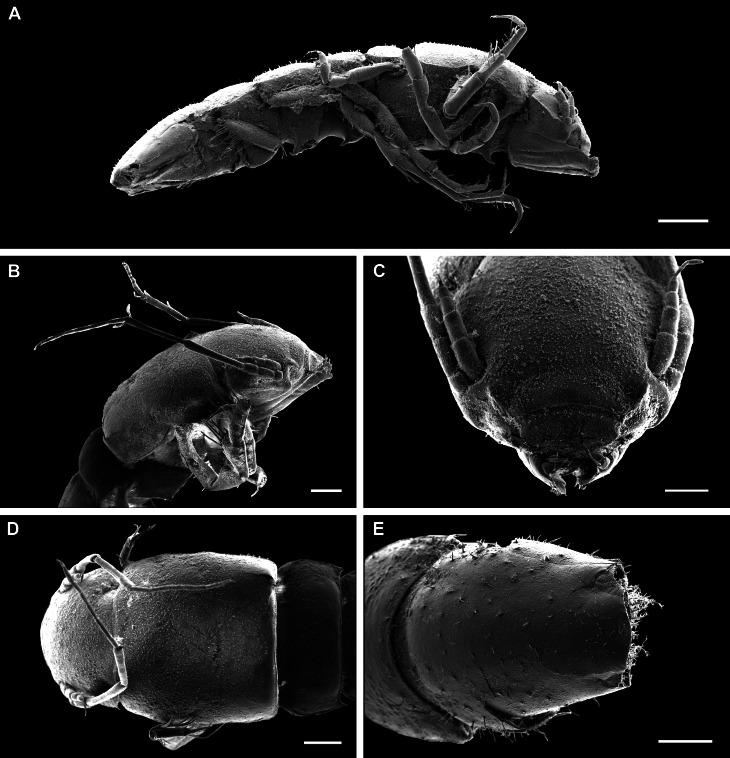
*Macrostylispapandreas* Johannsen, Riehl & Brandt, **sp. nov.** SEM images of female habitus and head appendages. **A**, **E.** Paratype non-ovigerous female (ZMH K-45150), **B–D**. Paratype non-ovigerous female (ZMH K-45154). **A.** Habitus lateral, antennae broken, pereopods 2 and 7 broken, uropod missing; **B.** Anterior habitus lateral, pereopod 3 broken; **C.** Head frontal; **D.** Fossosoma; **E.** Pleotelson dorsal. Scale bars: 200 µm (A); 100 µm (B, D–E), 60 µm (C).

**Figure 27.**
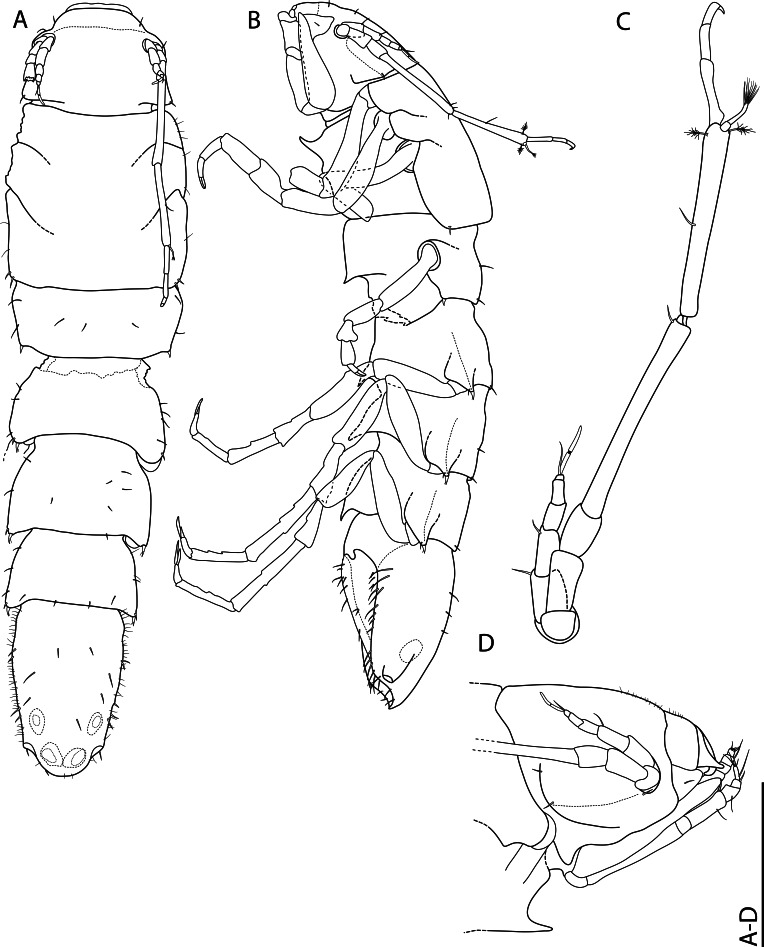
*Macrostylispapandreas* Johannsen, Riehl & Brandt, **sp. nov.** female habitus and head appendages. **A–D.** Paratype non-ovigerous female (ZMH K-45149). **A.** Habitus dorsal, uropods missing; **B.** Habitus lateral, pereopods 1 and 3 broken, uropod missing; **C.** Antennae, flagellum broken; **D.** Head lateral. Scale bars: 0.5 mm (A, B), 0.2 mm (C), 0.3 mm (D).

**Figure 28.**
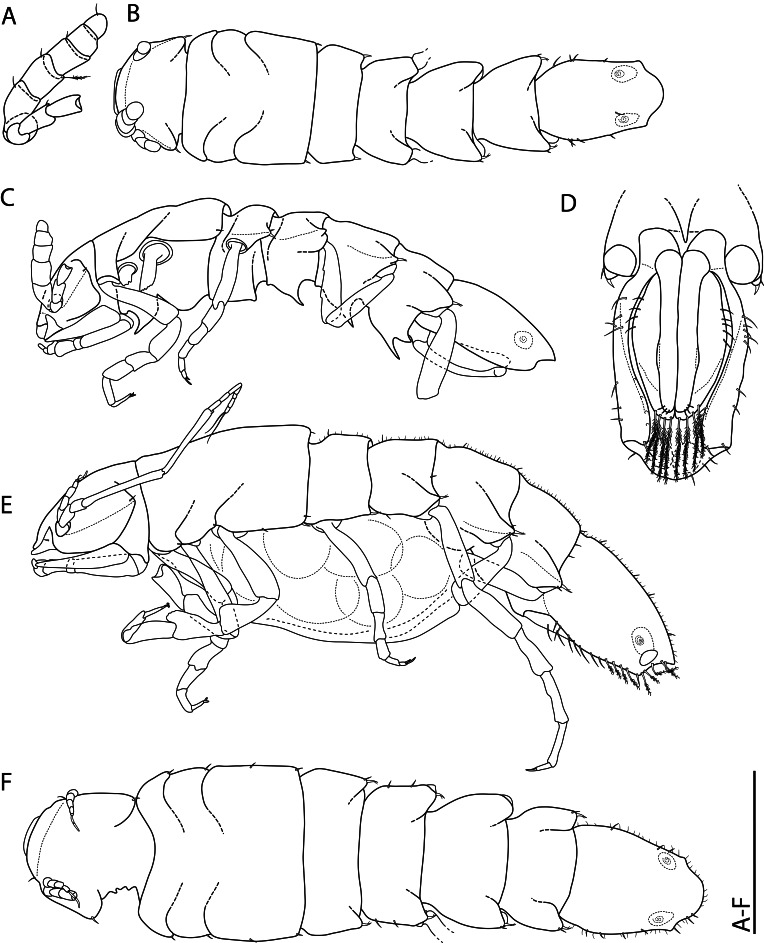
*Macrostylispapandreas* Johannsen, Riehl & Brandt, **sp. nov.** ovigerous female and subadult male. **A–D.** Paratype subadult male (ZMH K 45167), **E–F.** Paratype ovigerous female (ZMH K-45159). **A.** Antennula, antenna broken; **B.** Habitus dorsal, antenna broken, uropods missing; **C.** habitus lateral, antenna broken, pereopods 2–3, 4–7 broken; **D.** Pleotelson; **E.** Habitus lateral, pereopod 2 broken, pereopods 6–7 missing, uropod missing; **F.** Habitus dorsal, head damaged, uropods missing, antenna broken. Scale bars: 0.3 mm (A, D), 0.5 mm (B, C, E, F).

**Body** (Figs 25, 26, 27, 28) shape widest in anterior half, narrowing posteriorly; length 2.1–2.4 mm, 4.7–5.0 width, tergite surfaces with scattered setae, density of setation increasing from anterior to posterior tergites. **Ventral projections** present in ovigerous and non-ovigerous females, acute, on Prn1 prominent, orientated anteriorly; on Prn4 spine directed posteriorly, small, medially; on Prn5–Prn6 spine prominent, closer to posterior segment border; on Prn7 spine prominent. **Imbricate ornamentation** absent. **Ceph** length 0.76 width, 0.14 body length; frontal furrow proceeding slightly in front of A1–A2 insertions, originating from rim of A1–A2 sockets, slightly bent frontally; posterolateral setae present, flexibly articulated. **Fossosoma** length 1.0 width, 0.23 body length; ventral surface without carina, lateral tergite margins confluent. **Prn1** posterolaterally asetose. **Prn2**–**Prn3** posterolaterally setose. **Prn3** posterolateral setae robust, flexibly articulated, on pedestals. **Prn4** width 1.1 Prn5 width, length 0.38 width; with poorly-developed collum, anteriorly widest, with broadly rounded, blunt posterolateral margin; narrowing gradually towards posterior; posterolateral margins contracting laterally, rounded; posterolateral setae robust, spine-like, articulating on pedestals.

**Figure 29.**
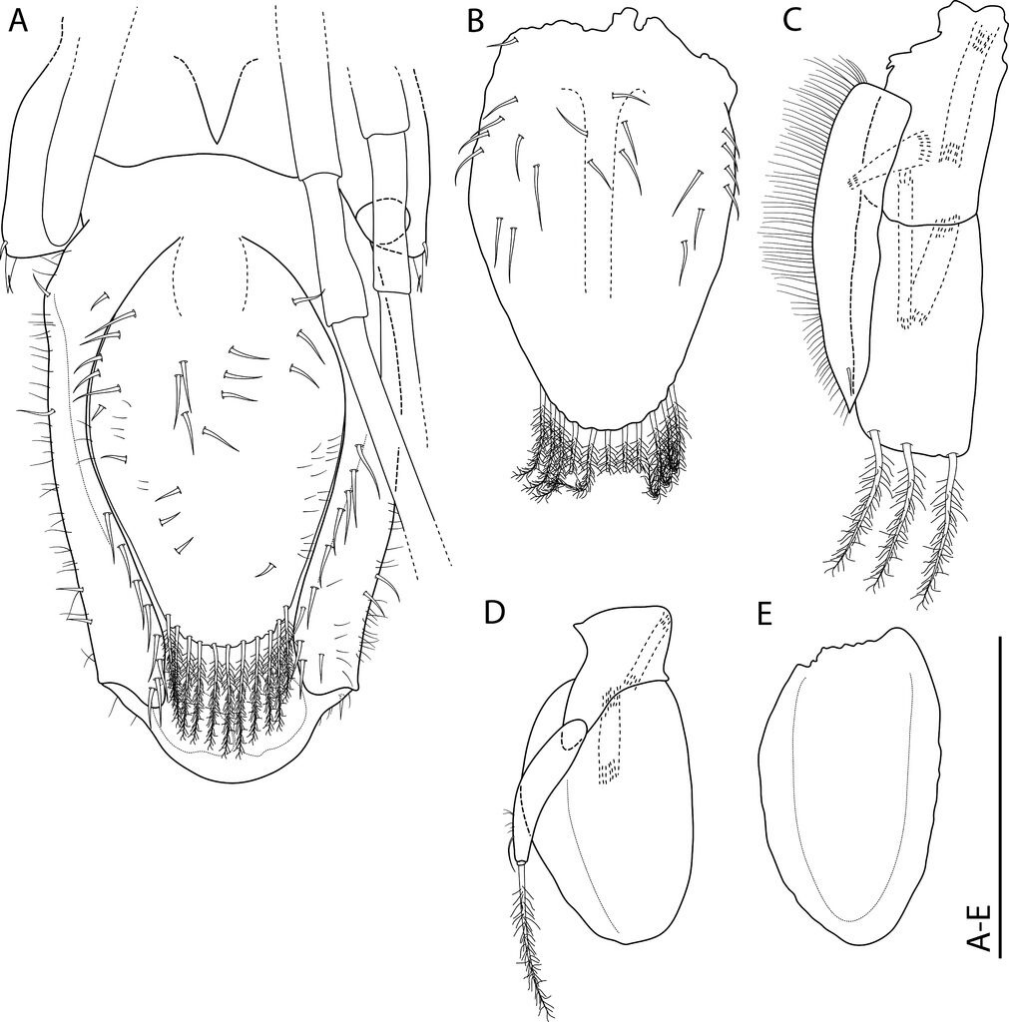
*Macrostylispapandreas* Johannsen, Riehl & Brandt, **sp. nov.** female pleotelson appendages. **A–E.** Paratype non-ovigerous female (ZMH K-45149). **A.** Pleotelson ventral; **B.** Operculum; **C.** Pleopod 3; **D.** Pleopod 4; **E.** Pleopod 5. Scale bars: 0.3 mm (A, B), 0.2 mm (C–E).

**Posterior tagma** posterolateral margins rounded; posterolateral setae in pairs, robust, spine-like. **Prn5** length 0.48 width, 1.2 Prn4 length; posterolateral setae on pedestals. **Prn6** length 0.70 width, 1.3 Prn 5 length; posterolateral margin projecting. **Prn7** with posterolateral projections, similar to Prn5–6, length 0.53 width. **Plt** (Figs 25, 26, 27, 28, 29), near-oval; length 0.22 body length, 1.6 width, narrower than Prn7; dorsal slot-like apertures diagonal across longitudinal axis, concave; posterior margin at Urp insertions concave, apex convex, broadly rounded, of semicircular shape, laterally extending to Urp insertions, apex length 0.19 Plt length; apical setae 8 altogether, positioned on and around apex; Plp cavity width 0.73 Plt width, setal ridges present, not visible in dorsal view; longitudinal trough width 0.36 Plt width; anal opening caudally in the trough, exposed and superficial, parallel to frontal plane.

**Figure 30.**
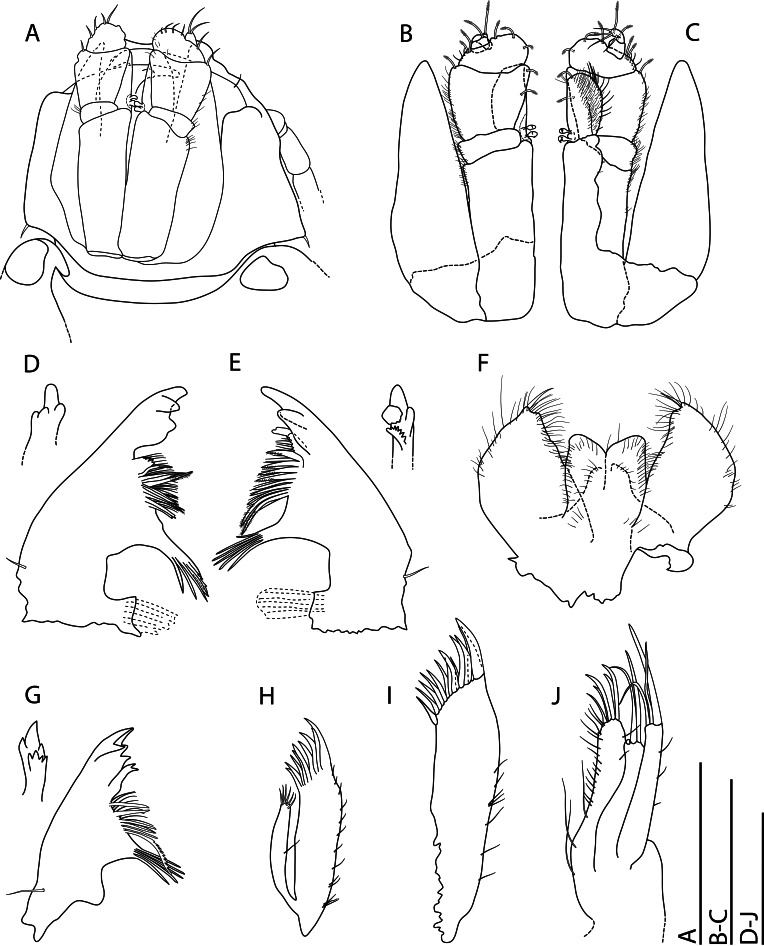
*Macrostylispapandreas* Johannsen, Riehl & Brandt, **sp. nov.** mouthparts. **A–F**, **I–J**: Paratype non-ovigerous female (ZMH K-45149). **G**–**H.** paratype adult male (ZMH K-45166). **A.** Head ventral; **B.** Maxilliped ventral; **C.** Maxilliped dorsal; **D.** Left mandible (female); **E.** Right mandible (female); **F.** Paragnaths; **G.** Left mandible (male); **H.** Maxillula (male); **I.** Maxillula (female), medial lobe broken; **J.** Maxilla. Scale bars: 0.3 mm (A), 0.2 mm (B, C), 0.1 mm (D–J).

**A1** (Figs 25, 26, 27) length 0.40 head width, 0.18 A2 length, width 1.0 A2 width; relative length ratios of art 1.0, 0.63, 0.38, 0.38, 0.13; L/W ratios of art 2.0, 1.7, 1.5, 1.5, 0.50; terminal art with 1 aesthetasc with intermediate belt of constrictions. **A2** (Figs 25, 26, 27) length 0.47 body length; coxa length shorter than width; basis length exceeding width, longer than twice coxa length; ischium length exceeding width, longer than coxa; merus longer than coxa, basis and ischium combined; carpus length subsimilar merus length; flagellum with 7 art. **Md** (Fig. 30) in medial view narrowest proximally to incisor; with lateral setae; molar process length subsimilar incisor length; left Md incisor process oligodentate with dorsal and ventral subdistal teeth that partly enclose lacinia, with 4 cusps, *lacinia mobilis* robust, cusp shape similar to incisor process, with 4 cusps; right Md incisor process oligodentate with dorsal and ventral subdistal cusps that partly enclose lacinia, with 3 cusps, *lacinia mobilis* robust, construction similar to incisor process, larger than left *lacinia mobilis*, with 7 cusps. **Mxp** (Fig. 30) basis length 2.2 width; medioventrally setose, distally with setulate sensillae; palp art2 wider than art1, art1 shorter than art3; epipod length 2.0 width, 0.40 coxa-basis length.

**Figure 31.**
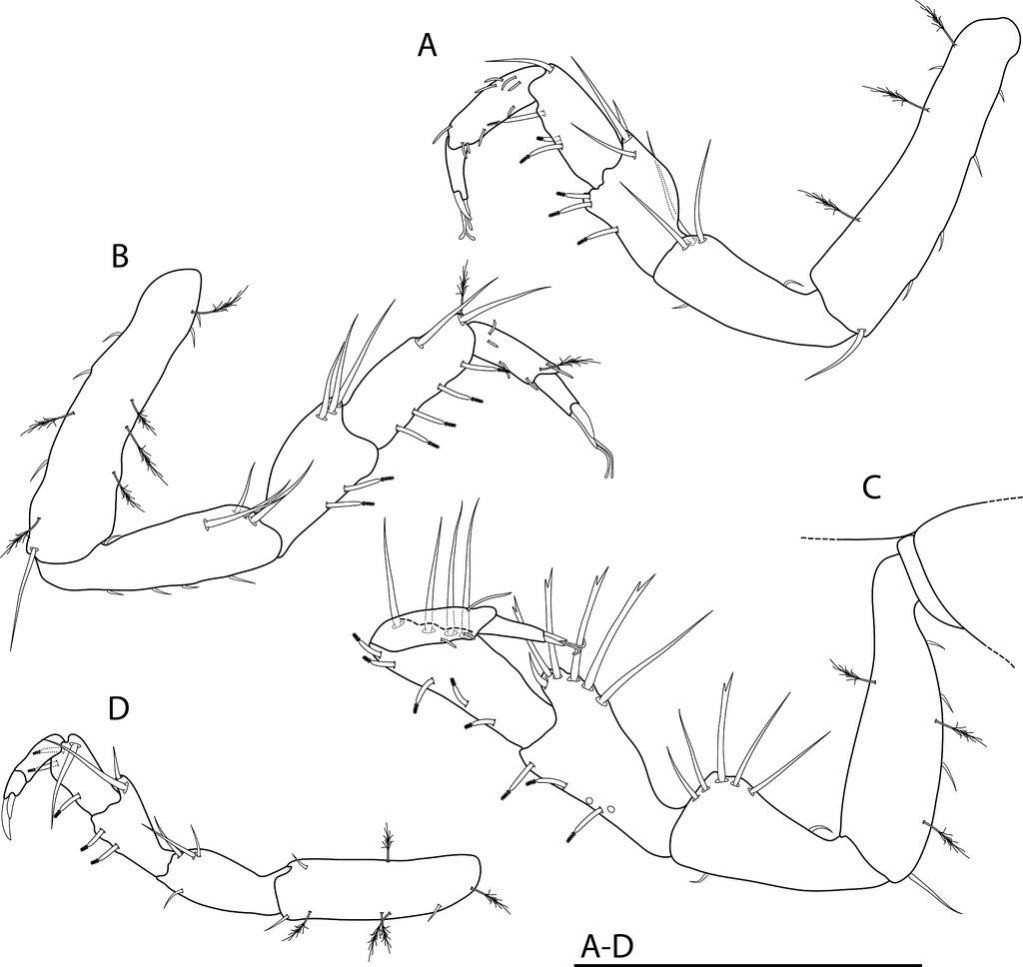
*Macrostylispapandreas* Johannsen, Riehl & Brandt, **sp. nov.** female anterior pereopods. **A–D.** Paratype ovigerous female (ZMH K-45159). **A.** Pereopod 1; **B.** Pereopod 2; **C.** Pereopod 3; **D.** Pereopod 4. Scale bars: 0.3 mm (A–D).

**P1** (Fig. 31) length 0.37 body length; ischium dorsal margin with 4 setae; merus dorsal margin with 3 setae, ventral margin with 3 bisetulate setae; carpus dorsally with 1 seta; dactylus medially-subdistally with 3 sensillae, terminal claw length 0.60 dactylus length. **P2** (Fig. 31) length 0.39 body length; ischium dorsally with 5 setae submarginally; merus dorsally with 3 setae, ventrally with 2 bisetulate setae; carpus dorsally with 1 asetulate seta, 1 broom seta, 1 bifid seta, ventrally with 4 bisetulate setae; dactylus medially-subdistally with 2 sensillae.

**Figure 32.**
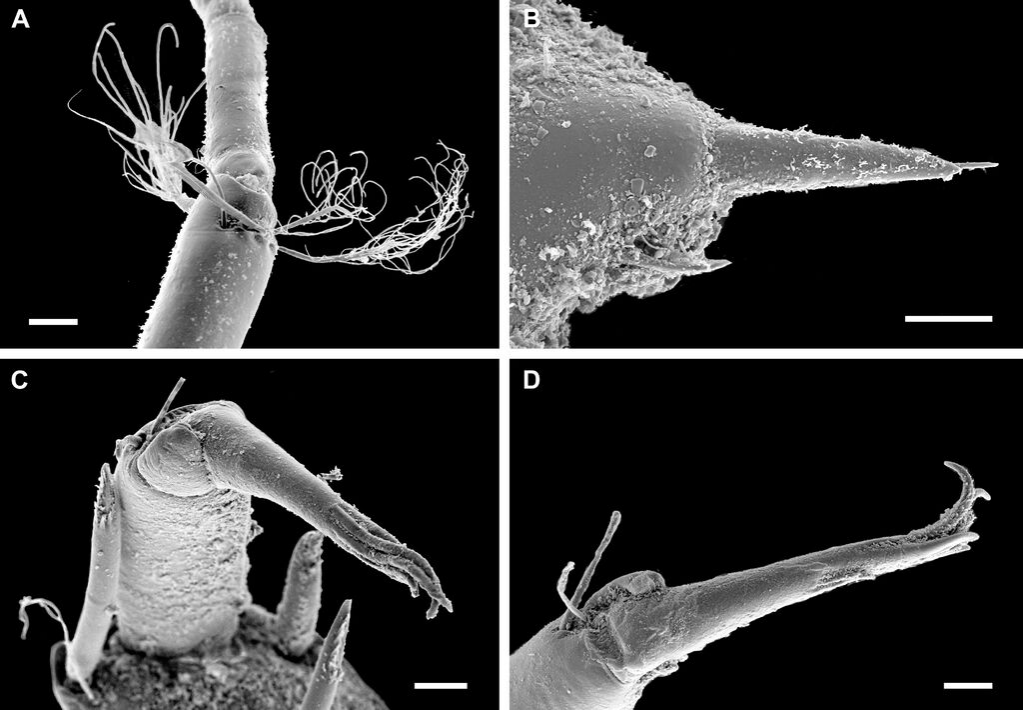
*Macrostylispapandreas* Johannsen, Riehl & Brandt, **sp. nov.** SEM images of setae. **A.** Paratype non-ovigerous female (ZMH K-45154), **B–D.** Paratype adult male (ZMH K-45165). **A.** Antennal distal broom setae; **B.** Pereonite 5 posterolateral setae; **C.** Pereopod 2 dactylus; **D.** Pereopod 3, dactylus. Scale bars: 10 µm (A–D).

**Figure 33.**
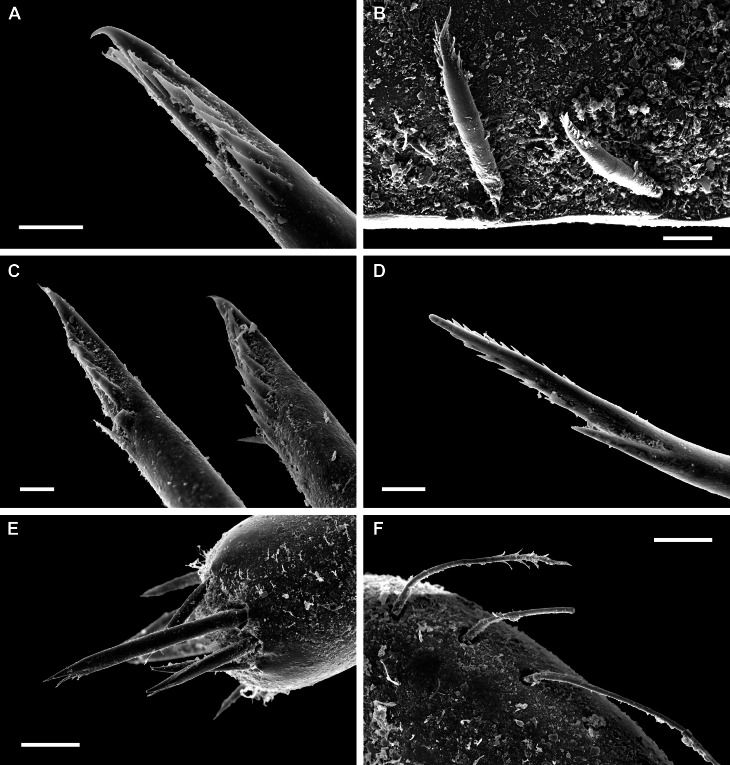
*Macrostylispapandreas* Johannsen, Riehl & Brandt, **sp. nov.** SEM images of setae. **A, C, E–F.** Paratype adult male (ZMH K-45168), **B.** Paratype adult male (ZMH K-45165), **D.** Paratype non-ovigerous female (ZMH K-45154). **A.** Pereopod 2 carpus distolateral seta tip; **B.** Pereopod 3 merus ventral setae; **C.** Pereopod 3 merus dorsal setae tips; **D.** Pereopod 3 carpus dorsal setae; **E.** Pereopod 4 merus distoventral setae; **F.** Pereopod 7 basis posterior dorsal setae. Scale bars: 4 µm (A, D), 10 µm (B, F), 3 µm (C), 20 µm (E).

**Figure 34.**
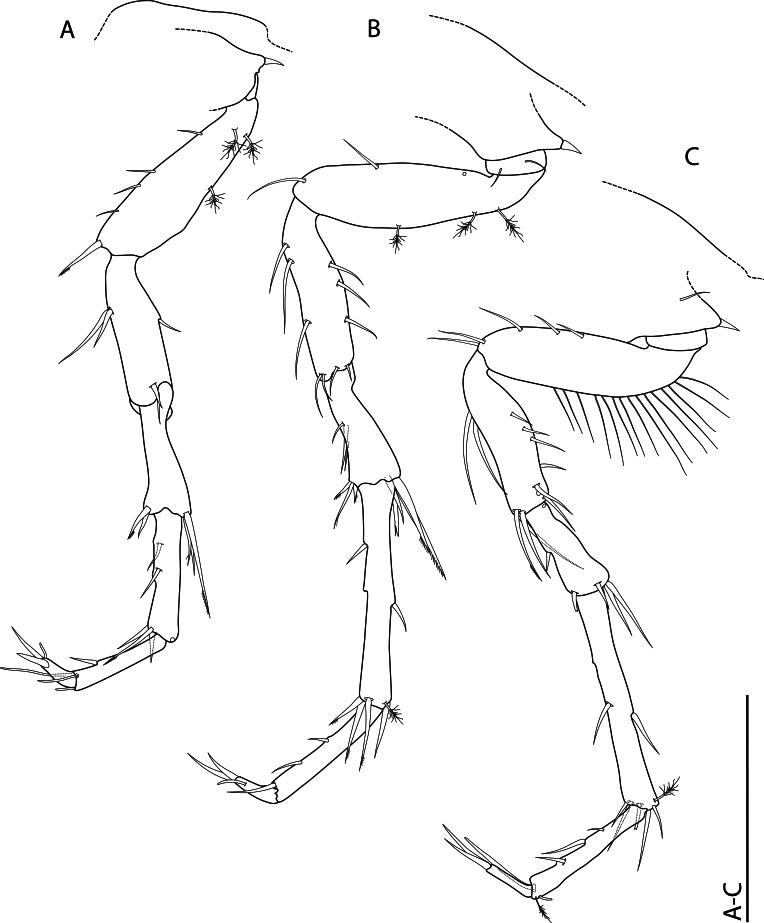
*Macrostylispapandreas* Johannsen, Riehl & Brandt, **sp. nov.** female posterior pereopods. **A–C.** Paratype non-ovigerous female (ZMH K 45149). **A.** Pereopod 5; **B.** Pereopod 6; **C.** Pereopod 7. Scale bars: 0.3 mm (A–C).

**P3** (Figs 31, 32, 33) length 0.42 body length; ischium dorsal lobe subtriangular; proximally with 2 setulate setae, apex with 1 prominent seta; apical seta simple, bifurcate, straight, flexibly articulated; distally with 2 setulate setae; merus dorsally with 6 bifurcate setae, ventrally with 5 bisetulate setae; carpus dorsally with 4 setae, ventrally with 5 bisetulate setae; dactylus medially-subdistally with 3 sensillae. **P4** (Fig. 31) length 0.23 body length; carpus oval in cross section. **P5** (Fig. 34) length 0.40 body length; art L/W ratios 3.1, 3.3, 2.3, 4.3, 4.3, 2.5; relative art length ratios 1.0, 0.80, 0.56, 0.68, 0.52, 0.20. **P6** (Fig. 34) length 0.51 body length; art L/W ratios 3.1, 3.7, 2.3, 7.5, 5.7, 3.0; relative art length ratios 1.0, 0.79, 0.57, 1.1, 0.61, 0.21. **P7** (Fig. 34) 0.48 body length; relative art length ratios 1.0, 0.84, 0.56, 1.2, 0.72, 0.24; basis length 3.1 width, dorsal margin with row of 15 elongate setae, exceeding beyond proximal half of art, setae longer basis width, ventral margin with row of 3 elongate setae, setae shorter basis width; ischium length 3.5 width, mediodorsally with 4 setae, medioventrally with 2 setae, grouped, distoventrally with 3 setae; merus length 2.3 width, distodorsally with 3 setae, medioventrally and distoventrally with 1 seta. Carpus length 7.3 width, mediodorsally with 1 seta, distodorsally with 3 setae, medioventrally with 2 setae, distoventrally with 3 setae; propodus length 6.0 width, dactylus length 3.0 width.

**Op** (Fig. 29) stout; length 1.5 width, 0.70 Plt dorsal length, not reaching anus; apical width 0.44 Op width; distally tapered; distal margin narrow, broadly rounded; ventrally with rounded, edgeless keel; with lateral fringe consisting of 4 setae on either side, separate from apical row of 11 broom setae; apical setae short, extending to anal opening. **Plp3** (Fig. 29) protopod length 2.0 width, 0.50 Plp3 length, endopod plumose setae shorter than endopod; exopod length 0.70 Plp3 length, monoarticulate, with one conspicuous subapical seta. **Plp4** (Fig. 29) length 0.81 width, endopod length 1.5 width; exopod length 4.5 width, 0.53 endopod length, lateral fringe of setae present. **Urp** (Fig. 25) length greater than pleotelson length; protopod length 13 width, 0.80 Plt length, cylindrical, distal margin blunt, endopod insertion terminally; endopod broken off and missing.

**Description of male** (where different from female)

**Figure 35.**
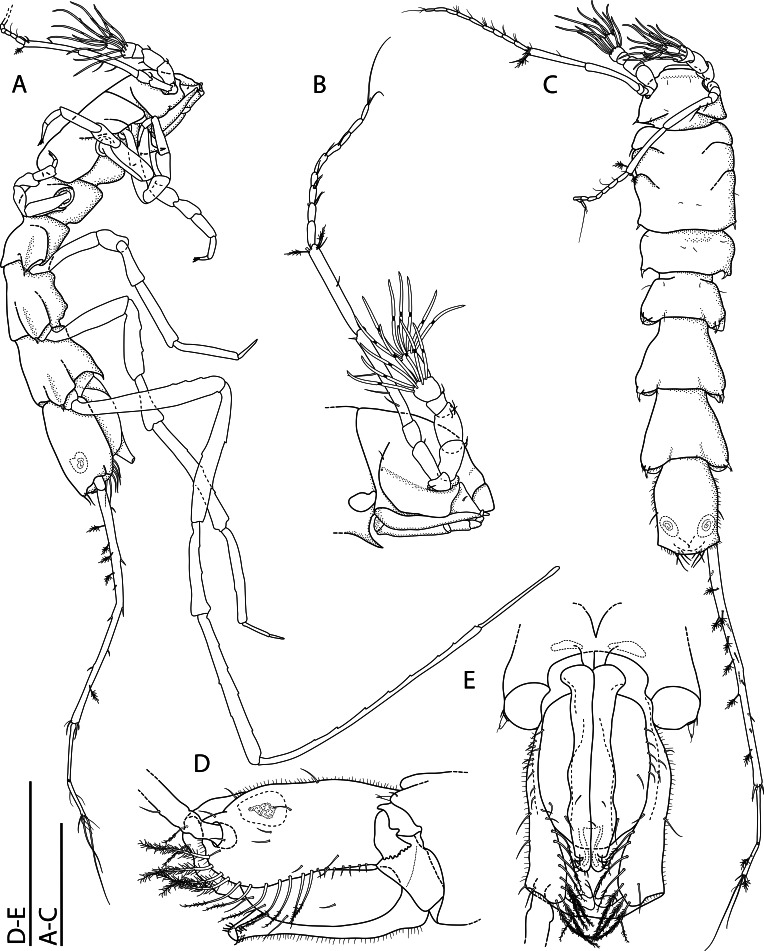
*Macrostylispapandreas* Johannsen, Riehl & Brandt, **sp. nov.** male habitus, head and pleotelson. **A–E.** Paratype adult male (ZMH K 45166). **A.** Habitus lateral; **B.** Head lateral; **C.** Habitus dorsal; **D.** Pleotelson lateral; **E.** Pleotelson ventral. Scale bars: 0.5 mm (A, C), 0.3 mm (B, D, E).

**Figure 36.**
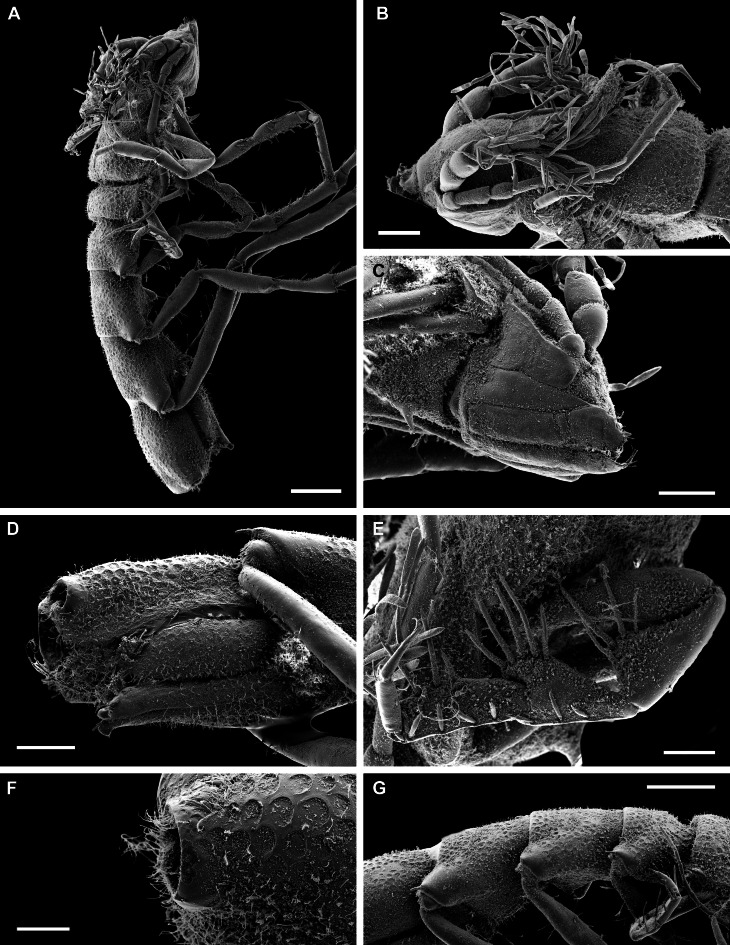
*Macrostylispapandreas* Johannsen, Riehl & Brandt, **sp. nov.** SEM images of adult male. **A–G.** Paratype adult male (ZMH K-45165). **A.** Habitus lateral; **B.** Head and antennae dorsolateral; **C.** Head ventrolateral; **D.** Pleotelson ventrolateral; **E.** Pereopod III; **F.** Uropod articulation; **G.** Ventral spines pereonites 5–7. Scale bars: 200 µm (A, G), 100 µm (B–D), 60 µm (E), 40 µm (F).

**Body** (Figs 35, 36) more elongate than female, length 2.0 mm, 5.4 width. **Ventral projections** absent on Prn 3–Prn5; on Prn6 projection blunt, small, located closer to posterior segment border, on Prn7 projection spinifom, prominent. **Imbricate ornamentation** absent from Ceph and Prn1–Prn2; covering entire tergites, except posterolateral projections of Prn3–Prn7 and Plt. **Ceph** (Figs 35, 36) length 0.73 width, 0.14 body length; frontal furrow present, straight; posterolateral setae present. **Prn3** posterolateral margin not projecting posteriorly; with 1 posterolateral seta, seta sensillate, robust, flexibly articulated on pedestals. **Posterolateral setae** on Prn4-Prn7 robust, sensillate, spine-like, articulating on pedestals. **Prn4** length 0.45 width; integration with other segments distinct from both anterior and posterior Prn: with well-developed collum, widest medially, relatively small posterolateral projections; lateral margins sinusoid, convex in anterior half, narrowing posteriorly towards posterolaterally orientated projections; posterolateral margins projecting posteriorly, tapering. **Prn5** length 0.56 width. **Prn6** length 0.81 width, 1.5 Prn 5 length. **Plt** (Figs 35, 36) subrectangular, waist present, width maximum both anteriorly and posteriorly to waist; width smaller than Prn7 width; posterior margin at Urp insertions straight to convex; posterior apex convex, very flat curvature between Urp insertions; length 0.08 Plt length; Plp cavity width 0.77 Plt width, longitudinal trough width 0.35 Plt width.

**Figure 37.**
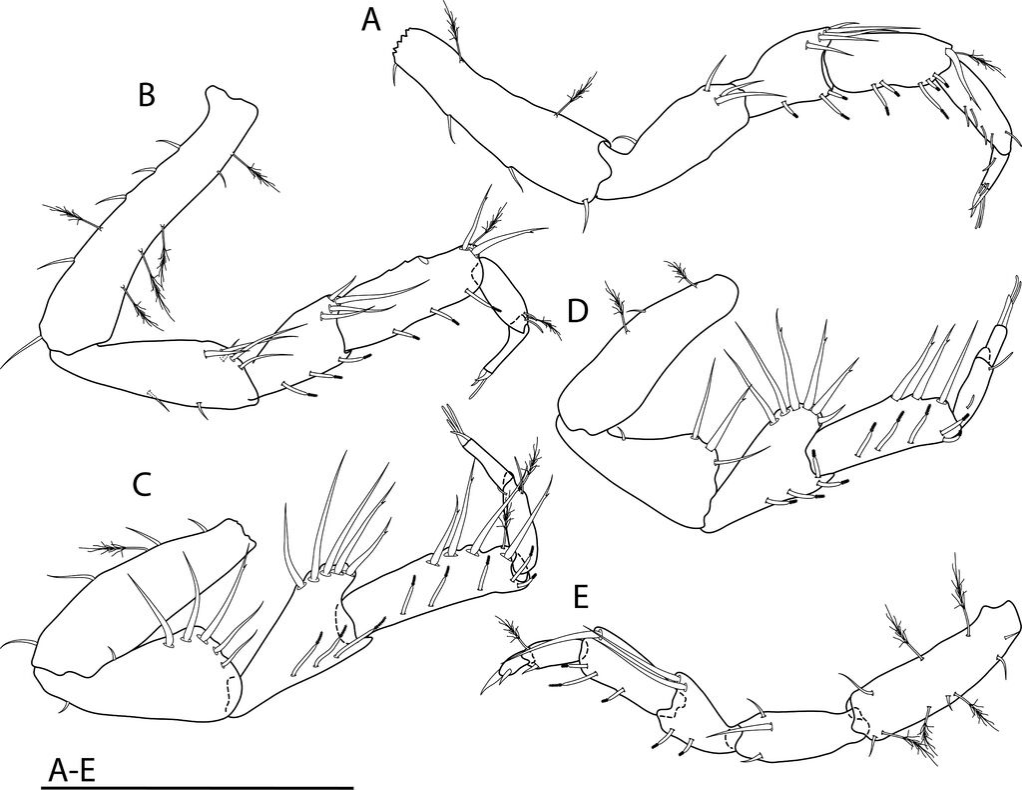
*Macrostylispapandreas* Johannsen, Riehl & Brandt, **sp. nov.** male anterior pereopods. **A–C**, **E.** Paratype adult male (ZMH K-45166), **D.** Paratype subadult male (ZMH K-45167). **A.** Pereopod 1; **B.** Pereopod 2; **C.** Pereopod 3 (adult male); **D.** Pereopod 3 (subadult male); **E.** Pereopod 4. Scale bars: 0.3 mm (A–E).

**A1** (Figs 35, 36) length 0.95 Ceph width, 0.33 A2 length, width 1.2 A2 width; art L/W ratios 1.7, 1.8, 1.0, 1.0, 2.0; relative art length ratios 1.0, 1.1, 0.50, 0.60, 0.80; art1, art2 and art5 elongate tubular; art3 and art4 squat or noticeably shorter; terminal and penultimate art with 7 aesthetascs each; aesthetasc length subsimilar A1 length or shorter; art 2 length subsimilar art1 length; art5 shorter than art1. **A2** (Figs 35, 36) length 0.54 body length; flagellum of 9 art; coxa squat; basis elongate, widening distally, longer than coxa; ischium squat, cylindrical, longer than coxa; merus longer than coxa, basis and ischium together; carpus shorter than merus. **Prp1** (Fig. 37) length 0.12 body length; art L/W ratios 3.3, 2.7, 1.1, 2.0, 3.3, 3.5; relative art length ratios 1.0, 0.70, 0.35, 0.52, 0.43, 0.30. **Prp2** (Fig. 37) length 0.45 body length; art L/W ratios 4.1, 2.8, 1.7, 2.5, 2.7, 4.5; relative art length ratios 1.0, 0.59, 0.34, 0.52, 0.28, 0.31. **Prp3** (Figs 33, 36, 37) length 0.44 body length; art L/W ratios 2.9, 1.9, 1.4, 3.2, 3.3, 4.5; relative art length ratios 1.0, 0.74, 0.57, 0.70, 0.43, 0.39. **Prp4** (Fig. 37) length 0.30 body length; art L/W ratios 3.8, 2.4, 1.2, 2.2, 3.0, 6.0; relative art length ratios 1.0, 0.63, 0.37, 0.58, 0.32, 0.32.

**Figure 38.**
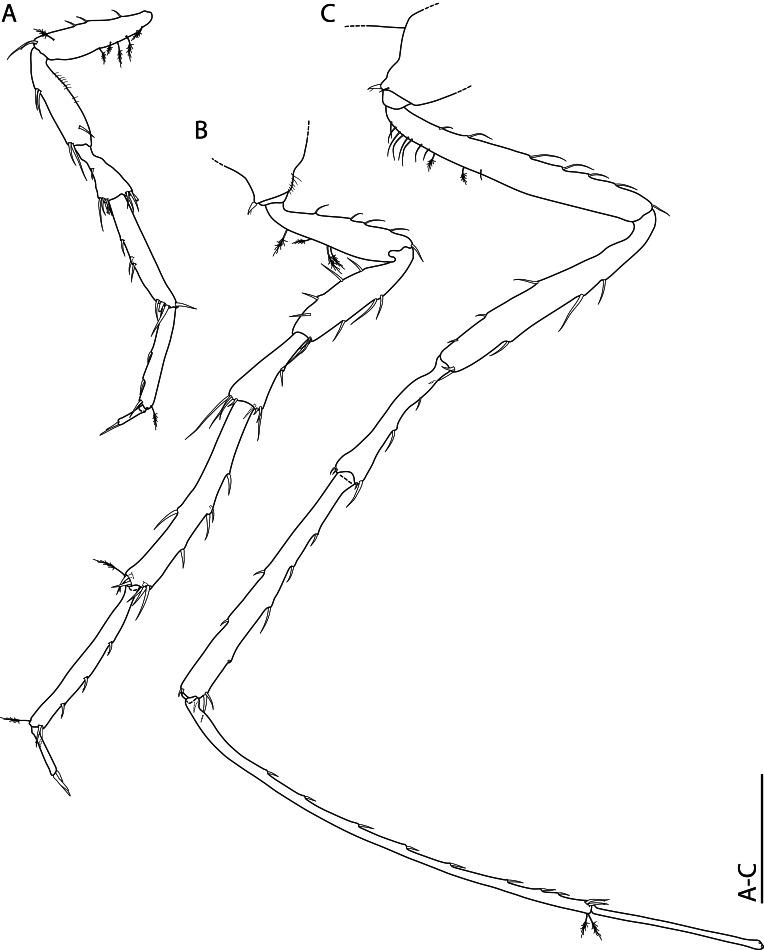
*Macrostylispapandreas* Johannsen, Riehl & Brandt, **sp. nov.** male posterior pereopods. **A–C.** Paratype adult male (ZMH K-45166). **A.** Pereopod 5; **B.** Pereopod 6; **C.** Pereopod 7. Scale bars: 0.3 mm (A–C).

**Prp5** (Fig. 38) 0.63 body length; art L/W ratios 3.9, 3.2, 1.8, 5.8, 6.0, 3.5, relative art length ratios 1.0, 0.85, 0.52, 1.1, 0.89, 0.26. **Prp6** (Fig. 38) length/body-length ratio distinctly elongate, length 0.91 body length; art L/W ratios 3.9, 3.8, 2.3, 7.0, 8.0, 5.5; relative art length ratios 1.0, 0.97, 0.68, 1.6, 1.3, 0.35. **Prp7** (Fig. 38) length/body-length ratio sexually dimorphic, distinctly longer than in female, length 1.8 body length; relative art length ratios 1.0, 0.90, 0.53, 1.0, 1.7, 0.64; basis length 8.0 width, dorsal margin with row of 8 simple setae proximally, 2 broom setae, medially submarginally with 1 simple seta, ventral margin with row of 7 simple setae, setae shorter than basis width, row distributed along margin from near-proximally to distally; ischium length 6.3 width, mediodorsally and medioventrally with 3 setae respectively, distoventrally with 2 setae; merus length 4.6 width, distodorsally with 2 setae, medioventrally with 2 setae, distoventrally with 2 setae; carpus length 11 width, mediodorsally with 2 setae, distodorsally with 2 setae, medioventrally with 4 setae, distoventrally with 3 setae; propodus length 35 width; dactylus length 41 width.

**Figure 39.**
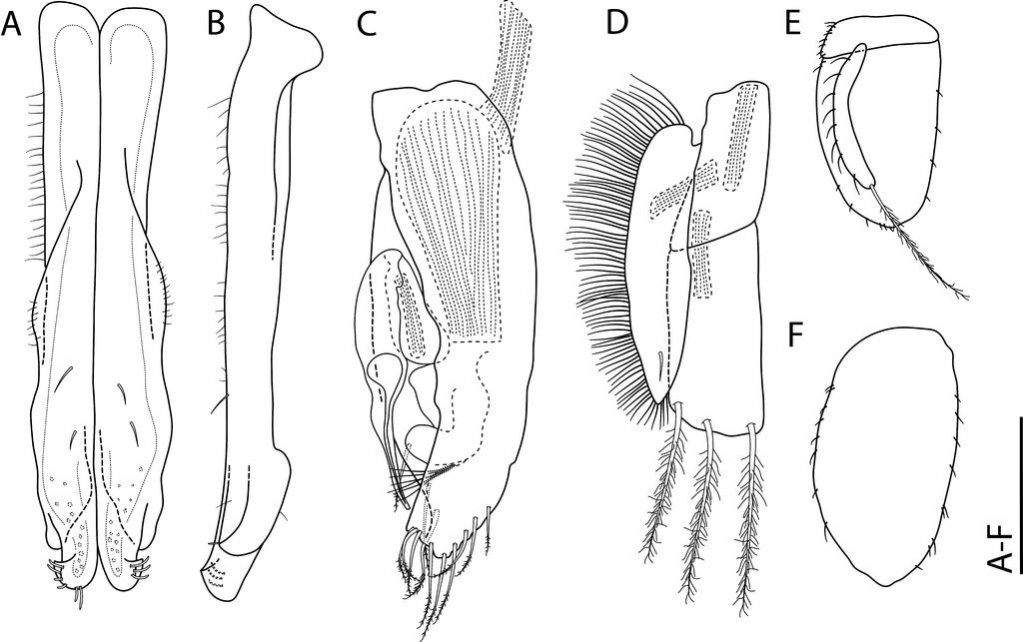
*Macrostylispapandreas* Johannsen, Riehl & Brandt, **sp. nov.** male pleopods. **A–F.** Paratype adult male (ZMH K-45166). **A.** Pleopods 1 ventral; **B.** Pleopod 1 lateral; **C.** Pleopod 2; **D.** Pleopod 3; **E.** Pleopod 4; **F.** Pleopod 5. Scale bars: 0.1 mm (A–F).

**Plp.** (Fig. 39) Male Op vaulted. **Plp1** length 0.95 Plt length, shorter Plp2, with the latter projecting beyond Plp1, lateral lobes not projecting, medial lobes projecting distally forming hook-like distolateral processes; medial lobes distally with 3–6 sensillae, ventrally with 2 setae, respectively; distally curved, projecting ventrally beyond Plp2 ventral margin. **Plp2** protopod apex tapering, distally enclosing Plp1 and converging towards counterpart, with 3 setae on distolateral margin and 9 pappose setae distally; endopod distance of insertion from protopod distal margin 0.30 protopod length; stylet length 0.53 protopod length, quasi-straight, extending to near distal margin of protopod. **Urp** (Fig. 35) length 3.5 Plt length; protopod length 25 width, 1.5 Plt length or longer; endopod length 0.37 protopod length, 37.0 width, width smaller protopod width.

#### Diagnosis

With significant sexual dimorphism, mostly affecting body length-width ratio, posterior pereopod length and antennula. *Females and juvenile males*: Body widest in anterior half, narrowing posteriorly, elongate, subcylindrical in cross section; tergite and sternite surfaces setose. Ventral projections spiniform on Prn1 and Prn4, absent in Prn2–Prn3. Fossosoma without carina, tergal plates laterally merged seamlessly with sternites, lateral tergite margins confluent. Prn3 posterolateral margins not projecting, posterolateral setae articulating on pedestals, posterior margin smooth. Prn4 with poorly-developed collum, anteriorly widest, with broadly rounded, blunt posterolateral margin; lateral margins anteriorly widest, narrowing gradually towards posterior. Prn5–Prn7 posterolateral margins projecting, rounded. Pln1 tergal articulation with Plt absent. Plt narrower than Prn7, near-oval, without waist, apex convex. A1 of 5 art decreasing in size from proximal to distal, art1 longer than wide, terminal art minute. A2 coxa length subsimilar width, ischium length exceeding width, longer than coxa. A2 flagellum of 7 art. P3 ischium subtriangular. P7 length subsimilar to P6 length. Op stout, distally tapered, not reaching anus, apical width subsimilar or smaller, 0.50 operculum width. Urp length longer Plt length; protopod length 0.80 Plt length, cylindrical, distal margin blunt, endopod insertion terminally, endopod monoarticulate. *Differences in adult males*: Body more elongate than female (L/W ratio 4.7–5.0 in female, 5.4 in male). Plt shape subrectangular with waist, width maximum both anteriorly and posteriorly to waist, posterior margin at uropod insertions straight to convex, apex convex. A1 art1, art2 and art5 elongate tubular; art3 and art4 squat or noticeably shorter, terminal and subterminal art with several long aesthetascs. A2 art not significantly hypertrophied compared to A1. Male operculum vaulted; Plp1 ca. 0.95 Plt length, lateral lobes not projecting, medial lobes projecting distally forming hook-like distolateral processes, subdistally with even ventral surface, distally projecting ventrally beyond Plp2 ventral margin. Plp2 distally projecting beyond Plp1, protopod apex tapering, distally enclosing Plp1 and converging towards counterpart.

**Molecular Diagnosis**: On the basis of the mtDNA 16S rRNA gene, this species can be distinguished from other *Macrostylis* species by the following unique asymmetric nucleotide combinations: position: TCTAAAAGTTTAGAAT (162-177), AAAATTAGA (184-192) and CTCTTTAGAATAGAGA (231-245).

#### Etymology

The specific epithet "*papandreas*" is a noun in apposition and was the nickname of the late Andreas Heitland, father of Nele Johannsen, whose memory is honoured by this eponym.

#### Distribution

Tropical North Atlantic, eastern and western Vema Fracture Zone, abyssal depths of 5,002.5 m to 5,119 m.

#### Taxon discussion

*Macrostylispapandreas* Johannsen, Brandt & Riehl, **sp. nov.** represents the first species of the family from the Atlantic Ocean for which adult males with "extremely" elongate posterior pereopods and other aberrant character states (see diagnosis) could be linked to a female by means of DNA sequence data. Sexually dimorphic character states have been discussed in detail for species with a much lower degree of differences between the sexes (Riehl et al. 2012). After previous reports from the Northwest Pacific (Bober et al. 2017) which was the first location to shed light on this "extreme" sexual dimorphism, this description and molecular characterisation of *M.papandreas* **sp. nov.** brings us one step closer to the identification of female counterparts of the "weird" male forms represented, for instance, by *M.longipes* Hansen, 1916 and *M.longipedis* Brandt, 2004 (see Hansen (1916), Brandt (2004)).

#### Notes

##### Methods

**Sampling**: *Macrostylispapandreas*, **sp. nov.**, was discovered during the Vema‑TRANSIT expedition (SO237, R/V Sonne, Dez/Jan 2014/15, Atlantic Ocean (Devey et al. 2015)).

**Sample treatment and DNA sequencing**: The samples were collected using a camera-epibenthic sledge (C-EBS) (Brandt et al. 2012) and treated following the protocol by Riehl et al. (2014b) to fixate and extract benthic macrofauna for molecular and morphological studies. Tissue samples were processed by LGC Genomics (Berlin) and previously published by Riehl et al. (2018).

**Species description**: Five individuals of *M.papandreas* **sp. nov.** were transferred into glycerine to prepare temporary slides for taxonomic line drawings following Wilson (2008). The holotype was used for the illustration of the habitus. Paratype ZMH K-45149 was dissected for illustration of the habitus and appendages. Paratype ZMH K-45166 and non-type ZMH K-45159 were dissected for illustration of the habitus and appendages. Paratype ZMH K-45167 was used for illustration of the habitus. Ovigerous female, 2.1 mm, ZMH K-45159, from Vema-TRANSIT expedition (SO-237) station 11–1 was dissected for illustration of the habitus and appendages. Pencil drawings were prepared on a Leica Dialux compound microscope equipped with camera lucida and a custom-made LED lighting system by BW Optik (Aschendorf). Calibrations were done with the help of a stage micrometer (1.0 mm x 0.01 mm). All drawings were scanned as greyscale PDF and traced using a digital drawing board (Wacom Intuos 4) and the vector-graphic software Adobe^®^ Illustrator^®^ CS 6 (Coleman 2003, Coleman 2009). To improve the impression of depth and structure in the line drawings, digital stippling was added to selected illustrations (Bober and Riehl 2014). Measurements were taken from the digitalised drawings with the measuring tool in Adobe^®^ Acrobat^®^ XI Pro following the standards of Hessler (1970).

For SEM, four individuals of *M.papandreas* were critical-point dried and sputter-coated with graphite; a Zeiss LEO 1525 was used. Where several scans were required to illustrate a specimen, total-projection images were merged using the Microsoft Image Composite Editor version 2.0.3.0 (64 bit). Figures of line drawings and SEM pictures were edited using Adobe^®^ Photoshop^®^ CS6 and Adobe^®^ Illustrator^®^ CS6.

Morphological characters were conceptualised and character states were scored in an updated DELTA (Dallwitz 1980, Dallwitz 1993, Dallwitz 2010) database for Macrostylidae (Riehl et al. 2012, Riehl and De Smet 2020) which is publicly available via the Zenodo repository (Riehl 2024b). Description texts and diagnoses were generated from this database. Terminology and structure of each description follows previous work on Macrostylidae (Riehl et al. 2012, Riehl et al. 2014a, Riehl 2014). The articles of the antennae are named according to Hansen (1893).

From all types and other material, selected posterior pereopods from one side of the body were dissected for DNA extraction. For the molecular diagnosis, all nucleotide sequences for Macrostylidae were downloaded from GenBank (Benson et al. 2012). These included the mtDNA 16S mRNA previously published for *Macrostylispapandreas* **sp. nov.** (Riehl et al. 2018), the only sequences available for this species to date. A multiple sequence alignment was constructed using MAFFT (Katoh et al. 2009) implemented in Geneious (Kearse et al. 2012) with default settings. The alignment is publicly available via the Zenodo repository (Riehl 2024a). The molecular diagnosis was prepared using the online tool DeSigNate (Hütter et al. 2020). Under consideration of single nucleotides, no position of the alignment reached a discriminative power of 1.0 between the query group (*Macrostylispapandreas* **sp. nov.**) and the reference group (all other macrostylid species). Consequently, neighbouring noisy positions were manually combined until a combined asymmetric candidate character (those strings of homologous nucleotides of the reference group that are not uniform, but different from the character state in the query group) was obtained.

Specimens were deposited at the Leibniz Institute for Biodiversity Change Analysis (LIB), Museum of Nature Hamburg, Germany, using the collection code “ZMH”.

### Austroniscus indobathyasellus

Kaiser, Kniesz & Kihara sp. nov.

0221DC51-D934-584D-8389-445353CD625E

0E7541DA-9DFF-4C49-B8FE-AED5C76CCBB3

#### Materials

- **Type status:** Holotype. **Occurrence:** catalogNumber: SMF 61327; recordedBy: K. Kniesz & T.C. Kihara; individualCount: 1; sex: male; lifeStage: adult; preparations: 96% EtOH; associatedSequences: http://www.ncbi.nlm.nih.gov/nuccore/OR825323; occurrenceID: 9C41CDEC-C376-5815-8ACD-49581FB40DDD; **Taxon:** scientificName: *Austroniscusindobathyasellus* Kaiser, Kniesz & Kihara; kingdom: Animalia; phylum: Arthropoda; class: Malacostraca; order: Isopoda; family: Nannoniscidae; genus: Austroniscus; specificEpithet: *indobathyasellus*; taxonRank: species; scientificNameAuthorship: Kaiser, Kniesz & Kihara; nomenclaturalCode: ICZN; **Location:** higherGeography: Indian Ocean; waterBody: Central Indian Ocean Ridge; locality: Station No. I19_036RO_BB_01; verbatimDepth: 2628 m; locationRemarks: 150 m distance of a hydrothermal vent field; verbatimLatitude: 25°28'S; verbatimLongitude: 69°55'E; decimalLatitude: -25.466675; decimalLongitude: 69.916682; **Identification:** identifiedBy: S. Kaiser, K. Kniesz, T. Kihara; dateIdentified: 05/2022; **Event:** samplingProtocol: Rock picking by Remotely Operated Vehicle, rock washing over sieves on deck; eventDate: 14/11/2019; **Record Level:** institutionCode: SMF; basisOfRecord: PreservedSpecimen

#### Description

**Figure 40.**
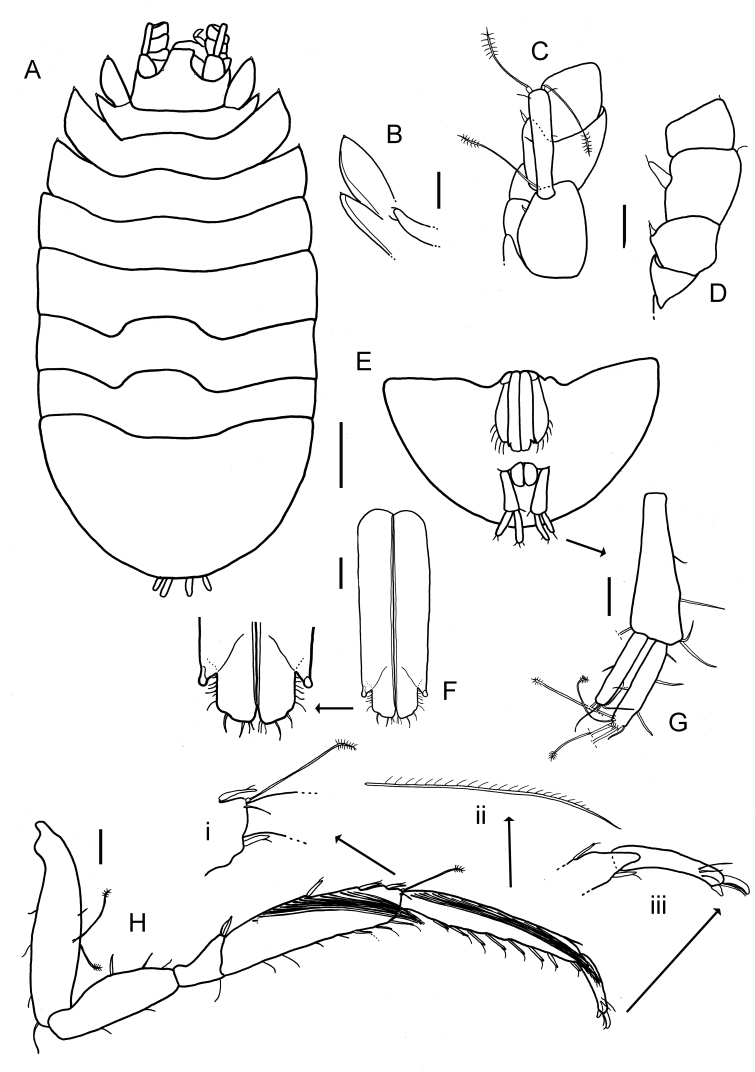
*Austroniscusindobathyasellus* Kaiser, Kniesz & Kihara, **sp. nov.**, holotype male (SMF 61327). **A.** Habitus, dorsal view; **B.** Pereonite 1 coxal extension, ventral view; **C.** Antenna 1 articles 1–2 and antenna 2 articles 1–4, drawn *in situ*; **D.** Right antenna 1, articles 1–4, ventral view, drawn *in situ*; **E.** Pleotelson, ventral view; **F.** Pleopod 1, detail: distal margin, drawn *in situ*; **G.** Uropod, drawn *in situ*; **H.** Pereopod 5, details: **i)** carpus distal margin, **ii)** setulae on natatory seta, **iii)** dactylus and unguis. Scale bars: 500 µm (A, E), 100 µm (B–D, F–H).

**Figure 41.**
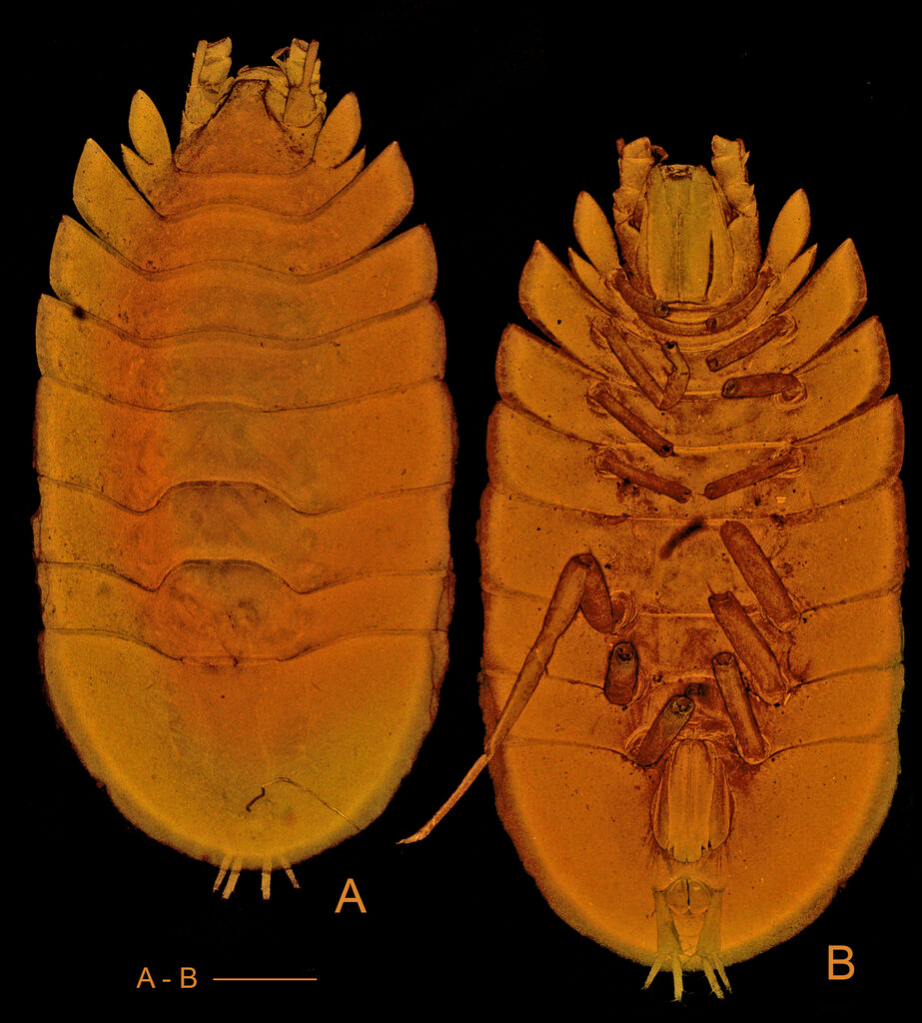
*Austroniscusindobathyasellus* Kaiser, Kniesz & Kihara, **sp. nov.**, holotype male (SMF 61327), confocal laser scanning microscopy images. **A.** Habitus, dorsal view; **B.** Habitus, ventral view. Scale bar: 500 µm.

**Figure 42.**
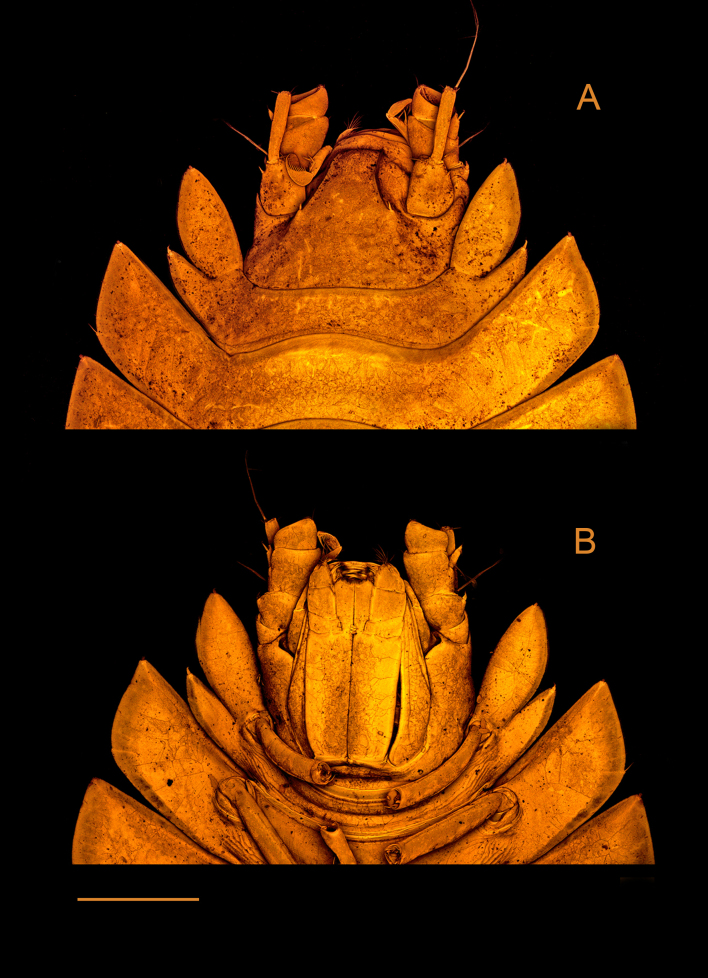
*Austroniscusindobathyasellus* Kaiser, Kniesz & Kihara, **sp. nov.**, holotype male (SMF 61327), confocal laser scanning microscopy images. **A.** Cephalothorax and pereonite 1 and 2, dorsal view; **B.** Cephalothorax and pereonite 1 and 2, ventral view. Scale bar: 400 µm.

**Figure 43.**
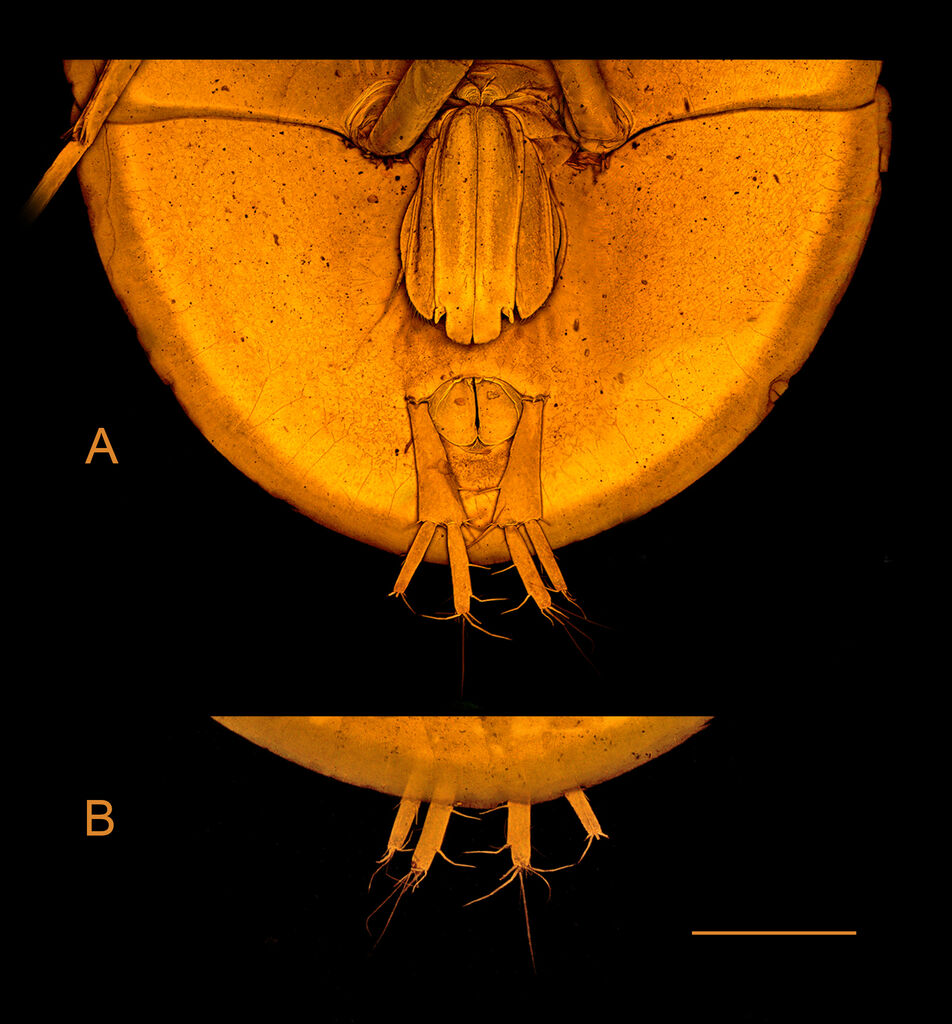
*Austroniscusindobathyasellus* Kaiser, Kniesz & Kihara, **sp. nov.**, holotype male (SMF 61327), confocal laser scanning microscopy images. **A.** Pleotelson, ventral view; **B.** Pleotelson posterior margin and uropods, dorsal view. Scale bar: 400 µm.

**Habitus** (Fig. 40A, E and Figs 41, 42, 43) body length 4.0 mm; body dorsoventrally flattened and broadened, body length 2.3 Prn2 width and 1.9 maximum body width (pereonite 5); Prn4–7 and Plt of similar width; Prn1–3 with strongly frontally directed lateral margins, tipped with a small spine-like seta apically. Prn1 narrowest, length 0.1 width; Prn2 width 1.4 Prn1 width, length 1.8 Prn1 length; Prn2 and 3 of similar length. Prn4–6 similar length at mid-line, 2.5 Prn1 length; Prn7 longest, length 3.2 Prn1 length; Prn5 anterior margin straight; Prn6–7 anterior margins strongly convex. **Prn2–7** coxa inserting ventrally and, hence, not visible in dorsal view; Prn1 coxa inserting anteriorly, each with a spine-like frontally directed coxal extension, tipped with a small spine-like seta and clearly visible in dorsal view, almost reaching second art of A1; length coxal extension 2.5 width, with width broadest in the middle and narrowing both proximally and distally. **Plt** length 0.3 body length, length 0.5 width; width 1.7 Prn1 width, posterior margin semicircular and rounded, anterior margin slightly concave. **Urp** length 0.5 Plt length, slightly projecting beyond posterior margin.

**Ceph** (Figs 40, 41, 42A) free, length 0.7 width. Rostrum well developed. Ceph anterior margin straight, posterior margin slightly rounded, A1 and A2 inserting anterolaterally in a deep fold between rostral crest and anterolateral, triangular projections; each fold with a single robust seta medially.

**A1** (Fig. 40A–C and Fig. 42A), broken off from third art onwards; first art ovoid and broad, length 1.2 width, distally with 1 long broom seta and 1 slender simple seta; second art length 1.3 art1 length, 5.0 width, distally with 2 long broom setae and 2 simple setae.

**A2** (Fig. 40C–D and Fig. 42A); broken off, only peduncular art1–art4 present; art1–art4 short; art2 with a small robust seta tipped with a small setula distally; art3 with 1 large robust scale, tipped with a small seta distally.

**P5** (Fig. 40H) basis length 3.9 width, with 2 long broom setae and 1 simple seta dorsally, with 3 simple setae ventrally and 1 somewhat longer simple seta distoventrally; ischium length 0.7 basis length, length 2.9 width, with 3 simple setae (2 small, 1 somewhat more robust) dorsally, with 2 simple setae ventrally; merus length 0.4 ischium length, length 1.2 width, with 2 robust simple setae distodorsally, with 1 slender simple seta distoventrally; carpus length 3.8 merus length, length 4.1 width, with a row of 17 long natatory setae each bearing 1 row of setulae and 3 robust unequally bifid setae dorsally, with 1 long broom seta and 1 small simple seta distodorsally, with 8 setae (4 simple and 4 robust unequally bifid) ventrally; propodus as long as carpus, length 6.6 width, with a row of 17 long natatory setae each bearing 1 row of setulae and 1 unequally bifid seta dorsally, with 8 unequally bifid setae ventrally; dactylus length 0.3 propodus length, length 5.0 width, with 3 simple setae medially; unguis length 0.3 dactylus length, with 2 slender setae between unguis and ventral claw.

**Plp1** (Fig. 40F and Fig. 43A); length 3.0 proximal width; distal projection width 0.7 proximal width, lateral margins straight; lateral lobes tapering sharply; distal margins almost straight, with 10–11 simple setae of varying length each.

**Urp** (Fig. 40E, G and Fig. 43A); biramous; sympod triangular, length 2.9 distal width, with 2 simple setae of varying size laterally, with 3 setae distally (2 long simple, 1 broken off); exopod length 0.6 sympod length, length 5.1 width, with 2 setae laterally (1 simple, 1 broken off), with 7 setae terminally (4 broken off, 3 long slender); endopod length 1.2 exopod length, length 5.4 width, with 4 simple setae of varying size laterally, with 9 (3 broom, 6 broken off) setae terminally.

#### Diagnosis

Pereonite 1 lateral margins frontally directed; coxae of pereonite 1 inserting anteriorly, each with a spine-like frontally directed appendix, tipped with a small spine-like seta and clearly visible in dorsal view, almost reaching second article of the antenna I; length-width ratio of the coxal extension < 3; pereonites 1–3 anterolateral margins each with a spine-like seta. Pleopod 1 distal margins almost straight, lateral lobes tapering sharply.

Molecular diagnosis: The new species is differing in the COI-gene from other species within *Austroniscus* in the nucleotides G (position 47 of the alignment), T (48), G (62), C (69), C (80), C (95), C (111), C (145), T (173), T (175), C (206), T (208), A (232), A (278), G (298), A (391), G (430), C (438), T (449), A (514), C (518), T (520), C (595) and C (601).

#### Etymology

The specific epithet *indobathyasellus* is a compound noun in apposition, crafted from Greek and Latin elements representing origin, habitat and morphological resemblance of the new species. It is to be treated as a Latin noun in nominative singular. The element *indo* refers to the ocean where the species dwells, honouring the first report of the genus and family in this region. The element *bathy* refers to the bathyal hydrothermal vents near which the holotype was found, implying potential adaptations to this habitat. The element *asellus*, along with the element *oniscus* in *Austroniscus*, refers to the terrestrial isopod *Oniscusasellus* Linnaeus, 1758, highlighting the new species’ form similarity with its terrestrial counterpart.

#### Taxon discussion

Creating a taxonomic description based on a single individual, especially when lacking many appendages, is not considered optimal. However, the new species has distinctive features, notably the anteriorly inserting coxae of pereonite 1, that help distinguish it from all other known species of the genus. Furthermore, since the specimen is a male, there may be gender-dependent differences that are not accounted for. Nonetheless, species in the genus typically display minimal or only slightly expressed sexual dimorphism (e.g., Kaiser et al. 2023), allowing us to make reasonable inferences about the female characteristics.

This new species marks the first formal description of a species within the genus *Austroniscus* from the Indian Ocean. Even more remarkably, this constitutes also the first described species within the family Nannoniscidae originating from this vast geographical region. This highlights how little is known about the deep-sea areas of this region. Moreover, it stresses the potential of new species discoveries through enhanced exploration and taxonomic analysis to bridge existing biogeographic gaps.

The new species exhibits similarities with a cluster of species distinguished by a wide body width (where body length is less than 2.1 times the maximum body width) from all other species in the genus, specifically *A.chelus*, *A.brandtae*, *A.obscurus* and *A.ovalis*. Additionally, the new species displays a distinctive elongation of the coxae of pereopod 1, extending notably beyond the anterolateral margins of pereonite 1 (in dorsal view), a feature shared with *A.chelus*, *A.brandtae* and *A.obscurus*. The comparison of the new species is limited to this specific group.

No males have been described for *A.obscurus* and *A.chelus*. Therefore, comparisons with the new species are based on differing genders for these species. Only in the case of *A.brandtae*, the comparison with the new species is focused on male features. *A.indobathyasellus* sp. nov. differs from these species by the following characters: 1) coxa of pereonite 1 inserting anteriorly (vs. centrally in the other species); 2) length-width ratio of the coxal extension < 3 (vs. > 4); 3) width of the coxal extension broadest in the middle and narrowing both distally and proximally (vs. width progressively tapering towards the distal end); and 4) pereonite 1 width ≤ 0.7 pereonite 2 width (vs. pereonite 1 width > 0.8 pereonite 2 width). *A.indobathyasellus* sp. nov. can be further distinguished from *A.brandtae* as follows: Pleopod 1 distal margins almost straight, lateral lobes tapering sharply (vs. distal margins semicircular, lateral lobes rounded).

#### Notes

##### Methods

The holotype was collected from hard substrates with maximum distance of 500 m to hydrothermal vents, but at least 20 m apart from active venting of a newly-discovered hydrothermal vent field on the Central Indian Ridge. Samples were obtained during the INDEX2019 expedition onboard RV Sonne (SO271-1) (Station No. I19_036RO_BB_01; latitude -25.466675, longitude 69.916682, 2628 m depth) through rock picking using a remotely operated vehicle (ROV). Upon retrieval on deck, the rocks were washed over sieves and the samples promptly fixed in 96% ethanol. For DNA analyses of the mitochondrial cytochrome c oxidase subunit 1 (COI) gene, extraction, amplification and sequencing were carried out following protocols provided by Kniesz et al. (2022) and the resulting sequences are accessible via GenBank (accession no. OR825323).

Morphological examination was conducted using a Leica MZ 8 stereomicroscope, pencil drawings being created using a Leica DM750 microscope with a camera lucida and subsequently inked. Confocal laser scanning microscopy (CLSM) was performed following the methods described in Kaiser et al. (2023). Morphological terminology follows Hessler (1970) and Riehl and Brandt (2010), whilst morphological measurements follow methods proposed by Hessler (1970). In addition, body length to maximum body width was measured, where body length is defined as the mid-sagittal distance from the anterior edge of the cephalothorax to the posterior tip of the pleotelson and maximum width is determined at the specimen's broadest extent observed from the ventral view. Finally, the length of the coxal extension was measured ventrally from the distal end to the proximal mid-point where it meets the coxa and width determined at its broadest point from the ventral view. The specimen is stored in the Crustacean collection of the Senckenberg Research Institute and Natural History Museum in Frankfurt am Main (SMF) under catalogue number SMF 61327.

The species was compared with relevant primary literature to assess its similarities with other species in the genus (Kaiser and Brandt 2007; Kaiser et al. 2023), as well as with the type material of the following species: *Austroniscusbrandtae*, Senckenberg Research Institute and Natural History Museum, holotype male (SMF 57927) and paratype female (SMF 57930) and *Austroniscusrotundatus*, holotype male, Museum für Naturkunde Berlin (No. 17685).

For molecular diagnosis, we employed the open-access tool DeSignate (Hütter et al. 2020) to discern pairwise diagnostic disparities (i.e. nucleotide variations) amongst species using the COI marker. A reference alignment was generated using the MAFFT plugin with default settings in Geneious v.2023.2.1, encompassing the new species and four congenerics, where data were available in GenBank: Austroniscuscf.groenlandicus (GenBank accession No. MZ151074.1); *Austroniscus* sp. voucher D3D51 (MZ151108.1); *Austroniscus* sp. Voucher D3D30 (MZ151128.1); *Austroniscusbrandtae* (OM892250.1).

### Apseudopsis daria

Esquete & Tato sp. nov.

063E587F-95B1-5342-B5D6-5980F14ACD37

E0758169-F657-4930-BEAF-A99FDAB807C3

#### Materials

- **Type status:** Holotype. **Occurrence:** catalogNumber: MNCN20.04/20824; recordedBy: Ramiro Tato; individualCount: 1; sex: female; lifeStage: adult; preparations: whole animal (ETOH); occurrenceID: 970E2FE6-6D51-59C8-BA97-703D6AB80468; **Taxon:** scientificName: *Apseudopsisdaria* Esquete & Tato; kingdom: Animalia; phylum: Arthropoda; class: Malacostraca; order: Tanaidacea; family: Apseudidae; genus: Apseudopsis; specificEpithet: *daria*; scientificNameAuthorship: Esquete & Tato; nomenclaturalCode: ICZN; **Location:** continent: Europe; waterBody: Ría de Ferrol; country: Spain; stateProvince: A Coruña; locality: inlet inside the Ría of Ferrol; verbatimDepth: 10 m; locationRemarks: clayey mud; verbatimCoordinates: 43° 46.376' N 008° 24.085' W; **Event:** samplingProtocol: Van-Veen grab, sieved through 0.5 mm mesh; eventDate: 17/04/2012; fieldNumber: REG SEM EST 2 04/12; **Record Level:** institutionCode: MNCN; basisOfRecord: PreservedSpecimen
- **Type status:** Paratype. **Occurrence:** catalogNumber: MNCN20.04/20825; recordedBy: Ramiro Tato; individualCount: 1; sex: male; lifeStage: adult; preparations: whole animal (ETOH); occurrenceID: 6870CD58-BDC5-548C-9273-EDD96B097B6E; **Taxon:** scientificName: *Apseudopsisdaria* Esquete & Tato; kingdom: Animalia; phylum: Arthropoda; class: Malacostraca; order: Tanaidacea; family: Apseudidae; genus: Apseudopsis; specificEpithet: *daria*; scientificNameAuthorship: Esquete & Tato; nomenclaturalCode: ICZN; **Location:** continent: Europe; waterBody: Ría de Ferrol; country: Spain; stateProvince: A Coruña; locality: inlet inside the Ría of Ferrol; verbatimDepth: 10 m; locationRemarks: clayey mud; verbatimCoordinates: 43° 46.376' N 008° 24.085' W; **Event:** samplingProtocol: Van-Veen grab, sieved through 0.5 mm mesh; eventDate: 17/04/2012; fieldNumber: REG SEM EST 2 04/12; **Record Level:** institutionCode: MNCN; basisOfRecord: PreservedSpecimen
- **Type status:** Paratype. **Occurrence:** catalogNumber: DBUA0003243.04.a; recordedBy: Ramiro Tato; individualCount: 1; sex: female; lifeStage: female; preparations: whole animal (ETOH); occurrenceID: 1C4DB208-D4FB-5883-8F2A-69D3068667E1; **Taxon:** scientificName: *Apseudopsisdaria* Esquete & Tato; kingdom: Animalia; phylum: Arthropoda; class: Malacostraca; order: Tanaidacea; family: Apseudidae; genus: Apseudopsis; specificEpithet: *daria*; scientificNameAuthorship: Esquete & Tato; nomenclaturalCode: ICZN; **Location:** continent: Europe; waterBody: Ría de Ferrol; country: Spain; stateProvince: A Coruña; locality: inlet inside the Ría of Ferrol; verbatimDepth: 12.1 m; locationRemarks: clayey mud; verbatimCoordinates: 43° 46.376' N 008° 24.085' W; **Event:** samplingProtocol: Van-Veen grab, sieved through 0.5 mm mesh; eventDate: 06/07/2011; fieldNumber: REG TRIM EST 2 07/11; **Record Level:** institutionCode: DBUA; basisOfRecord: PreservedSpecimen
- **Type status:** Paratype. **Occurrence:** catalogNumber: DBUA0003243.04.b; recordedBy: Ramiro Tato; individualCount: 1; sex: female; lifeStage: female; preparations: whole animal (ETOH); occurrenceID: E39B8B9A-C19E-50A5-B722-898E11CA2FC1; **Taxon:** scientificName: *Apseudopsisdaria* Esquete & Tato; kingdom: Animalia; phylum: Arthropoda; class: Malacostraca; order: Tanaidacea; family: Apseudidae; genus: Apseudopsis; specificEpithet: *daria*; scientificNameAuthorship: Esquete & Tato; nomenclaturalCode: ICZN; **Location:** continent: Europe; waterBody: Ría de Ferrol; country: Spain; stateProvince: A Coruña; locality: inlet inside the Ría of Ferrol; verbatimDepth: 12.1 m; locationRemarks: clayey mud; verbatimCoordinates: 43° 46.376' N 008° 24.085' W; **Event:** samplingProtocol: Van-Veen grab, sieved through 0.5 mm mesh; eventDate: 06/07/2011; fieldNumber: REG TRIM EST 2 07/11; **Record Level:** institutionCode: DBUA; basisOfRecord: PreservedSpecimen
- **Type status:** Other material. **Occurrence:** catalogNumber: DBUA0003243.01; recordedBy: Ramiro Tato; individualCount: 1; sex: neuter; lifeStage: juvenile; preparations: whole animal (ETOH); occurrenceID: B207E795-EE72-537F-A5A1-E15899F22EC9; **Taxon:** scientificName: *Apseudopsisdaria* Esquete & Tato; kingdom: Animalia; phylum: Arthropoda; class: Malacostraca; order: Tanaidacea; family: Apseudidae; genus: Apseudopsis; specificEpithet: *daria*; scientificNameAuthorship: Esquete & Tato; nomenclaturalCode: ICZN; **Location:** continent: Europe; waterBody: Ría de Ferrol; country: Spain; stateProvince: A Coruña; locality: inlet inside the Ría of Ferrol; verbatimDepth: 12 m; locationRemarks: clayey mud; verbatimCoordinates: 43° 46.376' N 008° 24.085' W; **Event:** samplingProtocol: Van-Veen grab, sieved through 0.5 mm mesh; eventDate: 16/04/2009; fieldNumber: REG SEM EST 2 04/09; **Record Level:** institutionCode: DBUA; basisOfRecord: PreservedSpecimen
- **Type status:** Other material. **Occurrence:** catalogNumber: DBUA0003243.02; recordedBy: Ramiro Tato; individualCount: 2; sex: female, neuter; lifeStage: adult, juvenile; preparations: whole animal (ETOH); occurrenceID: 9C9F3CF2-D400-59C0-9F5B-F2A526D4ADEB; **Taxon:** scientificName: *Apseudopsisdaria* Esquete & Tato; kingdom: Animalia; phylum: Arthropoda; class: Malacostraca; order: Tanaidacea; family: Apseudidae; genus: Apseudopsis; specificEpithet: *daria*; scientificNameAuthorship: Esquete & Tato; nomenclaturalCode: ICZN; **Location:** continent: Europe; waterBody: Ría de Ferrol; country: Spain; stateProvince: A Coruña; locality: inlet inside the Ría of Ferrol; verbatimDepth: 6.7 m; locationRemarks: mud with shells; verbatimCoordinates: 43° 46.222' N 008° 26.798' W; **Event:** samplingProtocol: Van-Veen grab, sieved through 0.5 mm mesh; eventDate: 14/04/2010; fieldNumber: REG SEM EST 8 04/10; **Record Level:** institutionCode: DBUA; basisOfRecord: PreservedSpecimen
- **Type status:** Other material. **Occurrence:** catalogNumber: DBUA0003243.03; recordedBy: Ramiro Tato; individualCount: 2; sex: neuter; lifeStage: juvenile; preparations: whole animal (ETOH); occurrenceID: CDAAC32E-C8CF-5C34-978E-7A40838F088E; **Taxon:** scientificName: *Apseudopsisdaria* Esquete & Tato; kingdom: Animalia; phylum: Arthropoda; class: Malacostraca; order: Tanaidacea; family: Apseudidae; genus: Apseudopsis; specificEpithet: *daria*; scientificNameAuthorship: Esquete & Tato; nomenclaturalCode: ICZN; **Location:** continent: Europe; waterBody: Ría de Ferrol; country: Spain; stateProvince: A Coruña; locality: inlet inside the Ría of Ferrol; verbatimDepth: 10.9 m; locationRemarks: clayey mud; verbatimCoordinates: 43° 46.376' N 008° 24.085' W; **Event:** samplingProtocol: Van-Veen grab, sieved through 0.5 mm mesh; eventDate: 19/04/2011; fieldNumber: REG SEM EST 2 04/11; **Record Level:** institutionCode: DBUA; basisOfRecord: PreservedSpecimen
- **Type status:** Other material. **Occurrence:** catalogNumber: DBUA0003243.05; recordedBy: Ramiro Tato; individualCount: 1; sex: neuter; lifeStage: juvenile; preparations: whole animal (ETOH); occurrenceID: 914A9566-DB40-53D4-9BFE-6F293EE99155; **Taxon:** scientificName: *Apseudopsisdaria* Esquete & Tato; kingdom: Animalia; phylum: Arthropoda; class: Malacostraca; order: Tanaidacea; family: Apseudidae; genus: Apseudopsis; specificEpithet: *daria*; scientificNameAuthorship: Esquete & Tato; nomenclaturalCode: ICZN; **Location:** continent: Europe; waterBody: Ría de Ferrol; country: Spain; stateProvince: A Coruña; locality: inlet inside the Ría of Ferrol; verbatimDepth: 5.2 m; locationRemarks: mud; verbatimCoordinates: 43° 27.7365' N, 008° 14.742' W; **Event:** samplingProtocol: Van-Veen grab, sieved through 0.5 mm mesh; eventDate: 15/09/2011; fieldNumber: REG BIM Z4 R2 09/11; **Record Level:** institutionCode: DBUA; basisOfRecord: PreservedSpecimen
- **Type status:** Other material. **Occurrence:** catalogNumber: DBUA0003243.06; recordedBy: Ramiro Tato; individualCount: 1; sex: neuter; lifeStage: juvenile; preparations: whole animal (ETOH); occurrenceID: 7857A4C4-EE4B-51A1-A145-98A5172E392C; **Taxon:** scientificName: *Apseudopsisdaria* Esquete & Tato; kingdom: Animalia; phylum: Arthropoda; class: Malacostraca; order: Tanaidacea; family: Apseudidae; genus: Apseudopsis; specificEpithet: *daria*; scientificNameAuthorship: Esquete & Tato; nomenclaturalCode: ICZN; **Location:** continent: Europe; waterBody: Ría de Ferrol; country: Spain; stateProvince: A Coruña; locality: inlet inside the Ría of Ferrol; verbatimDepth: 12.3 m; locationRemarks: clayey mud; verbatimCoordinates: 43° 46.376' N 008° 24.085' W; **Event:** samplingProtocol: Van-Veen grab, sieved through 0.5 mm mesh; eventDate: 09/11/2011; fieldNumber: REG SEM EST 2 11/11; **Record Level:** institutionCode: DBUA; basisOfRecord: PreservedSpecimen
- **Type status:** Other material. **Occurrence:** catalogNumber: DBUA0003243.06; recordedBy: Ramiro Tato; individualCount: 3; sex: female, neuter; lifeStage: adult, juveniles; preparations: whole animal (ETOH); occurrenceID: 2A180C3E-9208-5E76-A7B2-7AD8092F94C5; **Taxon:** scientificName: *Apseudopsisdaria* Esquete & Tato; kingdom: Animalia; phylum: Arthropoda; class: Malacostraca; order: Tanaidacea; family: Apseudidae; genus: Apseudopsis; specificEpithet: *daria*; scientificNameAuthorship: Esquete & Tato; nomenclaturalCode: ICZN; **Location:** continent: Europe; waterBody: Ría de Ferrol; country: Spain; stateProvince: A Coruña; locality: inlet inside the Ría of Ferrol; verbatimDepth: 12.3 m; locationRemarks: clayey mud; verbatimCoordinates: 43° 46.376' N 008° 24.085' W; **Event:** samplingProtocol: Van-Veen grab, sieved through 0.5 mm mesh; eventDate: 09/11/2011; fieldNumber: REG SEM EST 2 11/11; **Record Level:** institutionCode: DBUA; basisOfRecord: PreservedSpecimen
- **Type status:** Other material. **Occurrence:** catalogNumber: DBUA0003243.07; recordedBy: Ramiro Tato; individualCount: 1; sex: male; lifeStage: adult; preparations: whole animal (ETOH); occurrenceID: 73F1A50F-7647-514D-A281-D47A6BB6B6D9; **Taxon:** scientificName: *Apseudopsisdaria* Esquete & Tato; kingdom: Animalia; phylum: Arthropoda; class: Malacostraca; order: Tanaidacea; family: Apseudidae; genus: Apseudopsis; specificEpithet: *daria*; scientificNameAuthorship: Esquete & Tato; nomenclaturalCode: ICZN; **Location:** continent: Europe; waterBody: Ría de Ferrol; country: Spain; stateProvince: A Coruña; locality: inlet inside the Ría of Ferrol; verbatimDepth: 12.9 m; locationRemarks: clayey mud; verbatimCoordinates: 43° 46.376' N 008° 24.085' W; **Event:** samplingProtocol: Van-Veen grab, sieved through 0.5 mm mesh; eventDate: 21/02/2012; fieldNumber: REG TRIM EST 2 02/12; **Record Level:** institutionCode: DBUA; basisOfRecord: PreservedSpecimen
- **Type status:** Other material. **Occurrence:** catalogNumber: DBUA0003243.08; recordedBy: Ramiro Tato; individualCount: 1; sex: male; lifeStage: adult; preparations: whole animal (ETOH); occurrenceID: DEE6C31D-834E-5945-93A3-FA3CCD9A5072; **Taxon:** scientificName: *Apseudopsisdaria* Esquete & Tato; kingdom: Animalia; phylum: Arthropoda; class: Malacostraca; order: Tanaidacea; family: Apseudidae; genus: Apseudopsis; specificEpithet: *daria*; scientificNameAuthorship: Esquete & Tato; nomenclaturalCode: ICZN; **Location:** continent: Europe; waterBody: Ría de Ferrol; country: Spain; stateProvince: A Coruña; locality: inlet inside the Ría of Ferrol; verbatimDepth: 11.7 m; locationRemarks: clayey mud; verbatimCoordinates: 43° 46.376' N 008° 24.085' W; **Event:** samplingProtocol: Van-Veen grab, sieved through 0.5 mm mesh; eventDate: 13/06/2012; fieldNumber: REG TRIM EST 2 06/12; **Record Level:** institutionCode: DBUA; basisOfRecord: PreservedSpecimen
- **Type status:** Other material. **Occurrence:** catalogNumber: DBUA0003243.09; recordedBy: Ramiro Tato; individualCount: 1; sex: neuter; lifeStage: juvenile; preparations: whole animal (ETOH); occurrenceID: 3943D67F-B029-5BC0-9737-DF8E7DD7625E; **Taxon:** scientificName: *Apseudopsisdaria* Esquete & Tato; kingdom: Animalia; phylum: Arthropoda; class: Malacostraca; order: Tanaidacea; family: Apseudidae; genus: Apseudopsis; specificEpithet: *daria*; scientificNameAuthorship: Esquete & Tato; nomenclaturalCode: ICZN; **Location:** continent: Europe; waterBody: Ría de Ferrol; country: Spain; stateProvince: A Coruña; locality: inlet inside the Ría of Ferrol; verbatimDepth: 8.9 m; locationRemarks: mud; verbatimCoordinates: 43° 46.222' N 008° 26.798' W; **Event:** samplingProtocol: Van-Veen grab, sieved through 0.5 mm mesh; eventDate: 13/06/2012; fieldNumber: REG TRIM EST 8 06/12; **Record Level:** institutionCode: DBUA; basisOfRecord: PreservedSpecimen
- **Type status:** Other material. **Occurrence:** catalogNumber: DBUA0003243.10; recordedBy: Ramiro Tato; individualCount: 1; sex: neuter; lifeStage: juvenile; preparations: whole animal (ETOH); occurrenceID: D00DE0F3-C652-592E-9B7F-DBAE07D1160A; **Taxon:** scientificName: *Apseudopsisdaria* Esquete & Tato; kingdom: Animalia; phylum: Arthropoda; class: Malacostraca; order: Tanaidacea; family: Apseudidae; genus: Apseudopsis; specificEpithet: *daria*; scientificNameAuthorship: Esquete & Tato; nomenclaturalCode: ICZN; **Location:** continent: Europe; waterBody: Ría de Ferrol; country: Spain; stateProvince: A Coruña; locality: inlet inside the Ría of Ferrol; verbatimDepth: 10 m; locationRemarks: mud; verbatimCoordinates: 43° 27.6391' N, 008° 15.910' W; **Event:** samplingProtocol: Van-Veen grab, sieved through 0.5 mm mesh; eventDate: 18/04/2013; fieldNumber: REG ANU EST 4 04/13; **Record Level:** institutionCode: DBUA; basisOfRecord: PreservedSpecimen
- **Type status:** Other material. **Occurrence:** catalogNumber: DBUA0003243.11; recordedBy: Ramiro Tato; individualCount: 1; sex: male; lifeStage: adult; preparations: whole animal (ETOH); occurrenceID: 134DDC84-0E79-57DC-A975-BD4F8CF18F91; **Taxon:** scientificName: *Apseudopsisdaria* Esquete & Tato; kingdom: Animalia; phylum: Arthropoda; class: Malacostraca; order: Tanaidacea; family: Apseudidae; genus: Apseudopsis; specificEpithet: *daria*; scientificNameAuthorship: Esquete & Tato; nomenclaturalCode: ICZN; **Location:** continent: Europe; waterBody: Ría de Ferrol; country: Spain; stateProvince: A Coruña; locality: inlet inside the Ría of Ferrol; verbatimDepth: 12 m; locationRemarks: clayey mud; verbatimCoordinates: 43° 46.376' N 008° 24.085' W; **Event:** samplingProtocol: Van-Veen grab, sieved through 0.5 mm mesh; eventDate: 29/04/2014; fieldNumber: REG SEM EST 2 04/14; **Record Level:** institutionCode: DBUA; basisOfRecord: PreservedSpecimen
- **Type status:** Other material. **Occurrence:** catalogNumber: DBUA0003243.12; recordedBy: Ramiro Tato; individualCount: 1; sex: neuter; lifeStage: juvenile; preparations: whole animal (ETOH); occurrenceID: 8E26621D-4197-5BC3-818E-84C6C2019ED4; **Taxon:** scientificName: *Apseudopsisdaria* Esquete & Tato; kingdom: Animalia; phylum: Arthropoda; class: Malacostraca; order: Tanaidacea; family: Apseudidae; genus: Apseudopsis; specificEpithet: *daria*; scientificNameAuthorship: Esquete & Tato; nomenclaturalCode: ICZN; **Location:** continent: Europe; waterBody: Ría de Ferrol; country: Spain; stateProvince: A Coruña; locality: inlet inside the Ría of Ferrol; verbatimDepth: 12.4 m; locationRemarks: clayey mud; verbatimCoordinates: 43° 46.376' N 008° 24.085' W; **Event:** samplingProtocol: Van-Veen grab, sieved through 0.5 mm mesh; eventDate: 10/07/2014; fieldNumber: REG TRIM EST 2 07/14; **Record Level:** institutionCode: DBUA; basisOfRecord: PreservedSpecimen
- **Type status:** Other material. **Occurrence:** catalogNumber: DBUA0003243.13; recordedBy: Ramiro Tato; individualCount: 1; sex: female; lifeStage: adult; preparations: whole animal (ETOH); occurrenceID: 38D0D35B-C42F-5CFE-B826-1471493C8018; **Taxon:** scientificName: *Apseudopsisdaria* Esquete & Tato; kingdom: Animalia; phylum: Arthropoda; class: Malacostraca; order: Tanaidacea; family: Apseudidae; genus: Apseudopsis; specificEpithet: *daria*; scientificNameAuthorship: Esquete & Tato; nomenclaturalCode: ICZN; **Location:** continent: Europe; waterBody: Ría de Ferrol; country: Spain; stateProvince: A Coruña; locality: inlet inside the Ría of Ferrol; verbatimDepth: 12.1 m; locationRemarks: clayey mud; verbatimCoordinates: 43° 46.376' N 008° 24.085' W; **Event:** samplingProtocol: Van-Veen grab, sieved through 0.5 mm mesh; eventDate: 14/10/2014; fieldNumber: REG SEM EST 2 10/14; **Record Level:** institutionCode: DBUA; basisOfRecord: PreservedSpecimen
- **Type status:** Other material. **Occurrence:** catalogNumber: DBUA0003243.14; recordedBy: Ramiro Tato; individualCount: 1; sex: male; lifeStage: adult; preparations: whole animal (ETOH); occurrenceID: F0FE13D4-3AB1-5BF5-9D23-0CCF83244C3E; **Taxon:** scientificName: *Apseudopsisdaria* Esquete & Tato; kingdom: Animalia; phylum: Arthropoda; class: Malacostraca; order: Tanaidacea; family: Apseudidae; genus: Apseudopsis; specificEpithet: *daria*; scientificNameAuthorship: Esquete & Tato; nomenclaturalCode: ICZN; **Location:** continent: Europe; waterBody: Ría de Ferrol; country: Spain; stateProvince: A Coruña; locality: inlet inside the Ría of Ferrol; verbatimDepth: 6.3 m; locationRemarks: mud; verbatimCoordinates: 43° 27.7365' N, 008° 14.742' W; **Event:** samplingProtocol: Van-Veen grab, sieved through 0.5 mm mesh; eventDate: 26/06/2015; fieldNumber: REG BIM Z4 R1 06/15; **Record Level:** institutionCode: DBUA; basisOfRecord: PreservedSpecimen
- **Type status:** Other material. **Occurrence:** catalogNumber: DBUA0003243.15; recordedBy: Ramiro Tato; individualCount: 1; sex: neuter; lifeStage: juvenile; preparations: whole animal (ETOH); occurrenceID: 09982A20-526F-5928-B1FD-B0CCD3D37C4F; **Taxon:** scientificName: *Apseudopsisdaria* Esquete & Tato; kingdom: Animalia; phylum: Arthropoda; class: Malacostraca; order: Tanaidacea; family: Apseudidae; genus: Apseudopsis; specificEpithet: *daria*; scientificNameAuthorship: Esquete & Tato; nomenclaturalCode: ICZN; **Location:** continent: Europe; waterBody: Ría de Ferrol; country: Spain; stateProvince: A Coruña; locality: inlet inside the Ría of Ferrol; verbatimDepth: 12.3 m; locationRemarks: clayey mud; verbatimCoordinates: 43° 46.376' N 008° 24.085' W; **Event:** samplingProtocol: Van-Veen grab, sieved through 0.5 mm mesh; eventDate: 21/10/2016; fieldNumber: REG SEM EST 2 10/16; **Record Level:** institutionCode: DBUA; basisOfRecord: PreservedSpecimen

#### Description

**Figure 44.**
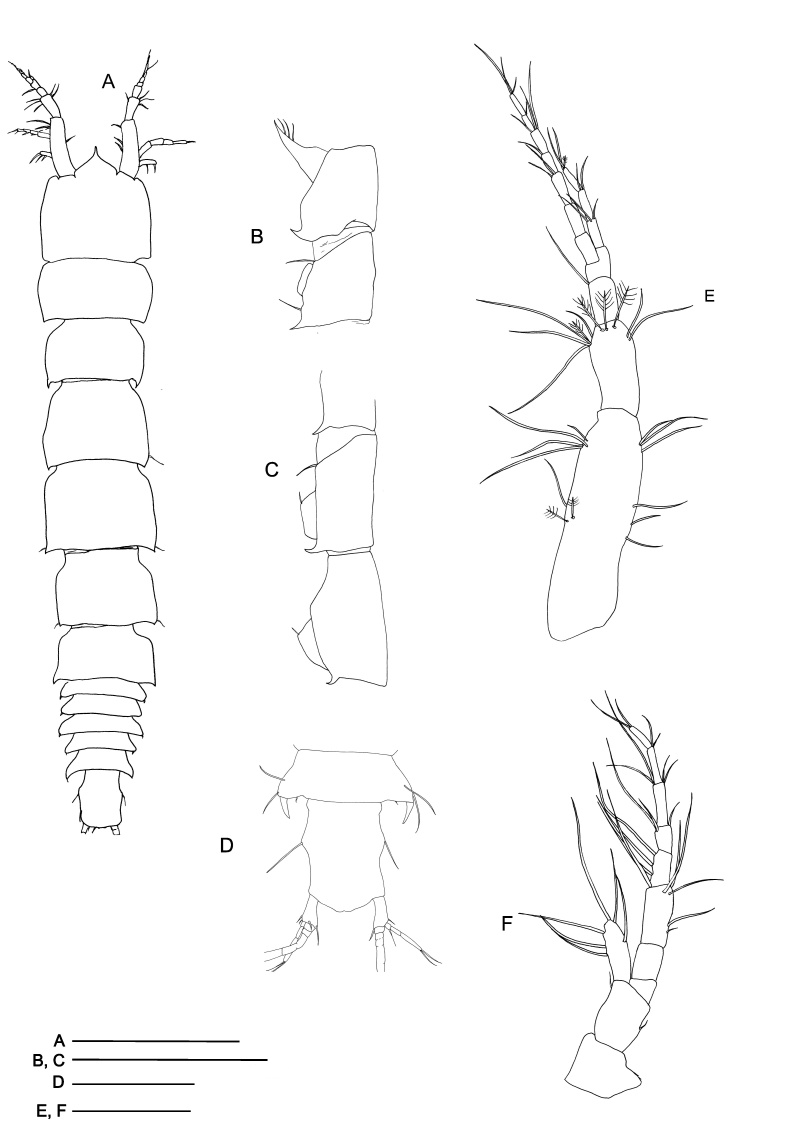
*Apseudopsisdaria* Esquete & Tato, **sp. nov.** Female paratype DBUA0003243.13. **A.** Habitus, dorsal view; **B.** Pereonites 1 and 2, lateral view; **C.** Pereonites 4 and 5, lateral view; **D.** Pleonite 5, pleotelson and uropods; **E.** Antennula; **F.** Antenna. Scale bars: 1 mm (A–C), 0.5 mm (D), 0.2 mm (E, F).

**Figure 45.**
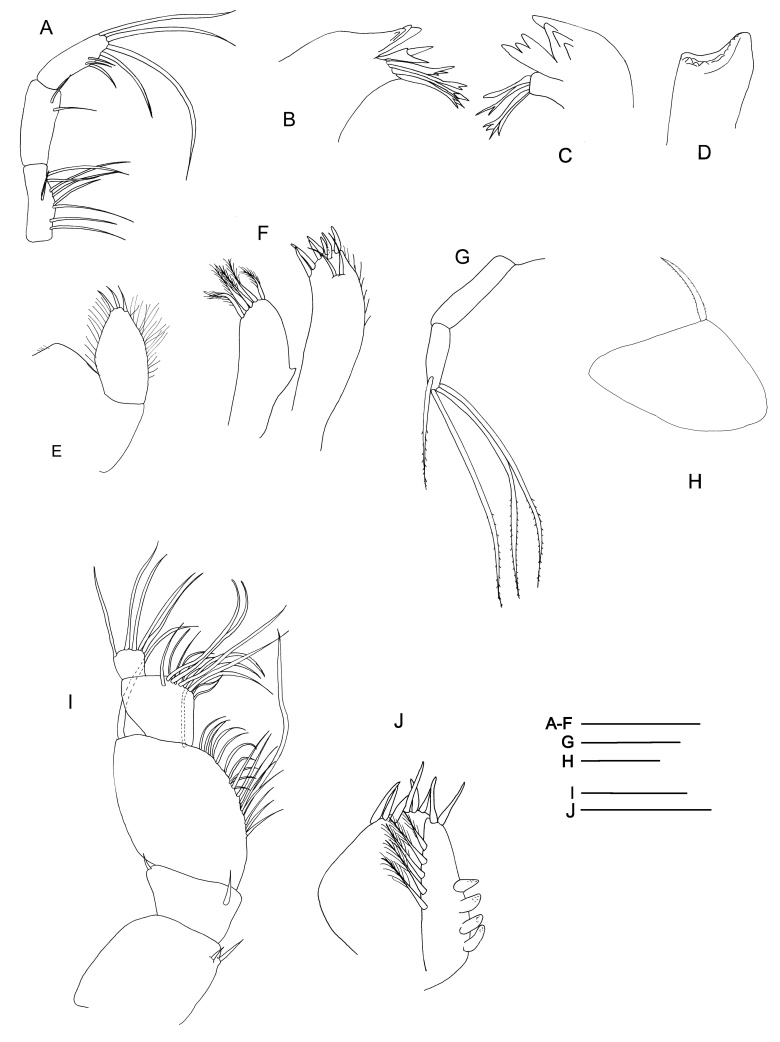
*Apseudopsisdaria* Esquete & Tato, **sp. nov.** Female paratype DBUA0003243.04.a. **A.** Mandible palp; **B.** Left mandible *pars incisiva*; **C.** Right mandible *pars incisiva*; **D.** *Pars molaris*; **E.** Labium palp; **F.** Maxillula endites; **G.** Maxillula palp; **H.** Epignath; **I.** Maxilliped palp; **J.** Maxilliped endite. Scale bars: 0.1 mm (A–H), 0.2 mm (I, J).

**Figure 46.**
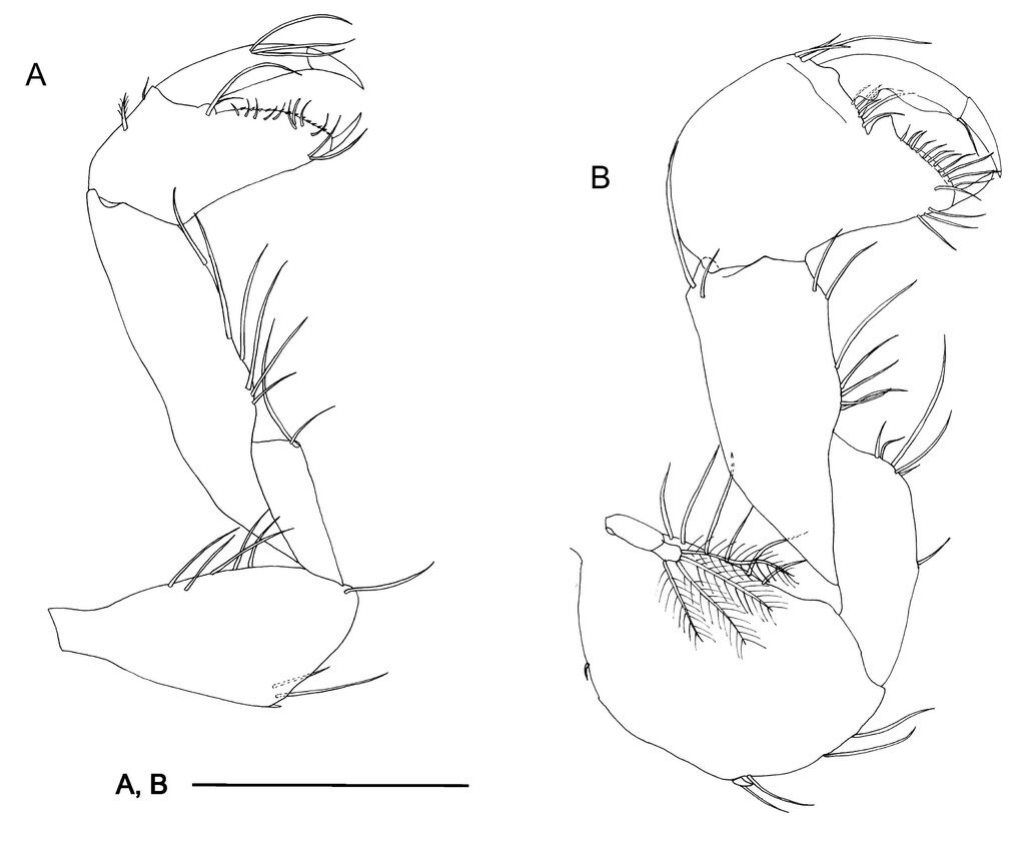
*Apseudopsisdaria* Esquete & Tato, **sp. nov. A.** Female paratype DBUA0003243.04.b, cheliped; **B.** Male DBUA0003243.14, cheliped. Scale bar: 0.5 mm.

**Figure 47.**
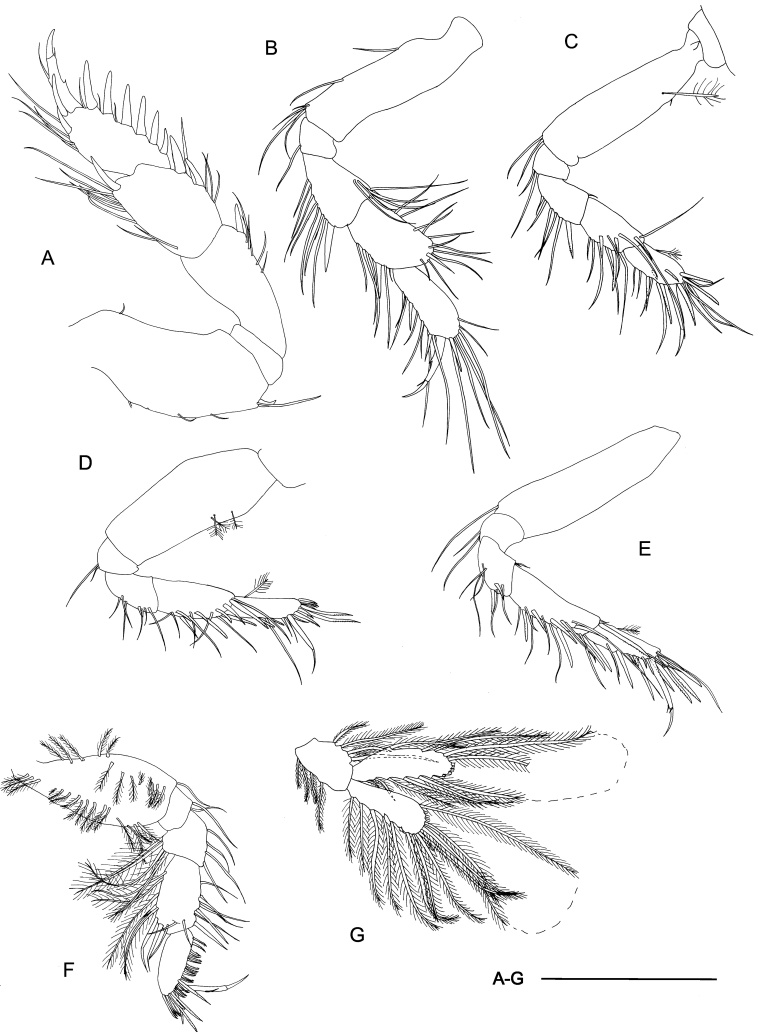
*Apseudopsisdaria* Esquete & Tato, **sp. nov.** Female paratype DBUA0003243.04.b. **A**–**F.** Pereopods 1 to 6, respectively (exopodites of cheliped and pereopod 1 not illustrated); **G.** Pleopod 1. Scale bar: 0.5 mm.

**Non-ovigerous female** (Figs 44, 45, Fig. 46A and Fig. 47). With general characters of *Apseudopsis* (Guţu 2006, Esquete et al. 2012a, Esquete et al. 2012b, Esquete et al. 2019, Carvalho et al. 2019). **Rostrum** (Fig. 44A) short, pointed, with rounded shoulders. **Prn1–6** (Fig. 44A–C) with posterolateral spiniform apophyses, hyposphenia only present on Prn6. **A1** (Fig. 44E) inner flagellum with three art, outer flagellum with seven art. **Mouthparts** (Fig. 45) as in other *Apseudopsis*; **Md** palp (Fig. 45A) art2 with two simple setae; **Mxp** palp (Fig. 45I) art1 with two inner distal short spines, endite (Fig. 45J) with four coupling hooks. **P1** (Fig. 47A) coxa (Fig. 44B) projected anteriorly; merus without dorsodistal spines; propodus with six ventral spines. **P3** basis with dorsal proximal spiniform apophysis (Fig. 47C). **Plp1** (Fig. 47G) basis with four inner and four outer plumose setae; **Plp2, 3 and 4** with four inner and three outer plumose setae; **Plp5** with three inner and two outer plumose setae.

**Male.** With general characters for *Apseudopsis* and same diagnostic characters as female holotype. **Ch** (Fig. 46B) carpus without apophyses, propodus without ventral projection, cutting edge with proximal semicircular apophysis, dactylus with invagination. Difference with female: Ch more robust (Fig. 46B).

#### Diagnosis

*Apseudopsis* Norman, 1899 with short, pointed rostrum, six ventral spines on pereopod 1 propodus and all pereonites with posterolateral projections. The only species with a posterior projection proximally on the basis of pereopod 3 in the adults.

#### Etymology

This species of *Apseudopsis* was first found in Ria de Ferrol, in Galicia (NW Spain). In Galician, “da ría” means “from the ria”. Noun in apposition.

#### Taxon discussion

Previous taxonomic studies revealed that spination and setation of most of the appendages vary intraspecifically according to the size and life stage of the individuals (Esquete et al. 2012a, Esquete et al. 2012b) and, hence, are not reliable for identification; therefore, they have been excluded from the description, although they can be consulted in the figures. The characters that allow distinction between species of *Apseudopsis*, regardless of the life stage of the individuals, are: 1) the shape of the rostrum; 2) the presence and/or position of apophyses on pereonites; 3) the number of hyposphenia on adult females without marsupium; 4) the number of articles on the flagella of the antennula and 5) the number of spines on pereopod 1 propodus (Esquete et al. 2012b). Additional characters are unique for certain species and typically involve ornamentation on the male cheliped or cephalothorax (Esquete et al. 2012a, Esquete et al. 2016, Esquete et al. 2019, Carvalho et al. 2019).

*Apseudopsisdaria* **sp. nov.** has a dorsal spiniform apophysis proximally on pereopod 3 (Fig. 47C), which is a unique character in the genus. Only three other species of *Apseudopsis* have six spines on pereopod 1 propodus: *A.arguinensis* (Guţu, 2002), *A.hastifrons* (Norman & Stebbing, 1886) and *A.isochelatus* Guţu, 2006. *Apseudopsisarguinensis* differs in lacking the posterolateral apophysis on pereonite 1, having short setae on anterolateral corners of pereonites and a hyposphenium on pereonite 2; *A.hastifrons* has anterolateral apophyses on pereonites 3 to 6, hyposphenia on pereonites 2, 3, 5 and 6 and a proximal row of spinules on the basis of pereopod 5; and *A.isochelatus* has a long rostrum, no apophyses on pereonite 1, hyposphenia on pereonites 2 to 6, antennule outer flagellum with up to 9 articles and the cheliped in males is as slender as in females. This is the only species of the genus with a proximal spiniform apophysis on pereopod 3; however, this character is very subtle and might have been overlooked in the descriptions of its congeners.

#### Notes

##### Methods

Twenty-three specimens were retrieved from two inlets inside the Ría of Ferrol (NW Iberian Peninsula). The bottoms of the ría are mostly sedimentary, composed of mud in the sheltered areas with different proportions of sand. The study sites range from 2 to 13 m in depth and experience an average value of temperature and salinity of 15°C and 35‰ throughout the year. The salinity values range between 33 and 36‰. The inlets are in the middle part of the ría with a small stream mouth in the O Baño inlet. The coordinates delimitating them are the following: Santa Lucía (8° 14.4570' W, 43° 27.8103' N; 8° 15.0513' W, 43° 27.8564' N; 8° 14.9853' W, 43° 27.6813' N; 8° 14.5612' W, 43° 27.6316' N) and O Baño (8° 15.7487' W, 43° 27.7495' N; 8° 16.1401' W, 43° 27.7661' N; 8° 16.2372' W, 43° 27.4804' N; 8° 16.0011' W, 43° 27.2739' N). Samples were obtained using a Van-Veen grab and sieved through 0.5 mm mesh. The faunal specimens were sorted and fixed in 5% formaldehyde, rinsed with freshwater and then preserved in 70% ethanol. Taxonomic treatment and terminology followed Błażewicz-Paszkowycz and Bamber (2007) and Esquete and Cunha (2017). The type material is deposited at the Museo Nacional de Ciencias Naturales, Madrid (MNCN), while other material at the Biological Research Collection of the Biology Department of the Universidade de Aveiro (DBUA).

### Psychropotes buglossa

E. Perrier 1886

BEC74BAC-B1AE-5D03-B97B-41A8007873DE

https://www.marinespecies.org/aphia.php?p=taxdetails&id=124773

*Psychropotesbuglossa* E. Perrier 1886: 283, fig. 200; R. Perrier 1896: 902; R. Perrier 1900: 119; R. Perrier 1902: 445, fig. 7, pl. XX figs. 16–28; Hérouard 1923: 105, Plate I, fig. 32, Plate VI, fig. 2.
*Psychropotesgrimaldii* Hérouard 1896: 167, figs. 2 a–c; 1902: 25, plate III figs. 1, 2; plate VIII figs. 10–15.
**Type material**: Nineteen syntypes (R. Perrier 1902); 13 individuals were collected in a single dredge. Specimens from this single dredge from between the Azores and France, *Talisman* Station 135, 43°15' N, 21°40' W, depth 4165 m, should be used to select a lectotype if this is considered necessary in the future. Fourteen syntypes are known to be in the collections of the Muséum national d'Histoire naturelle, Paris: 2013-17710, 2013-17712, 2013-13194, 2013-13196, 2013-13197, 2013-13198, 2013-13199, 2013-13283, 2013-13299, 2013-13316, 2013-13317, 2013-13318, 2013-13327 and 2013-13338. Examined from photographs.
**Type locality**: NE Atlantic: Between the Azores and France, *Talisman* Station 135, 43°15' N, 21°40' W, depth 4165 m; *Talisman* Station 133, 42°15' N, 23°37' W, depth 3975 m; *Talisman* Station 134, 42°19' N, 23°36' W, depth 4060 m; *Talisman* Station 136, 44°20' N, 19°31' W, depth 4255 m; *Talisman* Station 137, 44°29'-44°21' N, 15°52'-15°53'W, depth 4975-5005 m; Coast of Morocco, *Talisman* Station 38, 30°09' N, 14°01' W, depth 2210m.
**Remarks**: Edmond Perrier named this species in a popular book with a clear illustration of the whole animal in life position (E. Perrier (1886): 283, fig. 200). Rémy Perrier later described it in full, including E. Perrier’s previously-published illustration drawn from the largest of the syntype specimens (R. Perrier 1896, R. Perrier 1902). The publication with the earliest use of the name with a clearly identifiable illustration is considered the taxonomic authority and, therefore, the early problems with synonymy of *P.buglossa* with the species *P.grimaldii* Herouard, 1896 are irrelevant as the name was actually validly published a decade earlier (Hérouard 1896, R. Perrier 1896, R. Perrier 1900, Hérouard 1902, R. Perrier 1902, Hérouard 1923).
*Material examined*. Thirteen specimens collected by the RRS *James Cook* from the Porcupine Abyssal Plain Sustained Observatory in the NE Atlantic at 4840–4629 m.

#### Materials

- **Type status:** Other material. **Occurrence:** catalogNumber: JC231-082-012; recordedBy: Amanda Serpell-Stevens | Tammy Horton; individualCount: 1; lifeStage: adult; occurrenceStatus: present; preparations: ethanol; occurrenceID: 0CA1B0E7-3112-56DB-ACA2-B3421E717D69; **Taxon:** scientificNameID: urn:lsid:marinespecies.org:taxname:124773; scientificName: *Psychropotesbuglossa* E. Perrier, 1886; kingdom: Animalia; phylum: Echinodermata; class: Holothuroidea; order: Elasipodida; family: Psychropotidae; genus: Psychropotes; specificEpithet: *buglossa*; taxonRank: species; scientificNameAuthorship: E. Perrier, 1886; nomenclaturalCode: ICZN; **Location:** locationID: http://marineregions.org/mrgid/63025; higherGeographyID: http://vocab.nerc.ac.uk/collection/C19/current/; higherGeography: ATLANTIC OCEAN | NORTH ATLANTIC OCEAN | NORTHEAST ATLANTIC OCEAN (40W) | Porcupine Abyssal Plain; waterBody: NORTHEAST ATLANTIC OCEAN (40W); locality: Porcupine Abyssal Plain - Sustained Observatory; verbatimDepth: 4840 - 4844; minimumDepthInMeters: 4840; maximumDepthInMeters: 4844; maximumDistanceAboveSurfaceInMeters: 1.5; verbatimCoordinates: 48° 53.176' N, 016° 27.503' W to 48° 53.151' N, 016° 36.704' W; verbatimCoordinateSystem: degrees decimal minutes; verbatimSRS: EPSG:4326; decimalLatitude: 48.8860752112; decimalLongitude: -16.5350583759; geodeticDatum: EPSG:4326; coordinateUncertaintyInMeters: 5603; coordinatePrecision: 0.00016666666666667; footprintWKT: LINESTRING(-16.458 48.886, -16.612 48.886); footprintSRS: EPSG:4326; **Identification:** identifiedBy: Amanda Serpell-Stevens; dateIdentified: 2023; **Event:** eventID: JC231-082; samplingProtocol: OTSB14; eventDate: 13/05/2022T00:01:00Z/13/05/2022T02:22:00Z; verbatimEventDate: 13/05/2022 00:01 UTC - 13/05/2022 02:22 UTC; fieldNumber: JC231-082; fieldNotes: Hartman, S.E., 2022. RRS James Cook Cruise 231, 01 May - 19 May 2022. Time-series studies at the Porcupine Abyssal Plain Sustained Observatory. Southampton, National Oceanography Centre, 201pp. (National Oceanography Centre Cruise Report, 77). | https://nora.nerc.ac.uk/id/eprint/533356/1/Report%2077_JC231_Hartman.pdf; eventRemarks: Good catch. Dist run = 11.205 km (6.05 nm).; **Record Level:** language: en; institutionID: https://www.gbif.org/grscicoll/institution/74ae2bc3-e5a8-443f-bc8b-89cc223500d1; institutionCode: NOC; collectionCode: DISCOLL; basisOfRecord: PreservedSpecimen
- **Type status:** Other material. **Occurrence:** catalogNumber: JC231-082-EJC05; recordedBy: Amanda Serpell-Stevens | Tammy Horton; individualCount: 1; lifeStage: adult; occurrenceStatus: present; preparations: ethanol; associatedSequences: PP079631; occurrenceID: F0CB18BE-2B7E-5C60-8C89-CD6169E1764B; **Taxon:** scientificNameID: urn:lsid:marinespecies.org:taxname:124773; scientificName: *Psychropotesbuglossa* E. Perrier, 1886; kingdom: Animalia; phylum: Echinodermata; class: Holothuroidea; order: Elasipodida; family: Psychropotidae; genus: Psychropotes; specificEpithet: *buglossa*; taxonRank: species; scientificNameAuthorship: E. Perrier, 1886; nomenclaturalCode: ICZN; **Location:** locationID: http://marineregions.org/mrgid/63025; higherGeographyID: http://vocab.nerc.ac.uk/collection/C19/current/; higherGeography: ATLANTIC OCEAN | NORTH ATLANTIC OCEAN | NORTHEAST ATLANTIC OCEAN (40W) | Porcupine Abyssal Plain; waterBody: NORTHEAST ATLANTIC OCEAN (40W); locality: Porcupine Abyssal Plain - Sustained Observatory; verbatimDepth: 4840 - 4844; minimumDepthInMeters: 4840; maximumDepthInMeters: 4844; maximumDistanceAboveSurfaceInMeters: 1.5; verbatimCoordinates: 48° 53.176' N, 016° 27.503' W to 48° 53.151' N, 016° 36.704' W; verbatimCoordinateSystem: degrees decimal minutes; verbatimSRS: EPSG:4326; decimalLatitude: 48.8860752112; decimalLongitude: -16.5350583759; geodeticDatum: EPSG:4326; coordinateUncertaintyInMeters: 5603; coordinatePrecision: 0.00016666666666667; footprintWKT: LINESTRING(-16.458 48.886, -16.612 48.886); footprintSRS: EPSG:4326; **Identification:** identifiedBy: Amanda Serpell-Stevens; dateIdentified: 2023; **Event:** eventID: JC231-082; samplingProtocol: OTSB14; eventDate: 13/05/2022T00:01:00Z/13/05/2022T02:22:00Z; verbatimEventDate: 13/05/2022 00:01 UTC - 13/05/2022 02:22 UTC; fieldNumber: JC231-082; fieldNotes: Hartman, S.E., 2022. RRS James Cook Cruise 231, 01 May - 19 May 2022. Time-series studies at the Porcupine Abyssal Plain Sustained Observatory. Southampton, National Oceanography Centre, 201pp. (National Oceanography Centre Cruise Report, 77). | https://nora.nerc.ac.uk/id/eprint/533356/1/Report%2077_JC231_Hartman.pdf; eventRemarks: Good catch. Dist run = 11.205 km (6.05 nm).; **Record Level:** language: en; institutionID: https://www.gbif.org/grscicoll/institution/74ae2bc3-e5a8-443f-bc8b-89cc223500d1; institutionCode: NOC; collectionCode: DISCOLL; basisOfRecord: PreservedSpecimen
- **Type status:** Other material. **Occurrence:** catalogNumber: JC231-086-027; recordedBy: Amanda Serpell-Stevens | Tammy Horton; individualCount: 1; lifeStage: adult; occurrenceStatus: present; preparations: ethanol; occurrenceID: FB805F75-1EF7-56B6-9752-DE14BBC99ADF; **Taxon:** scientificNameID: urn:lsid:marinespecies.org:taxname:124773; scientificName: *Psychropotesbuglossa* E. Perrier, 1886; kingdom: Animalia; phylum: Echinodermata; class: Holothuroidea; order: Elasipodida; family: Psychropotidae; genus: Psychropotes; specificEpithet: *buglossa*; taxonRank: species; scientificNameAuthorship: E. Perrier, 1886; nomenclaturalCode: ICZN; **Location:** locationID: http://marineregions.org/mrgid/63025; higherGeographyID: http://vocab.nerc.ac.uk/collection/C19/current/; higherGeography: ATLANTIC OCEAN | NORTH ATLANTIC OCEAN | NORTHEAST ATLANTIC OCEAN (40W) | Porcupine Abyssal Plain; waterBody: NORTHEAST ATLANTIC OCEAN (40W); locality: Porcupine Abyssal Plain - Sustained Observatory; verbatimDepth: 4838 - 4841; minimumDepthInMeters: 4838; maximumDepthInMeters: 4841; maximumDistanceAboveSurfaceInMeters: 1.5; verbatimCoordinates: 48° 54.974' N, 016° 43.110' W to 48° 51.613' N, 016° 48.225' W; verbatimCoordinateSystem: degrees decimal minutes; verbatimSRS: EPSG:4326; decimalLatitude: 48.888235848; decimalLongitude: -16.7611321281; geodeticDatum: EPSG:4326; coordinateUncertaintyInMeters: 4408; coordinatePrecision: 0.00016666666666667; footprintWKT: LINESTRING(-16.718 48.916, -16.804 48.860); footprintSRS: EPSG:4326; **Identification:** identifiedBy: Amanda Serpell-Stevens; dateIdentified: 2023; **Event:** eventID: JC231-086; samplingProtocol: OTSB14; eventDate: 13/05/2022T23:34:00Z/14/05/2022T01:18:00Z; verbatimEventDate: 13/05/2022 23:34 UTC - 14/05/2022 01:18 UTC; fieldNumber: JC231-086; fieldNotes: Hartman, S.E., 2022. RRS James Cook Cruise 231, 01 May - 19 May 2022. Time-series studies at the Porcupine Abyssal Plain Sustained Observatory. Southampton, National Oceanography Centre, 201pp. (National Oceanography Centre Cruise Report, 77). | https://nora.nerc.ac.uk/id/eprint/533356/1/Report%2077_JC231_Hartman.pdf; eventRemarks: Good catch. Dist run = 8.816 km (6.05 nm).; **Record Level:** language: en; institutionID: https://www.gbif.org/grscicoll/institution/74ae2bc3-e5a8-443f-bc8b-89cc223500d1; institutionCode: NOC; collectionCode: DISCOLL; basisOfRecord: PreservedSpecimen
- **Type status:** Other material. **Occurrence:** catalogNumber: JC237-055-10; recordedBy: Amanda Serpell-Stevens | Tammy Horton; individualCount: 1; lifeStage: adult; occurrenceStatus: present; preparations: ethanol; occurrenceID: 1964A273-0293-5055-BEEA-D481AAFBE665; **Taxon:** scientificNameID: urn:lsid:marinespecies.org:taxname:124773; scientificName: *Psychropotesbuglossa* E. Perrier, 1886; kingdom: Animalia; phylum: Echinodermata; class: Holothuroidea; order: Elasipodida; family: Psychropotidae; genus: Psychropotes; specificEpithet: *buglossa*; taxonRank: species; scientificNameAuthorship: E. Perrier, 1886; nomenclaturalCode: ICZN; **Location:** locationID: http://marineregions.org/mrgid/63025; higherGeographyID: http://vocab.nerc.ac.uk/collection/C19/current/; higherGeography: ATLANTIC OCEAN | NORTH ATLANTIC OCEAN | NORTHEAST ATLANTIC OCEAN (40W) | Porcupine Abyssal Plain; waterBody: NORTHEAST ATLANTIC OCEAN (40W); locality: Porcupine Abyssal Plain - Sustained Observatory; verbatimDepth: 4629; minimumDepthInMeters: 4629; maximumDepthInMeters: 4629; maximumDistanceAboveSurfaceInMeters: 1.5; verbatimCoordinates: 48° 57.3600' N, 016° 32.6885' W; verbatimCoordinateSystem: degrees decimal minutes; verbatimSRS: EPSG:4326; decimalLatitude: 48.956; decimalLongitude: -16.544808333333; geodeticDatum: EPSG:4326; **Identification:** identifiedBy: Amanda Serpell-Stevens; dateIdentified: 2023; **Event:** eventID: JC237-055-10; samplingProtocol: OTSB14; eventDate: 28/08/2022T09:35:00Z; verbatimEventDate: 28/08/2022 09:35:00 UTC; fieldNumber: JC237-055-10; **Record Level:** language: en; institutionID: https://www.gbif.org/grscicoll/institution/74ae2bc3-e5a8-443f-bc8b-89cc223500d1; institutionCode: NOC; collectionCode: DISCOLL; basisOfRecord: PreservedSpecimen
- **Type status:** Other material. **Occurrence:** catalogNumber: JC247-056-044; recordedBy: Amanda Serpell-Stevens | Tammy Horton; individualCount: 1; lifeStage: adult; occurrenceStatus: present; preparations: ethanol; occurrenceID: 3EAF517B-E625-5425-9FBE-E4B72F97BED7; **Taxon:** scientificNameID: urn:lsid:marinespecies.org:taxname:124773; scientificName: *Psychropotesbuglossa* E. Perrier, 1886; kingdom: Animalia; phylum: Echinodermata; class: Holothuroidea; order: Elasipodida; family: Psychropotidae; genus: Psychropotes; specificEpithet: *buglossa*; taxonRank: species; scientificNameAuthorship: E. Perrier, 1886; nomenclaturalCode: ICZN; **Location:** locationID: http://marineregions.org/mrgid/63025; higherGeographyID: http://vocab.nerc.ac.uk/collection/C19/current/; higherGeography: ATLANTIC OCEAN | NORTH ATLANTIC OCEAN | NORTHEAST ATLANTIC OCEAN (40W) | Porcupine Abyssal Plain; waterBody: NORTHEAST ATLANTIC OCEAN (40W); locality: Porcupine Abyssal Plain - Sustained Observatory; verbatimDepth: 4844 - 4846; minimumDepthInMeters: 4844; maximumDepthInMeters: 4846; maximumDistanceAboveSurfaceInMeters: 1.5; verbatimCoordinates: 49° 2.637' N, 016° 56.952' W to 48° 58.106' N, 016° 57.815' W; verbatimCoordinateSystem: degrees decimal minutes; verbatimSRS: EPSG:4326; geodeticDatum: EPSG:4326; **Identification:** identifiedBy: Amanda Serpell-Stevens; dateIdentified: 2023; **Event:** eventID: JC247-056; samplingProtocol: OTSB14; eventDate: 16/05/2023T22:52:00Z/17/05/2023T00:34:00Z; verbatimEventDate: 16/05/2023 22:52 UTC - 17/05/2023 00:34 UTC; fieldNumber: JC247-056; eventRemarks: Small, clean catch. Dist. Run = 8.464 km; **Record Level:** language: en; institutionID: https://www.gbif.org/grscicoll/institution/74ae2bc3-e5a8-443f-bc8b-89cc223500d1; institutionCode: NOC; collectionCode: DISCOLL; basisOfRecord: PreservedSpecimen
- **Type status:** Other material. **Occurrence:** catalogNumber: JC247-056-081; recordedBy: Amanda Serpell-Stevens | Tammy Horton; individualCount: 1; lifeStage: adult; occurrenceStatus: present; preparations: ethanol; occurrenceID: 3EC1CA04-4CCB-5BA3-9BB6-2E3DBDCF3DCA; **Taxon:** scientificNameID: urn:lsid:marinespecies.org:taxname:124773; scientificName: *Psychropotesbuglossa* E. Perrier, 1886; kingdom: Animalia; phylum: Echinodermata; class: Holothuroidea; order: Elasipodida; family: Psychropotidae; genus: Psychropotes; specificEpithet: *buglossa*; taxonRank: species; scientificNameAuthorship: E. Perrier, 1886; nomenclaturalCode: ICZN; **Location:** locationID: http://marineregions.org/mrgid/63025; higherGeographyID: http://vocab.nerc.ac.uk/collection/C19/current/; higherGeography: ATLANTIC OCEAN | NORTH ATLANTIC OCEAN | NORTHEAST ATLANTIC OCEAN (40W) | Porcupine Abyssal Plain; waterBody: NORTHEAST ATLANTIC OCEAN (40W); locality: Porcupine Abyssal Plain - Sustained Observatory; verbatimDepth: 4844 - 4846; minimumDepthInMeters: 4844; maximumDepthInMeters: 4846; maximumDistanceAboveSurfaceInMeters: 1.5; verbatimCoordinates: 49° 2.637' N, 016° 56.952' W to 48° 58.106' N, 016° 57.815' W; verbatimCoordinateSystem: degrees decimal minutes; verbatimSRS: EPSG:4326; geodeticDatum: EPSG:4326; **Identification:** identifiedBy: Amanda Serpell-Stevens; dateIdentified: 2023; **Event:** eventID: JC247-056; samplingProtocol: OTSB14; eventDate: 16/05/2023T22:52:00Z/17/05/2023T00:34:00Z; verbatimEventDate: 16/05/2023 22:52 UTC - 17/05/2023 00:34 UTC; fieldNumber: JC247-056; eventRemarks: Small, clean catch. Dist. Run = 8.464 km; **Record Level:** language: en; institutionID: https://www.gbif.org/grscicoll/institution/74ae2bc3-e5a8-443f-bc8b-89cc223500d1; institutionCode: NOC; collectionCode: DISCOLL; basisOfRecord: PreservedSpecimen
- **Type status:** Other material. **Occurrence:** catalogNumber: JC247-056-082; recordedBy: Amanda Serpell-Stevens | Tammy Horton; individualCount: 1; lifeStage: adult; occurrenceStatus: present; preparations: ethanol; associatedSequences: PP079630; occurrenceID: EECD1497-F16B-53CF-BA5A-A36BA6116938; **Taxon:** scientificNameID: urn:lsid:marinespecies.org:taxname:124773; scientificName: *Psychropotesbuglossa* E. Perrier, 1886; kingdom: Animalia; phylum: Echinodermata; class: Holothuroidea; order: Elasipodida; family: Psychropotidae; genus: Psychropotes; specificEpithet: *buglossa*; taxonRank: species; scientificNameAuthorship: E. Perrier, 1886; nomenclaturalCode: ICZN; **Location:** locationID: http://marineregions.org/mrgid/63025; higherGeographyID: http://vocab.nerc.ac.uk/collection/C19/current/; higherGeography: ATLANTIC OCEAN | NORTH ATLANTIC OCEAN | NORTHEAST ATLANTIC OCEAN (40W) | Porcupine Abyssal Plain; waterBody: NORTHEAST ATLANTIC OCEAN (40W); locality: Porcupine Abyssal Plain - Sustained Observatory; verbatimDepth: 4844 - 4846; minimumDepthInMeters: 4844; maximumDepthInMeters: 4846; maximumDistanceAboveSurfaceInMeters: 1.5; verbatimCoordinates: 49° 2.637' N, 016° 56.952' W to 48° 58.106' N, 016° 57.815' W; verbatimCoordinateSystem: degrees decimal minutes; verbatimSRS: EPSG:4326; geodeticDatum: EPSG:4326; **Identification:** identifiedBy: Amanda Serpell-Stevens; dateIdentified: 2023; **Event:** eventID: JC247-056; samplingProtocol: OTSB14; eventDate: 16/05/2023T22:52:00Z/17/05/2023T00:34:00Z; verbatimEventDate: 16/05/2023 22:52 UTC - 17/05/2023 00:34 UTC; fieldNumber: JC247-056; eventRemarks: Small, clean catch. Dist. Run = 8.464 km; **Record Level:** language: en; institutionID: https://www.gbif.org/grscicoll/institution/74ae2bc3-e5a8-443f-bc8b-89cc223500d1; institutionCode: NOC; collectionCode: DISCOLL; basisOfRecord: PreservedSpecimen
- **Type status:** Other material. **Occurrence:** catalogNumber: JC247-056-083; recordedBy: Amanda Serpell-Stevens | Tammy Horton; individualCount: 1; lifeStage: adult; occurrenceStatus: present; preparations: ethanol; occurrenceID: 82D0433F-0B9F-5100-8FDA-9A576ED91341; **Taxon:** scientificNameID: urn:lsid:marinespecies.org:taxname:124773; scientificName: *Psychropotesbuglossa* E. Perrier, 1886; kingdom: Animalia; phylum: Echinodermata; class: Holothuroidea; order: Elasipodida; family: Psychropotidae; genus: Psychropotes; specificEpithet: *buglossa*; taxonRank: species; scientificNameAuthorship: E. Perrier, 1886; nomenclaturalCode: ICZN; **Location:** locationID: http://marineregions.org/mrgid/63025; higherGeographyID: http://vocab.nerc.ac.uk/collection/C19/current/; higherGeography: ATLANTIC OCEAN | NORTH ATLANTIC OCEAN | NORTHEAST ATLANTIC OCEAN (40W) | Porcupine Abyssal Plain; waterBody: NORTHEAST ATLANTIC OCEAN (40W); locality: Porcupine Abyssal Plain - Sustained Observatory; verbatimDepth: 4844 - 4846; minimumDepthInMeters: 4844; maximumDepthInMeters: 4846; maximumDistanceAboveSurfaceInMeters: 1.5; verbatimCoordinates: 49° 2.637' N, 016° 56.952' W to 48° 58.106' N, 016° 57.815' W; verbatimCoordinateSystem: degrees decimal minutes; verbatimSRS: EPSG:4326; geodeticDatum: EPSG:4326; **Identification:** identifiedBy: Amanda Serpell-Stevens; dateIdentified: 2023; **Event:** eventID: JC247-056; samplingProtocol: OTSB14; eventDate: 16/05/2023T22:52:00Z/17/05/2023T00:34:00Z; verbatimEventDate: 16/05/2023 22:52 UTC - 17/05/2023 00:34 UTC; fieldNumber: JC247-056; eventRemarks: Small, clean catch. Dist. Run = 8.464 km; **Record Level:** language: en; institutionID: https://www.gbif.org/grscicoll/institution/74ae2bc3-e5a8-443f-bc8b-89cc223500d1; institutionCode: NOC; collectionCode: DISCOLL; basisOfRecord: PreservedSpecimen
- **Type status:** Other material. **Occurrence:** catalogNumber: JC247-051-011; recordedBy: Amanda Serpell-Stevens | Tammy Horton; individualCount: 1; lifeStage: adult; occurrenceStatus: present; preparations: ethanol; occurrenceID: 005A913C-752A-53BF-AFC2-BF1C89FE3B48; **Taxon:** scientificNameID: urn:lsid:marinespecies.org:taxname:124773; scientificName: *Psychropotesbuglossa* E. Perrier, 1886; kingdom: Animalia; phylum: Echinodermata; class: Holothuroidea; order: Elasipodida; family: Psychropotidae; genus: Psychropotes; specificEpithet: *buglossa*; taxonRank: species; scientificNameAuthorship: E. Perrier, 1886; nomenclaturalCode: ICZN; **Location:** locationID: http://marineregions.org/mrgid/63025; higherGeographyID: http://vocab.nerc.ac.uk/collection/C19/current/; higherGeography: ATLANTIC OCEAN | NORTH ATLANTIC OCEAN | NORTHEAST ATLANTIC OCEAN (40W) | Porcupine Abyssal Plain; waterBody: NORTHEAST ATLANTIC OCEAN (40W); locality: Porcupine Abyssal Plain - Sustained Observatory; verbatimDepth: 4843 - 4848; minimumDepthInMeters: 4843; maximumDepthInMeters: 4848; maximumDistanceAboveSurfaceInMeters: 1.5; verbatimCoordinates: 49° 5.435' N, 016° 53.023' W to 49° 01.021' N, 016° 57.875' W; verbatimCoordinateSystem: degrees decimal minutes; verbatimSRS: EPSG:4326; geodeticDatum: EPSG:4326; **Identification:** identifiedBy: Amanda Serpell-Stevens; dateIdentified: 2023; **Event:** eventID: JC247-051; samplingProtocol: OTSB14; eventDate: 17/05/2023T21:51:00Z/18/05/2023T01:00:00Z; verbatimEventDate: 17/05/2023 22:51 UTC - 18/05/2023 01:00 UTC; fieldNumber: JC247-051; eventRemarks: Moderate, clean catch. Dist. Run = 10.075 km; **Record Level:** language: en; institutionID: https://www.gbif.org/grscicoll/institution/74ae2bc3-e5a8-443f-bc8b-89cc223500d1; institutionCode: NOC; collectionCode: DISCOLL; basisOfRecord: PreservedSpecimen
- **Type status:** Other material. **Occurrence:** catalogNumber: JC247-051-079; recordedBy: Amanda Serpell-Stevens | Tammy Horton; individualCount: 1; lifeStage: adult; occurrenceStatus: present; preparations: ethanol; occurrenceID: CB0FE904-DDBC-531A-ADE1-3EBC95F13B9A; **Taxon:** scientificNameID: urn:lsid:marinespecies.org:taxname:124773; scientificName: *Psychropotesbuglossa* E. Perrier, 1886; kingdom: Animalia; phylum: Echinodermata; class: Holothuroidea; order: Elasipodida; family: Psychropotidae; genus: Psychropotes; specificEpithet: *buglossa*; taxonRank: species; scientificNameAuthorship: E. Perrier, 1886; nomenclaturalCode: ICZN; **Location:** locationID: http://marineregions.org/mrgid/63025; higherGeographyID: http://vocab.nerc.ac.uk/collection/C19/current/; higherGeography: ATLANTIC OCEAN | NORTH ATLANTIC OCEAN | NORTHEAST ATLANTIC OCEAN (40W) | Porcupine Abyssal Plain; waterBody: NORTHEAST ATLANTIC OCEAN (40W); locality: Porcupine Abyssal Plain - Sustained Observatory; verbatimDepth: 4843 - 4848; minimumDepthInMeters: 4843; maximumDepthInMeters: 4848; maximumDistanceAboveSurfaceInMeters: 1.5; verbatimCoordinates: 49° 5.435' N, 016° 53.023' W to 49° 01.021' N, 016° 57.875' W; verbatimCoordinateSystem: degrees decimal minutes; verbatimSRS: EPSG:4326; geodeticDatum: EPSG:4326; **Identification:** identifiedBy: Amanda Serpell-Stevens; dateIdentified: 2023; **Event:** eventID: JC247-051; samplingProtocol: OTSB14; eventDate: 17/05/2023T21:51:00Z/18/05/2023T01:00:00Z; verbatimEventDate: 17/05/2023 22:51 UTC - 18/05/2023 01:00 UTC; fieldNumber: JC247-051; eventRemarks: Moderate, clean catch. Dist. Run = 10.075 km; **Record Level:** language: en; institutionID: https://www.gbif.org/grscicoll/institution/74ae2bc3-e5a8-443f-bc8b-89cc223500d1; institutionCode: NOC; collectionCode: DISCOLL; basisOfRecord: PreservedSpecimen
- **Type status:** Other material. **Occurrence:** catalogNumber: JC247-051-033; recordedBy: Amanda Serpell-Stevens | Tammy Horton; individualCount: 1; lifeStage: adult; occurrenceStatus: present; preparations: ethanol; occurrenceID: 688EC9CB-D74F-5981-91C4-27F16FD729A7; **Taxon:** scientificNameID: urn:lsid:marinespecies.org:taxname:124773; scientificName: *Psychropotesbuglossa* E. Perrier, 1886; kingdom: Animalia; phylum: Echinodermata; class: Holothuroidea; order: Elasipodida; family: Psychropotidae; genus: Psychropotes; specificEpithet: *buglossa*; taxonRank: species; scientificNameAuthorship: E. Perrier, 1886; nomenclaturalCode: ICZN; **Location:** locationID: http://marineregions.org/mrgid/63025; higherGeographyID: http://vocab.nerc.ac.uk/collection/C19/current/; higherGeography: ATLANTIC OCEAN | NORTH ATLANTIC OCEAN | NORTHEAST ATLANTIC OCEAN (40W) | Porcupine Abyssal Plain; waterBody: NORTHEAST ATLANTIC OCEAN (40W); locality: Porcupine Abyssal Plain - Sustained Observatory; verbatimDepth: 4843 - 4848; minimumDepthInMeters: 4843; maximumDepthInMeters: 4848; maximumDistanceAboveSurfaceInMeters: 1.5; verbatimCoordinates: 49° 5.435' N, 016° 53.023' W to 49° 01.021' N, 016° 57.875' W; verbatimCoordinateSystem: degrees decimal minutes; verbatimSRS: EPSG:4326; geodeticDatum: EPSG:4326; **Identification:** identifiedBy: Amanda Serpell-Stevens; dateIdentified: 2023; **Event:** eventID: JC247-051; samplingProtocol: OTSB14; eventDate: 17/05/2023T21:51:00Z/18/05/2023T01:00:00Z; verbatimEventDate: 17/05/2023 22:51 UTC - 18/05/2023 01:00 UTC; fieldNumber: JC247-051; eventRemarks: Moderate, clean catch. Dist. Run = 10.075 km; **Record Level:** language: en; institutionID: https://www.gbif.org/grscicoll/institution/74ae2bc3-e5a8-443f-bc8b-89cc223500d1; institutionCode: NOC; collectionCode: DISCOLL; basisOfRecord: PreservedSpecimen
- **Type status:** Other material. **Occurrence:** catalogNumber: JC247-051-005; recordedBy: Amanda Serpell-Stevens | Tammy Horton; individualCount: 1; lifeStage: adult; occurrenceStatus: present; preparations: ethanol; occurrenceID: 482F656C-AEB3-5974-A3AA-04D7677DDEF4; **Taxon:** scientificNameID: urn:lsid:marinespecies.org:taxname:124773; scientificName: *Psychropotesbuglossa* E. Perrier, 1886; kingdom: Animalia; phylum: Echinodermata; class: Holothuroidea; order: Elasipodida; family: Psychropotidae; genus: Psychropotes; specificEpithet: *buglossa*; taxonRank: species; scientificNameAuthorship: E. Perrier, 1886; nomenclaturalCode: ICZN; **Location:** locationID: http://marineregions.org/mrgid/63025; higherGeographyID: http://vocab.nerc.ac.uk/collection/C19/current/; higherGeography: ATLANTIC OCEAN | NORTH ATLANTIC OCEAN | NORTHEAST ATLANTIC OCEAN (40W) | Porcupine Abyssal Plain; waterBody: NORTHEAST ATLANTIC OCEAN (40W); locality: Porcupine Abyssal Plain - Sustained Observatory; verbatimDepth: 4843 - 4848; minimumDepthInMeters: 4843; maximumDepthInMeters: 4848; maximumDistanceAboveSurfaceInMeters: 1.5; verbatimCoordinates: 49° 5.435' N, 016° 53.023' W to 49° 01.021' N, 016° 57.875' W; verbatimCoordinateSystem: degrees decimal minutes; verbatimSRS: EPSG:4326; geodeticDatum: EPSG:4326; **Identification:** identifiedBy: Amanda Serpell-Stevens; dateIdentified: 2023; **Event:** eventID: JC247-051; samplingProtocol: OTSB14; eventDate: 17/05/2023T21:51:00Z/18/05/2023T01:00:00Z; verbatimEventDate: 17/05/2023 22:51 UTC - 18/05/2023 01:00 UTC; fieldNumber: JC247-051; eventRemarks: Moderate, clean catch. Dist. Run = 10.075 km; **Record Level:** language: en; institutionID: https://www.gbif.org/grscicoll/institution/74ae2bc3-e5a8-443f-bc8b-89cc223500d1; institutionCode: NOC; collectionCode: DISCOLL; basisOfRecord: PreservedSpecimen
- **Type status:** Other material. **Occurrence:** catalogNumber: JC247-051-110; recordedBy: Amanda Serpell-Stevens | Tammy Horton; individualCount: 1; lifeStage: adult; occurrenceStatus: present; preparations: ethanol; occurrenceID: D66060DC-CCBA-5CC6-A333-4225C0752AD6; **Taxon:** scientificNameID: urn:lsid:marinespecies.org:taxname:124773; scientificName: *Psychropotesbuglossa* E. Perrier, 1886; kingdom: Animalia; phylum: Echinodermata; class: Holothuroidea; order: Elasipodida; family: Psychropotidae; genus: Psychropotes; specificEpithet: *buglossa*; taxonRank: species; scientificNameAuthorship: E. Perrier, 1886; nomenclaturalCode: ICZN; **Location:** locationID: http://marineregions.org/mrgid/63025; higherGeographyID: http://vocab.nerc.ac.uk/collection/C19/current/; higherGeography: ATLANTIC OCEAN | NORTH ATLANTIC OCEAN | NORTHEAST ATLANTIC OCEAN (40W) | Porcupine Abyssal Plain; waterBody: NORTHEAST ATLANTIC OCEAN (40W); locality: Porcupine Abyssal Plain - Sustained Observatory; verbatimDepth: 4843 - 4848; minimumDepthInMeters: 4843; maximumDepthInMeters: 4848; maximumDistanceAboveSurfaceInMeters: 1.5; verbatimCoordinates: 49° 5.435' N, 016° 53.023' W to 49° 01.021' N, 016° 57.875' W; verbatimCoordinateSystem: degrees decimal minutes; verbatimSRS: EPSG:4326; geodeticDatum: EPSG:4326; **Identification:** identifiedBy: Amanda Serpell-Stevens; dateIdentified: 2023; **Event:** eventID: JC247-051; samplingProtocol: OTSB14; eventDate: 17/05/2023T21:51:00Z/18/05/2023T01:00:00Z; verbatimEventDate: 17/05/2023 22:51 UTC - 18/05/2023 01:00 UTC; fieldNumber: JC247-051; eventRemarks: Moderate, clean catch. Dist. Run = 10.075 km; **Record Level:** language: en; institutionID: https://www.gbif.org/grscicoll/institution/74ae2bc3-e5a8-443f-bc8b-89cc223500d1; institutionCode: NOC; collectionCode: DISCOLL; basisOfRecord: PreservedSpecimen

#### Description

**Figure 48.**
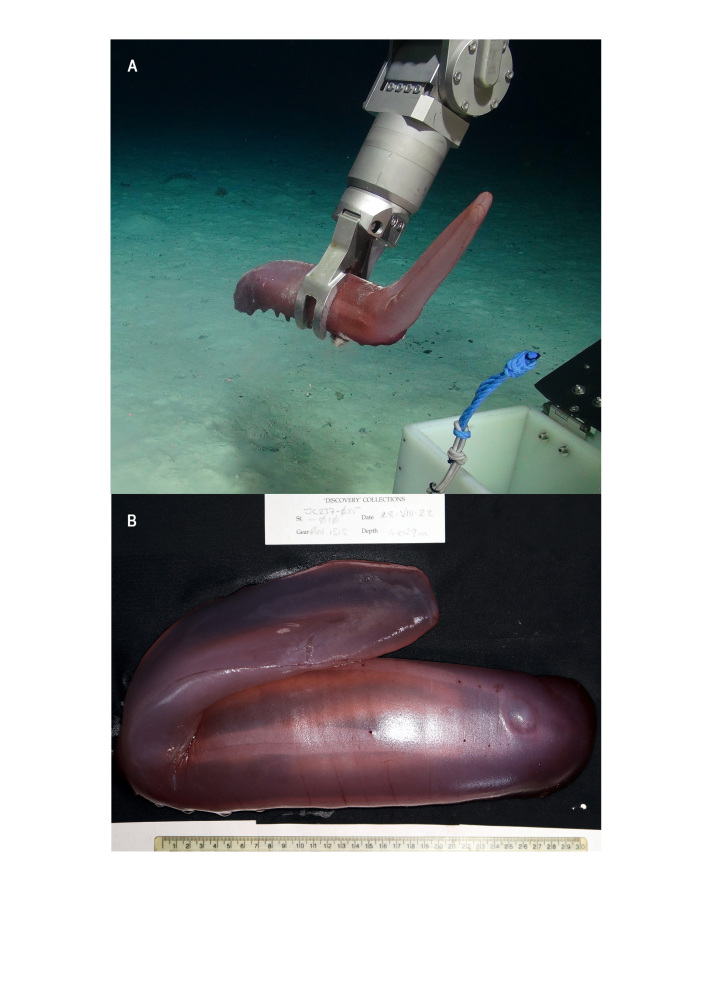
Photographs of specimen JC231-055-10 of *Psychropotesbuglossa* E. Perrier, 1886. **A.** Collection by ROV ISIS; **B.** Fresh specimen, prior to preservation.

Body length in preservative up to 230 mm. Fresh and preserved specimens dark violet (see Fig. 48). Body elongated and of even height along its length, slightly depressed at the anterior end. Tentacles 18, anterior and posterior brim distinct, widest at well-developed anterior end. Anterior brim consists of 13–17 pairs of small tube feet; posterior brim with 3–6 pairs of small tube feet. Usually 10 pairs of large ventrolateral tube feet (up to 11 pairs). Mid-ventral tube feet conspicuous in an alternating double row (between 13 and 20 pairs). Usually with 5 pairs of minute dorsal papillae. Unpaired dorsal appendage large, placed at the posterior end of the body, base almost as broad as the body, terminal end variable from acutely rounded to subsquare, length variable, but usually more than half the length of the body. Not considered a reliable character owing to damage in many specimens.

**Figure 49.**
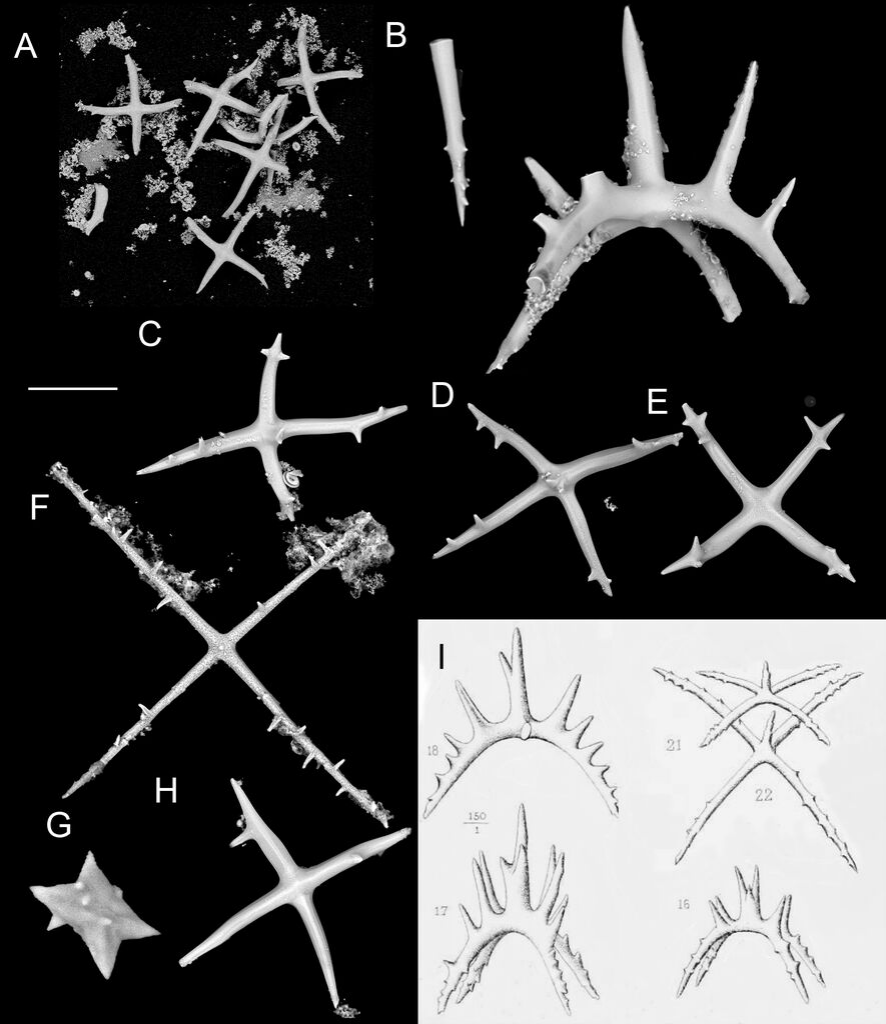
SEM images of ossicles (dermal deposits) prepared from specimen JC247-056-082 of *Psychropotesbuglossa* E. Perrier, 1886. **A.** Cluster of prepared ossicles from dorsal surface, showing additional broken fragments; **B.** Superficial layer dorsal ossicle with adjacent broken thorn; **C**–**E.** Typical dorsal ossicles; **F**–**G.** Dorsal appendage ossicles; **H.** Typical ventral ossicle; **I.** Extract from R. Perrier, 1902, Plate XX (https://www.biodiversitylibrary.org/page/44798009); figs. 16-18, superficial dermal layer dorsal ossicles; figs. 21-22, deeper dermal layer dorsal ossicles. Scale bars: 100 μm (A), 50 μm (B–H).

Dorsal ossicles (Fig. 49) two layers (superficial and deeper dermal) of densely arranged cruciform deposits. Ossicles in the superficial layer (Fig. 49B, I16–18) are large (225 μm), strongly convex, equally broad as high, bearing very long apical spines, with the central apical spine tallest, decreasing distally; central apical spine sometimes bifurcated or multiple apical spines present.

Dorsal ossicles in the deeper dermal layer (Fig. 49A, C–E and I21–22) smaller cruciform deposits around 180 μm, overall generally flat, with arms very slightly curved and somewhat irregular, with a small central spine, arms with occasional small spines. Dorsal appendage ossicles (Fig. 49F, G), cruciform with very thin long straight arms with small spines, relatively sparse in the tissue, 240–280 μm from tip to tip. Rarely present, small irregular solid ossicles, oblong, but angled with ornamenting thorns, around 75 μm. Ventral ossicles similar to those of the deeper dermal layer with arms often less ornamented (Fig. 49H).

#### Diagnosis

Tentacles 18, anterior brim 13–17 pairs of small tube feet, posterior brim 3–6 pairs of small tube feet, 10 (9–11) large ventrolateral tube feet, 13–20 pairs of mid-ventral tube feet in alternating double rows, 5 pairs of minute dorsal papillae, large unpaired dorsal appendage at posterior end of body. Dorsal superficial layer ossicles strongly convex, as broad as high, with very long apical spines, central apical spine tallest, sometimes bifurcated or multiple apical spines present. Modified after E. Perrier (1886) and R. Perrier (1902).

#### Distribution

Known from the type locality in the NE Atlantic between the Azores and France, Talisman Station 135, 43°15' N, 21°40' W, 4165 m and the Porcupine Abyssal Plain, 4840–4629 m (this study).

#### Taxon discussion

*Psychropotesbuglossa* was synonymised, along with 11 other nominal taxa, under *P.longicauda* by Hansen (1975), who considered this a cosmopolitan and variable species. This view prevailed until the DNA analyses by Gubili et al. (2017) revealed that the species comprised a number of different clades. The type locality of *P.longicauda* has been restricted to the Antarctic part of the Indian Ocean (Gebruk et al. 2020) and a number of names previously included as junior synonyms of *P.longicauda* have been re-established as separate lineages and valid species (Gebruk et al. 2020). In their revision, Gebruk et al. (2020) explicitly excluded the Atlantic species *P.buglossa*, *P.grimaldii* and *P.fucata* R. Perrier, 1896, as junior synonyms in the revised synonymy for *P.longicauda*, noting that further work was required to determine their validity.

*P.grimaldii* is herein synonymised with *P.buglossa* but the status of *P.fucata*, which was collected only once from the same type locality and depth as *P.buglossa*, requires further material from the North Atlantic to confirm its validity and it is, therefore, here designated *species inquirenda*. *P.raripes* known from the North Pacific, is included in Table 5 since it is genetically very similar and is nested in the same molecular clade as *P.buglossa* in Gubili et al. (2017).

*Psychropotesbuglossa* can be distinguished from *P.longicauda* by the larger number of pairs of ventrolateral tube feet (25–26 in *P.longicauda* vs. 10 in *P.buglossa*), mid-ventral tube feet and characters of the ossicles (Table 5, Fig. 49). *Psychropotesbuglossa* is differentiated from *P.fucata* by the smaller number of pairs of dorsal papillae (3) and by the shape of the ossicles which are not as highly thorned or as varied in form as those in *P.buglossa*. *Psychropotesbuglossa* is differentiated from *P.raripes* by the smaller number of pairs of ventrolateral tube feet (7-8) and by the shape of the ossicles.

*Psychropotesbuglossa* is considered here to be recognised as valid. Therefore, *P.buglossa* is re-established as the valid name for the common and abundant species occurring in the Atlantic at abyssal depths at the Porcupine Abyssal Plain Sustained Observatory (Hartman et al. 2021). COI sequences for two specimens examined here (JC231-082-EJC-05; JC247-056-082) were 100% identical to “haplotype 23” from the NE Atlantic, Porcupine Abyssal Plain of Gubili et al. (2017) in the region where the fragments overlap. Haplotype 23 was not reported outside of the NE Atlantic Ocean in their study. Sequences deposited in GenBank have accession numbers PP079630 and PP079631.

#### Notes

##### Methods

Samples used in this study were collected by means of OTSB 14 trawls deployed at the Porcupine Abyssal Plain at depths of 4629–4848 m during 2022 and 2023 on the RRS *James Cook* (Cruises JC231 (Hartman et al. 2022), JC237 (Huvenne 2024) and JC247 (Gates et al. 2023)) preserved in 95% ethanol. Tissue snips from two specimens (JC231-082-EJC-05; JC247-056-082, in the NOC Discovery Collections; http://grbio.org/cool/91qj-xx7i) were used for DNA extraction using QIAamp DNA Micro Kit (QIAGEN), following the manufacturer’s protocol. The cytochrome oxidase subunit I (COI primers COIef and COIer, Arndt et al. (1996), Miller et al. (2017)) and 12S ribosomal DNA (12S primers 12SA and 12SB,Janies et al. (2011), Miller et al. (2017)) were amplified using repliQa HiFi ToughMix from ThermoFisher, following the PCR programmes for COI and 12S from Thomas et al. (2020). Ossicles preparations were made using tissue from the respective area of the body of the specimen which was then dissolved using thin bleach to reveal the ossicles, visualised with a Hitachi tabletop SEM.

## Checklists

### Systematic notes and amendments

#### Placiphorella

Carpenter in Dall 1879

AB169C3F-C153-534F-9744-FFEBEF4E992D

https://www.marinespecies.org/aphia.php?p=taxdetails&id=138187

**Type species**: *Placiphorellavelata* (Carpenter in Dall, 1879), by original designation.
**Composition**: Fifteen valid species. *Placiphorellaalbitestae* Is. Taki, 1954, *Placiphorellaatlantica* (A. E. Verrill & S. I. Smith in Verrill, 1882), *Placiphorellablainvillii* (Broderip in Broderip & Sowerby I, 1832), *Placiphorellaborealijaponica* Saito & Okutani, 1989, *Placiphorellaborealis* Pilsbry, 1893, *Placiphorellahanselmani* R. N. Clark, 1994, *Placiphorellaisaotakii* Saito, Fujikura & Tsuchida, 2008, *Placiphorellalaurae* R. N. Clark, 2019, *Placiphorellamirabilis* R. N. Clark, 1994, *Placiphorellaokutanii* Saito, Fujikura & Tsuchida, 2008, *Placiphorellapacifica* S. S. Berry, 1919, *Placiphorellarufa* S. S. Berry, 1917, *Placiphorellastimpsoni* (A. Gould, 1859), *Placiphorellavelata* (Carpenter in Dall, 1879) and *Placiphorellamethanophila* Vončina, **sp. nov.**
**Diagnosis**: Small to medium size chitons, round to oval in outline. Valves very wide and short; lateral areas usually well defined. Articulamentum white to blue-green; head valve with 8–24 slits; intermediate valves with one slit per side; tail valve with one slit on each side (sometimes obsolete), separated by a caudal sinus. Girdle broadly extended anteriorly and bearing scaled bristles. Pallial fold modified anteriorly into numerous finger-like extensions (precephalic tentacles). Radula with tricuspid major lateral teeth. Edited after Clark (1994): 291, with modifications.

#### Lepetodrilus

McLean 1988

890CD7FB-5D76-5214-AA02-2825BB468B81

https://www.marinespecies.org/aphia.php?p=taxdetails&id=180907

**Type species**: *Lepetodriluspustulosus* McLean, 1988; by original designation.
**Composition**: Seventeen valid species, one of which has two valid subspecies. *Lepetodrilusatlanticus* Warén & Bouchet, 2001, *Lepetodrilusconcentricus* Linse, Roterman & Chen, 2019, *Lepetodriluscorrugatus* McLean, 1993, *Lepetodriluscristatus* McLean, 1988, *Lepetodriluselevatuselevatus* McLean, 1988, *Lepetodriluselevatusgalriftensis* McLean, 1988, *Lepetodrilusfijiensis* L. Beck in Chen & Sigwart, 2023, *Lepetodrilusfucensis* McLean, 1988, *Lepetodrilusgordensis* Johnson, Young, Jones, Warén & Vrijenhoek, 2006, *Lepetodrilusguaymasensis* McLean, 1988, *Lepetodrilusjaponicus* Okutani, Fujikura & Sasaki, 1993, *Lepetodrilusnux* (Okutani, Fujikura & Sasaki, 1993), *Lepetodrilusovalis* McLean, 1988, *Lepetodriluspustulosus* McLean, 1988, *Lepetodrilusschrolli* L. Beck, 1993, *Lepetodrilusshannonae* Warén & Bouchet, 2009, *Lepetodrilustevnianus* McLean, 1991 and *Lepetodrilusmarianae* Chen, Watanabe & Tsuda, **sp. nov.**

##### Diagnosis

See McLean (1988): 6–8.

#### Shinkailepas

Okutani, Saito & Hashimoto 1989

0357A1C1-CCE7-5D2E-A575-284B26015004

https://www.marinespecies.org/aphia.php?p=taxdetails&id=180861

**Type species**: *Shinkailepaskaikatensis* Okutani, Saito & Hashimoto, 1989; by original designation.
**Composition**: Six valid species. *Shinkailepasconspira* L. Beck in Chen & Sigwart, 2023, *Shinkailepaskaikatensis* Okutani, Saito & Hashimoto, 1989, *Shinkailepasmyojinensis* Sasaki, Okutani & Fujikura, 2003, *Shinkailepastollmanni* (L. Beck, 1992), *Shinkailepastufari* L. Beck, 1992 and *Shinkailepasgigas* Chen, Watanabe & Tsuda, **sp. nov.**

##### Diagnosis

See Fukumori et al. (2019), table 4.

#### Lyonsiellidae

Dall 1895

3F2817BA-5508-5C4F-97D8-79410BB91691

https://www.marinespecies.org/aphia.php?p=taxdetails&id=405866

##### Notes

Considered one of the rarest groups amongst Anomalodesmata, members of Lyonsiellidae are generally poorly sampled and under-represented in phylogenetic analyses (e.g., Combosch et al. 2017). Consequently, its family status has been debated for the past two decades. Dreyer et al. (2003) and Harper et al. (2006), mainly using a molecular approach, cast doubt on the monophyly of the Lyonsiellidae. Coan and Valentich-Scott (2012), for example, taking into account mainly shell features, considered lyonsiellids as a subfamily, Lyonsiellinae under family Verticordiidae. Crouch et al. (2021), in a budding-based calibrations approach, using a combination of molecular and morphological studies, corroborated the family level of Lyonsiellidae. More recently, however, Machado and Passos (2022) brought together for the first-time morphological characters from representatives of all Anomalodesmata families in a cladistic approach, recovering the Lyonsiellidae as polyphyletic.

#### Lyonsiella

G. O. Sars 1872

33ADA248-97CB-5136-A971-9E2FDD421026

https://www.marinespecies.org/aphia.php?p=taxdetails&id=138654

**Type species**: *Lyonsiellaabyssicola* (G. O. Sars, 1872).
**Composition**: Twenty valid species, of which 19 are databased (MolluscaBase 2024) and one, *Lyonsiellaillaesa* Machado & Sigwart, **sp. nov.**, is newly described here.
**Diagnosis**: Shell small to medium size (1 to ~ 25 mm in length), thin, usually inflated, quadrate to subrectangular, inequilateral, right valve generally larger than left valve with valve margins flexuous, slightly overlapping; outer surface granular or with spinules with sparse radial lirae or folds, frequently with adhering particles; hinge plate feeble, edentate, but anterior dorsal margin of left valve may be thickened; lithodesma elongate. Ctenidium reduced to few filaments aligned horizontally in pallial cavity, sometimes outer demibranch or its ascending lamella absent. Inhalant siphon cone-shaped with eversible capacity, usually surrounded by small and arborescent-shape tentacles. Taenioid muscles sometimes well developed. Usually hermaphrodite (after Allen and Turner (1974), Poutiers and Bernard (1995), Coan and Valentich-Scott (2012)).

#### Lepechinella

Stebbing 1908

CBC64E68-492A-5045-ACBD-4EDD00DD85A8

https://www.marinespecies.org/aphia.php?p=taxdetails&id=101578

**Type species**: *Lepechinellachrysotheras* Stebbing, 1908
**Composition**: Thirty-four valid species. *Lepechinellaarctica* Schellenberg, 1926, *Lepechinellaauca* J.L. Barnard, 1973, *Lepechinellabierii* J.L. Barnard, 1957, *Lepechinellacachi* J.L. Barnard, 1973, *Lepechinellacampensis* Sittrop & Serejo, 2009, *Lepechinellacetrata* J.L. Barnard, 1932, *Lepechinellachrysotheras* Stebbing, 1908, *Lepechinellacura* J.L. Barnard, 1973, *Lepechinellacurvispinosa* Pirlot, 1933, *Lepechinelladrygalskii* Schellenberg, 1926, *Lepechinellaechinata* (Chevreux, 1914), *Lepechinellaeupraxiella* J.L. Barnard, 1973, *Lepechinellagrimi* Thurston, 1980, *Lepechinellahelgii* Thurston, 1980, *Lepechinellahirsuta* Sittrop & Serejo, 2009, *Lepechinellahuaco* J.L. Barnard, 1973, *Lepechinellalaurensi* Sittrop & Serejo, 2009, *Lepechinellamadagascarensis* Ledoyer, 1983, *Lepechinellamanco* Barnard, 1973, *Lepechinellamonocuspidata* J.L. Barnard, 1961, *Lepechinellaocclo* J.L. Barnard, 1973, *Lepechinellapangola* J.L. Barnard, 1962, *Lepechinellaraua* J.L. Barnard, 1973, *Lepechinellasagamiensis* Gamó, 1981, *Lepechinellaschellenbergi* Stephensen, 1944, *Lepechinellaskarphedini* Thurston, 1980, *Lepechinellasucia* J.L. Barnard, 1961, *Lepechinellaturpis* J.L. Barnard, 1967, *Lepechinellauchu* J.L. Barnard, 1973, *Lepechinellaultraabyssalis* Birstein & N. Vinogradova, 1960, *Lepechinellavictoriae* Johansen & Vader, 2015, *Lepechinellavitrea* Kamenskaya, 1977, *Lepechinellawolffi* Dahl, 1959 and and *Lepechinellanaces* Lörz & Engel, **sp. nov.**

##### Diagnosis

See Sittrop and Serejo (2009): 475, followed by Johansen and Vader (2015): 4.

#### Cuniculomaera

Tandberg & Jażdżewska gen. nov.

A060D920-ECE7-5A4B-A320-D0BA48BC52FA

See the genus taxon treatment **proper**.

#### Bopyridae

Rafinesque 1815

3AD1AFD2-15C2-5A4F-943E-0AA6F27474F0

https://www.marinespecies.org/aphia.php?p=taxdetails&id=1195

##### Parasite of

Calcinid and pagurid hermit crabs (Anomura, Paguroidea). Including *Pseudionellapumulaensis* Williams & Landschoff, **sp. nov.**, there are now 98 species of bopyrid isopods known to parasitise hermit crabs worldwide as ecto- or endoparasites (Markham 2003, McDermott et al. 2010, An et al. 2016, Detorre et al. 2023, this work). As for many regions of the world, bopyrids have been poorly studied in South Africa (Williams and Boyko 2012). Based largely on the work of Barnard (1920), Barnard (1940), Barnard (1955)and Barnard 1958, there were 24 described species of epicaridean isopod parasites reported from crustacean hosts, but only two abdominal bopyrid species (and one hyperparasite) known from hermit crabs in South Africa (Bourdon 1972, Kensley 1978, Grygier 1981, Bourdon 1982, Markham 2005, Markham 2016). Host populations from South Africa are ripe for such studies on these parasites, not only for critical taxonomic work, but also research delving into their potential impacts on ecologically and commercially important hosts.

#### Pseudionella

Shiino 1949

486645BE-DB26-5DED-85AB-C673D52A9017

https://www.marinespecies.org/aphia.php?p=taxdetails&id=249236

**Type species**: *Pseudionellaattenuata* Shiino, 1949
**Composition**: Six species. *Pseudionellaakuaku* Boyko & Williams, 2001, *Pseudionellaattenuata* Shiino, 1949, *Pseudionelladeflexa* Bourdon, 1979, *Pseudionellamarkhami* (Adkison & Heard, 1978), *Pseudionellaspiropaguri* An, Li & Markham, 2013 and *Pseudionellapumulaensis* Williams & Landschoff, **sp. nov.**

##### Diagnosis

Adult female and male generic characters and character states are given by An et al. (2013): 567 and followed here.

#### Mastigoniscus

Lincoln 1985

93BF46E1-36B0-590F-BE26-52B19F1050EB

https://www.marinespecies.org/aphia.php?p=taxdetails&id=248939

**Type species**: *Mastigoniscuspistus* Lincoln, 1985
**Composition**: Fourteen species. *Mastigoniscusandeepi* Brökeland & Brandt, 2006, *Mastigoniscusconcavus* (Menzies & George, 1972), *Mastigoniscuselegans* Park, 2000, *Mastigoniscusgeneralis* (Menzies & George, 1972), *Mastigoniscusgratissimus* (Menzies & George, 1972), *Mastigoniscusgratus* (Menzies & George, 1972), *Mastigoniscuslatus* (Birstein, 1971), *Mastigoniscusmicrocephalus* (Gamó, 1989), *Mastigoniscuspistus* Lincoln, 1985, *Mastigoniscusplatovatus* Park, 2000, *Mastigoniscuspolygomphios* Brökeland & Brandt, 2006, *Mastigoniscuspseudoelegans* Brökeland & Brandt, 2006, *Mastigoniscusstenocephalus* Park, 2000, and *Mastigoniscusminimus* Wenz, Knauber & Riehl, **sp. nov.**

##### Notes

The most recent previous generic diagnosis for *Mastigoniscus* provided by Brökeland and Brandt (2006), page 86, contains several characters that do not apply to *M.latus* (Birstein, 1971), *M.microcephalus* (Gamó, 1989) and *M.minimus* Wenz, Knauber & Riehl, **sp. nov.** from the northwest Pacific Ocean. Character states listed by Brökeland and Brandt (2006) applying only to the remaining species of *Mastigoniscus* are: posterior body part (pereonites 5–7 and pleotelson) length exceeding length of anterior part; pleotelson posterolateral processes strongly projecting in males, shorter in females; pereopod VI carpus distodorsally with spine-like setae.

The similarities of *M.latus*, *M.microcephalus* and *M.minimus* Wenz, Knauber & Riehl, **sp. nov.**, as well as the geographic proximity of their occurrence, can be interpreted as evidence for a shared recent ancestry and may justify further studies on their relationships with the remaining species of *Mastigoniscus*. However, whether the observed similarities are chance similarities or justify the appraisal of a separate genus-level taxon or subgroup within *Mastigoniscus* requires a thorough systematic analysis, preferably including a broader genetic representation of *Mastigoniscus*.

##### Diagnosis

Head without rostral process; pereonites 5–7 and pleotelson tergites medially fused, sutures more or less distinct; pereonite 7 reduced in adults, short, with fully developed pereopods 7; antenna 2 article 3 dorsal projection distal margin serrated; male pleopods 1 and 2 large, covering most of the pleotelson ventral surface, pleopod 2 endopod elongate, article 1 curved backwards, article 2 much longer than article 1, forming slender copulatory filament; female operculum relatively smaller in relation to pleotelson than male operculum.

#### Macrostylis

G. O. Sars 1864

A163AE68-E0FF-54F6-AEB9-20611FB0AC21

https://www.marinespecies.org/aphia.php?p=taxdetails&id=118371

**Type species**: *Macrostylisspinifera* G.O. Sars, 1864
**Composition**: Ninety species, of which 87 are valid species, two are *nomina dubia* and *Macrostylispapandreas* Johannsen, Brandt & Riehl, **sp. nov.** is described here.
*Macrostylisabyssalis* Brandt, 2004, *Macrostylisabyssicola* Hansen, 1916, *Macrostylisaffinis* Birstein, 1963, *Macrostylisamaliae* Bober, Riehl, Henne & Brandt, 2017, *Macrostylisamplinexa* Mezhov, 1989, *Macrostylisangolensis* Brandt, 2004, *Macrostylisangulata* Mezhov, 1999, *Macrostylisantennamagna* Riehl & Brandt, 2010, *Macrostylisbelyaevi* Mezhov, 1989, *Macrostylisbifurcatus* Menzies, 1962, *Macrostylisbipunctatus* Menzies, 1962, *Macrostylisbirsteini* Mezhov, 1993, *Macrostyliscapito* Mezhov, 1989, *Macrostyliscaribbicus* Menzies, 1962, *Macrostyliscarinifera* Mezhov, 1988, *Macrostyliscerritus* Vey & Brix, 2009, *Macrostyliscompactus* Birstein, 1963, *Macrostylisconfinis* Mezhov, 2003, *Macrostyliscurticornis* Birstein, 1963, *Macrostylisdaniae* Bober, Riehl, Henne & Brandt, 2017, *Macrostylisdellacrocei* Aydogan, Wägele & Park, 2000, *Macrostylisdiatona* Mezhov, 2003, *Macrostylisdorsaetosa* Riehl, Wilson & Hessler, 2012, *Macrostyliselongata* Hansen, 1916, *Macrostylisemarginata* Mezhov, 2000, *Macrostylisexpolita* Mezhov, 2004, *Macrostylisfoveata* Mezhov, 2000, *Macrostylisfragosa* Mezhov, 2003, *Macrostylisgalatheae* Wolff, 1956, *Macrostylisgerdesi* (Brandt, 2002), *Macrostylisgestuosa* Mezhov, 1993, *Macrostylisgrandis* Birstein, 1970, *Macrostylishadalis* Wolff, 1956, *Macrostylishirsuticaudis* Menzies, 1962, *Macrostylislacunosa* Mezhov, 2004, *Macrostylislatifrons* Beddard, 1886, *Macrostylislatiuscula* Mezhov, 2004, *Macrostylislongifera* Menzies & George, 1972, *Macrostylislongipedis* Brandt, 2004, *Macrostylislongipes* Hansen, 1916, *Macrostylislongiremis* (Meinert, 1890), *Macrostylislongispinis* Brandt, 2004, *Macrostylislongissima* Mezhov, 1981, *Macrostylislongiuscula* Mezhov, 1981, *Macrostylislongula* Birstein, 1970, *Macrostylismagnifica* Wolff, 1962, *Macrostylismariana* Mezhov, 1993, *Macrostylismarionae* Kniesz, 2018, *Macrostylismatildae* Riehl & Brandt, 2013, *Macrostylismedioxima* Mezhov, 2003, *Macrostylismetallicola* Riehl & De Smet, 2020, *Macrostylismeteorae* Brandt, 2004, *Macrostylisminuscularia* Mezhov, 2003, *Macrostylisminutus* Menzies, 1962, *Macrostylispapillata* Riehl, Wilson & Hessler, 2012, *Macrostylispectorosa* Mezhov, 2003, *Macrostylispolaris* Malyutina & Kussakin, 1996, *Macrostylisporrecta* Mezhov, 1988, *Macrostylisprofundissima* Birstein, 1970, *Macrostylisprolixa* Mezhov, 2003, *Macrostylispumicosa* Mezhov, 2003, *Macrostylisquadratura* Birstein, 1970, *Macrostylisrectangulata* Mezhov, 1989, *Macrostylisreticulata* Birstein, 1963, *Macrostylisroaldi* Riehl & Kaiser, 2012, *Macrostylisrobusta* Brandt, 2004, *Macrostylissabinae* Bober, Riehl, Henne & Brandt, 2017, *Macrostylisscotti* Riehl & Brandt, 2013, *Macrostylissensitiva* Birstein, 1970, *Macrostylissetifer* Menzies, 1962, *Macrostylissetulosa* Mezhov, 1992, *Macrostylisspiniceps* Barnard, 1920, *Macrostylisspinifera* G. O. Sars, 1864, *Macrostylissqualida* Mezhov, 2000, *Macrostylisstrigosa* Mezhov, 1999, *Macrostylissubinermis* Hansen, 1916, *Macrostylistruncatex* Menzies, 1962, *Macrostylistumulosa* Mezhov, 1989, *Macrostylisuniformis* Riehl & Brandt, 2010, *Macrostylisurceolata* Mezhov, 1989, *Macrostylisvemae* Menzies, 1962, *Macrostylisvigorata* Mezhov, 1999, *Macrostylisvinogradovae* Mezhov, 1992, *Macrostylisviriosa* Mezhov, 1999, *Macrostylisvitjazi* Birstein, 1963, *Macrostyliswolffi* Mezhov, 1988, *Macrostyliszenkevitchi* Birstein, 1963 and *Macrostylispapandreas* Johannsen, Brandt & Riehl, **sp. nov.**

##### Diagnosis

Pereonal tagmosis 3:1:3 with Prn1–Prn3 forming a highly integrated fossosoma with various degrees of expression of segment borders, Prn4 standing out from all other segments with a pronounced anterior collum region and Prn5–Prn7 the flexibly articulated posterior tagma. Ceph prognathous. Oostegites only on Prn3 and 4. Ventral spines may be present to various degrees on Prn1–Prn7, never on Ceph or Plt. Plt with paired statocyst, Plp cavity posteriorly open, anus located inside caudal extension of Plp cavity. A1 articulation position anterodorsally, basal article orientation anterodorsally, flagellum aesthetasc number in adult male per article two to many, hypertrophy in adult males. A2 axis straight, article 1 (precoxa) and article 3 (basis) scale absent, article 6 length exceeding combined length of articles. Md without palp, with lateral seta approximately at location of palp articulation, right *lacinia mobilis* differentiated from spine row. P1–3 coxae disc-like. P2–3 ‘fossorial’ with an elongate ischium, merus and carpus, all with broadened margins and dorsal and ventral rows of robust setae, propodus slender and paucisetose. P3–4 orientated somewhat dorsally and often held in a lateral position. P3 ischium with dorsal lobe and prominent dorsal setation, with carpo-propodal joint rotation. P4 short. Male Plp1 medial and lateral lobes lateral to each other. Female PlpII distal pappose, long. Urp long, cylindrical or conical, endopod relatively long, exopod absent. After Riehl et al. (2014a).

#### Austroniscus

Vanhöffen 1914

C40B1EDD-B013-53C7-BE93-634F7286C9FE

https://www.marinespecies.org/aphia.php?p=taxdetails&id=118379

**Type species**: *Austroniscusovalis* Vanhöffen, 1914
**Composition**: Twelve species. *Austroniscusacutus* Birstein, 1970, *Austroniscusbrandtae* Kaiser, Stransky & Brix, 2023, *Austroniscuschelus* Kaiser & Brandt, 2007, *Austroniscuscoronatus* Schiecke & Modigh-Tota, 1976, *Austroniscusgroenlandicus* (Hansen, 1916), *Austroniscuskaramani* Birstein, 1962, *Austroniscusnorbi* Svavarsson, 1982, *Austroniscusobscurus* Kaiser & Brandt, 2007, *Austroniscusovalis* Vanhöffen, 1914, *Austroniscusrotundatus* Vanhöffen, 1914, *Austroniscusvinogradovi* (Gurjanova, 1950) and *Austroniscusindobathyasellus* Kaiser, Kniesz & Kihara, **sp. nov.**

##### Diagnosis

Following Kaiser et al. (2023): 415.

#### Apseudopsis

Norman 1899

A9B29DFC-822E-5357-92C4-62899FBB1BF3

https://www.marinespecies.org/aphia.php?p=taxdetails&id=136186

**Type species**: *Apseudopsisacutifrons* (Sars, 1882).
**Composition**: Twenty-six species. *Apseudopsisacutifrons* (Sars, 1882), *Apseudopsisadami* Esquete & Bamber in Esquete et al., 2012, *Apseudopsisannabensis* (Guţu, 2002), *Apseudopsisapocryphus* (Guţu, 2002), *Apseudopsisarguinensis* (Guţu, 2002), *Apseudopsisbacescui* (Guţu, 2002), *Apseudopsisbruneinigma* (Bamber, 1998), *Apseudopsiscaribbeanus* Guţu, 2006, *Apseudopsiscuanzanus* Bochert, 2012, *Apseudopsiselisae* (Bacescu, 1961), *Apseudopsiserythraeicus* (Bacescu, 1984), *Apseudopsisformosus* Carvalho, Pereira & Esquete in Carvalho et al., 2019, *Apseudopsisgabesi* Esquete in Esquete et al., 2019, *Apseudopsishastifrons* (Norman & Stebbing, 1886), *Apseudopsisisochelatus* Guţu, 2006, *Apseudopsislatreillii* (Milne-Edwards, 1828), *Apseudopsismediterraneus* (Bacescu, 1961), *Apseudopsisminimus* (Guţu, 2002), *Apseudopsisolimpiae* (Guţu, 1986), *Apseudopsisopisthoscolops* Bamber, Chatterjee & Marshall, 2012, *Apseudopsisostroumovi* Băcescu & Cărăuşu, 1947, *Apseudopsisrogi* Esquete in Esquete et al., 2016, *Apseudopsistridens* (Guţu, 2002), *Apseudopsistuski* (Błażewicz-Paszkowycz & Bamber, 2007), *Apseudopsisuncidigitatus* (Norman & Stebbing, 1886) and *Apseudopsisdaria* Esquete & Tato, **sp. nov**.

##### Diagnosis

The most recent diagnosis was provided by Guţu (2006): 61. The diagnosis is detailed, but vague, even contradictory and Guţu (2006) listed a number of *Apseudopsis* species that do not correspond to his own diagnosis. The genus needs revision.

#### Psychropotes

Théel 1882

A65C2F32-01BA-5D33-88A0-E45C32F3FEC4

https://www.marinespecies.org/aphia.php?p=taxdetails&id=123532

**Type species**: *Psychropoteslongicauda* Théel, 1882, by subsequent designation.
**Composition**: Twenty valid species and subspecies. *Psychropotesbelyaevi* Hansen, 1975, *Psychropotesbuglossa* E. Perrier, 1886, *Psychropotesdepressa* (Théel, 1882), *Psychropotesdubiosa* Ludwig, 1893, *Psychropotesdyscrita* (Clark, 1920), *Psychropotesfuscopurpurea* Théel, 1882, *Psychropoteshyalinus* Pawson, 1985, *Psychropoteslongicauda* Théel, 1882, *Psychropotesloveni* Théel, 1882, *Psychropotesminuta* Koehler & Vaney, 1905, *Psychropotesmirabilis* Hansen, 1975, *Psychropotesmonstrosa* Théel, 1882, *Psychropotesmoskalevi* Gebruk & Kremenetskaia in Gebruk et al., 2020, *Psychropotespawsoni* Gebruk & Kremenetskaia in Gebruk et al., 2020, *Psychropotesraripes* Ludwig, 1893, *Psychropotesscotiae* (Vaney, 1908), *Psychropotessemperiana* Théel, 1882, *Psychropotesverrucicaudatus* Xiao, Gong, Kou & Li, 2019, *Psychropotesverrucosa* (Ludwig, 1893) and *Psychropotesxenochromata* Rogacheva & Billett in Rogacheva et al., 2009.

##### Diagnosis

See Théel (1882): 96, followed by Gebruk et al. (2020): 2.

## Discussion

### Conclusions

This paper illustrates that collaboration on a consortium level is feasible in taxonomy, a field often fragmented by its focus on different taxa. By encompassing all animal phyla, marine geographical regions, depth zones and ecological settings, the Ocean Species Discoveries (OSD) publication series centres on describing new taxa. Consequently, OSD introduces a novel approach to the various existing methods (see, for example, Coleman (2003), Renner (2016), Vences (2020), Orr et al. (2021), Sigwart et al. (2023)), all aiming to expedite the too slow yet fundamental cataloguing of marine biodiversity (Mora et al. 2011, Appeltans et al. 2012, Bouchet et al. 2023). This effort is crucial for researchers, conservationists and communicators in response to the escalating global threats to ocean species (Orr et al. 2021, Wiens and Zelinka 2024).

Encouraged by the experiences with the compilation of this publication and the collaboration between the various contributing experts, we are already working on the next issue of Ocean Species Discoveries. As a natural next step, the integration with the taxonomic species description service of SOSA (Sigwart et al. 2023) is being pursued, so that OSD will be available as an optional publication medium for the customers of the SOSA Discovery Unit in the future. In the medium term, we are developing species-specific description templates, routines for practical laboratory work and the preparation of species description texts, as well as glossaries and ontologies for the standardisation and, in the long term, growing level of automation of taxonomic species descriptions. Against this background, OSD forms an experimental platform to advance the urgently needed acceleration of marine species description.

## Supplementary Material

XML Treatment for Placiphorella methanophila

XML Treatment for Lepetodrilus marianae

XML Treatment for Shinkailepas gigas

XML Treatment for Lyonsiella illaesa

XML Treatment for Lepechinella naces

XML Treatment for Cuniculomaera

XML Treatment for Cuniculomaera grata

XML Treatment for Pseudionella pumulaensis

XML Treatment for Mastigoniscus minimus

XML Treatment for Macrostylis papandreas

XML Treatment for Austroniscus indobathyasellus

XML Treatment for Apseudopsis daria

XML Treatment for Psychropotes buglossa

XML Treatment for Placiphorella

XML Treatment for Lepetodrilus

XML Treatment for Shinkailepas

XML Treatment for Lyonsiellidae

XML Treatment for Lyonsiella

XML Treatment for Lepechinella

XML Treatment for Cuniculomaera

XML Treatment for Bopyridae

XML Treatment for Pseudionella

XML Treatment for Mastigoniscus

XML Treatment for Macrostylis

XML Treatment for Austroniscus

XML Treatment for Apseudopsis

XML Treatment for Psychropotes

78714A98-BFD7-52C1-9109-50058F196321 10.3897/BDJ.12.e128431.suppl1 Supplementary material 1 Ocean Species Discoveries 1-12 habitat summary Data type table Brief description Summary of habitat, depth and substrate of the twelve species addressed in this work. File: oo_1044292.csv https://binary.pensoft.net/file/1044292 Senckenberg Ocean Species Alliance (SOSA), Angelika Brandt, Chong Chen, Laura Engel, Patricia Esquete, Tammy Horton, Anna M. Jażdżewska, Nele Johannsen, Stefanie Kaiser, Terue C. Kihara, Henry Knauber, Katharina Kniesz, Jannes Landschoff, Anne-Nina Lörz, Fabrizio M. Machado, Carlos A. Martínez-Muñoz, Torben Riehl, Amanda Serpell-Stevens, Julia D. Sigwart, Anne Helene S. Tandberg, Ramiro Tato, Miwako Tsuda, Katarzyna Vončina, Hiromi K.Watanabe, Christian Wenz, Jason D. Williams

## Figures and Tables

**Table 1.** Genetic distances between *Placiphorella* species collected from mitochondrial cytochrome oxidase subunit 1 (COI) partial gene pairwise comparisons.

**GenBank number**	**Taxon name**		**1**	**2**	**3**	**4**	**5**	**6**	**7**	**8**	**9**	**10**	**11**	**12**	**13**	**14**	**15**
PP133101	*Placiphorellamethanophila*	1															
OP759456	*Placiphorellastimpsoni*	2	13.5%														
LC718184	*Placiphorellastimpsoni*	3	13.0%	4.3%													
MG450351	*Placiphorellastimpsoni*	4	13.7%	3.0%	3.5%												
EF159591	*Placiphorellavelata*	5	11.3%	13.0%	11.3%	11.5%											
KJ574090	*Placiphorella* sp.	6	4.1%	13.9%	14.1%	14.3%	13.3%										
GU806074	Chitonida sp.	7	4.8%	13.7%	13.5%	14.1%	12.6%	5.0%									
GU806075	Chitonida sp.	8	10.7%	13.9%	14.1%	14.3%	12.6%	11.7%	10.2%								
GU806076	Chitonida sp.	9	9.6%	13.7%	13.9%	14.6%	12.4%	10.7%	9.1%	2.0%							
GU806077	Chitonida sp.	10	4.6%	13.9%	13.7%	14.3%	12.4%	4.8%	0.2%	10.4%	9.3%						
GU806078	Chitonida sp.	11	4.8%	13.7%	13.9%	13.7%	12.2%	5.0%	0.9%	10.2%	9.1%	0.7%					
GU806080	Chitonida sp.	12	4.6%	13.9%	14.1%	13.9%	12.4%	4.8%	1.1%	10.2%	9.1%	0.9%	0.7%				
GU806115	Chitonida sp.	13	4.8%	13.7%	13.5%	14.1%	12.6%	5.0%	0.0%	10.2%	9.1%	0.2%	0.9%	1.1%			
GU806116	Chitonida sp.	14	9.6%	13.7%	13.9%	14.6%	12.4%	10.7%	9.1%	2.0%	0.0%	9.3%	9.1%	9.1%	9.1%		
GU806118	Chitonida sp.	15	5.0%	14.1%	14.3%	14.1%	12.6%	5.2%	1.5%	10.4%	9.3%	1.3%	1.1%	0.4%	1.5%	9.3%	

**Table 2.** Shell length, width and height of the holotype and paratype lots 1–5 of *Lepetodrilusmarianae* Chen, Watanabe & Tsuda, **sp. nov.**

**Type status**	**Catalogue number**	**Shell length (mm)**	**Shell width (mm)**	**Shell height (mm)**
Holotype	SMF 373150	8.3	5.9	4.2
Paratype 1	NSMT-Mo 79482	6.1	4.9	3.6
Paratype 2	NSMT-Mo 79483	8.4	5.5	4.1
Paratype 3	MNHN-IM-2019-34806	5.6	4.2	3.1
Paratype 4	MNHN-IM-2023-431	8.0	5.8	3.7
Paratype 5	SMF 373151	8.5	6.1	4.5

**Table 3.** Shell length, width and height of the holotype and paratypes of *Shinkailepasgigas* Chen, Watanabe & Tsuda, **sp. nov.**

**Type status**	**Catalogue number**	**Shell length (mm)**	**Shell width (mm)**	**Shell height (mm)**
Holotype	SMF 373153	23.0	17.9	8.1
Paratype 1	NSMT-Mo 79486	21.5	17.2	8.2
Paratype 2	SMF 373154	19.1	15.0	8.2
Paratype 3	MNHN-IM-2019-34808	17.6	13.3	5.5
Paratype 4	NSMT-Mo 79487	Fragmented	Fragmented	Fragmented

**Table 4.** Characters and character states that separate *Lepechinellagrimi* Thurston, 1980, *Lepechinellaocclo* Barnard, 1973, *Lepechinellapangola* J.L. Barnard, 1962, and *Lepechinellavictoriae* Johansen & Vader, 2015 from *Lepechinellanaces* Lörz & Engel, **sp. nov.**

**Species**	**Body**	**Prn2**–**7**	**A1**	**A2**	**Rostrum**	**First cephalic tooth**	**C1**	**C7**	**U2**	**T**
*L.grimi* Thurston, 1980	Many dorsal/lateral spines	P2–3 with single upright tooth; P4–7 pointed posteriorly	Art1 of peduncle 0.25x of length of art2	Longer than body; peduncle art 4 is 0.8x art 5	Curved; 0.3x of length of art1 of A1	1.35x of length of rostrum	Not bifid, slipper-shaped	Posterior distal angle produced	Outer ramus 0.85x of length of inner ramus	Cleft 65% of length; each lobe with one long and two short spines subapically
*L.occlo* Barnard, 1973	Covered with large spines	Each with one small erect tooth	Art 1 of peduncle 0.5x of length of art2	Longer than body; peduncle art 4 and 5 nearly same length	Pointing upwards; 0.4x of length of art1 of A1	0.67x of length of rostrum	Not bifid, slipper-shaped	Posterior distal angle produced	Outer ramus slightly shorter than inner ramus	Cleft 65% of length; each lobe with one long apical spine
*L.pangola* Barnard, 1962	Naked	Each with one tooth; anterior dorsal teeth rudimentary; posterior teeth of small to medium size	-	Shorter than body	Straight; around 0.5x of length of art1 of A1	As long as rostrum	Not bifid, slightly truncate	Posterior distal angle produced	Rami around same length	Cleft nearly 50% of length; each lobe with apical spine of unknown length
*L.victoriae* Johansen & Vader, 2015	Sparsely covered with spines	Each with one long tooth	Art1 of peduncle 0.5x of length of art2	Longer than body; peduncle 4 is 0.6x art 5	Straight; 0.5x of length of art1 of A1	As long as rostrum	Bifid	Posterior distal angle produced	Outer ramus is 0.7x of length of inner ramus	Cleft 40% of length; each lobe with one marginal and one long apical spine
*L.naces* Lorz & Engel, sp. nov.	Setose, some dorsal/lateral spines	Carinae, each segment with one subacute weak tooth pointed posteriorly	Art1 of peduncle 0.5x of length of art2	Shorter than body; peduncle art 4 is 0.6x art 5	Slightly curved; 0.2x of length of art1 of A1	Longer than rostrum	Not bifid, slipper-shaped	Posterior distal angle rounded	Outer ramus slightly shorter than inner ramus	Cleft 30% of length; each lobe with one long apical spine

**Table 5.** Locality and morphometric data for *Psychropotes* specimens examined or extracted from literature. Ventrolateral, mid-ventral, anterior and posterior tube feet and dorsal papillae counts are in pairs. Some specimens were damaged and where characters could not be confidently counted, these are indicated by an hyphen (-). *Ventral area greatly folded and tube feet hard to find. **Posterior brim count possibly included in ventrolateral tube feet count.

**Species**	**Specimen**	**Material**	**Depth (m)**	**Locality**	**Sole Length (mm)**	**Preserved Weight (g)**	**Tentacles**	**Ventrolateral**	**Midventral**	**Anterior brim**	Posterior brim	Dorsal papillae	Ossicle/DNA prep
*Psychropotesbuglossa*	JC231-082-012	other material	4840 – 4844	Porcupine Abyssal Plain, North Atlantic	163	124.7	18	10	>17	17	5	>4	–
*Psychropotesbuglossa*	JC231-082-EJC05	other material	"	Porcupine Abyssal Plain, North Atlantic	151	159.4	18	10	20	19	4	5	N/Y
*Psychropotesbuglossa*	JC231-086-027	other material	4838 – 4841	Porcupine Abyssal Plain, North Atlantic	153	164.6	18	9	17	16	–	–	–
*Psychropotesbuglossa*	JC237-055-10	other material	4629	Porcupine Abyssal Plain, North Atlantic	257	376.9	18	10	14	13	4	5	N/Y
*Psychropotesbuglossa*	JC247-056-044	other material	4844 – 4846	Porcupine Abyssal Plain, North Atlantic	195	227.2	18	10	16	13	4	5	–
*Psychropotesbuglossa*	JC247-056-081	other material	"	Porcupine Abyssal Plain, North Atlantic	181	133.1	18	9	20	16	5	5 or 6	–
*Psychropotesbuglossa*	JC247-056-082	other material	"	Porcupine Abyssal Plain, North Atlantic	157	103.8	18	11	13	17	3	5	Y/Y
*Psychropotesbuglossa*	JC247-056-083	other material	"	Porcupine Abyssal Plain, North Atlantic	148	94.5	18	10	18	15	4	5	N/Y
*Psychropotesbuglossa*	JC247-051-011	other material	4843 – 4848	Porcupine Abyssal Plain, North Atlantic	166	187.3	18	10	20	13	6	–	–
*Psychropotesbuglossa*	JC247-051-079	other material	"	Porcupine Abyssal Plain, North Atlantic	161	118.5	18	10	18	15	6	–	–
*Psychropotesbuglossa*	JC247-051-033	other material	"	Porcupine Abyssal Plain, North Atlantic	170	115.5	18	10	21	15	4	5	–
*Psychropotesbuglossa*	JC247-051-005	other material	"	Porcupine Abyssal Plain, North Atlantic	191	187.3	17	10	12*	15	5	5	–
*Psychropotesbuglossa*	JC247-051-110	other material	"	Porcupine Abyssal Plain, North Atlantic	159	135.7	18	10	16	14	5	5	Y/N
*Psychropotesbuglossa*		Syntype series average	4165	43° 15' N, 21° 40' W	118-204	–	18	14-16**	13-20	14	–	4-7	–
*Psychropotesfucata*		Holotype	4165	43° 15' N, 21° 40' W	147	–	18	15-16**	13-14	–	–	3	–
*Psychropotesgrimaldii*		Holotype	4020	38° 09' N, 11° 36' W	140	–	18	12	–	–	–	–	–
*Psychropotesraripes*	USNM 18173	Holotype	2800	01° 07' N, 80° 21' W	175	–	18	7-8 (10)	21	18-20	6-7	5	–
*Psychropoteslongicauda*	NHM 1883.6.18.58	Type series	3268	53° 55' S, 108° 35' E	140–145	–	18	24	27	–	–	5	–
